# *BRCA2* deficiency is a potential driver for human primary ovarian insufficiency

**DOI:** 10.1038/s41419-019-1720-0

**Published:** 2019-06-17

**Authors:** Yilong Miao, Pan Wang, Bingteng Xie, Mo Yang, Sen Li, Zhaokang Cui, Yong Fan, Mo Li, Bo Xiong

**Affiliations:** 1 College of Animal Science and Technology, Nanjing Agricultural University, 210095 Nanjing, China; 2 Center for Reproductive Medicine, Peking University Third Hospital, 100191 Beijing, China; 3 Reproductive Medicine Center, Guangdong Second Provincial General Hospital, 510317 Guangzhou, China; 4 Key Laboratory for Major Obstetric Diseases of Guangdong Province, The Third Affiliated Hospital of Guangzhou Medical University, 510150 Guangzhou, China

**Keywords:** Infertility, Disease model

## Abstract

Reproductive problem has been one of the top issues for women health worldwide in recent decades. As a typical female disease, primary ovarian insufficiency (POI) results in a loss of ovarian follicles and oocytes that thus destroys women fertility. However, due to the complex of POI etiology and rare resource of human POI oocytes, few biomarkers have been identified in clinics and no effective strategy could be applied to treat POI patients. In the search of possible association between DNA damage and POI by Smart-Seq2 and RT^2^ profiler PCR array, we find that *BRCA2*, a core DNA repair gene for homologous recombination shows significantly lower expression in two POI patient oocytes. In line with this, we generated oocyte-specific knockout mouse model driven by *Gdf9-Cre*. The *Brca2*-deficient mice are infertile because of the arrested follicle development and defective oocyte quality caused by the accumulation of DNA damage. Notably, ectopic expression of Brca2 in *Brca2*-deficient oocytes could partially restore the oocyte maturation and chromosome stability. Collectively, our data assign a definite deficiency to *BRCA2* as a POI driver during follicle development and oocyte maturation, and provide a potential fertility treatment strategy for POI patients induced by *BRCA2* deficiency.

## Introduction

Primary ovarian insufficiency (POI), also known as premature ovarian failure or premature menopause, is defined as a loss of ovarian function before the age of 40 and diagnosed by elevated serum follicle-stimulating hormone (FSH) levels (>40 IU/L), which accounts for one major cause of female infertility^1,2^. POI relevance is continuously growing because of the increasing number of women desiring conception beyond 30 years of age, when POI prevalence is >1%^3^. Potential etiologies for POI can be divided into genetic, autoimmune, and iatrogenic categories. Unfortunately, for most patients presenting with POI, the cause remains unexplained^4^. On molecular level, POI often has a genetic basis with over 50 genes in which mutations can be causative and many other genes that can be implicated^5^. These genes can affect various processes such as gonadal development, meiotic progress, hormonal signaling, immune function, and metabolism. Among the genes associated with POI, only a few (such as *FMR1* premutation, *BMP15*, *GDF9*, and *FSHR*) have been applied as diagnostic biomarkers^6^, and scientific community and patients ask for more research before using other genes as a routine tool. An understanding of general ovarian biology provides a framework for interpreting the etiology of POI.

Substantial evidence has revealed that DNA damage in our bodies is one of the leading causes for diseases such as tumor, neurodegenerative disorder, and immune deficiency^7^. Of note, every cell generates up to 10^5^ DNA lesions per day that are induced by exogenous physical agents, spontaneous chemical reactions, and products of endogenous metabolism^8^. Most of the damages including crosslinks, base mismatches, single strand breaks, and double-strand breaks (DSBs) could be sensed and fixed timely by the DNA damage response (DDR) system^9,10^. Among these damages, DSBs are the most deleterious lesion because no intact complementary strand is present as a template for the repair. Failure of DSBs repair results in genomic instability, exposing the local tissues to high risk^11–13^. For ovary, using gene knockout models in mice, scientists have shown that mutations in various DDR genes cause the early exhaustion of the follicle pool, which mimics the phenotypes of POI such as a developmental reduction in the number of primordial follicles, an accelerated rate of primordial follicle recruitment, and increased atresia or destruction of growing follicles^14–16^. However, since POI patients have few oocytes, it is very difficult to obtain clinical resource to decipher this disease, and thus direct evidence that deficiency of DNA damage response factors causes clinic POI is still absent.

In this report, we systematically analyze the expression patterns of 84 DNA damage response factors in single oocyte from 30 POI patients combined by Smart-Seq2 and RT^2^ profiler PCR array. Notably, oocytes from two patients express significantly lower *BRCA2*, a core gene for repair DSBs by homologous recombination (HR). This observation is corroborated by the finding that *Brca2*-deficient mice are infertile and exhibit defective follicle growth and oocyte development. We further demonstrate that recovery of Brca2 protein level in *Brca2*-deficient oocytes could restore the meiotic failure during in vitro maturation. Thus, our findings not only define the deficiency of *BRCA2* as a POI driver during ovarian development, but also give a clinical implication for improving the oocyte quality of POI patients caused by *BRCA2* deficiency, which in turn contributes to increase the female fertility.

## Results

### Downregulation of BRCA2 in human POI oocytes

**Fig. 1:**
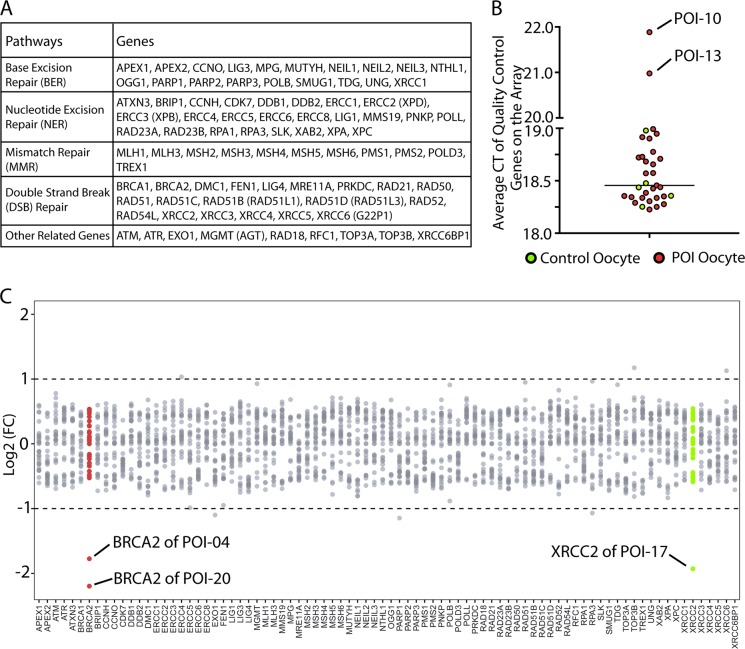
Expression level of DNA damage response genes in human POI oocytes. **a** The gene panel on the RT^2^ Profiler PCR Array. These genes are crucial factors in BER, NER, MMR, DSB repair, or other relative pathways in DNA damage response. **b** Quality control test of the whole panel of the array. Five control genes (ACTB, B2M, GAPDH, HPRT1, and RPLPO) were contained on the array as quality control. Average CT value of the control genes were summarized from real-time qPCR of the array. All the included oocytes showed qualified CT value except oocyte POI-10 and oocyte POI-13. These two oocytes were thus excluded from the subsequent experiments. **c** Expression level of the 84 DNA damage response genes among the individual human oocytes. POI-04 and POI-20 oocytes showed significantly lower expression of *BRCA2*, while POI-17 oocyte expressed lower *XRCC2*

To investigate potential involvement of DDR network in POI etiology, we began the experiment with clinical samples of oocytes from POI patients. These oocytes were collected from POI women who underwent assisted reproductive technology and failed in vitro fertilization (IVF). Five normal oocytes obtained from healthy donors were used as controls. All oocytes used in this study were received under approval of the Institutional Medical and Ethical Review Committee of The Third Affiliated Hospital of Guangzhou Medical University. During the full course of oocyte selection, 32 oocytes from 30 POI patients were harvested in total. Among them, 3 POI oocytes became dead during in vitro culture, while one POI oocyte failed in the following test of single cell library assessment (GAPDH quality control before running array). And thus 28 POI oocytes were used for subsequent experiments. Full information of the oocytes was summarized in Table S1. Due to rare resource of the samples, we strategized to operate these oocytes in single cell experiments. Single oocyte cDNA library was prepared as the previous reports^17,18^. RT^2^ Profiler PCR Array (Qiagen) was employed to assess the expression pattern of DDR factors. This array integrated 84 core DDR genes including ATM, FEN1, Ligase family, RAD family, etc (Fig. 1a and Table S2). cDNA from the 33 human oocytes (28 POI oocytes and 5 control oocytes) were loaded onto the array, respectively. Five control genes (ACTB, B2M, GAPDH, HPRT1, and RPLPO) were contained on the array as the quality control. Among all the 33 oocytes, 2 oocytes (POI-10 and POI-13) showed turbulent expression of the control genes and were excluded (Fig. 1b). Therefore, 31 oocytes (26 POI oocytes and 5 control oocytes) were finally included in the study and their detailed mRNA expressions were provided in Table S3.

On the array, we found two POI oocytes (POI-04 and POI-20) showed significantly lower expression of *BRCA2* compared with the control. In addition, the expression of another DNA repair gene *XRCC2* was obviously decreased in another POI oocyte (POI-17) (Fig. 1c). The expression variations of the other 82 genes could almost be clustered to 0.5–2 fold difference among the oocytes. (Fig. 1c). As *BRCA2* is a core factor in DDR^7,19,20^, we focused on this gene in our study.

### Brca2 in oocytes is essential for female fertility

**Fig. 2:**
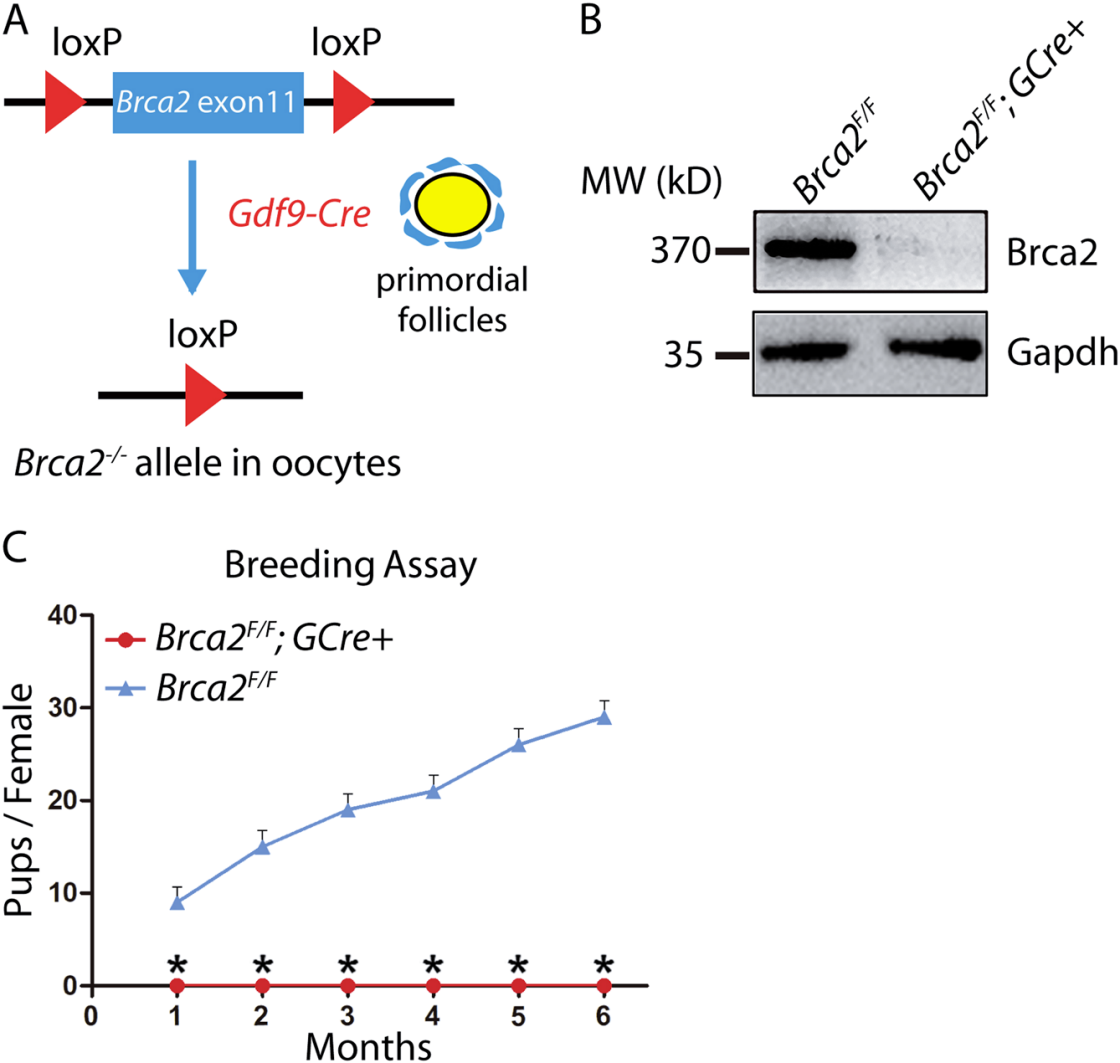
Deletion of *Brca2* in oocytes leads to female infertility. **a** Schematic illustration of deletion of *Brca2* exons and creation of *Brca2* knockout allele by *Gdf9-Cre*-mediated recombination in oocytes. **b** Immunoblotting analysis of Brca2 protein expression in control and *Brca2*-deficient oocytes. The amount of Gapdh was used as an internal control. Molecular mass is given in kilodaltons. Two hundered germinal vesicle oocytes isolated from ovaries of 2-month-old mice were used for each lane of the blots. For each experiment, at least five mice of each genotype were used. **c** Comparison of breeding between control and *Brca2*-deficient females. The female mice were cocaged with fertile male mice. The breeding assay was recorded for at least 6 months

To validate the link between BRCA2 deficiency and POI, we generated mutant mice in which the *Brca2* gene was deleted in oocytes of primordial and further developed follicles. This was achieved by crossing *Brca2*^*loxP/loxP*^ mice with transgenic mice expressing *growth differentiation factor 9* (*Gdf-9*) promoter-mediated Cre recombinase (Fig. 2a). In *Gdf9-Cre* mice, Cre is specifically expressed in oocytes of primordial follicles and later stage follicles since postnatal day 3^21,22^. By immunoblotting analysis, we found that protein expression of Brca2 was almost completely absent in germinal vesicle (GV) oocytes from *Brca2*^*F/F*^;*Gdf9-Cre* females (Fig. 2b), confirming that oocyte-specific knockout of *Brca2* was successful.

To test if the deletion of *Brca2* has an effect on the female fertility, we performed a breeding assay by mating control (*Brca2*^*F/F*^) or *Brca2*-deficient (*Brca2*^*F/F*^;*Gdf9-Cre*) female mice with wild-type males of proven fertility for 6 months. As shown in Fig. 2c, *Brca2*-deficient female mice exhibited completely infertile, indicating that Brca2 is essential for female fertility.

### Brca2 in oocytes is required for follicle and ovary development

**Fig. 3:**
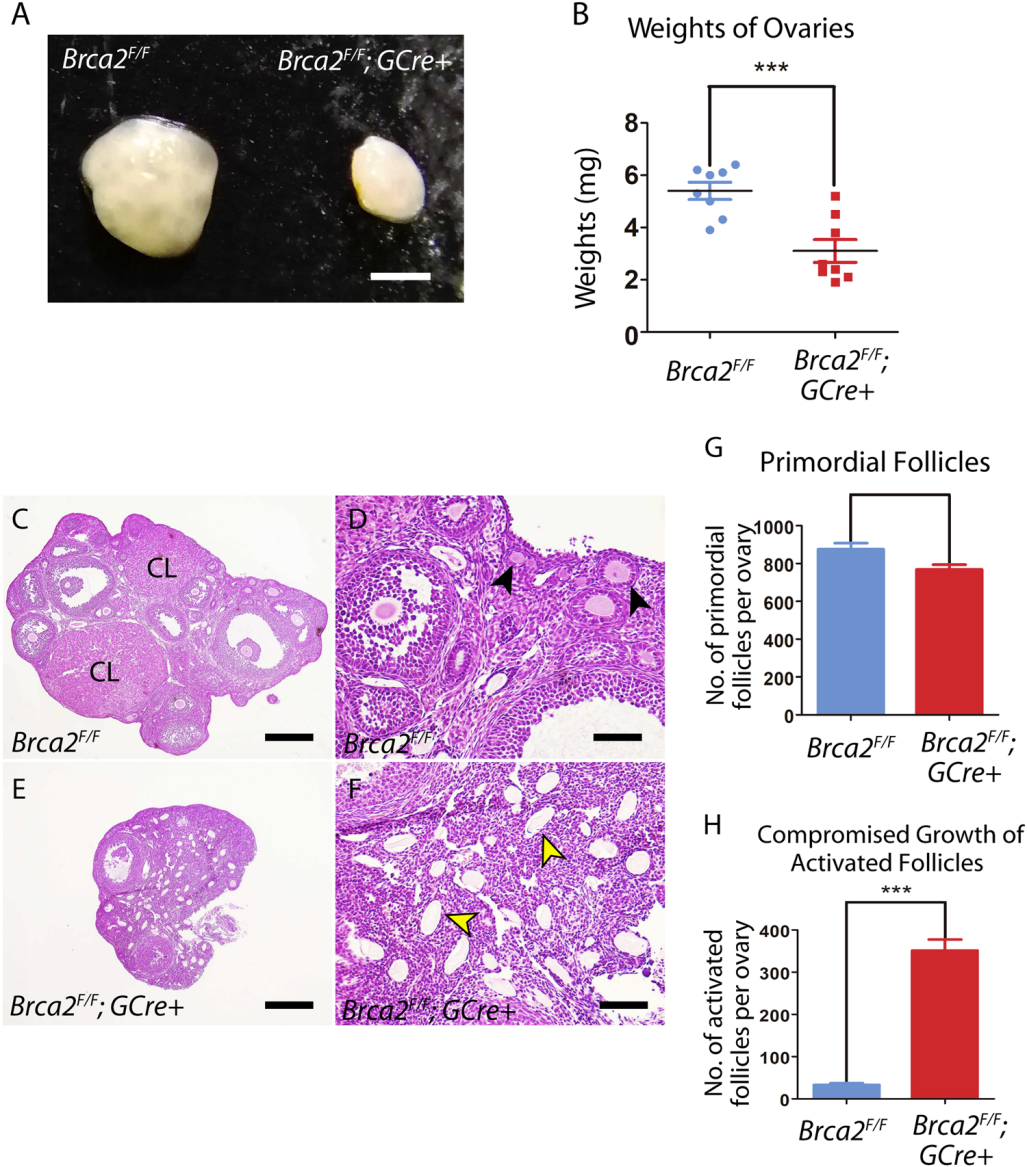
Deletion of *Brca2* in oocytes impairs ovarian development. **a** Representative images of ovarian size from control and *Brca2*-deficient mice. Scale bar, 200 μm. **b** Average weights of ovaries obtained from control and *Brca2*-deficient females. Data are shown as mean ± SEM. **c**–**f** Histology of ovarian sections from young adult control and *Brca2*-deficient females. Ovaries from 2-month-old mice were embedded in paraffin, and sections of 8 μm thickness were prepared and stained with H&E. Black arrows in (**d**) show the growing follicles at different developmental stages; yellow arrows in (**f**) indicate the developmentally arrested follicles with degenerating oocytes. CL, corpus luteum. Scale bars in (**c**) and (**e**), 250 μm; scale bars in (**d**) and (**f**), 50 μm. **g** Quantification analysis of primordial follicles between control and *Brca2*-deficient ovaries. **h** Quantification analysis of activated follicles between control and *Brca2*-deficient ovaries. *Brca2*-deficient ovary contained a significantly increased number of activated follicles with developmental arrest and degraded oocytes compared with the control ovary

To explore the reason leading to the infertility, we observed the morphology of ovaries from control and *Brca2*-deficient female mice. We found that the size of ovaries from young adult mutant mice (2-month-old) was apparently smaller than those from control mice (Fig. 3a). Also, the quantitation analysis of the ovary weight showed that mutant ovaries were dramatically lighter compared to controls, suggesting that loss of Brca2 impairs the ovarian development (Fig. 3b).

To further define the developmental changes of ovaries in the absence of Brca2, we assessed the histological sections of ovaries from mutant mice at 2 months of age. The histological analysis displayed that a majority of follicular structures were compromised, with many degenerating oocytes in transient follicles being seen. Even, some of them were devoid of oocytes and follicles (Fig. 3e, f). In comparison, ovaries from control mice (Fig. 3c, d) contained the growing follicles at different developmental stages and corpora lutea (CL). The quantification analysis of ovarian follicles revealed the number of primordial follicles was comparable between control and mutant ovaries (Fig. 3g). However, a significantly increased number of activated follicles with developmental arrest and degraded oocytes was present in the mutant ovaries compared to controls (Fig. 3h). Collectively, our data demonstrate that the defective follicular development, especially the compromised growth of activated follicles accounts for the infertility of *Brca2*-deficient female mice.

### Brca2 in oocytes is indispensable for oocyte development during meiotic progression

**Fig. 4:**
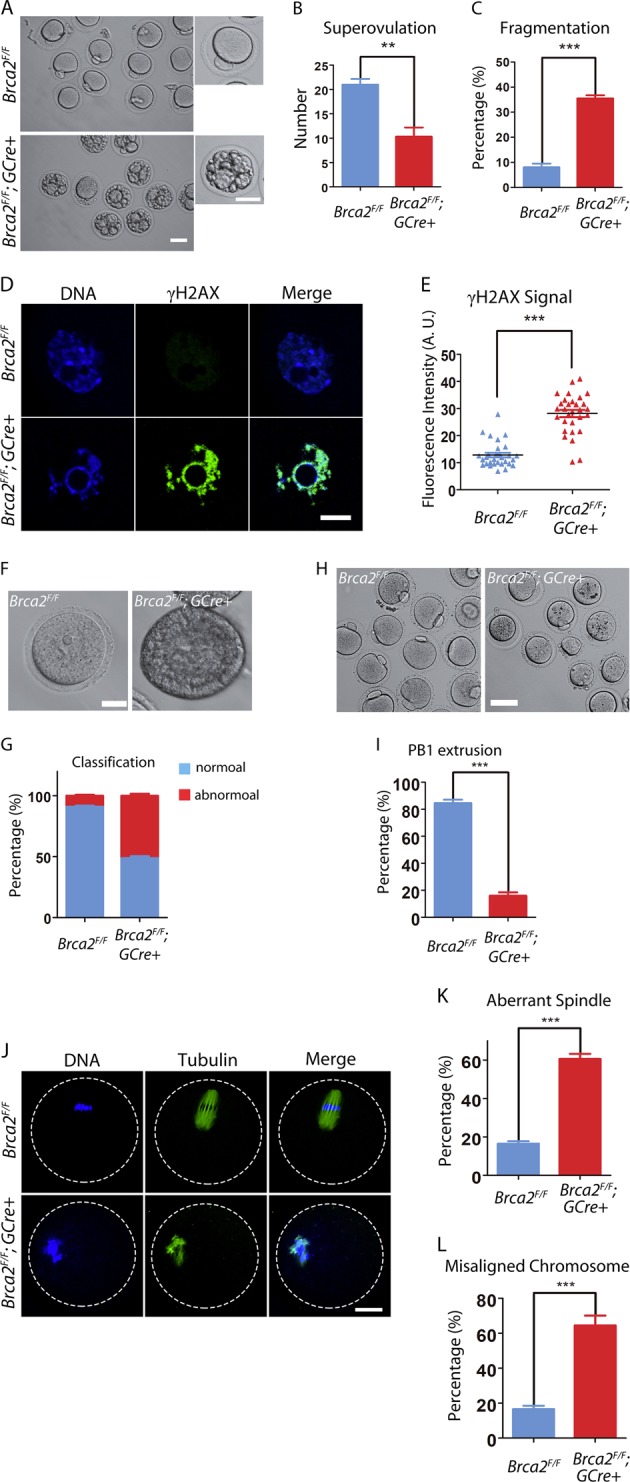
Deletion of *Brca2* compromises oocyte meiotic maturation. **a** Representative images of in vivo matured eggs from control and *Brca2*-deficient mice. Scale bar, 20μm. **b** The number of eggs obtained from control and *Brca2*-deficient mice. **c** The rate of fragmented eggs retrieved from control and *Brca2*-deficient mice. **d** Immunofluorescence of phosphorylated H2AX (γH2AX) in control and *Brca2*-deficient oocytes. **e** Fluorescence intensity of γH2AX signal in control and *Brca2*-deficient oocytes were summarized. **f** Representative images of GV oocytes obtained from control and *Brca2*-deficient ovaries. Scale bar, 20 μm. **g** The rate of developmentally arrested oocytes in control and *Brca2*-deficient mice. **h** Representative images of MII oocytes matured in vitro from control and *Brca2*-deficient GV oocytes. Scale bar, 20 μm. **i** The rate of first polar body extrusion of control and *Brca2*-deficient oocytes matured in vitro. **j** Representative images of spindle morphology and chromosome alignment in control and *Brca2*-deficient oocytes after in vitro maturation. Oocytes were immunostained with anti-α-tubulin-FITC antibody to visualize spindles and counterstained with Hoechst to visualize chromosomes. Scale bar, 20 μm. **k** The percentage of oocytes with aberrant spindle in control and *Brca2*-deficient groups. **l** The percentage of oocytes with misaligned chromosome in control and *Brca2*-deficient groups. Statistical data were presented as mean percentage (mean ± SEM) of at least three independent experiments. Asterisk denotes statistical difference at a *p* < 0.05 level of significance

Since we had observed the degenerating oocytes from histological sections of mutant ovaries, we next set out to examine the oocyte development in loss of Brca2. Female mice at 2 months of age were superovulated to collect the in vivo matured eggs. Compared to controls, the number of eggs retrieved from mutant mice was significantly reduced by half (Fig. 4a, b). In addition, more than 30% of superovulated eggs from mutant mice were fragmented (Fig. 4a, c). Due to the deficiency of Brca2, nearly all of the oocytes from mutant mice had obvious DNA damage, stained by DNA damage marker γH2AX (Fig. 4d, e). We next investigated the oocyte development by in vitro maturation. GV oocytes were retrieved from both control and mutant ovaries. In controls, almost all of oocytes are fully grown with a well-surrounded zona pellucida. In mutant mice, however, roughly half of oocytes were immature without a well-formed zona pellucida (Fig. 4f, g), suggesting that the growth of mutant oocytes were arrested at the early stage of development. Most of fully-grown GV oocytes in controls could be in vitro matured to MII stage and extrude the first polar body. By striking contrast, less than 20% of mutant GV oocytes reached MII stage with the first polar body after in vitro maturation (Fig. 4h, i). Immunostaining analysis of arrested mutant oocytes showed that the mutant oocytes which could not extrude the first polar body were arrested at MI stage with morphologically aberrant spindles and misaligned chromosomes (Fig. 4j–l). Taken together, these observations validate that deletion of Brac2 in oocytes leads to the meiotic maturation arrest and developmental failure, consequently contributing to the female infertility.

### Exogenous expression of Brca2 in *Brca2*-deficient oocytes restores the meiotic failure

**Fig. 5:**
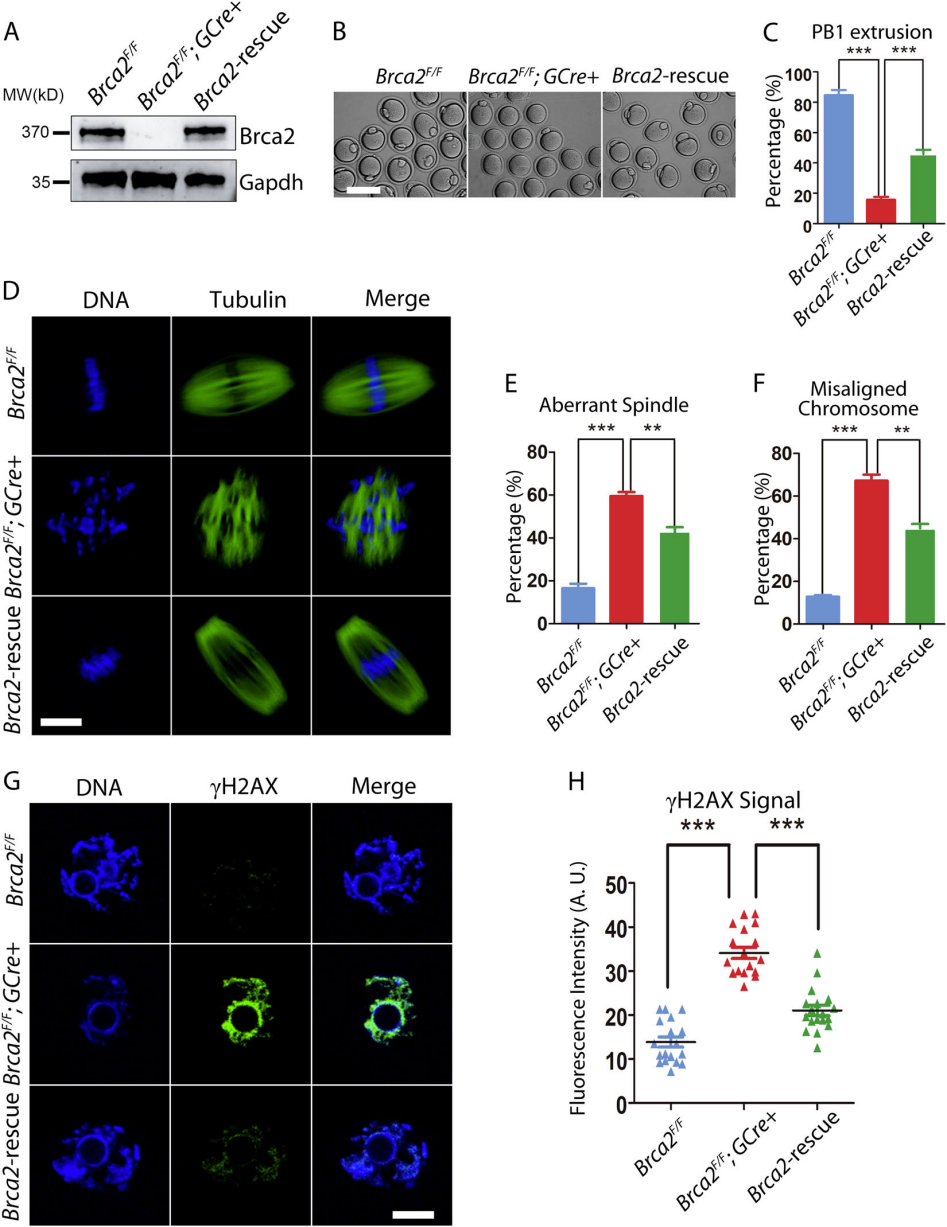
Ectopic expression of Brca2 recovers the quality of *Brca2*-deficient oocytes. **a** Immunoblotting analysis of Brca2-HA protein expression in *Brca2*-deficient oocytes. The blot was probed with anti-Brca2 antibody. The amount of Gapdh was used as an internal control. Two hundered germinal vesicle oocytes isolated from ovaries of 2-month-old mice were used for each lane of the blots. For each experiment, at least five mice of each genotype were used. **b** Representative images of MII oocytes matured in vitro from control (*Brca2*-proficient), *Brca2*-deficient, and Brca2-rescued GV oocytes. Scale bar, 20 μm. **c** The rate of first polar body extrusion of control, *Brca2*-deficient, and Brca2-rescued oocytes matured in vitro. **d** Representative images of spindle morphology and chromosome alignment in control, *Brca2*-deficient, and Brca2-rescued oocytes after in vitro maturation. Oocytes were immunostained with anti-α-tubulin-FITC antibody to visualize spindles and counterstained with Hoechst to visualize chromosomes. Scale bar, 20 μm. (**e**, **f**) The rates of disorganized spindles and misaligned chromosomes in control, *Brca2*-deficient and Brca2-rescued oocytes. **g** Immunofluorescence of γH2AX in control, *Brca2*-deficient and Brca2-rescued oocytes. **h** Fluorescence intensity of γH2AX signal in control, *Brca2*-deficient and Brca2-rescued oocytes were summarized. Statistical data were presented as mean percentage (mean ± SEM) of at least three independent experiments. Asterisk denotes statistical difference at a *p* < 0.05 level of significance

Given that some fully-grown GV oocytes still can be obtained from *Brca2*-deficient mice, we hypothesized that recovery of Brca2 level in mutant oocytes might restore the meiotic progression and oocyte quality. To test it, we expressed exogenous Brca2-HA mRNA in *Brca2*-deficient GV oocytes. Immunoblotting analysis confirmed that Brca2-HA was successfully expressed in mutant oocytes and the expression level was comparable to controls (Fig. 5a). Consistent with our hypothesis, a considerably higher proportion of rescued oocytes were able to reach MII stage with extruded first polar body compared to mutant oocytes (Fig. 5b, c), suggesting that meiotic arrest caused by deletion of Brca2 in oocytes could be restored by elevation of Brca2 protein level. We further examined the spindle morphology and chromosome alignment in rescued MII oocytes, and found that ectopic expression of Brca2 partially improved the mutant oocyte quality (Fig. 5d–f). Accordingly, the exogenous expression of Brca2 significantly reduced the DNA damage in *Brca2*-deficient oocytes (Fig. 5g, h). These data indicate that Brca2 is an essential factor for oocyte development during meiosis.

## Discussion

*BRCA* genes belong to the family of ataxia-telangiectasia mutated (ATM)-mediated DNA DSBs repair signaling pathway that plays a critical role in the safeguarding of DNA integrity^23^. Deleterious mutations in *BRCA1* and *BRCA2* genes may lead to accumulation of DNA damages that predispose female carriers to a high risk of breast and ovarian malignancies, often occurring prior to menopause^24,25^. Cancer treatments or primary prevention strategies have a direct effect on the female fertility and therefore reduce the possibilities to become genetic parents. In addition, several lines of evidence indicate that *BRCA* germline mutations are associated with ovarian reserve and female fertility^24^. The studies in human clinics and transgenic mouse models have demonstrated that compromise of BRCA1-related DNA double-strand break repair would lead to diminished ovarian reserve and accelerated ovarian aging^26^. However, controversial data exist regarding the impact of *BRCA2* mutations on the reproductive function. The investigations by Oktay group reported that there is not a clear relationship between *BRCA2* gene function and age in females younger than 41 years^27^. Also, the number of superovulated oocytes, litter size and primordial follicle density are not affected in *Brca2*^*Δ27/Δ27*^ mice in which only the *RAD51*-interacting region encoded by exon 27 is deleted^27^. By contrast, the study using the *Brca2*-null mice carrying a human BAC with the *BRCA2* gene showed that there is remarkable depletion of germ cells in adult female mutant mice, although some mutant oocytes can progress through meiotic prophase I with a high frequency of abnormalities^28^. The discrepancy might be due to the different mouse models they used for the investigations.

In our current study, we firstly found the downexpression of *BRCA2* gene in oocytes from POI patients, suggesting that *BRCA2* might be involved in the female reproductive events. Further, to clearly define the function of Brca2 during ovarian growth and oocyte development, we generated the conditional knockout mice by crossing *Brca2*^*loxP/loxP*^ mice with transgenic mice expressing *Gdf-9* promoter-mediated Cre recombinase so that *Brca2* is specifically deleted in oocytes of primordial follicles and later stage follicles. Our findings from the conditional knockout mouse model revealed that BRCA2 is required for follicle development, oocyte maturation and female fertility. Our observations are consistent with a recent clinic finding reporting that two sisters with XX ovarian dysgenesis carried compound heterozygous truncating mutations in the *BRCA2* gene that led to reduced BRCA2 protein levels and an impaired response to DNA damage^29^. Moreover, *BRCA2* knockout animal mutant was modeled in the drosophila showing the underdeveloped ovaries and infertility^29^. Subsequently, additional compound heterozygous *BRCA2* mutations were identified in Chinese and Italian patients with premature ovarian insufficiency, which expands the phenotypic spectrum associated with biallelic hypomorphic *BRCA2* variants^30,31^.

Due to the rare resource of human POI oocytes, we cannot enlarge the number of samples in the study. Nevertheless, it is still beyond our expectation that across the 84 DDR genes only *BRCA2* and *XRCC2* showed significant expression difference among these POI oocytes. Mechanically, to repair DSBs BRCA2 interacts with the central recombination protein RAD51, forming nucleoprotein filaments on ssDNA to exert homology search and DNA strand invasion^19,32^. This event lies the downstream of HR pathway, namely effector event, and is the most important process for HR pathway^33,34^. In parallel, two other Rad51-related proteins, XRCC2 and XRCC3 are expressed in human cells. Both of them interact with Rad51 and facilitate HR repair^35,36^. Thus, it is possible that dysfunction of effector genes (downstream genes) in HR pathway could significantly cause a failure of DSBs repair and potentially induce POI.

In *Brca2*-deficent mouse ovaries, the number of primordial follicle pool was not lost, but the activated transient follicles were developmentally arrested with degraded oocytes, which means that the early events during ovarian development such as oogenesis or follicle recruitment are not easily affected by the accumulative DNA damage. In mammals, oocytes development is a long process that lasts for several months or years in follicular microenvironment depending on species^37,38^. This requires the oocytes to have high tolerance to various threats including types of DNA damages in their microenvironment. Hence, oocytes tend to survive and develop until they encounter serious problems such as unrepaired DSBs, the most deleterious DNA damage for the genetic material. In terms of evolution, mammalian females tend to ensure their generation rather than sacrifice their flawed gametes. This view could be also supported by previous findings that up to 95% of chromosomal disorders and errors originate from oocytes but not sperms^39^. As such, core DSBs repair factors may be more important for oocyte development. Fortunately in our study, some fully-grown GV oocytes could still be obtained from mutant ovaries although a lot of them had meiotic defects during both in vivo and in vitro maturation. We then hypothesized and tested if elevation of the protein level of Brca2 in *Brca2*-deficent oocytes in vitro can ameliorate oocyte quality. The data confirmed our hypothesis that the quality of partial mutant oocytes was indeed recovered. Thus, we assume that several core DSBs repair factors such as BRCA2, RAD51, XRCC2, and XRCC3 could be potentially diagnostic biomarkers for POI, and offer an opportunity to change the fertility outcome of POI patients caused by *BRCA2* deficiency.

## Materials and methods

### Human POI oocyte collection

This study is approved by the Institutional Medical and Ethical Review Committee of The Third Affiliated Hospital of Guangzhou Medical University. Informed consent was obtained from all participants. Human POI oocytes were collected from The Third Affiliated Hospital of Guangzhou Medical University. Oocytes from POI patients with failed IVF that were planned to be discarded were harvested. The inclusion criteria of POI patients were according to previous reports^40,41^. In total, 32 POI oocytes from 30 patients after failed IVF were obtained. Among them three oocytes went to death during in vitro maturation, and one oocyte failed in the test of single cell library assessment. Another two oocytes showed unqualified CT values of quality control genes on the array and were also excluded. Thus, 26 POI oocytes were finally included in the study. Five normal oocytes obtained from healthy donors were used as the control.

### Transcriptional expression assay of DDR factors in human POI oocytes

Preparation of single oocyte cDNAs was performed following previously reported modified Smart-seq2 protocol^42,43^. Briefly, a single oocyte was transferred to 4 μl cell lysis buffer using a mouth pipet under a microscope. Each sample was vortexed for 45 s and incubated at 72 °C for 3 min to release the RNAs. Then, the first-strand cDNA was reverse-synthesized using oligo(dT) and template-switching oligonucleotides (TSO). Reverse transcription reaction was carried out by incubating at 25 °C for 5 min, 42 °C for 60 min, 50 °C for 30 min, and 70 °C for 10 min. The PCR preamplification was performed using IS PCR primer and 3′ P2 primer. The reaction was incubated at 95 °C for 3 min, cycled 4 times between (98 °C 20 s, 65 °C 30 s, 72 °C 5 min), and cycled 15 times between (98 °C 20 s, 67 °C 15 s, 72 °C 5 min) followed by a final extension at 72 °C for 5 min. Then, the cDNA was diluted to 100 μl. oligo(dT), TCAGACGTGTGCTCTTCCGATCTAACGTGATDDDDDDDDT_25_; TSO, AAGCAGTGGTATCAACGCAGAGTACATrGrG+G; IS PCR primer, AAGCAGTGGTATCAACGCAGAGT; 3′ P2 primer, GTGACTGGAGTTCAGACGTGTGCTCTTCCGATC. For transcriptional expression assay, the cDNA of each oocyte was loaded onto the RT^2^ Profiler PCR Array (Qiagen, PAHS-042ZA).

### Mice

Mice lacking *Brca2* in oocytes (referred to as *Brca2*^*F/F*^;*Gdf9-Cre*) were generated by crossing *Brca2*^*F/F*^ mice with *Gdf9-Cre* mice. *Brca2*^*F/F*^ mice were produced by our lab with LoxP sites inserted into introns surrounding exon 11 in the *Brca2* locus. *Gdf9-Cre* mice were kindly provided by Dr. Hengyu Fan at Zhejiang University. Both transgenic mouse lines have C57BL/6J genomic background. The mice were housed under controlled environmental conditions with free access to water and food. Light was provided between 08:00 and 20:00. Animal care and handling were conducted according to the guidelines of the Animal Research Committee of Nanjing Agricultural University, China.

### Antibodies

Rabbit polyclonal anti-Brca2 antibody was purchased from Santa Cruz Biotechnology (Dallas, TA, USA; Cat#: sc-293185); mouse monoclonal anti-α-tubulin-fluorescein isothiocyanate (FITC) antibody was purchased from Sigma (St. Louis, MO, USA; Cat#: F2168); Rabbit monoclonal anti-γH2AX and anti-Gapdh antibodies were purchased from Cell Signaling Technology (Danvers, MA, USA; Cat#: 9718, 2118); HRP-conjugated secondary antibodies were purchased from Jackson ImmunoResearch Laboratories (West Grove, PA, USA); FITC-conjugated goat anti-rabbit IgG (H + L), TRITC-conjugated goat anti-rabbit IgG (H + L) and FITC-conjugated goat anti-mouse IgG (H + L) were purchased from Zhongshan Golden Bridge Biotechnology Co., LTD (Beijing, China).

### Breeding assay

In breeding assays, *Brca2*^*F/F*^ and *Brca2*^*F/F*^;*Gdf9-Cre* female mice with sexual maturity were continually mated to *Brca2*^*F/F*^ male mice with known fertility for 6 months. Cages were checked daily for counting the number of litters and pups.

### Histological analysis and immunostaining of ovaries

Ovaries used for histological analysis were collected from young adult female mice (2-month-old) and fixed in 4% paraformaldehyde (pH 7.5) overnight at 4 °C, dehydrated, and embedded in paraffin. Paraffin-embedded ovaries were sectioned at a thickness of 8 μm for hematoxylin and eosin (H&E) staining. One or both ovaries from more than three mice of each genotype were used for the analysis.

Paraffin-embedded ovarian tissue sections were deparaffinized, immersed in retrieval solution (10 mM sodium citrate), heated in an autoclave, blocked with 10% normal goat serum, and then incubated overnight with the indicated primary antibodies. For immunofluorescence, localization of the primary antibody was performed by incubation of the sections with the corresponding secondary antibodies (Invitrogen) at 1:500 dilution for 1 h at room temperature. Finally, nuclei were stained with DAPI. At least three different samples from each genotype were analyzed in parallel.

### Quantification of ovarian follicles

To count the numbers of follicles, paraffin-embedded ovaries were serially sectioned at 8 μm thickness and every fifth section was mounted on slides. Then these sections were stained with hematoxylin and eosin for morphological analysis. Ovarian follicles at different developmental stages, including primordial and activated follicles (transient follicles containing enlarged oocytes surrounded by flattened pregranulosa cells, primary (type 3 and type 4), secondary (type 5), and antral follicles (type 6 and type 7)) were counted in all sections of an ovary, based on the well-accepted standards established by Peterson and Peters^44^. In each section, only those follicles in which the nucleus of the oocyte was clearly visible were scored and the cumulative follicle counts were multiplied by a correction factor of five to represent the estimated number of total follicles in an ovary. Judged from careful morphological analysis, the incidence of counting the same follicle twice or of missing a follicle was low.

### Mouse oocyte collection and culture

Female mice (2-month-old) were sacrificed by cervical dislocation. Fully-grown oocytes arrested at prophase of meiosis I were collected from ovaries in M2 medium (Sigma, St. Louis, MO, USA). Only those immature oocytes displaying a GV were cultured further in M16 medium (Sigma, St. Louis, MO, USA) under liquid paraffin oil at 37 °C in an atmosphere of 5% CO2 incubator for in vitro maturation. At different time points after culture, oocytes were collected for subsequent analysis. Oocytes at the second metaphase of meiosis (MII) were collected from oviductal ampullae 13–14 h after female mice were superovulated by an intraperitoneal injection of 5 IU pregnant mare serum gonadotropin followed by an injection of 5 IU human chorionic gonadotropin (both from Tianjin Animal Hormone Factory, Tianjin, China) 46–48 h later (100 μl/mouse/injection).

### cRNA construct and in vitro transcription

Wild-type mouse Brca2 cDNA was sub-cloned into pcDNA3.1/HA vector. Capped cRNA was synthesized from linearized plasmid using T7 mMessage mMachine kit (ThermoFisher), and purified with MEGAclear kit (ThermoFisher). Typically, 10–12 pl of 0.5–1.0 μg/μl cRNA was injected into oocytes and then arrested at the GV stage in M16 medium containing 2.5 µM milrinone for 6 h, allowing enough time for translation, followed by releasing into milrinone-free M16 medium for further study.

### Immunofluorescence and confocal microscopy

Oocytes were fixed in 4% paraformaldehyde in PBS (pH 7.4) for 30 min and permeabilized in 0.5% Triton-X-100 for 20 min at room temperature. Then, oocytes were blocked with 1% BSA-supplemented PBS for 1 h and incubated with the indicated primary antibody at 4 °C overnight. After washing four times (5 min each) in PBS containing 1% Tween 20 and 0.01% Triton-X 100, oocytes were incubated with an appropriate secondary antibody for 1 h at room temperature. After washing three times, oocytes were counterstained with PI (Propidium Iodide) or Hoechst 33342 (10 µg/ml) for 10 min. Finally, oocytes were mounted on glass slides and observed under a confocal laser scanning microscope (Carl Zeiss 700).

### Immunoblotting analysis

For immunoblotting, a pool of 200 oocytes was lysed in 4× LDS sample buffer (ThermoFisher, Waltham, MA, USA) containing protease inhibitor, and then separated on 10% Bis-Tris precast gels and transferred onto PVDF membranes. The blots were blocked in TBST (Tris-buffred saline containing 0.1% Tween 20) containing 5% low fat dry milk for 1 h at room temperature and then incubated with anti-Brca2 antibody (1:1000) overnight at 4 °C. After three times of washes in TBST, the blots were incubated with 1:10,000 dilution of HRP (Horse Radish Peroxidase) conjugated secondary antibodies for 1 h at room temperature. Chemiluminescence was detected with ECL Plus Western Blotting Detection System (GE, Piscataway, NJ, USA) and protein bands were visualized by Tanon-3900. The blots were then stripped and reblotted with anti-Gapdh antibody (1:5000) for loading control.

### Statistical analysis

All percentages from at least three repeated experiments were expressed as mean ± SEM, and the number of oocytes observed was labeled in parenthesesas (n). Data were analyzed by *t-*test, which was provided by SPSS16.0 statistical software. The level of significance was accepted as *p* < 0.05.

## Supplementary information

Supplementary Tables

## References

[1] Nelson LM (2009). Clinical practice. Primary ovarian insufficiency. N. Engl. J. Med..

[2] Goswami D, Conway GS (2005). Premature ovarian failure. Hum. Reprod. Update..

[3] Rafique S, Sterling EW, Nelson LM (2012). A new approach to primary ovarian insufficiency. Obstet. Gynecol. Clin. N. Am..

[4] Perry JR (2013). A genome-wide association study of early menopause and the combined impact of identified variants. Hum. Mol. Genet..

[5] Qin Y, Jiao X, Simpson JL, Chen ZJ (2015). Genetics of primary ovarian insufficiency: new developments and opportunities. Hum. Reprod. Update.

[6] De Vos M, Devroey P, Fauser BC (2010). Primary ovarian insufficiency. Lancet..

[7] Jackson SP, Bartek J (2009). The DNA-damage response in human biology and disease. Nature.

[8] Ciccia A, Elledge SJ (2010). The DNA damage response: making it safe to play with knives. Mol. Cell.

[9] Zhou BB, Elledge SJ (2000). The DNA damage response: putting checkpoints in perspective. Nature.

[10] Harper JW, Elledge SJ (2007). The DNA damage response: ten years after. Mol. Cell.

[11] van Gent DC, Hoeijmakers JH, Kanaar R (2001). Chromosomal stability and the DNA double-stranded break connection. Nat. Rev. Genet..

[12] Mostoslavsky R (2006). Genomic instability and aging-like phenotype in the absence of mammalian SIRT6. Cell.

[13] Halazonetis TD, Gorgoulis VG, Bartek J (2008). An oncogene-induced DNA damage model for cancer development. Science.

[14] Suh EK (2006). p63 protects the female germ line during meiotic arrest. Nature.

[15] Bolcun-Filas E, Rinaldi VD, White ME, Schimenti JC (2014). Reversal of female infertility by Chk2 ablation reveals the oocyte DNA damage checkpoint pathway. Science.

[16] Li W, Hu Q, Wan C (2016). Uptake and accumulation of nephrotoxic and carcinogenic aristolochic acids in food crops grown in aristolochia clematitis-contaminated soil and water. J. Agric. Food Chem..

[17] Tang F (2010). RNA-Seq analysis to capture the transcriptome landscape of a single cell. Nat. Protoc..

[18] Tang F (2009). mRNA-Seq whole-transcriptome analysis of a single cell. Nat. Methods.

[19] Jensen RB, Carreira A, Kowalczykowski SC (2010). Purified human BRCA2 stimulates RAD51-mediated recombination. Nature.

[20] Wooster R (1995). Identification of the breast cancer susceptibility gene BRCA2. Nature.

[21] Hu MW, Wang ZB, Schatten H, Sun QY (2012). New understandings on folliculogenesis/oogenesis regulation in mouse as revealed by conditional knockout. J. Genet. Genom..

[22] Sun QY, Liu K, Kikuchi K (2008). Oocyte-specific knockout: a novel in vivo approach for studying gene functions during folliculogenesis, oocyte maturation, fertilization, and embryogenesis. Biol. Reprod..

[23] Venkitaraman AR (2002). Cancer susceptibility and the functions of BRCA1 and BRCA2. Cell.

[24] Smith KR, Hanson HA, Hollingshaus MS (2013). BRCA1 and BRCA2 mutations and female fertility. Curr. Opin. Obstet. Gynecol..

[25] Scully R, Livingston DM (2000). In search of the tumour-suppressor functions of BRCA1 and BRCA2. Nature.

[26] Kutluk O, Volkan T, Shiny T, Robert S, Lin L (2015). BRCA mutations, DNA repair deficiency, and ovarian aging. Biol. Reprod..

[27] Shiny T (2013). Impairment of BRCA1-related DNA double-strand break repair leads to ovarian aging in mice and humans. Sci. Transl. Med..

[28] Sharan SK (2004). BRCA2 deficiency in mice leads to meiotic impairment and infertility. Development.

[29] Weinberg-Shukron A (2018). Essential role of BRCA2 in ovarian development and function. N. Engl. J. Med..

[30] Qin Y, Zhang F, Chen ZJ (2019). BRCA2 in ovarian development and function. N. Engl. J. Med..

[31] Turchetti D, Zuntini R, Tricarico R (2019). BRCA2 in ovarian development and function. N. Engl. J. Med..

[32] Liu J, Doty T, Gibson B, Heyer WD (2010). Human BRCA2 protein promotes RAD51 filament formation on RPA-covered single-stranded DNA. Nat. Struct. Mol. Biol..

[33] Sancar A, Lindsey-Boltz LA, Unsal-Kacmaz K, Linn S (2004). Molecular mechanisms of mammalian DNA repair and the DNA damage checkpoints. Annu. Rev. Biochem..

[34] Roy R, Chun J, Powell SN (2011). BRCA1 and BRCA2: different roles in a common pathway of genome protection. Nat. Rev. Cancer.

[35] Liu N (1998). XRCC2 and XRCC3, new human Rad51-family members, promote chromosome stability and protect against DNA cross-links and other damages. Mol. Cell.

[36] Karran P (2000). DNA double strand break repair in mammalian cells. Curr. Opin. Genet. Dev..

[37] Mehlmann LM (2005). Stops and starts in mammalian oocytes: recent advances in understanding the regulation of meiotic arrest and oocyte maturation. Reproduction.

[38] Tripathi A, Kumar KV, Chaube SK (2010). Meiotic cell cycle arrest in mammalian oocytes. J. Cell Physiol..

[39] Kuliev A, Zlatopolsky Z, Kirillova I, Spivakova J, Cieslak Janzen J (2011). Meiosis errors in over 20,000 oocytes studied in the practice of preimplantation aneuploidy testing. Reprod. Biomed. Online.

[40] Huang K (2018). CAV1 regulates primordial follicle formation via the Notch2 signalling pathway and is associated with premature ovarian insufficiency in humans. Hum. Reprod..

[41] Bidet M (2011). Resumption of ovarian function and pregnancies in 358 patients with premature ovarian failure. J. Clin. Endocrinol. Metab..

[42] Picelli S (2013). Smart-seq2 for sensitive full-length transcriptome profiling in single cells. Nat. Methods.

[43] Picelli S (2014). Full-length RNA-seq from single cells using Smart-seq2. Nat. Protoc..

[44] Pedersen T, Peters H (1968). Proposal for a classification of oocytes and follicles in the mouse ovary. J. Reprod. Fertil..

