# Supplementary material for: BRCA2 deficiency is a potential driver for human primary ovarian insufficiency
Source: Cell Death Dis. 2019 Jun 17;10(7):474. doi: 10.1038/s41419-019-1720-0 (PMC6572856; doi:10.1038/s41419-019-1720-0)
Supplement: Supplementary file 1 — Supplementary Tables [file 41419_2019_1720_MOESM1_ESM.docx]

| **Oocyte Information** | | | | | | | | | |
| --- | --- | --- | --- | --- | --- | --- | --- | --- | --- |
| **Oocyte** | **Patient** | **Patient Age (year)** | **Patient BMI (kg/m2)** | **Patient FSH (IU/l)** | **Patient Other Disease** | **In vitro Culture** | **GAPDH Control before the Array** | **Quality Control on the Array** | **Final** |
| POI oocyte 1 | Patient 1 | 29 | 19.56 | 64.27 | none | alive | CT<22 | passed | included |
| POI oocyte 2 | Patient 2 | 28 | 23.74 | 82.46 | none | dead | ND | ND | excluded |
| POI oocyte 3 | Patient 3 | 31 | 21.62 | 49.58 | none | alive | CT<22 | passed | included |
| POI oocyte 4 | Patient 4 | 23 | 19.52 | 85.36 | none | alive | CT<22 | passed | included |
| POI oocyte 5 | Patient 5 | 35 | 20.27 | 74.62 | none | alive | CT<22 | passed | included |
| POI oocyte 6 | Patient 6 | 32 | 18.65 | 68.47 | none | alive | CT<22 | passed | included |
| POI oocyte 7 | Patient 7 | 26 | 22.59 | 114.81 | none | alive | CT<22 | passed | included |
| POI oocyte 8 | Patient 8 | 34 | 17.94 | 83.59 | none | alive | CT<22 | passed | included |
| POI oocyte 9 | Patient 9 | 26 | 24.32 | 87.64 | none | alive | CT<22 | passed | included |
| POI oocyte 10 | Patient 10 | 28 | 19.75 | 62.81 | none | alive | CT<22 | failed | excluded |
| POI oocyte 11 | Patient 11 | 31 | 19.28 | 83.25 | none | alive | CT<22 | passed | included |
| POI oocyte 12 | Patient 12 | 25 | 23.62 | 68.74 | none | alive | CT<22 | passed | included |
| POI oocyte 13 | Patient 13 | 28 | 24.43 | 106.35 | none | alive | CT<22 | failed | excluded |
| POI oocyte 14 | Patient 14 | 25 | 18.26 | 68.46 | none | alive | CT<22 | passed | included |
| POI oocyte 15 | Patient 15 | 32 | 23.83 | 82.23 | none | alive | CT<22 | passed | included |
| POI oocyte 16 | Patient 16 | 28 | 24.41 | 75.49 | none | alive | CT<22 | passed | included |
| POI oocyte 17 | Patient 17 | 23 | 19.83 | 56.24 | none | alive | CT<22 | passed | included |
| POI oocyte 18 |  |  |  |  |  | alive | CT>24 | ND | excluded |
| POI oocyte 19 | Patient 18 | 27 | 22.67 | 84.52 | none | alive | CT<22 | passed | included |
| POI oocyte 20 | Patient 19 | 38 | 24.72 | 72.68 | none | alive | CT<22 | passed | included |
| POI oocyte 21 | Patient 20 | 35 | 19.38 | 66.94 | none | alive | CT<22 | passed | included |
| POI oocyte 22 | Patient 21 | 26 | 18.26 | 126.35 | none | alive | CT<22 | passed | included |
| POI oocyte 23 | Patient 22 | 24 | 21.67 | 83.71 | none | dead | ND | ND | excluded |
| POI oocyte 24 | Patient 23 | 27 | 26.45 | 74.53 | none | alive | CT<22 | passed | included |
| POI oocyte 25 | Patient 24 | 25 | 23.72 | 116.58 | none | alive | CT<22 | passed | included |
| POI oocyte 26 | Patient 25 | 28 | 19.48 | 71.24 | none | alive | CT<22 | passed | included |
| POI oocyte 27 | Patient 26 | 35 | 22.14 | 78.69 | none | dead | ND | ND | excluded |
| POI oocyte 28 | Patient 27 | 29 | 18.62 | 62.28 | none | alive | CT<22 | passed | included |
| POI oocyte 29 | Patient 28 | 32 | 19.46 | 83.17 | none | alive | CT<22 | passed | included |
| POI oocyte 30 |  |  |  |  |  | alive | CT<22 | passed | included |
| POI oocyte 31 | Patient 29 | 28 | 23.25 | 64.52 | none | alive | CT<22 | passed | included |
| POI oocyte 32 | Patient 30 | 24 | 20.68 | 84.39 | none | alive | CT<22 | passed | included |
| Control oocyte 1 | Donor 1 | 31 | 21.82 | 4.37 | none | alive | CT<22 | passed | included |
| Control oocyte 2 | Donor 2 | 25 | 23.66 | 8.16 | none | alive | CT<22 | passed | included |
| Control oocyte 3 | Donor 3 | 28 | 18.62 | 7.38 | none | alive | CT<22 | passed | included |
| Control oocyte 4 | Donor 4 | 30 | 21.47 | 11.24 | none | alive | CT<22 | passed | included |
| Control oocyte 5 | Donor 5 | 26 | 22.54 | 4.07 | none | alive | CT<22 | passed | included |

**Supplementary Tables**

**Table S1: Oocyte information**

**Table S2: Genes information on the array**

| **Position** | **UniGene** | **GenBank** | **Symbol** | **Description** |
| --- | --- | --- | --- | --- |
| A01 | Hs.73722 | NM_080649 | APEX1 | APEX nuclease (multifunctional DNA repair enzyme) 1 |
| A02 | Hs.659558 | NM_014481 | APEX2 | APEX nuclease (apurinic/apyrimidinic endonuclease) 2 |
| A03 | Hs.367437 | NM_000051 | ATM | Ataxia telangiectasia mutated |
| A04 | Hs.271791 | NM_001184 | ATR | Ataxia telangiectasia and Rad3 related |
| A05 | Hs.532632 | NM_004993 | ATXN3 | Ataxin 3 |
| A06 | Hs.194143 | NM_007294 | BRCA1 | Breast cancer 1, early onset |
| A07 | Hs.34012 | NM_000059 | BRCA2 | Breast cancer 2, early onset |
| A08 | Hs.532799 | NM_032043 | BRIP1 | BRCA1 interacting protein C-terminal helicase 1 |
| A09 | Hs.292524 | NM_001239 | CCNH | Cyclin H |
| A10 | Hs.3041 | NM_021147 | CCNO | Cyclin O |
| A11 | Hs.184298 | NM_001799 | CDK7 | Cyclin-dependent kinase 7 |
| A12 | Hs.290758 | NM_001923 | DDB1 | Damage-specific DNA binding protein 1, 127kDa |
| B01 | Hs.700338 | NM_000107 | DDB2 | Damage-specific DNA binding protein 2, 48kDa |
| B02 | Hs.339396 | NM_007068 | DMC1 | DMC1 dosage suppressor of mck1 homolog, meiosis-specific homologous recombination (yeast) |
| B03 | Hs.435981 | NM_001983 | ERCC1 | Excision repair cross-complementing rodent repair deficiency, complementation group 1 (includes overlapping antisense sequence) |
| B04 | Hs.487294 | NM_000400 | ERCC2 | Excision repair cross-complementing rodent repair deficiency, complementation group 2 |
| B05 | Hs.469872 | NM_000122 | ERCC3 | Excision repair cross-complementing rodent repair deficiency, complementation group 3 (xeroderma pigmentosum group B complementing) |
| B06 | Hs.567265 | NM_005236 | ERCC4 | Excision repair cross-complementing rodent repair deficiency, complementation group 4 |
| B07 | Hs.258429 | NM_000123 | ERCC5 | Excision repair cross-complementing rodent repair deficiency, complementation group 5 |
| B08 | Hs.654449 | NM_000124 | ERCC6 | Excision repair cross-complementing rodent repair deficiency, complementation group 6 |
| B09 | Hs.435237 | NM_000082 | ERCC8 | Excision repair cross-complementing rodent repair deficiency, complementation group 8 |
| B10 | Hs.498248 | NM_130398 | EXO1 | Exonuclease 1 |
| B11 | Hs.409065 | NM_004111 | FEN1 | Flap structure-specific endonuclease 1 |
| B12 | Hs.1770 | NM_000234 | LIG1 | Ligase I, DNA, ATP-dependent |
| C01 | Hs.100299 | NM_002311 | LIG3 | Ligase III, DNA, ATP-dependent |
| C02 | Hs.166091 | NM_002312 | LIG4 | Ligase IV, DNA, ATP-dependent |
| C03 | Hs.501522 | NM_002412 | MGMT | O-6-methylguanine-DNA methyltransferase |
| C04 | Hs.195364 | NM_000249 | MLH1 | MutL homolog 1, colon cancer, nonpolyposis type 2 (E. coli) |
| C05 | Hs.436650 | NM_014381 | MLH3 | MutL homolog 3 (E. coli) |
| C06 | Hs.500721 | NM_022362 | MMS19 | MMS19 nucleotide excision repair homolog (S. cerevisiae) |
| C07 | Hs.459596 | NM_002434 | MPG | N-methylpurine-DNA glycosylase |
| C08 | Hs.192649 | NM_005590 | MRE11A | MRE11 meiotic recombination 11 homolog A (S. cerevisiae) |
| C09 | Hs.597656 | NM_000251 | MSH2 | MutS homolog 2, colon cancer, nonpolyposis type 1 (E. coli) |
| C10 | Hs.280987 | NM_002439 | MSH3 | MutS homolog 3 (E. coli) |
| C11 | Hs.216639 | NM_002440 | MSH4 | MutS homolog 4 (E. coli) |
| C12 | Hs.647011 | NM_002441 | MSH5 | MutS homolog 5 (E. coli) |
| D01 | Hs.445052 | NM_000179 | MSH6 | MutS homolog 6 (E. coli) |
| D02 | Hs.271353 | NM_012222 | MUTYH | MutY homolog (E. coli) |
| D03 | Hs.512732 | NM_024608 | NEIL1 | Nei endonuclease VIII-like 1 (E. coli) |
| D04 | Hs.293818 | NM_145043 | NEIL2 | Nei endonuclease VIII-like 2 (E. coli) |
| D05 | Hs.405467 | NM_018248 | NEIL3 | Nei endonuclease VIII-like 3 (E. coli) |
| D06 | Hs.66196 | NM_002528 | NTHL1 | Nth endonuclease III-like 1 (E. coli) |
| D07 | Hs.380271 | NM_002542 | OGG1 | 8-oxoguanine DNA glycosylase |
| D08 | Hs.177766 | NM_001618 | PARP1 | Poly (ADP-ribose) polymerase 1 |
| D09 | Hs.409412 | NM_005484 | PARP2 | Poly (ADP-ribose) polymerase 2 |
| D10 | Hs.271742 | NM_005485 | PARP3 | Poly (ADP-ribose) polymerase family, member 3 |
| D11 | Hs.111749 | NM_000534 | PMS1 | PMS1 postmeiotic segregation increased 1 (S. cerevisiae) |
| D12 | Hs.632637 | NM_000535 | PMS2 | PMS2 postmeiotic segregation increased 2 (S. cerevisiae) |
| E01 | Hs.78016 | NM_007254 | PNKP | Polynucleotide kinase 3'-phosphatase |
| E02 | Hs.654484 | NM_002690 | POLB | Polymerase (DNA directed), beta |
| E03 | Hs.82502 | NM_006591 | POLD3 | Polymerase (DNA-directed), delta 3, accessory subunit |
| E04 | Hs.523230 | NM_013274 | POLL | Polymerase (DNA directed), lambda |
| E05 | Hs.491682 | NM_006904 | PRKDC | Protein kinase, DNA-activated, catalytic polypeptide |
| E06 | Hs.375684 | NM_020165 | RAD18 | RAD18 homolog (S. cerevisiae) |
| E07 | Hs.81848 | NM_006265 | RAD21 | RAD21 homolog (S. pombe) |
| E08 | Hs.643267 | NM_005053 | RAD23A | RAD23 homolog A (S. cerevisiae) |
| E09 | Hs.521640 | NM_002874 | RAD23B | RAD23 homolog B (S. cerevisiae) |
| E10 | Hs.655835 | NM_005732 | RAD50 | RAD50 homolog (S. cerevisiae) |
| E11 | Hs.631709 | NM_002875 | RAD51 | RAD51 homolog (S. cerevisiae) |
| E12 | Hs.172587 | NM_133509 | RAD51B | RAD51 homolog B (S. cerevisiae) |
| F01 | Hs.412587 | NM_058216 | RAD51C | RAD51 homolog C (S. cerevisiae) |
| F02 | Hs.631757 | NM_002878 | RAD51D | RAD51 homolog D (S. cerevisiae) |
| F03 | Hs.709202 | NM_134424 | RAD52 | RAD52 homolog (S. cerevisiae) |
| F04 | Hs.642042 | NM_003579 | RAD54L | RAD54-like (S. cerevisiae) |
| F05 | Hs.507475 | NM_002913 | RFC1 | Replication factor C (activator 1) 1, 145kDa |
| F06 | Hs.461925 | NM_002945 | RPA1 | Replication protein A1, 70kDa |
| F07 | Hs.487540 | NM_002947 | RPA3 | Replication protein A3, 14kDa |
| F08 | Hs.591922 | NM_014720 | SLK | STE20-like kinase |
| F09 | Hs.632721 | NM_014311 | SMUG1 | Single-strand-selective monofunctional uracil-DNA glycosylase 1 |
| F10 | Hs.584809 | NM_003211 | TDG | Thymine-DNA glycosylase |
| F11 | Hs.592115 | NM_004618 | TOP3A | Topoisomerase (DNA) III alpha |
| F12 | Hs.436401 | NM_003935 | TOP3B | Topoisomerase (DNA) III beta |
| G01 | Hs.707026 | NM_016381 | TREX1 | Three prime repair exonuclease 1 |
| G02 | Hs.191334 | NM_003362 | UNG | Uracil-DNA glycosylase |
| G03 | Hs.9822 | NM_020196 | XAB2 | XPA binding protein 2 |
| G04 | Hs.654364 | NM_000380 | XPA | Xeroderma pigmentosum, complementation group A |
| G05 | Hs.475538 | NM_004628 | XPC | Xeroderma pigmentosum, complementation group C |
| G06 | Hs.98493 | NM_006297 | XRCC1 | X-ray repair complementing defective repair in Chinese hamster cells 1 |
| G07 | Hs.647093 | NM_005431 | XRCC2 | X-ray repair complementing defective repair in Chinese hamster cells 2 |
| G08 | Hs.592325 | NM_005432 | XRCC3 | X-ray repair complementing defective repair in Chinese hamster cells 3 |
| G09 | Hs.567359 | NM_003401 | XRCC4 | X-ray repair complementing defective repair in Chinese hamster cells 4 |
| G10 | Hs.388739 | NM_021141 | XRCC5 | X-ray repair complementing defective repair in Chinese hamster cells 5 (double-strand-break rejoining) |
| G11 | Hs.292493 | NM_001469 | XRCC6 | X-ray repair complementing defective repair in Chinese hamster cells 6 |
| G12 | Hs.61188 | NM_033276 | XRCC6BP1 | XRCC6 binding protein 1 |
| H01 | Hs.520640 | NM_001101 | ACTB | Actin, beta |
| H02 | Hs.534255 | NM_004048 | B2M | Beta-2-microglobulin |
| H03 | Hs.592355 | NM_002046 | GAPDH | Glyceraldehyde-3-phosphate dehydrogenase |
| H04 | Hs.412707 | NM_000194 | HPRT1 | Hypoxanthine phosphoribosyltransferase 1 |
| H05 | Hs.546285 | NM_001002 | RPLP0 | Ribosomal protein, large, P0 |

**Table S3: mRNA expression of the genes on the array**

| **Control 1** |  |  |  |  | |  | |  | |  |  |  |  |  |  |
| --- | --- | --- | --- | --- | --- | --- | --- | --- | --- | --- | --- | --- | --- | --- | --- |
| Well Position | Gene Symbol | Reporter | Quencher | CT | | Delta Ct | | Delta Delta Ct | | 2^(-Delta Delta Ct) | Automatic Ct Threshold | Ct Threshold | Automatic Baseline | Amp Status | MTP |
| A1 | APEX1 | SYBR | None | 18.308 | | -0.049 | | 0 | | 1 | FALSE | 0.5 | TRUE | Amp | N |
| A2 | APEX2 | SYBR | None | 22.092 | | 3.735 | | 0 | | 1 | FALSE | 0.5 | TRUE | Amp | N |
| A3 | ATM | SYBR | None | 23.501 | | 5.144 | | 0 | | 1 | FALSE | 0.5 | TRUE | Amp | N |
| A4 | ATR | SYBR | None | 22.863 | | 4.506 | | 0 | | 1 | FALSE | 0.5 | TRUE | Amp | N |
| A5 | ATXN3 | SYBR | None | 20.577 | | 2.22 | | 0 | | 1 | FALSE | 0.5 | TRUE | Amp | N |
| A6 | BRCA1 | SYBR | None | 21.605 | | 3.248 | | 0 | | 1 | FALSE | 0.5 | TRUE | Amp | N |
| A7 | BRCA2 | SYBR | None | 18.801 | | 0.444 | | 0 | | 1 | FALSE | 0.5 | TRUE | Amp | N |
| A8 | BRIP1 | SYBR | None | 23.091 | | 4.734 | | 0 | | 1 | FALSE | 0.5 | TRUE | Amp | N |
| A9 | CCNH | SYBR | None | 24.906 | | 6.549 | | 0 | | 1 | FALSE | 0.5 | TRUE | Amp | N |
| A10 | CCNO | SYBR | None | 22.596 | | 4.239 | | 0 | | 1 | FALSE | 0.5 | TRUE | Amp | N |
| A11 | CDK7 | SYBR | None | 14.206 | | -4.151 | | 0 | | 1 | FALSE | 0.5 | TRUE | Amp | N |
| A12 | DDB1 | SYBR | None | 20.714 | | 2.357 | | 0 | | 1 | FALSE | 0.5 | TRUE | Amp | N |
| B1 | DDB2 | SYBR | None | 23.252 | | 4.895 | | 0 | | 1 | FALSE | 0.5 | TRUE | Amp | N |
| B2 | DMC1 | SYBR | None | 27.43 | | 9.073 | | 0 | | 1 | FALSE | 0.5 | TRUE | Amp | N |
| B3 | ERCC1 | SYBR | None | 22.201 | | 3.844 | | 0 | | 1 | FALSE | 0.5 | TRUE | Amp | N |
| B4 | ERCC2 | SYBR | None | 24.075 | | 5.718 | | 0 | | 1 | FALSE | 0.5 | TRUE | Amp | N |
| B5 | ERCC3 | SYBR | None | 21.567 | | 3.21 | | 0 | | 1 | FALSE | 0.5 | TRUE | Amp | N |
| B6 | ERCC4 | SYBR | None | 27.721 | | 9.364 | | 0 | | 1 | FALSE | 0.5 | TRUE | Amp | N |
| B7 | ERCC5 | SYBR | None | 25.048 | | 6.691 | | 0 | | 1 | FALSE | 0.5 | TRUE | Amp | N |
| B8 | ERCC6 | SYBR | None | 17.855 | | -0.502 | | 0 | | 1 | FALSE | 0.5 | TRUE | Amp | N |
| B9 | ERCC8 | SYBR | None | 22.734 | | 4.377 | | 0 | | 1 | FALSE | 0.5 | TRUE | Amp | N |
| B10 | EXO1 | SYBR | None | 20.283 | | 1.926 | | 0 | | 1 | FALSE | 0.5 | TRUE | Amp | N |
| B11 | FEN1 | SYBR | None | 17.677 | | -0.68 | | 0 | | 1 | FALSE | 0.5 | TRUE | Amp | N |
| B12 | LIG1 | SYBR | None | 22.073 | | 3.716 | | 0 | | 1 | FALSE | 0.5 | TRUE | Amp | N |
| C1 | LIG3 | SYBR | None | 25.47 | | 7.113 | | 0 | | 1 | FALSE | 0.5 | TRUE | Amp | N |
| C2 | LIG4 | SYBR | None | 25.19 | | 6.833 | | 0 | | 1 | FALSE | 0.5 | TRUE | Amp | N |
| C3 | MGMT | SYBR | None | 21.521 | | 3.164 | | 0 | | 1 | FALSE | 0.5 | TRUE | Amp | N |
| C4 | MLH1 | SYBR | None | 22.313 | | 3.956 | | 0 | | 1 | FALSE | 0.5 | TRUE | Amp | N |
| C5 | MLH3 | SYBR | None | 25.104 | | 6.747 | | 0 | | 1 | FALSE | 0.5 | TRUE | Amp | N |
| C6 | MMS19 | SYBR | None | 25.741 | | 7.384 | | 0 | | 1 | FALSE | 0.5 | TRUE | Amp | N |
| C7 | MPG | SYBR | None | 21.43 | | 3.073 | | 0 | | 1 | FALSE | 0.5 | TRUE | Amp | N |
| C8 | MRE11A | SYBR | None | 18.293 | | -0.064 | | 0 | | 1 | FALSE | 0.5 | TRUE | Amp | N |
| C9 | MSH2 | SYBR | None | 16.726 | | -1.631 | | 0 | | 1 | FALSE | 0.5 | TRUE | Amp | N |
| C10 | MSH3 | SYBR | None | 19.716 | | 1.359 | | 0 | | 1 | FALSE | 0.5 | TRUE | Amp | N |
| C11 | MSH4 | SYBR | None | 20.889 | | 2.532 | | 0 | | 1 | FALSE | 0.5 | TRUE | Amp | N |
| C12 | MSH5 | SYBR | None | 28.713 | | 10.356 | | 0 | | 1 | FALSE | 0.5 | TRUE | Amp | N |
| D1 | MSH6 | SYBR | None | 20.759 | | 2.402 | | 0 | | 1 | FALSE | 0.5 | TRUE | Amp | N |
| D2 | MUTYH | SYBR | None | 23.769 | | 5.412 | | 0 | | 1 | FALSE | 0.5 | TRUE | Amp | N |
| D3 | NEIL1 | SYBR | None | 26.98 | | 8.623 | | 0 | | 1 | FALSE | 0.5 | TRUE | Amp | N |
| D4 | NEIL2 | SYBR | None | 23.928 | | 5.571 | | 0 | | 1 | FALSE | 0.5 | TRUE | Amp | N |
| D5 | NEIL3 | SYBR | None | 21.221 | | 2.864 | | 0 | | 1 | FALSE | 0.5 | TRUE | Amp | N |
| D6 | NTHL1 | SYBR | None | 20.955 | | 2.598 | | 0 | | 1 | FALSE | 0.5 | TRUE | Amp | N |
| D7 | OGG1 | SYBR | None | 21.954 | | 3.597 | | 0 | | 1 | FALSE | 0.5 | TRUE | Amp | N |
| D8 | PARP1 | SYBR | None | 21.033 | | 2.676 | | 0 | | 1 | FALSE | 0.5 | TRUE | Amp | N |
| D9 | PARP2 | SYBR | None | 21.07 | | 2.713 | | 0 | | 1 | FALSE | 0.5 | TRUE | Amp | N |
| D10 | PARP3 | SYBR | None | 24.088 | | 5.731 | | 0 | | 1 | FALSE | 0.5 | TRUE | Amp | N |
| D11 | PMS1 | SYBR | None | 18.261 | | -0.096 | | 0 | | 1 | FALSE | 0.5 | TRUE | Amp | N |
| D12 | PMS2 | SYBR | None | 22.199 | | 3.842 | | 0 | | 1 | FALSE | 0.5 | TRUE | Amp | N |
| E1 | PNKP | SYBR | None | 24.578 | | 6.221 | | 0 | | 1 | FALSE | 0.5 | TRUE | Amp | N |
| E2 | POLB | SYBR | None | 17.712 | | -0.645 | | 0 | | 1 | FALSE | 0.5 | TRUE | Amp | N |
| E3 | POLD3 | SYBR | None | 20.122 | | 1.765 | | 0 | | 1 | FALSE | 0.5 | TRUE | Amp | N |
| E4 | POLL | SYBR | None | 24.874 | | 6.517 | | 0 | | 1 | FALSE | 0.5 | TRUE | Amp | N |
| E5 | PRKDC | SYBR | None | 23.816 | | 5.459 | | 0 | | 1 | FALSE | 0.5 | TRUE | Amp | N |
| E6 | RAD18 | SYBR | None | 17.925 | | -0.432 | | 0 | | 1 | FALSE | 0.5 | TRUE | Amp | N |
| E7 | RAD21 | SYBR | None | 18.492 | | 0.135 | | 0 | | 1 | FALSE | 0.5 | TRUE | Amp | N |
| E8 | RAD23A | SYBR | None | 22.49 | | 4.133 | | 0 | | 1 | FALSE | 0.5 | TRUE | Amp | N |
| E9 | RAD23B | SYBR | None | 24.651 | | 6.294 | | 0 | | 1 | FALSE | 0.5 | TRUE | Amp | N |
| E10 | RAD50 | SYBR | None | 19.973 | | 1.616 | | 0 | | 1 | FALSE | 0.5 | TRUE | Amp | N |
| E11 | RAD51 | SYBR | None | 21.398 | | 3.041 | | 0 | | 1 | FALSE | 0.5 | TRUE | Amp | N |
| E12 | RAD51B | SYBR | None | 20.179 | | 1.822 | | 0 | | 1 | FALSE | 0.5 | TRUE | Amp | N |
| F1 | RAD51C | SYBR | None | 19.064 | | 0.707 | | 0 | | 1 | FALSE | 0.5 | TRUE | Amp | N |
| F2 | RAD51D | SYBR | None | 23.879 | | 5.522 | | 0 | | 1 | FALSE | 0.5 | TRUE | Amp | N |
| F3 | RAD52 | SYBR | None | 25.957 | | 7.6 | | 0 | | 1 | FALSE | 0.5 | TRUE | Amp | N |
| F4 | RAD54L | SYBR | None | 22.751 | | 4.394 | | 0 | | 1 | FALSE | 0.5 | TRUE | Amp | N |
| F5 | RFC1 | SYBR | None | 23.912 | | 5.555 | | 0 | | 1 | FALSE | 0.5 | TRUE | Amp | N |
| F6 | RPA1 | SYBR | None | 17.443 | | -0.914 | | 0 | | 1 | FALSE | 0.5 | TRUE | Amp | N |
| F7 | RPA3 | SYBR | None | 23.702 | | 5.345 | | 0 | | 1 | FALSE | 0.5 | TRUE | Amp | N |
| F8 | SLK | SYBR | None | 20.916 | | 2.559 | | 0 | | 1 | FALSE | 0.5 | TRUE | Amp | N |
| F9 | SMUG1 | SYBR | None | 26.83 | | 8.473 | | 0 | | 1 | FALSE | 0.5 | TRUE | Amp | N |
| F10 | TDG | SYBR | None | 21.958 | | 3.601 | | 0 | | 1 | FALSE | 0.5 | TRUE | Amp | N |
| F11 | TOP3A | SYBR | None | 25.041 | | 6.684 | | 0 | | 1 | FALSE | 0.5 | TRUE | Amp | N |
| F12 | TOP3B | SYBR | None | 27.607 | | 9.25 | | 0 | | 1 | FALSE | 0.5 | TRUE | Amp | N |
| G1 | TREX1 | SYBR | None | 27.991 | | 9.634 | | 0 | | 1 | FALSE | 0.5 | TRUE | Amp | N |
| G2 | UNG | SYBR | None | 19.907 | | 1.55 | | 0 | | 1 | FALSE | 0.5 | TRUE | Amp | N |
| G3 | XAB2 | SYBR | None | 23.751 | | 5.394 | | 0 | | 1 | FALSE | 0.5 | TRUE | Amp | N |
| G4 | XPA | SYBR | None | 31.411 | | 13.054 | | 0 | | 1 | FALSE | 0.5 | TRUE | Amp | N |
| G5 | XPC | SYBR | None | 25.458 | | 7.101 | | 0 | | 1 | FALSE | 0.5 | TRUE | Amp | N |
| G6 | XRCC1 | SYBR | None | 20.635 | | 2.278 | | 0 | | 1 | FALSE | 0.5 | TRUE | Amp | N |
| G7 | XRCC2 | SYBR | None | 21.759 | | 3.402 | | 0 | | 1 | FALSE | 0.5 | TRUE | Amp | N |
| G8 | XRCC3 | SYBR | None | 23.81 | | 5.453 | | 0 | | 1 | FALSE | 0.5 | TRUE | Amp | N |
| G9 | XRCC4 | SYBR | None | 25.047 | | 6.69 | | 0 | | 1 | FALSE | 0.5 | TRUE | Amp | N |
| G10 | XRCC5 | SYBR | None | 17.538 | | -0.819 | | 0 | | 1 | FALSE | 0.5 | TRUE | Amp | N |
| G11 | XRCC6 | SYBR | None | 19.463 | | 1.106 | | 0 | | 1 | FALSE | 0.5 | TRUE | Amp | N |
| G12 | XRCC6BP1 | SYBR | None | 23.891 | | 5.534 | | 0 | | 1 | FALSE | 0.5 | TRUE | Amp | N |
| H1 | ACTB | SYBR | None | 18.322 | |  | |  | |  | FALSE | 0.5 | TRUE | Amp | N |
| H2 | B2M | SYBR | None | 16.781 | |  | |  | |  | FALSE | 0.5 | TRUE | Amp | N |
| H3 | GAPDH | SYBR | None | 20.805 | |  | |  | |  | FALSE | 0.5 | TRUE | Amp | N |
| H4 | HPRT1 | SYBR | None | 20.691 | |  | |  | |  | FALSE | 0.5 | TRUE | Amp | N |
| H5 | RPLP0 | SYBR | None | 15.186 | |  | |  | |  | FALSE | 0.5 | TRUE | Amp | N |
| **Control 2** |  |  |  |  | |  | |  | |  |  |  |  |  |  |
| Well Position | Gene Symbol | Reporter | Quencher | CT | | Delta Ct | | Delta Delta Ct | | 2^(-Delta Delta Ct) | Automatic Ct Threshold | Ct Threshold | Automatic Baseline | Amp Status | MTP |
| A1 | APEX1 | SYBR | None | 18.174 | | -0.0784 | | -0.0294 | | 1.020588 | FALSE | 0.5 | TRUE | Amp | N |
| A2 | APEX2 | SYBR | None | 22.441 | | 4.1886 | | 0.4536 | | 0.730218 | FALSE | 0.5 | TRUE | Amp | N |
| A3 | ATM | SYBR | None | 22.687 | | 4.4346 | | -0.7094 | | 1.635124 | FALSE | 0.5 | TRUE | Amp | N |
| A4 | ATR | SYBR | None | 23.39 | | 5.1376 | | 0.6316 | | 0.64546 | FALSE | 0.5 | TRUE | Amp | N |
| A5 | ATXN3 | SYBR | None | 20.376 | | 2.1236 | | -0.0964 | | 1.069102 | FALSE | 0.5 | TRUE | Amp | N |
| A6 | BRCA1 | SYBR | None | 21.838 | | 3.5856 | | 0.3376 | | 0.791357 | FALSE | 0.5 | TRUE | Amp | N |
| A7 | BRCA2 | SYBR | None | 19.036 | | 0.7836 | | 0.3396 | | 0.79026 | FALSE | 0.5 | TRUE | Amp | N |
| A8 | BRIP1 | SYBR | None | 23.452 | | 5.1996 | | 0.4656 | | 0.72417 | FALSE | 0.5 | TRUE | Amp | N |
| A9 | CCNH | SYBR | None | 24.432 | | 6.1796 | | -0.3694 | | 1.291815 | FALSE | 0.5 | TRUE | Amp | N |
| A10 | CCNO | SYBR | None | 21.957 | | 3.7046 | | -0.5344 | | 1.44834 | FALSE | 0.5 | TRUE | Amp | N |
| A11 | CDK7 | SYBR | None | 14.454 | | -3.7984 | | 0.3526 | | 0.783171 | FALSE | 0.5 | TRUE | Amp | N |
| A12 | DDB1 | SYBR | None | 21.003 | | 2.7506 | | 0.3936 | | 0.761228 | FALSE | 0.5 | TRUE | Amp | N |
| B1 | DDB2 | SYBR | None | 23.117 | | 4.8646 | | -0.0304 | | 1.021295 | FALSE | 0.5 | TRUE | Amp | N |
| B2 | DMC1 | SYBR | None | 27.282 | | 9.0296 | | -0.0434 | | 1.03054 | FALSE | 0.5 | TRUE | Amp | N |
| B3 | ERCC1 | SYBR | None | 22.539 | | 4.2866 | | 0.4426 | | 0.735807 | FALSE | 0.5 | TRUE | Amp | N |
| B4 | ERCC2 | SYBR | None | 24.317 | | 6.0646 | | 0.3466 | | 0.786435 | FALSE | 0.5 | TRUE | Amp | N |
| B5 | ERCC3 | SYBR | None | 21.967 | | 3.7146 | | 0.5046 | | 0.704856 | FALSE | 0.5 | TRUE | Amp | N |
| B6 | ERCC4 | SYBR | None | 26.583 | | 8.3306 | | -1.0334 | | 2.046842 | FALSE | 0.5 | TRUE | Amp | N |
| B7 | ERCC5 | SYBR | None | 25.483 | | 7.2306 | | 0.5396 | | 0.687962 | FALSE | 0.5 | TRUE | Amp | N |
| B8 | ERCC6 | SYBR | None | 18.01 | | -0.2424 | | 0.2596 | | 0.835319 | FALSE | 0.5 | TRUE | Amp | N |
| B9 | ERCC8 | SYBR | None | 23.261 | | 5.0086 | | 0.6316 | | 0.64546 | FALSE | 0.5 | TRUE | Amp | N |
| B10 | EXO1 | SYBR | None | 20.451 | | 2.1986 | | 0.2726 | | 0.827826 | FALSE | 0.5 | TRUE | Amp | N |
| B11 | FEN1 | SYBR | None | 18.522 | | 0.2696 | | 0.9496 | | 0.517776 | FALSE | 0.5 | TRUE | Amp | N |
| B12 | LIG1 | SYBR | None | 22.549 | | 4.2966 | | 0.5806 | | 0.668686 | FALSE | 0.5 | TRUE | Amp | N |
| C1 | LIG3 | SYBR | None | 25.039 | | 6.7866 | | -0.3264 | | 1.253881 | FALSE | 0.5 | TRUE | Amp | N |
| C2 | LIG4 | SYBR | None | 25.014 | | 6.7616 | | -0.0714 | | 1.050736 | FALSE | 0.5 | TRUE | Amp | N |
| C3 | MGMT | SYBR | None | 21.318 | | 3.0656 | | -0.0984 | | 1.070585 | FALSE | 0.5 | TRUE | Amp | N |
| C4 | MLH1 | SYBR | None | 22.551 | | 4.2986 | | 0.3426 | | 0.788619 | FALSE | 0.5 | TRUE | Amp | N |
| C5 | MLH3 | SYBR | None | 24.92 | | 6.6676 | | -0.0794 | | 1.056579 | FALSE | 0.5 | TRUE | Amp | N |
| C6 | MMS19 | SYBR | None | 25.299 | | 7.0466 | | -0.3374 | | 1.263478 | FALSE | 0.5 | TRUE | Amp | N |
| C7 | MPG | SYBR | None | 21.284 | | 3.0316 | | -0.0414 | | 1.029112 | FALSE | 0.5 | TRUE | Amp | N |
| C8 | MRE11A | SYBR | None | 18.45 | | 0.1976 | | 0.2616 | | 0.834162 | FALSE | 0.5 | TRUE | Amp | N |
| C9 | MSH2 | SYBR | None | 16.909 | | -1.3434 | | 0.2876 | | 0.819264 | FALSE | 0.5 | TRUE | Amp | N |
| C10 | MSH3 | SYBR | None | 20.169 | | 1.9166 | | 0.5576 | | 0.679431 | FALSE | 0.5 | TRUE | Amp | N |
| C11 | MSH4 | SYBR | None | 21.299 | | 3.0466 | | 0.5146 | | 0.699987 | FALSE | 0.5 | TRUE | Amp | N |
| C12 | MSH5 | SYBR | None | 29.389 | | 11.1366 | | 0.7806 | | 0.582125 | FALSE | 0.5 | TRUE | Amp | N |
| D1 | MSH6 | SYBR | None | 20.566 | | 2.3136 | | -0.0884 | | 1.06319 | FALSE | 0.5 | TRUE | Amp | N |
| D2 | MUTYH | SYBR | None | 23.981 | | 5.7286 | | 0.3166 | | 0.80296 | FALSE | 0.5 | TRUE | Amp | N |
| D3 | NEIL1 | SYBR | None | 26.361 | | 8.1086 | | -0.5144 | | 1.4284 | FALSE | 0.5 | TRUE | Amp | N |
| D4 | NEIL2 | SYBR | None | 23.417 | | 5.1646 | | -0.4064 | | 1.325374 | FALSE | 0.5 | TRUE | Amp | N |
| D5 | NEIL3 | SYBR | None | 21.377 | | 3.1246 | | 0.2606 | | 0.834741 | FALSE | 0.5 | TRUE | Amp | N |
| D6 | NTHL1 | SYBR | None | 21.456 | | 3.2036 | | 0.6056 | | 0.657198 | FALSE | 0.5 | TRUE | Amp | N |
| D7 | OGG1 | SYBR | None | 21.528 | | 3.2756 | | -0.3214 | | 1.249543 | FALSE | 0.5 | TRUE | Amp | N |
| D8 | PARP1 | SYBR | None | 21.356 | | 3.1036 | | 0.4276 | | 0.743498 | FALSE | 0.5 | TRUE | Amp | N |
| D9 | PARP2 | SYBR | None | 21.524 | | 3.2716 | | 0.5586 | | 0.678961 | FALSE | 0.5 | TRUE | Amp | N |
| D10 | PARP3 | SYBR | None | 24.588 | | 6.3356 | | 0.6046 | | 0.657654 | FALSE | 0.5 | TRUE | Amp | N |
| D11 | PMS1 | SYBR | None | 18.431 | | 0.1786 | | 0.2746 | | 0.826679 | FALSE | 0.5 | TRUE | Amp | N |
| D12 | PMS2 | SYBR | None | 22.573 | | 4.3206 | | 0.4786 | | 0.717674 | FALSE | 0.5 | TRUE | Amp | N |
| E1 | PNKP | SYBR | None | 23.999 | | 5.7466 | | -0.4744 | | 1.38934 | FALSE | 0.5 | TRUE | Amp | N |
| E2 | POLB | SYBR | None | 18.006 | | -0.2464 | | 0.3986 | | 0.758594 | FALSE | 0.5 | TRUE | Amp | N |
| E3 | POLD3 | SYBR | None | 20.478 | | 2.2256 | | 0.4606 | | 0.726684 | FALSE | 0.5 | TRUE | Amp | N |
| E4 | POLL | SYBR | None | 24.439 | | 6.1866 | | -0.3304 | | 1.257362 | FALSE | 0.5 | TRUE | Amp | N |
| E5 | PRKDC | SYBR | None | 23.662 | | 5.4096 | | -0.0494 | | 1.034834 | FALSE | 0.5 | TRUE | Amp | N |
| E6 | RAD18 | SYBR | None | 18.132 | | -0.1204 | | 0.3116 | | 0.805748 | FALSE | 0.5 | TRUE | Amp | N |
| E7 | RAD21 | SYBR | None | 18.889 | | 0.6366 | | 0.5016 | | 0.706323 | FALSE | 0.5 | TRUE | Amp | N |
| E8 | RAD23A | SYBR | None | 22.018 | | 3.7656 | | -0.3674 | | 1.290026 | FALSE | 0.5 | TRUE | Amp | N |
| E9 | RAD23B | SYBR | None | 25.154 | | 6.9016 | | 0.6076 | | 0.656288 | FALSE | 0.5 | TRUE | Amp | N |
| E10 | RAD50 | SYBR | None | 20.237 | | 1.9846 | | 0.3686 | | 0.774534 | FALSE | 0.5 | TRUE | Amp | N |
| E11 | RAD51 | SYBR | None | 21.588 | | 3.3356 | | 0.2946 | | 0.815298 | FALSE | 0.5 | TRUE | Amp | N |
| E12 | RAD51B | SYBR | None | 20.391 | | 2.1386 | | 0.3166 | | 0.80296 | FALSE | 0.5 | TRUE | Amp | N |
| F1 | RAD51C | SYBR | None | 19.206 | | 0.9536 | | 0.2466 | | 0.84288 | FALSE | 0.5 | TRUE | Amp | N |
| F2 | RAD51D | SYBR | None | 23.664 | | 5.4116 | | -0.1104 | | 1.079528 | FALSE | 0.5 | TRUE | Amp | N |
| F3 | RAD52 | SYBR | None | 25.386 | | 7.1336 | | -0.4664 | | 1.381657 | FALSE | 0.5 | TRUE | Amp | N |
| F4 | RAD54L | SYBR | None | 22.997 | | 4.7446 | | 0.3506 | | 0.784258 | FALSE | 0.5 | TRUE | Amp | N |
| F5 | RFC1 | SYBR | None | 23.418 | | 5.1656 | | -0.3894 | | 1.309849 | FALSE | 0.5 | TRUE | Amp | N |
| F6 | RPA1 | SYBR | None | 17.231 | | -1.0214 | | -0.1074 | | 1.077285 | FALSE | 0.5 | TRUE | Amp | N |
| F7 | RPA3 | SYBR | None | 24.296 | | 6.0436 | | 0.6986 | | 0.61617 | FALSE | 0.5 | TRUE | Amp | N |
| F8 | SLK | SYBR | None | 21.117 | | 2.8646 | | 0.3056 | | 0.809106 | FALSE | 0.5 | TRUE | Amp | N |
| F9 | SMUG1 | SYBR | None | 26.286 | | 8.0336 | | -0.4394 | | 1.35604 | FALSE | 0.5 | TRUE | Amp | N |
| F10 | TDG | SYBR | None | 22.445 | | 4.1926 | | 0.5916 | | 0.663607 | FALSE | 0.5 | TRUE | Amp | N |
| F11 | TOP3A | SYBR | None | 24.817 | | 6.5646 | | -0.1194 | | 1.086283 | FALSE | 0.5 | TRUE | Amp | N |
| F12 | TOP3B | SYBR | None | 27.964 | | 9.7116 | | 0.4616 | | 0.72618 | FALSE | 0.5 | TRUE | Amp | N |
| G1 | TREX1 | SYBR | None | 28.477 | | 10.2246 | | 0.5906 | | 0.664067 | FALSE | 0.5 | TRUE | Amp | N |
| G2 | UNG | SYBR | None | 20.113 | | 1.8606 | | 0.3106 | | 0.806306 | FALSE | 0.5 | TRUE | Amp | N |
| G3 | XAB2 | SYBR | None | 23.561 | | 5.3086 | | -0.0854 | | 1.060982 | FALSE | 0.5 | TRUE | Amp | N |
| G4 | XPA | SYBR | None | 30.849 | | 12.5966 | | -0.4574 | | 1.373065 | FALSE | 0.5 | TRUE | Amp | N |
| G5 | XPC | SYBR | None | 25.029 | | 6.7766 | | -0.3244 | | 1.252144 | FALSE | 0.5 | TRUE | Amp | N |
| G6 | XRCC1 | SYBR | None | 20.212 | | 1.9596 | | -0.3184 | | 1.246947 | FALSE | 0.5 | TRUE | Amp | N |
| G7 | XRCC2 | SYBR | None | 22.241 | | 3.9886 | | 0.5866 | | 0.66591 | FALSE | 0.5 | TRUE | Amp | N |
| G8 | XRCC3 | SYBR | None | 23.657 | | 5.4046 | | -0.0484 | | 1.034117 | FALSE | 0.5 | TRUE | Amp | N |
| G9 | XRCC4 | SYBR | None | 25.469 | | 7.2166 | | 0.5266 | | 0.694189 | FALSE | 0.5 | TRUE | Amp | N |
| G10 | XRCC5 | SYBR | None | 17.945 | | -0.3074 | | 0.5116 | | 0.701444 | FALSE | 0.5 | TRUE | Amp | N |
| G11 | XRCC6 | SYBR | None | 19.338 | | 1.0856 | | -0.0204 | | 1.014241 | FALSE | 0.5 | TRUE | Amp | N |
| G12 | XRCC6BP1 | SYBR | None | 24.496 | | 6.2436 | | 0.7096 | | 0.61149 | FALSE | 0.5 | TRUE | Amp | N |
| H1 | ACTB | SYBR | None | 18.017 | |  | |  | |  | FALSE | 0.5 | TRUE | Amp | N |
| H2 | B2M | SYBR | None | 16.99 | |  | |  | |  | FALSE | 0.5 | TRUE | Amp | N |
| H3 | GAPDH | SYBR | None | 20.989 | |  | |  | |  | FALSE | 0.5 | TRUE | Amp | N |
| H4 | HPRT1 | SYBR | None | 20.361 | |  | |  | |  | FALSE | 0.5 | TRUE | Amp | N |
| H5 | RPLP0 | SYBR | None | 14.905 | |  | |  | |  | FALSE | 0.5 | TRUE | Amp | N |
| **Control 3** |  |  |  |  | |  | |  | |  |  |  |  |  |  |
| Well Position | Gene Symbol | Reporter | Quencher | CT | | Delta Ct | | Delta Delta Ct | | 2^(-Delta Delta Ct) | Automatic Ct Threshold | Ct Threshold | Automatic Baseline | Amp Status | MTP |
| A1 | APEX1 | SYBR | None | 18.695 | | 0.258 | | 0.307 | | 0.808321 | FALSE | 0.5 | TRUE | Amp | N |
| A2 | APEX2 | SYBR | None | 21.974 | | 3.537 | | -0.198 | | 1.147107 | FALSE | 0.5 | TRUE | Amp | N |
| A3 | ATM | SYBR | None | 24.162 | | 5.725 | | 0.581 | | 0.6685 | FALSE | 0.5 | TRUE | Amp | N |
| A4 | ATR | SYBR | None | 22.393 | | 3.956 | | -0.55 | | 1.464086 | FALSE | 0.5 | TRUE | Amp | N |
| A5 | ATXN3 | SYBR | None | 20.108 | | 1.671 | | -0.549 | | 1.463071 | FALSE | 0.5 | TRUE | Amp | N |
| A6 | BRCA1 | SYBR | None | 21.409 | | 2.972 | | -0.276 | | 1.210833 | FALSE | 0.5 | TRUE | Amp | N |
| A7 | BRCA2 | SYBR | None | 19.373 | | 0.936 | | 0.492 | | 0.711039 | FALSE | 0.5 | TRUE | Amp | N |
| A8 | BRIP1 | SYBR | None | 22.905 | | 4.468 | | -0.266 | | 1.202469 | FALSE | 0.5 | TRUE | Amp | N |
| A9 | CCNH | SYBR | None | 24.758 | | 6.321 | | -0.228 | | 1.17121 | FALSE | 0.5 | TRUE | Amp | N |
| A10 | CCNO | SYBR | None | 22.813 | | 4.376 | | 0.137 | | 0.909408 | FALSE | 0.5 | TRUE | Amp | N |
| A11 | CDK7 | SYBR | None | 14.698 | | -3.739 | | 0.412 | | 0.751581 | FALSE | 0.5 | TRUE | Amp | N |
| A12 | DDB1 | SYBR | None | 20.585 | | 2.148 | | -0.209 | | 1.155887 | FALSE | 0.5 | TRUE | Amp | N |
| B1 | DDB2 | SYBR | None | 22.848 | | 4.411 | | -0.484 | | 1.398616 | FALSE | 0.5 | TRUE | Amp | N |
| B2 | DMC1 | SYBR | None | 27.275 | | 8.838 | | -0.235 | | 1.176907 | FALSE | 0.5 | TRUE | Amp | N |
| B3 | ERCC1 | SYBR | None | 21.688 | | 3.251 | | -0.593 | | 1.50838 | FALSE | 0.5 | TRUE | Amp | N |
| B4 | ERCC2 | SYBR | None | 23.519 | | 5.082 | | -0.636 | | 1.554015 | FALSE | 0.5 | TRUE | Amp | N |
| B5 | ERCC3 | SYBR | None | 21.893 | | 3.456 | | 0.246 | | 0.843231 | FALSE | 0.5 | TRUE | Amp | N |
| B6 | ERCC4 | SYBR | None | 27.295 | | 8.858 | | -0.506 | | 1.420107 | FALSE | 0.5 | TRUE | Amp | N |
| B7 | ERCC5 | SYBR | None | 25.294 | | 6.857 | | 0.166 | | 0.89131 | FALSE | 0.5 | TRUE | Amp | N |
| B8 | ERCC6 | SYBR | None | 17.441 | | -0.996 | | -0.494 | | 1.408344 | FALSE | 0.5 | TRUE | Amp | N |
| B9 | ERCC8 | SYBR | None | 23.066 | | 4.629 | | 0.252 | | 0.839731 | FALSE | 0.5 | TRUE | Amp | N |
| B10 | EXO1 | SYBR | None | 19.876 | | 1.439 | | -0.487 | | 1.401527 | FALSE | 0.5 | TRUE | Amp | N |
| B11 | FEN1 | SYBR | None | 17.975 | | -0.462 | | 0.218 | | 0.859756 | FALSE | 0.5 | TRUE | Amp | N |
| B12 | LIG1 | SYBR | None | 22.389 | | 3.952 | | 0.236 | | 0.849096 | FALSE | 0.5 | TRUE | Amp | N |
| C1 | LIG3 | SYBR | None | 24.92 | | 6.483 | | -0.63 | | 1.547565 | FALSE | 0.5 | TRUE | Amp | N |
| C2 | LIG4 | SYBR | None | 24.785 | | 6.348 | | -0.485 | | 1.399586 | FALSE | 0.5 | TRUE | Amp | N |
| C3 | MGMT | SYBR | None | 21.353 | | 2.916 | | -0.248 | | 1.18756 | FALSE | 0.5 | TRUE | Amp | N |
| C4 | MLH1 | SYBR | None | 22.187 | | 3.75 | | -0.206 | | 1.153486 | FALSE | 0.5 | TRUE | Amp | N |
| C5 | MLH3 | SYBR | None | 25.754 | | 7.317 | | 0.57 | | 0.673617 | FALSE | 0.5 | TRUE | Amp | N |
| C6 | MMS19 | SYBR | None | 25.287 | | 6.85 | | -0.534 | | 1.447938 | FALSE | 0.5 | TRUE | Amp | N |
| C7 | MPG | SYBR | None | 22.055 | | 3.618 | | 0.545 | | 0.685391 | FALSE | 0.5 | TRUE | Amp | N |
| C8 | MRE11A | SYBR | None | 18.577 | | 0.14 | | 0.204 | | 0.86814 | FALSE | 0.5 | TRUE | Amp | N |
| C9 | MSH2 | SYBR | None | 16.303 | | -2.134 | | -0.503 | | 1.417157 | FALSE | 0.5 | TRUE | Amp | N |
| C10 | MSH3 | SYBR | None | 19.581 | | 1.144 | | -0.215 | | 1.160704 | FALSE | 0.5 | TRUE | Amp | N |
| C11 | MSH4 | SYBR | None | 20.501 | | 2.064 | | -0.468 | | 1.383191 | FALSE | 0.5 | TRUE | Amp | N |
| C12 | MSH5 | SYBR | None | 28.186 | | 9.749 | | -0.607 | | 1.523089 | FALSE | 0.5 | TRUE | Amp | N |
| D1 | MSH6 | SYBR | None | 20.645 | | 2.208 | | -0.194 | | 1.143931 | FALSE | 0.5 | TRUE | Amp | N |
| D2 | MUTYH | SYBR | None | 23.99 | | 5.553 | | 0.141 | | 0.90689 | FALSE | 0.5 | TRUE | Amp | N |
| D3 | NEIL1 | SYBR | None | 27.63 | | 9.193 | | 0.57 | | 0.673617 | FALSE | 0.5 | TRUE | Amp | N |
| D4 | NEIL2 | SYBR | None | 23.517 | | 5.08 | | -0.491 | | 1.405419 | FALSE | 0.5 | TRUE | Amp | N |
| D5 | NEIL3 | SYBR | None | 20.686 | | 2.249 | | -0.615 | | 1.531558 | FALSE | 0.5 | TRUE | Amp | N |
| D6 | NTHL1 | SYBR | None | 20.552 | | 2.115 | | -0.483 | | 1.397647 | FALSE | 0.5 | TRUE | Amp | N |
| D7 | OGG1 | SYBR | None | 21.546 | | 3.109 | | -0.488 | | 1.402499 | FALSE | 0.5 | TRUE | Amp | N |
| D8 | PARP1 | SYBR | None | 20.845 | | 2.408 | | -0.268 | | 1.204137 | FALSE | 0.5 | TRUE | Amp | N |
| D9 | PARP2 | SYBR | None | 20.606 | | 2.169 | | -0.544 | | 1.458009 | FALSE | 0.5 | TRUE | Amp | N |
| D10 | PARP3 | SYBR | None | 24.763 | | 6.326 | | 0.595 | | 0.662044 | FALSE | 0.5 | TRUE | Amp | N |
| D11 | PMS1 | SYBR | None | 18.475 | | 0.038 | | 0.134 | | 0.911301 | FALSE | 0.5 | TRUE | Amp | N |
| D12 | PMS2 | SYBR | None | 22.837 | | 4.4 | | 0.558 | | 0.679243 | FALSE | 0.5 | TRUE | Amp | N |
| E1 | PNKP | SYBR | None | 24.421 | | 5.984 | | -0.237 | | 1.178539 | FALSE | 0.5 | TRUE | Amp | N |
| E2 | POLB | SYBR | None | 18.001 | | -0.436 | | 0.209 | | 0.865137 | FALSE | 0.5 | TRUE | Amp | N |
| E3 | POLD3 | SYBR | None | 20.638 | | 2.201 | | 0.436 | | 0.739181 | FALSE | 0.5 | TRUE | Amp | N |
| E4 | POLL | SYBR | None | 24.804 | | 6.367 | | -0.15 | | 1.109569 | FALSE | 0.5 | TRUE | Amp | N |
| E5 | PRKDC | SYBR | None | 23.695 | | 5.258 | | -0.201 | | 1.149495 | FALSE | 0.5 | TRUE | Amp | N |
| E6 | RAD18 | SYBR | None | 18.392 | | -0.045 | | 0.387 | | 0.764718 | FALSE | 0.5 | TRUE | Amp | N |
| E7 | RAD21 | SYBR | None | 19.01 | | 0.573 | | 0.438 | | 0.738157 | FALSE | 0.5 | TRUE | Amp | N |
| E8 | RAD23A | SYBR | None | 22.114 | | 3.677 | | -0.456 | | 1.371733 | FALSE | 0.5 | TRUE | Amp | N |
| E9 | RAD23B | SYBR | None | 24.869 | | 6.432 | | 0.138 | | 0.908778 | FALSE | 0.5 | TRUE | Amp | N |
| E10 | RAD50 | SYBR | None | 20.263 | | 1.826 | | 0.21 | | 0.864537 | FALSE | 0.5 | TRUE | Amp | N |
| E11 | RAD51 | SYBR | None | 20.946 | | 2.509 | | -0.532 | | 1.445932 | FALSE | 0.5 | TRUE | Amp | N |
| E12 | RAD51B | SYBR | None | 20.733 | | 2.296 | | 0.474 | | 0.719966 | FALSE | 0.5 | TRUE | Amp | N |
| F1 | RAD51C | SYBR | None | 18.686 | | 0.249 | | -0.458 | | 1.373636 | FALSE | 0.5 | TRUE | Amp | N |
| F2 | RAD51D | SYBR | None | 23.932 | | 5.495 | | -0.027 | | 1.018891 | FALSE | 0.5 | TRUE | Amp | N |
| F3 | RAD52 | SYBR | None | 25.516 | | 7.079 | | -0.521 | | 1.43495 | FALSE | 0.5 | TRUE | Amp | N |
| F4 | RAD54L | SYBR | None | 22.683 | | 4.246 | | -0.148 | | 1.108032 | FALSE | 0.5 | TRUE | Amp | N |
| F5 | RFC1 | SYBR | None | 23.734 | | 5.297 | | -0.258 | | 1.19582 | FALSE | 0.5 | TRUE | Amp | N |
| F6 | RPA1 | SYBR | None | 17.343 | | -1.094 | | -0.18 | | 1.132884 | FALSE | 0.5 | TRUE | Amp | N |
| F7 | RPA3 | SYBR | None | 23.937 | | 5.5 | | 0.155 | | 0.898132 | FALSE | 0.5 | TRUE | Amp | N |
| F8 | SLK | SYBR | None | 20.785 | | 2.348 | | -0.211 | | 1.15749 | FALSE | 0.5 | TRUE | Amp | N |
| F9 | SMUG1 | SYBR | None | 26.365 | | 7.928 | | -0.545 | | 1.45902 | FALSE | 0.5 | TRUE | Amp | N |
| F10 | TDG | SYBR | None | 22.547 | | 4.11 | | 0.509 | | 0.702709 | FALSE | 0.5 | TRUE | Amp | N |
| F11 | TOP3A | SYBR | None | 24.621 | | 6.184 | | -0.5 | | 1.414214 | FALSE | 0.5 | TRUE | Amp | N |
| F12 | TOP3B | SYBR | None | 27.529 | | 9.092 | | -0.158 | | 1.115739 | FALSE | 0.5 | TRUE | Amp | N |
| G1 | TREX1 | SYBR | None | 28.047 | | 9.61 | | -0.024 | | 1.016775 | FALSE | 0.5 | TRUE | Amp | N |
| G2 | UNG | SYBR | None | 19.793 | | 1.356 | | -0.194 | | 1.143931 | FALSE | 0.5 | TRUE | Amp | N |
| G3 | XAB2 | SYBR | None | 23.586 | | 5.149 | | -0.245 | | 1.185093 | FALSE | 0.5 | TRUE | Amp | N |
| G4 | XPA | SYBR | None | 32.135 | | 13.698 | | 0.644 | | 0.639936 | FALSE | 0.5 | TRUE | Amp | N |
| G5 | XPC | SYBR | None | 25.766 | | 7.329 | | 0.228 | | 0.853818 | FALSE | 0.5 | TRUE | Amp | N |
| G6 | XRCC1 | SYBR | None | 20.504 | | 2.067 | | -0.211 | | 1.15749 | FALSE | 0.5 | TRUE | Amp | N |
| G7 | XRCC2 | SYBR | None | 22.356 | | 3.919 | | 0.517 | | 0.698823 | FALSE | 0.5 | TRUE | Amp | N |
| G8 | XRCC3 | SYBR | None | 23.657 | | 5.22 | | -0.233 | | 1.175276 | FALSE | 0.5 | TRUE | Amp | N |
| G9 | XRCC4 | SYBR | None | 25.681 | | 7.244 | | 0.554 | | 0.681129 | FALSE | 0.5 | TRUE | Amp | N |
| G10 | XRCC5 | SYBR | None | 18.037 | | -0.4 | | 0.419 | | 0.747943 | FALSE | 0.5 | TRUE | Amp | N |
| G11 | XRCC6 | SYBR | None | 19.974 | | 1.537 | | 0.431 | | 0.741747 | FALSE | 0.5 | TRUE | Amp | N |
| G12 | XRCC6BP1 | SYBR | None | 24.107 | | 5.67 | | 0.136 | | 0.910039 | FALSE | 0.5 | TRUE | Amp | N |
| H1 | ACTB | SYBR | None | 18.405 | |  | |  | |  | FALSE | 0.5 | TRUE | Amp | N |
| H2 | B2M | SYBR | None | 16.937 | |  | |  | |  | FALSE | 0.5 | TRUE | Amp | N |
| H3 | GAPDH | SYBR | None | 20.625 | |  | |  | |  | FALSE | 0.5 | TRUE | Amp | N |
| H4 | HPRT1 | SYBR | None | 20.872 | |  | |  | |  | FALSE | 0.5 | TRUE | Amp | N |
| H5 | RPLP0 | SYBR | None | 15.346 | |  | |  | |  | FALSE | 0.5 | TRUE | Amp | N |
| **Control 4** |  |  |  |  | |  | |  | |  |  |  |  |  |  |
| Well Position | Gene Symbol | Reporter | Quencher | CT | | Delta Ct | | Delta Delta Ct | | 2^(-Delta Delta Ct) | Automatic Ct Threshold | Ct Threshold | Automatic Baseline | Amp Status | MTP |
| A1 | APEX1 | SYBR | None | 19.053 | | 0.0744 | | 0.1234 | | 0.918022 | FALSE | 0.5 | TRUE | Amp | N |
| A2 | APEX2 | SYBR | None | 22.612 | | 3.6334 | | -0.1016 | | 1.072963 | FALSE | 0.5 | TRUE | Amp | N |
| A3 | ATM | SYBR | None | 23.503 | | 4.5244 | | -0.6196 | | 1.536449 | FALSE | 0.5 | TRUE | Amp | N |
| A4 | ATR | SYBR | None | 23.333 | | 4.3544 | | -0.1516 | | 1.110801 | FALSE | 0.5 | TRUE | Amp | N |
| A5 | ATXN3 | SYBR | None | 20.591 | | 1.6124 | | -0.6076 | | 1.523722 | FALSE | 0.5 | TRUE | Amp | N |
| A6 | BRCA1 | SYBR | None | 22.281 | | 3.3024 | | 0.0544 | | 0.962995 | FALSE | 0.5 | TRUE | Amp | N |
| A7 | BRCA2 | SYBR | None | 19.847 | | 0.8684 | | 0.4244 | | 0.745149 | FALSE | 0.5 | TRUE | Amp | N |
| A8 | BRIP1 | SYBR | None | 23.784 | | 4.8054 | | 0.0714 | | 0.951714 | FALSE | 0.5 | TRUE | Amp | N |
| A9 | CCNH | SYBR | None | 24.906 | | 5.9274 | | -0.6216 | | 1.538581 | FALSE | 0.5 | TRUE | Amp | N |
| A10 | CCNO | SYBR | None | 23.69 | | 4.7114 | | 0.4724 | | 0.720765 | FALSE | 0.5 | TRUE | Amp | N |
| A11 | CDK7 | SYBR | None | 15.13 | | -3.8486 | | 0.3024 | | 0.810902 | FALSE | 0.5 | TRUE | Amp | N |
| A12 | DDB1 | SYBR | None | 20.715 | | 1.7364 | | -0.6206 | | 1.537514 | FALSE | 0.5 | TRUE | Amp | N |
| B1 | DDB2 | SYBR | None | 23.409 | | 4.4304 | | -0.4646 | | 1.379935 | FALSE | 0.5 | TRUE | Amp | N |
| B2 | DMC1 | SYBR | None | 28.029 | | 9.0504 | | -0.0226 | | 1.015788 | FALSE | 0.5 | TRUE | Amp | N |
| B3 | ERCC1 | SYBR | None | 22.33 | | 3.3514 | | -0.4926 | | 1.406978 | FALSE | 0.5 | TRUE | Amp | N |
| B4 | ERCC2 | SYBR | None | 24.797 | | 5.8184 | | 0.1004 | | 0.932774 | FALSE | 0.5 | TRUE | Amp | N |
| B5 | ERCC3 | SYBR | None | 21.714 | | 2.7354 | | -0.4746 | | 1.389533 | FALSE | 0.5 | TRUE | Amp | N |
| B6 | ERCC4 | SYBR | None | 28.169 | | 9.1904 | | -0.1736 | | 1.127869 | FALSE | 0.5 | TRUE | Amp | N |
| B7 | ERCC5 | SYBR | None | 25.182 | | 6.2034 | | -0.4876 | | 1.40211 | FALSE | 0.5 | TRUE | Amp | N |
| B8 | ERCC6 | SYBR | None | 18.873 | | -0.1056 | | 0.3964 | | 0.759752 | FALSE | 0.5 | TRUE | Amp | N |
| B9 | ERCC8 | SYBR | None | 23.421 | | 4.4424 | | 0.0654 | | 0.95568 | FALSE | 0.5 | TRUE | Amp | N |
| B10 | EXO1 | SYBR | None | 21.318 | | 2.3394 | | 0.4134 | | 0.750852 | FALSE | 0.5 | TRUE | Amp | N |
| B11 | FEN1 | SYBR | None | 18.104 | | -0.8746 | | -0.1946 | | 1.144407 | FALSE | 0.5 | TRUE | Amp | N |
| B12 | LIG1 | SYBR | None | 22.612 | | 3.6334 | | -0.0826 | | 1.058925 | FALSE | 0.5 | TRUE | Amp | N |
| C1 | LIG3 | SYBR | None | 25.508 | | 6.5294 | | -0.5836 | | 1.498584 | FALSE | 0.5 | TRUE | Amp | N |
| C2 | LIG4 | SYBR | None | 25.309 | | 6.3304 | | -0.5026 | | 1.416765 | FALSE | 0.5 | TRUE | Amp | N |
| C3 | MGMT | SYBR | None | 21.65 | | 2.6714 | | -0.4926 | | 1.406978 | FALSE | 0.5 | TRUE | Amp | N |
| C4 | MLH1 | SYBR | None | 23.388 | | 4.4094 | | 0.4534 | | 0.73032 | FALSE | 0.5 | TRUE | Amp | N |
| C5 | MLH3 | SYBR | None | 25.672 | | 6.6934 | | -0.0536 | | 1.037851 | FALSE | 0.5 | TRUE | Amp | N |
| C6 | MMS19 | SYBR | None | 26.094 | | 7.1154 | | -0.2686 | | 1.204638 | FALSE | 0.5 | TRUE | Amp | N |
| C7 | MPG | SYBR | None | 21.946 | | 2.9674 | | -0.1056 | | 1.075942 | FALSE | 0.5 | TRUE | Amp | N |
| C8 | MRE11A | SYBR | None | 19.289 | | 0.3104 | | 0.3744 | | 0.771426 | FALSE | 0.5 | TRUE | Amp | N |
| C9 | MSH2 | SYBR | None | 17.41 | | -1.5686 | | 0.0624 | | 0.95767 | FALSE | 0.5 | TRUE | Amp | N |
| C10 | MSH3 | SYBR | None | 20.093 | | 1.1144 | | -0.2446 | | 1.184764 | FALSE | 0.5 | TRUE | Amp | N |
| C11 | MSH4 | SYBR | None | 21.302 | | 2.3234 | | -0.2086 | | 1.155566 | FALSE | 0.5 | TRUE | Amp | N |
| C12 | MSH5 | SYBR | None | 28.686 | | 9.7074 | | -0.6486 | | 1.567646 | FALSE | 0.5 | TRUE | Amp | N |
| D1 | MSH6 | SYBR | None | 21.828 | | 2.8494 | | 0.4474 | | 0.733363 | FALSE | 0.5 | TRUE | Amp | N |
| D2 | MUTYH | SYBR | None | 23.832 | | 4.8534 | | -0.5586 | | 1.472839 | FALSE | 0.5 | TRUE | Amp | N |
| D3 | NEIL1 | SYBR | None | 26.91 | | 7.9314 | | -0.6916 | | 1.615074 | FALSE | 0.5 | TRUE | Amp | N |
| D4 | NEIL2 | SYBR | None | 24.021 | | 5.0424 | | -0.5286 | | 1.442529 | FALSE | 0.5 | TRUE | Amp | N |
| D5 | NEIL3 | SYBR | None | 21.359 | | 2.3804 | | -0.4836 | | 1.398228 | FALSE | 0.5 | TRUE | Amp | N |
| D6 | NTHL1 | SYBR | None | 21.313 | | 2.3344 | | -0.2636 | | 1.200471 | FALSE | 0.5 | TRUE | Amp | N |
| D7 | OGG1 | SYBR | None | 22.43 | | 3.4514 | | -0.1456 | | 1.106191 | FALSE | 0.5 | TRUE | Amp | N |
| D8 | PARP1 | SYBR | None | 22.099 | | 3.1204 | | 0.4444 | | 0.73489 | FALSE | 0.5 | TRUE | Amp | N |
| D9 | PARP2 | SYBR | None | 21.618 | | 2.6394 | | -0.0736 | | 1.052339 | FALSE | 0.5 | TRUE | Amp | N |
| D10 | PARP3 | SYBR | None | 24.483 | | 5.5044 | | -0.2266 | | 1.170074 | FALSE | 0.5 | TRUE | Amp | N |
| D11 | PMS1 | SYBR | None | 19.037 | | 0.0584 | | 0.1544 | | 0.898506 | FALSE | 0.5 | TRUE | Amp | N |
| D12 | PMS2 | SYBR | None | 23.275 | | 4.2964 | | 0.4544 | | 0.729814 | FALSE | 0.5 | TRUE | Amp | N |
| E1 | PNKP | SYBR | None | 25.713 | | 6.7344 | | 0.5134 | | 0.700569 | FALSE | 0.5 | TRUE | Amp | N |
| E2 | POLB | SYBR | None | 18.112 | | -0.8666 | | -0.2216 | | 1.166026 | FALSE | 0.5 | TRUE | Amp | N |
| E3 | POLD3 | SYBR | None | 20.594 | | 1.6154 | | -0.1496 | | 1.109262 | FALSE | 0.5 | TRUE | Amp | N |
| E4 | POLL | SYBR | None | 24.793 | | 5.8144 | | -0.7026 | | 1.627435 | FALSE | 0.5 | TRUE | Amp | N |
| E5 | PRKDC | SYBR | None | 24.165 | | 5.1864 | | -0.2726 | | 1.207983 | FALSE | 0.5 | TRUE | Amp | N |
| E6 | RAD18 | SYBR | None | 18.516 | | -0.4626 | | -0.0306 | | 1.021437 | FALSE | 0.5 | TRUE | Amp | N |
| E7 | RAD21 | SYBR | None | 18.878 | | -0.1006 | | -0.2356 | | 1.177396 | FALSE | 0.5 | TRUE | Amp | N |
| E8 | RAD23A | SYBR | None | 23.584 | | 4.6054 | | 0.4724 | | 0.720765 | FALSE | 0.5 | TRUE | Amp | N |
| E9 | RAD23B | SYBR | None | 24.796 | | 5.8174 | | -0.4766 | | 1.391461 | FALSE | 0.5 | TRUE | Amp | N |
| E10 | RAD50 | SYBR | None | 21.009 | | 2.0304 | | 0.4144 | | 0.750331 | FALSE | 0.5 | TRUE | Amp | N |
| E11 | RAD51 | SYBR | None | 22.07 | | 3.0914 | | 0.0504 | | 0.965669 | FALSE | 0.5 | TRUE | Amp | N |
| E12 | RAD51B | SYBR | None | 20.724 | | 1.7454 | | -0.0766 | | 1.05453 | FALSE | 0.5 | TRUE | Amp | N |
| F1 | RAD51C | SYBR | None | 19.518 | | 0.5394 | | -0.1676 | | 1.123188 | FALSE | 0.5 | TRUE | Amp | N |
| F2 | RAD51D | SYBR | None | 23.879 | | 4.9004 | | -0.6216 | | 1.538581 | FALSE | 0.5 | TRUE | Amp | N |
| F3 | RAD52 | SYBR | None | 25.901 | | 6.9224 | | -0.6776 | | 1.599477 | FALSE | 0.5 | TRUE | Amp | N |
| F4 | RAD54L | SYBR | None | 22.855 | | 3.8764 | | -0.5176 | | 1.431572 | FALSE | 0.5 | TRUE | Amp | N |
| F5 | RFC1 | SYBR | None | 24.279 | | 5.3004 | | -0.2546 | | 1.193005 | FALSE | 0.5 | TRUE | Amp | N |
| F6 | RPA1 | SYBR | None | 18.231 | | -0.7476 | | 0.1664 | | 0.891063 | FALSE | 0.5 | TRUE | Amp | N |
| F7 | RPA3 | SYBR | None | 23.821 | | 4.8424 | | -0.5026 | | 1.416765 | FALSE | 0.5 | TRUE | Amp | N |
| F8 | SLK | SYBR | None | 21.409 | | 2.4304 | | -0.1286 | | 1.093232 | FALSE | 0.5 | TRUE | Amp | N |
| F9 | SMUG1 | SYBR | None | 28.033 | | 9.0544 | | 0.5814 | | 0.668315 | FALSE | 0.5 | TRUE | Amp | N |
| F10 | TDG | SYBR | None | 22.028 | | 3.0494 | | -0.5516 | | 1.46571 | FALSE | 0.5 | TRUE | Amp | N |
| F11 | TOP3A | SYBR | None | 25.626 | | 6.6474 | | -0.0366 | | 1.025694 | FALSE | 0.5 | TRUE | Amp | N |
| F12 | TOP3B | SYBR | None | 27.556 | | 8.5774 | | -0.6726 | | 1.593943 | FALSE | 0.5 | TRUE | Amp | N |
| G1 | TREX1 | SYBR | None | 28.093 | | 9.1144 | | -0.5196 | | 1.433558 | FALSE | 0.5 | TRUE | Amp | N |
| G2 | UNG | SYBR | None | 20.079 | | 1.1004 | | -0.4496 | | 1.365662 | FALSE | 0.5 | TRUE | Amp | N |
| G3 | XAB2 | SYBR | None | 23.705 | | 4.7264 | | -0.6676 | | 1.588428 | FALSE | 0.5 | TRUE | Amp | N |
| G4 | XPA | SYBR | None | 32.175 | | 13.1964 | | 0.1424 | | 0.906011 | FALSE | 0.5 | TRUE | Amp | N |
| G5 | XPC | SYBR | None | 26.129 | | 7.1504 | | 0.0494 | | 0.966338 | FALSE | 0.5 | TRUE | Amp | N |
| G6 | XRCC1 | SYBR | None | 21.31 | | 2.3314 | | 0.0534 | | 0.963663 | FALSE | 0.5 | TRUE | Amp | N |
| G7 | XRCC2 | SYBR | None | 22.138 | | 3.1594 | | -0.2426 | | 1.183123 | FALSE | 0.5 | TRUE | Amp | N |
| G8 | XRCC3 | SYBR | None | 23.906 | | 4.9274 | | -0.5256 | | 1.439532 | FALSE | 0.5 | TRUE | Amp | N |
| G9 | XRCC4 | SYBR | None | 26.176 | | 7.1974 | | 0.5074 | | 0.703489 | FALSE | 0.5 | TRUE | Amp | N |
| G10 | XRCC5 | SYBR | None | 18.252 | | -0.7266 | | 0.0924 | | 0.937961 | FALSE | 0.5 | TRUE | Amp | N |
| G11 | XRCC6 | SYBR | None | 19.959 | | 0.9804 | | -0.1256 | | 1.090961 | FALSE | 0.5 | TRUE | Amp | N |
| G12 | XRCC6BP1 | SYBR | None | 24.406 | | 5.4274 | | -0.1066 | | 1.076688 | FALSE | 0.5 | TRUE | Amp | N |
| H1 | ACTB | SYBR | None | 18.921 | |  | |  | |  | FALSE | 0.5 | TRUE | Amp | N |
| H2 | B2M | SYBR | None | 17.436 | |  | |  | |  | FALSE | 0.5 | TRUE | Amp | N |
| H3 | GAPDH | SYBR | None | 21.503 | |  | |  | |  | FALSE | 0.5 | TRUE | Amp | N |
| H4 | HPRT1 | SYBR | None | 21.213 | |  | |  | |  | FALSE | 0.5 | TRUE | Amp | N |
| H5 | RPLP0 | SYBR | None | 15.82 | |  | |  | |  | FALSE | 0.5 | TRUE | Amp | N |
| Control 5 |  |  |  |  | |  | |  | |  |  |  |  |  |  |
| Well Position | Gene Symbol | Reporter | Quencher | CT | | Delta Ct | | Delta Delta Ct | | 2^(-Delta Delta Ct) | Automatic Ct Threshold | Ct Threshold | Automatic Baseline | Amp Status | MTP |
| A1 | APEX1 | SYBR | None | 17.852 | | -0.6248 | | -0.5758 | | 1.490504 | FALSE | 0.5 | TRUE | Amp | N |
| A2 | APEX2 | SYBR | None | 22.698 | | 4.2212 | | 0.4862 | | 0.713903 | FALSE | 0.5 | TRUE | Amp | N |
| A3 | ATM | SYBR | None | 23.484 | | 5.0072 | | -0.1368 | | 1.099464 | FALSE | 0.5 | TRUE | Amp | N |
| A4 | ATR | SYBR | None | 23.479 | | 5.0022 | | 0.4962 | | 0.708972 | FALSE | 0.5 | TRUE | Amp | N |
| A5 | ATXN3 | SYBR | None | 20.767 | | 2.2902 | | 0.0702 | | 0.952506 | FALSE | 0.5 | TRUE | Amp | N |
| A6 | BRCA1 | SYBR | None | 21.893 | | 3.4162 | | 0.1682 | | 0.889952 | FALSE | 0.5 | TRUE | Amp | N |
| A7 | BRCA2 | SYBR | None | 19.12 | | 0.6432 | | 0.1992 | | 0.871033 | FALSE | 0.5 | TRUE | Amp | N |
| A8 | BRIP1 | SYBR | None | 22.987 | | 4.5102 | | -0.2238 | | 1.167805 | FALSE | 0.5 | TRUE | Amp | N |
| A9 | CCNH | SYBR | None | 25.012 | | 6.5352 | | -0.0138 | | 1.009611 | FALSE | 0.5 | TRUE | Amp | N |
| A10 | CCNO | SYBR | None | 23.232 | | 4.7552 | | 0.5162 | | 0.699211 | FALSE | 0.5 | TRUE | Amp | N |
| A11 | CDK7 | SYBR | None | 14.66 | | -3.8168 | | 0.3342 | | 0.793224 | FALSE | 0.5 | TRUE | Amp | N |
| A12 | DDB1 | SYBR | None | 20.24 | | 1.7632 | | -0.5938 | | 1.509217 | FALSE | 0.5 | TRUE | Amp | N |
| B1 | DDB2 | SYBR | None | 23.291 | | 4.8142 | | -0.0808 | | 1.057604 | FALSE | 0.5 | TRUE | Amp | N |
| B2 | DMC1 | SYBR | None | 28.121 | | 9.6442 | | 0.5712 | | 0.673057 | FALSE | 0.5 | TRUE | Amp | N |
| B3 | ERCC1 | SYBR | None | 21.841 | | 3.3642 | | -0.4798 | | 1.39455 | FALSE | 0.5 | TRUE | Amp | N |
| B4 | ERCC2 | SYBR | None | 24.119 | | 5.6422 | | -0.0758 | | 1.053945 | FALSE | 0.5 | TRUE | Amp | N |
| B5 | ERCC3 | SYBR | None | 21.103 | | 2.6262 | | -0.5838 | | 1.498792 | FALSE | 0.5 | TRUE | Amp | N |
| B6 | ERCC4 | SYBR | None | 28.053 | | 9.5762 | | 0.2122 | | 0.86322 | FALSE | 0.5 | TRUE | Amp | N |
| B7 | ERCC5 | SYBR | None | 24.886 | | 6.4092 | | -0.2818 | | 1.215711 | FALSE | 0.5 | TRUE | Amp | N |
| B8 | ERCC6 | SYBR | None | 18.374 | | -0.1028 | | 0.3992 | | 0.758279 | FALSE | 0.5 | TRUE | Amp | N |
| B9 | ERCC8 | SYBR | None | 22.826 | | 4.3492 | | -0.0278 | | 1.019456 | FALSE | 0.5 | TRUE | Amp | N |
| B10 | EXO1 | SYBR | None | 20.285 | | 1.8082 | | -0.1178 | | 1.085079 | FALSE | 0.5 | TRUE | Amp | N |
| B11 | FEN1 | SYBR | None | 17.977 | | -0.4998 | | 0.1802 | | 0.882581 | FALSE | 0.5 | TRUE | Amp | N |
| B12 | LIG1 | SYBR | None | 22.289 | | 3.8122 | | 0.0962 | | 0.935494 | FALSE | 0.5 | TRUE | Amp | N |
| C1 | LIG3 | SYBR | None | 24.938 | | 6.4612 | | -0.6518 | | 1.571127 | FALSE | 0.5 | TRUE | Amp | N |
| C2 | LIG4 | SYBR | None | 24.716 | | 6.2392 | | -0.5938 | | 1.509217 | FALSE | 0.5 | TRUE | Amp | N |
| C3 | MGMT | SYBR | None | 21.436 | | 2.9592 | | -0.2048 | | 1.152527 | FALSE | 0.5 | TRUE | Amp | N |
| C4 | MLH1 | SYBR | None | 22.178 | | 3.7012 | | -0.2548 | | 1.19317 | FALSE | 0.5 | TRUE | Amp | N |
| C5 | MLH3 | SYBR | None | 24.996 | | 6.5192 | | -0.2278 | | 1.171048 | FALSE | 0.5 | TRUE | Amp | N |
| C6 | MMS19 | SYBR | None | 25.562 | | 7.0852 | | -0.2988 | | 1.230121 | FALSE | 0.5 | TRUE | Amp | N |
| C7 | MPG | SYBR | None | 21.29 | | 2.8132 | | -0.2598 | | 1.197313 | FALSE | 0.5 | TRUE | Amp | N |
| C8 | MRE11A | SYBR | None | 18.371 | | -0.1058 | | -0.0418 | | 1.029397 | FALSE | 0.5 | TRUE | Amp | N |
| C9 | MSH2 | SYBR | None | 16.586 | | -1.8908 | | -0.2598 | | 1.197313 | FALSE | 0.5 | TRUE | Amp | N |
| C10 | MSH3 | SYBR | None | 19.812 | | 1.3352 | | -0.0238 | | 1.016634 | FALSE | 0.5 | TRUE | Amp | N |
| C11 | MSH4 | SYBR | None | 21.168 | | 2.6912 | | 0.1592 | | 0.895522 | FALSE | 0.5 | TRUE | Amp | N |
| C12 | MSH5 | SYBR | None | 29.115 | | 10.6382 | | 0.2822 | | 0.822336 | FALSE | 0.5 | TRUE | Amp | N |
| D1 | MSH6 | SYBR | None | 20.264 | | 1.7872 | | -0.6148 | | 1.531346 | FALSE | 0.5 | TRUE | Amp | N |
| D2 | MUTYH | SYBR | None | 23.364 | | 4.8872 | | -0.5248 | | 1.438734 | FALSE | 0.5 | TRUE | Amp | N |
| D3 | NEIL1 | SYBR | None | 26.458 | | 7.9812 | | -0.6418 | | 1.560275 | FALSE | 0.5 | TRUE | Amp | N |
| D4 | NEIL2 | SYBR | None | 24.539 | | 6.0622 | | 0.4912 | | 0.711433 | FALSE | 0.5 | TRUE | Amp | N |
| D5 | NEIL3 | SYBR | None | 21.786 | | 3.3092 | | 0.4452 | | 0.734482 | FALSE | 0.5 | TRUE | Amp | N |
| D6 | NTHL1 | SYBR | None | 20.796 | | 2.3192 | | -0.2788 | | 1.213185 | FALSE | 0.5 | TRUE | Amp | N |
| D7 | OGG1 | SYBR | None | 22.175 | | 3.6982 | | 0.1012 | | 0.932257 | FALSE | 0.5 | TRUE | Amp | N |
| D8 | PARP1 | SYBR | None | 20.861 | | 2.3842 | | -0.2918 | | 1.224167 | FALSE | 0.5 | TRUE | Amp | N |
| D9 | PARP2 | SYBR | None | 21.619 | | 3.1422 | | 0.4292 | | 0.742673 | FALSE | 0.5 | TRUE | Amp | N |
| D10 | PARP3 | SYBR | None | 24.121 | | 5.6442 | | -0.0868 | | 1.062012 | FALSE | 0.5 | TRUE | Amp | N |
| D11 | PMS1 | SYBR | None | 18.761 | | 0.2842 | | 0.3802 | | 0.768331 | FALSE | 0.5 | TRUE | Amp | N |
| D12 | PMS2 | SYBR | None | 22.578 | | 4.1012 | | 0.2592 | | 0.835551 | FALSE | 0.5 | TRUE | Amp | N |
| E1 | PNKP | SYBR | None | 24.143 | | 5.6662 | | -0.5548 | | 1.468965 | FALSE | 0.5 | TRUE | Amp | N |
| E2 | POLB | SYBR | None | 17.325 | | -1.1518 | | -0.5068 | | 1.420895 | FALSE | 0.5 | TRUE | Amp | N |
| E3 | POLD3 | SYBR | None | 20.387 | | 1.9102 | | 0.1452 | | 0.904254 | FALSE | 0.5 | TRUE | Amp | N |
| E4 | POLL | SYBR | None | 24.465 | | 5.9882 | | -0.5288 | | 1.442729 | FALSE | 0.5 | TRUE | Amp | N |
| E5 | PRKDC | SYBR | None | 23.44 | | 4.9632 | | -0.4958 | | 1.410102 | FALSE | 0.5 | TRUE | Amp | N |
| E6 | RAD18 | SYBR | None | 17.749 | | -0.7278 | | -0.2958 | | 1.227565 | FALSE | 0.5 | TRUE | Amp | N |
| E7 | RAD21 | SYBR | None | 19.055 | | 0.5782 | | 0.4432 | | 0.735501 | FALSE | 0.5 | TRUE | Amp | N |
| E8 | RAD23A | SYBR | None | 22.688 | | 4.2112 | | 0.0782 | | 0.947239 | FALSE | 0.5 | TRUE | Amp | N |
| E9 | RAD23B | SYBR | None | 24.228 | | 5.7512 | | -0.5428 | | 1.456797 | FALSE | 0.5 | TRUE | Amp | N |
| E10 | RAD50 | SYBR | None | 20.063 | | 1.5862 | | -0.0298 | | 1.020871 | FALSE | 0.5 | TRUE | Amp | N |
| E11 | RAD51 | SYBR | None | 21.981 | | 3.5042 | | 0.4632 | | 0.725376 | FALSE | 0.5 | TRUE | Amp | N |
| E12 | RAD51B | SYBR | None | 19.753 | | 1.2762 | | -0.5458 | | 1.45983 | FALSE | 0.5 | TRUE | Amp | N |
| F1 | RAD51C | SYBR | None | 18.728 | | 0.2512 | | -0.4558 | | 1.371543 | FALSE | 0.5 | TRUE | Amp | N |
| F2 | RAD51D | SYBR | None | 23.425 | | 4.9482 | | -0.5738 | | 1.488439 | FALSE | 0.5 | TRUE | Amp | N |
| F3 | RAD52 | SYBR | None | 26.005 | | 7.5282 | | -0.0718 | | 1.051027 | FALSE | 0.5 | TRUE | Amp | N |
| F4 | RAD54L | SYBR | None | 22.851 | | 4.3742 | | -0.0198 | | 1.013819 | FALSE | 0.5 | TRUE | Amp | N |
| F5 | RFC1 | SYBR | None | 23.774 | | 5.2972 | | -0.2578 | | 1.195654 | FALSE | 0.5 | TRUE | Amp | N |
| F6 | RPA1 | SYBR | None | 17.967 | | -0.5098 | | 0.4042 | | 0.755655 | FALSE | 0.5 | TRUE | Amp | N |
| F7 | RPA3 | SYBR | None | 23.583 | | 5.1062 | | -0.2388 | | 1.180011 | FALSE | 0.5 | TRUE | Amp | N |
| F8 | SLK | SYBR | None | 20.79 | | 2.3132 | | -0.2458 | | 1.18575 | FALSE | 0.5 | TRUE | Amp | N |
| F9 | SMUG1 | SYBR | None | 26.662 | | 8.1852 | | -0.2878 | | 1.220777 | FALSE | 0.5 | TRUE | Amp | N |
| F10 | TDG | SYBR | None | 21.486 | | 3.0092 | | -0.5918 | | 1.507126 | FALSE | 0.5 | TRUE | Amp | N |
| F11 | TOP3A | SYBR | None | 25.397 | | 6.9202 | | 0.2362 | | 0.848979 | FALSE | 0.5 | TRUE | Amp | N |
| F12 | TOP3B | SYBR | None | 27.573 | | 9.0962 | | -0.1538 | | 1.112496 | FALSE | 0.5 | TRUE | Amp | N |
| G1 | TREX1 | SYBR | None | 28.674 | | 10.1972 | | 0.5632 | | 0.676799 | FALSE | 0.5 | TRUE | Amp | N |
| G2 | UNG | SYBR | None | 19.551 | | 1.0742 | | -0.4758 | | 1.390689 | FALSE | 0.5 | TRUE | Amp | N |
| G3 | XAB2 | SYBR | None | 23.595 | | 5.1182 | | -0.2758 | | 1.210665 | FALSE | 0.5 | TRUE | Amp | N |
| G4 | XPA | SYBR | None | 31.625 | | 13.1482 | | 0.0942 | | 0.936792 | FALSE | 0.5 | TRUE | Amp | N |
| G5 | XPC | SYBR | None | 25.569 | | 7.0922 | | -0.0088 | | 1.006118 | FALSE | 0.5 | TRUE | Amp | N |
| G6 | XRCC1 | SYBR | None | 20.54 | | 2.0632 | | -0.2148 | | 1.160543 | FALSE | 0.5 | TRUE | Amp | N |
| G7 | XRCC2 | SYBR | None | 21.335 | | 2.8582 | | -0.5438 | | 1.457807 | FALSE | 0.5 | TRUE | Amp | N |
| G8 | XRCC3 | SYBR | None | 23.692 | | 5.2152 | | -0.2378 | | 1.179193 | FALSE | 0.5 | TRUE | Amp | N |
| G9 | XRCC4 | SYBR | None | 25.134 | | 6.6572 | | -0.0328 | | 1.022996 | FALSE | 0.5 | TRUE | Amp | N |
| G10 | XRCC5 | SYBR | None | 17.353 | | -1.1238 | | -0.3048 | | 1.235247 | FALSE | 0.5 | TRUE | Amp | N |
| G11 | XRCC6 | SYBR | None | 19.529 | | 1.0522 | | -0.0538 | | 1.037995 | FALSE | 0.5 | TRUE | Amp | N |
| G12 | XRCC6BP1 | SYBR | None | 23.978 | | 5.5012 | | -0.0328 | | 1.022996 | FALSE | 0.5 | TRUE | Amp | N |
| H1 | ACTB | SYBR | None | 18.562 | |  | |  | |  | FALSE | 0.5 | TRUE | Amp | N |
| H2 | B2M | SYBR | None | 16.893 | |  | |  | |  | FALSE | 0.5 | TRUE | Amp | N |
| H3 | GAPDH | SYBR | None | 21.075 | |  | |  | |  | FALSE | 0.5 | TRUE | Amp | N |
| H4 | HPRT1 | SYBR | None | 20.864 | |  | |  | |  | FALSE | 0.5 | TRUE | Amp | N |
| H5 | RPLP0 | SYBR | None | 14.99 | |  | |  | |  | FALSE | 0.5 | TRUE | Amp | N |
| POI 1 |  |  |  |  | |  | |  | |  |  |  |  |  |  |
| Well Position | Gene Symbol | Reporter | Quencher | CT | | Delta Ct | | Delta Delta Ct | | 2^(-Delta Delta Ct) | Automatic Ct Threshold | Ct Threshold | Automatic Baseline | Amp Status | MTP |
| A1 | APEX1 | SYBR | None | 18.215 | | -0.2114 | | -0.1624 | | 1.119147 | FALSE | 0.5 | TRUE | Amp | N |
| A2 | APEX2 | SYBR | None | 22.272 | | 3.8456 | | 0.1106 | | 0.926203 | FALSE | 0.5 | TRUE | Amp | N |
| A3 | ATM | SYBR | None | 23.378 | | 4.9516 | | -0.1924 | | 1.142663 | FALSE | 0.5 | TRUE | Amp | N |
| A4 | ATR | SYBR | None | 23.475 | | 5.0486 | | 0.5426 | | 0.686533 | FALSE | 0.5 | TRUE | Amp | N |
| A5 | ATXN3 | SYBR | None | 21.137 | | 2.7106 | | 0.4906 | | 0.711729 | FALSE | 0.5 | TRUE | Amp | N |
| A6 | BRCA1 | SYBR | None | 21.251 | | 2.8246 | | -0.4234 | | 1.341084 | FALSE | 0.5 | TRUE | Amp | N |
| A7 | BRCA2 | SYBR | None | 19.214 | | 0.7876 | | 0.3436 | | 0.788072 | FALSE | 0.5 | TRUE | Amp | N |
| A8 | BRIP1 | SYBR | None | 23.666 | | 5.2396 | | 0.5056 | | 0.704367 | FALSE | 0.5 | TRUE | Amp | N |
| A9 | CCNH | SYBR | None | 25.137 | | 6.7106 | | 0.1616 | | 0.894033 | FALSE | 0.5 | TRUE | Amp | N |
| A10 | CCNO | SYBR | None | 22.155 | | 3.7286 | | -0.5104 | | 1.424445 | FALSE | 0.5 | TRUE | Amp | N |
| A11 | CDK7 | SYBR | None | 14.425 | | -4.0014 | | 0.1496 | | 0.9015 | FALSE | 0.5 | TRUE | Amp | N |
| A12 | DDB1 | SYBR | None | 20.67 | | 2.2436 | | -0.1134 | | 1.081775 | FALSE | 0.5 | TRUE | Amp | N |
| B1 | DDB2 | SYBR | None | 23.85 | | 5.4236 | | 0.5286 | | 0.693227 | FALSE | 0.5 | TRUE | Amp | N |
| B2 | DMC1 | SYBR | None | 27.072 | | 8.6456 | | -0.4274 | | 1.344808 | FALSE | 0.5 | TRUE | Amp | N |
| B3 | ERCC1 | SYBR | None | 22.451 | | 4.0246 | | 0.1806 | | 0.882336 | FALSE | 0.5 | TRUE | Amp | N |
| B4 | ERCC2 | SYBR | None | 24.274 | | 5.8476 | | 0.1296 | | 0.914085 | FALSE | 0.5 | TRUE | Amp | N |
| B5 | ERCC3 | SYBR | None | 21.627 | | 3.2006 | | -0.0094 | | 1.006537 | FALSE | 0.5 | TRUE | Amp | N |
| B6 | ERCC4 | SYBR | None | 27.609 | | 9.1826 | | -0.1814 | | 1.133984 | FALSE | 0.5 | TRUE | Amp | N |
| B7 | ERCC5 | SYBR | None | 25.371 | | 6.9446 | | 0.2536 | | 0.838801 | FALSE | 0.5 | TRUE | Amp | N |
| B8 | ERCC6 | SYBR | None | 17.488 | | -0.9384 | | -0.4364 | | 1.353223 | FALSE | 0.5 | TRUE | Amp | N |
| B9 | ERCC8 | SYBR | None | 22.604 | | 4.1776 | | -0.1994 | | 1.148221 | FALSE | 0.5 | TRUE | Amp | N |
| B10 | EXO1 | SYBR | None | 20.786 | | 2.3596 | | 0.4336 | | 0.740412 | FALSE | 0.5 | TRUE | Amp | N |
| B11 | FEN1 | SYBR | None | 17.281 | | -1.1454 | | -0.4654 | | 1.3807 | FALSE | 0.5 | TRUE | Amp | N |
| B12 | LIG1 | SYBR | None | 22.116 | | 3.6896 | | -0.0264 | | 1.018468 | FALSE | 0.5 | TRUE | Amp | N |
| C1 | LIG3 | SYBR | None | 25.126 | | 6.6996 | | -0.4134 | | 1.331821 | FALSE | 0.5 | TRUE | Amp | N |
| C2 | LIG4 | SYBR | None | 25.351 | | 6.9246 | | 0.0916 | | 0.938481 | FALSE | 0.5 | TRUE | Amp | N |
| C3 | MGMT | SYBR | None | 21.479 | | 3.0526 | | -0.1114 | | 1.080276 | FALSE | 0.5 | TRUE | Amp | N |
| C4 | MLH1 | SYBR | None | 22.216 | | 3.7896 | | -0.1664 | | 1.122255 | FALSE | 0.5 | TRUE | Amp | N |
| C5 | MLH3 | SYBR | None | 24.75 | | 6.3236 | | -0.4234 | | 1.341084 | FALSE | 0.5 | TRUE | Amp | N |
| C6 | MMS19 | SYBR | None | 25.629 | | 7.2026 | | -0.1814 | | 1.133984 | FALSE | 0.5 | TRUE | Amp | N |
| C7 | MPG | SYBR | None | 21.101 | | 2.6746 | | -0.3984 | | 1.318045 | FALSE | 0.5 | TRUE | Amp | N |
| C8 | MRE11A | SYBR | None | 18.457 | | 0.0306 | | 0.0946 | | 0.936532 | FALSE | 0.5 | TRUE | Amp | N |
| C9 | MSH2 | SYBR | None | 16.662 | | -1.7644 | | -0.1334 | | 1.096876 | FALSE | 0.5 | TRUE | Amp | N |
| C10 | MSH3 | SYBR | None | 19.42 | | 0.9936 | | -0.3654 | | 1.288239 | FALSE | 0.5 | TRUE | Amp | N |
| C11 | MSH4 | SYBR | None | 20.478 | | 2.0516 | | -0.4804 | | 1.39513 | FALSE | 0.5 | TRUE | Amp | N |
| C12 | MSH5 | SYBR | None | 28.312 | | 9.8856 | | -0.4704 | | 1.385494 | FALSE | 0.5 | TRUE | Amp | N |
| D1 | MSH6 | SYBR | None | 20.353 | | 1.9266 | | -0.4754 | | 1.390304 | FALSE | 0.5 | TRUE | Amp | N |
| D2 | MUTYH | SYBR | None | 23.422 | | 4.9956 | | -0.4164 | | 1.334593 | FALSE | 0.5 | TRUE | Amp | N |
| D3 | NEIL1 | SYBR | None | 26.84 | | 8.4136 | | -0.2094 | | 1.156207 | FALSE | 0.5 | TRUE | Amp | N |
| D4 | NEIL2 | SYBR | None | 24.104 | | 5.6776 | | 0.1066 | | 0.928774 | FALSE | 0.5 | TRUE | Amp | N |
| D5 | NEIL3 | SYBR | None | 21.113 | | 2.6866 | | -0.1774 | | 1.130844 | FALSE | 0.5 | TRUE | Amp | N |
| D6 | NTHL1 | SYBR | None | 20.51 | | 2.0836 | | -0.5144 | | 1.4284 | FALSE | 0.5 | TRUE | Amp | N |
| D7 | OGG1 | SYBR | None | 21.563 | | 3.1366 | | -0.4604 | | 1.375923 | FALSE | 0.5 | TRUE | Amp | N |
| D8 | PARP1 | SYBR | None | 20.643 | | 2.2166 | | -0.4594 | | 1.37497 | FALSE | 0.5 | TRUE | Amp | N |
| D9 | PARP2 | SYBR | None | 21.617 | | 3.1906 | | 0.4776 | | 0.718171 | FALSE | 0.5 | TRUE | Amp | N |
| D10 | PARP3 | SYBR | None | 24.699 | | 6.2726 | | 0.5416 | | 0.687009 | FALSE | 0.5 | TRUE | Amp | N |
| D11 | PMS1 | SYBR | None | 17.938 | | -0.4884 | | -0.3924 | | 1.312575 | FALSE | 0.5 | TRUE | Amp | N |
| D12 | PMS2 | SYBR | None | 22.769 | | 4.3426 | | 0.5006 | | 0.706813 | FALSE | 0.5 | TRUE | Amp | N |
| E1 | PNKP | SYBR | None | 24.462 | | 6.0356 | | -0.1854 | | 1.137132 | FALSE | 0.5 | TRUE | Amp | N |
| E2 | POLB | SYBR | None | 17.362 | | -1.0644 | | -0.4194 | | 1.337371 | FALSE | 0.5 | TRUE | Amp | N |
| E3 | POLD3 | SYBR | None | 20.372 | | 1.9456 | | 0.1806 | | 0.882336 | FALSE | 0.5 | TRUE | Amp | N |
| E4 | POLL | SYBR | None | 24.462 | | 6.0356 | | -0.4814 | | 1.396098 | FALSE | 0.5 | TRUE | Amp | N |
| E5 | PRKDC | SYBR | None | 23.343 | | 4.9166 | | -0.5424 | | 1.456393 | FALSE | 0.5 | TRUE | Amp | N |
| E6 | RAD18 | SYBR | None | 18.157 | | -0.2694 | | 0.1626 | | 0.893414 | FALSE | 0.5 | TRUE | Amp | N |
| E7 | RAD21 | SYBR | None | 18.559 | | 0.1326 | | -0.0024 | | 1.001665 | FALSE | 0.5 | TRUE | Amp | N |
| E8 | RAD23A | SYBR | None | 22.15 | | 3.7236 | | -0.4094 | | 1.328133 | FALSE | 0.5 | TRUE | Amp | N |
| E9 | RAD23B | SYBR | None | 24.277 | | 5.8506 | | -0.4434 | | 1.359805 | FALSE | 0.5 | TRUE | Amp | N |
| E10 | RAD50 | SYBR | None | 20.464 | | 2.0376 | | 0.4216 | | 0.746596 | FALSE | 0.5 | TRUE | Amp | N |
| E11 | RAD51 | SYBR | None | 21.576 | | 3.1496 | | 0.1086 | | 0.927488 | FALSE | 0.5 | TRUE | Amp | N |
| E12 | RAD51B | SYBR | None | 20.382 | | 1.9556 | | 0.1336 | | 0.911554 | FALSE | 0.5 | TRUE | Amp | N |
| F1 | RAD51C | SYBR | None | 19.372 | | 0.9456 | | 0.2386 | | 0.847567 | FALSE | 0.5 | TRUE | Amp | N |
| F2 | RAD51D | SYBR | None | 24.469 | | 6.0426 | | 0.5206 | | 0.697082 | FALSE | 0.5 | TRUE | Amp | N |
| F3 | RAD52 | SYBR | None | 25.588 | | 7.1616 | | -0.4384 | | 1.355101 | FALSE | 0.5 | TRUE | Amp | N |
| F4 | RAD54L | SYBR | None | 22.637 | | 4.2106 | | -0.1834 | | 1.135557 | FALSE | 0.5 | TRUE | Amp | N |
| F5 | RFC1 | SYBR | None | 23.774 | | 5.3476 | | -0.2074 | | 1.154605 | FALSE | 0.5 | TRUE | Amp | N |
| F6 | RPA1 | SYBR | None | 17.13 | | -1.2964 | | -0.3824 | | 1.303509 | FALSE | 0.5 | TRUE | Amp | N |
| F7 | RPA3 | SYBR | None | 24.291 | | 5.8646 | | 0.5196 | | 0.697565 | FALSE | 0.5 | TRUE | Amp | N |
| F8 | SLK | SYBR | None | 21.172 | | 2.7456 | | 0.1866 | | 0.878674 | FALSE | 0.5 | TRUE | Amp | N |
| F9 | SMUG1 | SYBR | None | 27.106 | | 8.6796 | | 0.2066 | | 0.866577 | FALSE | 0.5 | TRUE | Amp | N |
| F10 | TDG | SYBR | None | 22.498 | | 4.0716 | | 0.4706 | | 0.721664 | FALSE | 0.5 | TRUE | Amp | N |
| F11 | TOP3A | SYBR | None | 24.616 | | 6.1896 | | -0.4944 | | 1.408735 | FALSE | 0.5 | TRUE | Amp | N |
| F12 | TOP3B | SYBR | None | 27.575 | | 9.1486 | | -0.1014 | | 1.072814 | FALSE | 0.5 | TRUE | Amp | N |
| G1 | TREX1 | SYBR | None | 27.515 | | 9.0886 | | -0.5454 | | 1.459425 | FALSE | 0.5 | TRUE | Amp | N |
| G2 | UNG | SYBR | None | 19.595 | | 1.1686 | | -0.3814 | | 1.302605 | FALSE | 0.5 | TRUE | Amp | N |
| G3 | XAB2 | SYBR | None | 23.373 | | 4.9466 | | -0.4474 | | 1.363581 | FALSE | 0.5 | TRUE | Amp | N |
| G4 | XPA | SYBR | None | 31.275 | | 12.8486 | | -0.2054 | | 1.153006 | FALSE | 0.5 | TRUE | Amp | N |
| G5 | XPC | SYBR | None | 25.648 | | 7.2216 | | 0.1206 | | 0.919805 | FALSE | 0.5 | TRUE | Amp | N |
| G6 | XRCC1 | SYBR | None | 21.209 | | 2.7826 | | 0.5046 | | 0.704856 | FALSE | 0.5 | TRUE | Amp | N |
| G7 | XRCC2 | SYBR | None | 22.271 | | 3.8446 | | 0.4426 | | 0.735807 | FALSE | 0.5 | TRUE | Amp | N |
| G8 | XRCC3 | SYBR | None | 23.745 | | 5.3186 | | -0.1344 | | 1.097636 | FALSE | 0.5 | TRUE | Amp | N |
| G9 | XRCC4 | SYBR | None | 25.667 | | 7.2406 | | 0.5506 | | 0.682736 | FALSE | 0.5 | TRUE | Amp | N |
| G10 | XRCC5 | SYBR | None | 17.963 | | -0.4634 | | 0.3556 | | 0.781545 | FALSE | 0.5 | TRUE | Amp | N |
| G11 | XRCC6 | SYBR | None | 19.136 | | 0.7096 | | -0.3964 | | 1.316219 | FALSE | 0.5 | TRUE | Amp | N |
| G12 | XRCC6BP1 | SYBR | None | 23.408 | | 4.9816 | | -0.5524 | | 1.466523 | FALSE | 0.5 | TRUE | Amp | N |
| H1 | ACTB | SYBR | None | 18.282 | |  | |  | |  | FALSE | 0.5 | TRUE | Amp | N |
| H2 | B2M | SYBR | None | 16.969 | |  | |  | |  | FALSE | 0.5 | TRUE | Amp | N |
| H3 | GAPDH | SYBR | None | 21.007 | |  | |  | |  | FALSE | 0.5 | TRUE | Amp | N |
| H4 | HPRT1 | SYBR | None | 20.541 | |  | |  | |  | FALSE | 0.5 | TRUE | Amp | N |
| H5 | RPLP0 | SYBR | None | 15.333 | |  | |  | |  | FALSE | 0.5 | TRUE | Amp | N |
| POI 2 | Dead during in vitro culture. | | |  | |  | |  | |  |  |  |  |  |  |
| POI 3 |  |  |  |  | |  | |  | |  |  |  |  |  |  |
| Well Position | Gene Symbol | Reporter | Quencher | CT | | Delta Ct | | Delta Delta Ct | | 2^(-Delta Delta Ct) | Automatic Ct Threshold | Ct Threshold | Automatic Baseline | Amp Status | MTP |
| A1 | APEX1 | SYBR | None | 18.179 | | -0.165 | | -0.116 | | 1.083726 | FALSE | 0.5 | TRUE | Amp | N |
| A2 | APEX2 | SYBR | None | 22.353 | | 4.009 | | 0.274 | | 0.827023 | FALSE | 0.5 | TRUE | Amp | N |
| A3 | ATM | SYBR | None | 23.73 | | 5.386 | | 0.242 | | 0.845572 | FALSE | 0.5 | TRUE | Amp | N |
| A4 | ATR | SYBR | None | 22.692 | | 4.348 | | -0.158 | | 1.115739 | FALSE | 0.5 | TRUE | Amp | N |
| A5 | ATXN3 | SYBR | None | 20.175 | | 1.831 | | -0.389 | | 1.309485 | FALSE | 0.5 | TRUE | Amp | N |
| A6 | BRCA1 | SYBR | None | 21.257 | | 2.913 | | -0.335 | | 1.261377 | FALSE | 0.5 | TRUE | Amp | N |
| A7 | BRCA2 | SYBR | None | 18.725 | | 0.381 | | -0.063 | | 1.044636 | FALSE | 0.5 | TRUE | Amp | N |
| A8 | BRIP1 | SYBR | None | 22.678 | | 4.334 | | -0.4 | | 1.319508 | FALSE | 0.5 | TRUE | Amp | N |
| A9 | CCNH | SYBR | None | 25.117 | | 6.773 | | 0.224 | | 0.856188 | FALSE | 0.5 | TRUE | Amp | N |
| A10 | CCNO | SYBR | None | 22.478 | | 4.134 | | -0.105 | | 1.075494 | FALSE | 0.5 | TRUE | Amp | N |
| A11 | CDK7 | SYBR | None | 14.425 | | -3.919 | | 0.232 | | 0.851454 | FALSE | 0.5 | TRUE | Amp | N |
| A12 | DDB1 | SYBR | None | 20.583 | | 2.239 | | -0.118 | | 1.085229 | FALSE | 0.5 | TRUE | Amp | N |
| B1 | DDB2 | SYBR | None | 23.847 | | 5.503 | | 0.608 | | 0.656106 | FALSE | 0.5 | TRUE | Amp | N |
| B2 | DMC1 | SYBR | None | 27.261 | | 8.917 | | -0.156 | | 1.114194 | FALSE | 0.5 | TRUE | Amp | N |
| B3 | ERCC1 | SYBR | None | 21.835 | | 3.491 | | -0.353 | | 1.277214 | FALSE | 0.5 | TRUE | Amp | N |
| B4 | ERCC2 | SYBR | None | 24.721 | | 6.377 | | 0.659 | | 0.633317 | FALSE | 0.5 | TRUE | Amp | N |
| B5 | ERCC3 | SYBR | None | 21.134 | | 2.79 | | -0.42 | | 1.337928 | FALSE | 0.5 | TRUE | Amp | N |
| B6 | ERCC4 | SYBR | None | 27.589 | | 9.245 | | -0.119 | | 1.085982 | FALSE | 0.5 | TRUE | Amp | N |
| B7 | ERCC5 | SYBR | None | 25.72 | | 7.376 | | 0.685 | | 0.622006 | FALSE | 0.5 | TRUE | Amp | N |
| B8 | ERCC6 | SYBR | None | 18.342 | | -0.002 | | 0.5 | | 0.707107 | FALSE | 0.5 | TRUE | Amp | N |
| B9 | ERCC8 | SYBR | None | 22.566 | | 4.222 | | -0.155 | | 1.113422 | FALSE | 0.5 | TRUE | Amp | N |
| B10 | EXO1 | SYBR | None | 19.947 | | 1.603 | | -0.323 | | 1.250929 | FALSE | 0.5 | TRUE | Amp | N |
| B11 | FEN1 | SYBR | None | 18.189 | | -0.155 | | 0.525 | | 0.694959 | FALSE | 0.5 | TRUE | Amp | N |
| B12 | LIG1 | SYBR | None | 21.724 | | 3.38 | | -0.336 | | 1.262252 | FALSE | 0.5 | TRUE | Amp | N |
| C1 | LIG3 | SYBR | None | 26.093 | | 7.749 | | 0.636 | | 0.643495 | FALSE | 0.5 | TRUE | Amp | N |
| C2 | LIG4 | SYBR | None | 25.379 | | 7.035 | | 0.202 | | 0.869345 | FALSE | 0.5 | TRUE | Amp | N |
| C3 | MGMT | SYBR | None | 21.365 | | 3.021 | | -0.143 | | 1.104199 | FALSE | 0.5 | TRUE | Amp | N |
| C4 | MLH1 | SYBR | None | 22.173 | | 3.829 | | -0.127 | | 1.092021 | FALSE | 0.5 | TRUE | Amp | N |
| C5 | MLH3 | SYBR | None | 25.606 | | 7.262 | | 0.515 | | 0.699793 | FALSE | 0.5 | TRUE | Amp | N |
| C6 | MMS19 | SYBR | None | 25.578 | | 7.234 | | -0.15 | | 1.109569 | FALSE | 0.5 | TRUE | Amp | N |
| C7 | MPG | SYBR | None | 21.058 | | 2.714 | | -0.359 | | 1.282537 | FALSE | 0.5 | TRUE | Amp | N |
| C8 | MRE11A | SYBR | None | 18.804 | | 0.46 | | 0.524 | | 0.695441 | FALSE | 0.5 | TRUE | Amp | N |
| C9 | MSH2 | SYBR | None | 17.236 | | -1.108 | | 0.523 | | 0.695923 | FALSE | 0.5 | TRUE | Amp | N |
| C10 | MSH3 | SYBR | None | 19.375 | | 1.031 | | -0.328 | | 1.255272 | FALSE | 0.5 | TRUE | Amp | N |
| C11 | MSH4 | SYBR | None | 20.722 | | 2.378 | | -0.154 | | 1.11265 | FALSE | 0.5 | TRUE | Amp | N |
| C12 | MSH5 | SYBR | None | 28.298 | | 9.954 | | -0.402 | | 1.321338 | FALSE | 0.5 | TRUE | Amp | N |
| D1 | MSH6 | SYBR | None | 21.301 | | 2.957 | | 0.555 | | 0.680657 | FALSE | 0.5 | TRUE | Amp | N |
| D2 | MUTYH | SYBR | None | 24.306 | | 5.962 | | 0.55 | | 0.68302 | FALSE | 0.5 | TRUE | Amp | N |
| D3 | NEIL1 | SYBR | None | 27.52 | | 9.176 | | 0.553 | | 0.681601 | FALSE | 0.5 | TRUE | Amp | N |
| D4 | NEIL2 | SYBR | None | 24.175 | | 5.831 | | 0.26 | | 0.835088 | FALSE | 0.5 | TRUE | Amp | N |
| D5 | NEIL3 | SYBR | None | 20.814 | | 2.47 | | -0.394 | | 1.314032 | FALSE | 0.5 | TRUE | Amp | N |
| D6 | NTHL1 | SYBR | None | 21.167 | | 2.823 | | 0.225 | | 0.855595 | FALSE | 0.5 | TRUE | Amp | N |
| D7 | OGG1 | SYBR | None | 22.531 | | 4.187 | | 0.59 | | 0.664343 | FALSE | 0.5 | TRUE | Amp | N |
| D8 | PARP1 | SYBR | None | 21.301 | | 2.957 | | 0.281 | | 0.82302 | FALSE | 0.5 | TRUE | Amp | N |
| D9 | PARP2 | SYBR | None | 21.648 | | 3.304 | | 0.591 | | 0.663883 | FALSE | 0.5 | TRUE | Amp | N |
| D10 | PARP3 | SYBR | None | 24.722 | | 6.378 | | 0.647 | | 0.638607 | FALSE | 0.5 | TRUE | Amp | N |
| D11 | PMS1 | SYBR | None | 18.463 | | 0.119 | | 0.215 | | 0.861546 | FALSE | 0.5 | TRUE | Amp | N |
| D12 | PMS2 | SYBR | None | 22.704 | | 4.36 | | 0.518 | | 0.698339 | FALSE | 0.5 | TRUE | Amp | N |
| E1 | PNKP | SYBR | None | 24.092 | | 5.748 | | -0.473 | | 1.387993 | FALSE | 0.5 | TRUE | Amp | N |
| E2 | POLB | SYBR | None | 17.653 | | -0.691 | | -0.046 | | 1.032399 | FALSE | 0.5 | TRUE | Amp | N |
| E3 | POLD3 | SYBR | None | 19.958 | | 1.614 | | -0.151 | | 1.110339 | FALSE | 0.5 | TRUE | Amp | N |
| E4 | POLL | SYBR | None | 24.424 | | 6.08 | | -0.437 | | 1.353786 | FALSE | 0.5 | TRUE | Amp | N |
| E5 | PRKDC | SYBR | None | 23.34 | | 4.996 | | -0.463 | | 1.378405 | FALSE | 0.5 | TRUE | Amp | N |
| E6 | RAD18 | SYBR | None | 17.556 | | -0.788 | | -0.356 | | 1.279872 | FALSE | 0.5 | TRUE | Amp | N |
| E7 | RAD21 | SYBR | None | 18.116 | | -0.228 | | -0.363 | | 1.286097 | FALSE | 0.5 | TRUE | Amp | N |
| E8 | RAD23A | SYBR | None | 23.021 | | 4.677 | | 0.544 | | 0.685867 | FALSE | 0.5 | TRUE | Amp | N |
| E9 | RAD23B | SYBR | None | 25.319 | | 6.975 | | 0.681 | | 0.623733 | FALSE | 0.5 | TRUE | Amp | N |
| E10 | RAD50 | SYBR | None | 20.164 | | 1.82 | | 0.204 | | 0.86814 | FALSE | 0.5 | TRUE | Amp | N |
| E11 | RAD51 | SYBR | None | 20.938 | | 2.594 | | -0.447 | | 1.363203 | FALSE | 0.5 | TRUE | Amp | N |
| E12 | RAD51B | SYBR | None | 20.744 | | 2.4 | | 0.578 | | 0.669892 | FALSE | 0.5 | TRUE | Amp | N |
| F1 | RAD51C | SYBR | None | 18.676 | | 0.332 | | -0.375 | | 1.29684 | FALSE | 0.5 | TRUE | Amp | N |
| F2 | RAD51D | SYBR | None | 24.459 | | 6.115 | | 0.593 | | 0.662963 | FALSE | 0.5 | TRUE | Amp | N |
| F3 | RAD52 | SYBR | None | 26.577 | | 8.233 | | 0.633 | | 0.644834 | FALSE | 0.5 | TRUE | Amp | N |
| F4 | RAD54L | SYBR | None | 23.294 | | 4.95 | | 0.556 | | 0.680185 | FALSE | 0.5 | TRUE | Amp | N |
| F5 | RFC1 | SYBR | None | 24.161 | | 5.817 | | 0.262 | | 0.833931 | FALSE | 0.5 | TRUE | Amp | N |
| F6 | RPA1 | SYBR | None | 17.975 | | -0.369 | | 0.545 | | 0.685391 | FALSE | 0.5 | TRUE | Amp | N |
| F7 | RPA3 | SYBR | None | 22.723 | | 4.379 | | -0.966 | | 1.953417 | FALSE | 0.5 | TRUE | Amp | N |
| F8 | SLK | SYBR | None | 20.53 | | 2.186 | | -0.373 | | 1.295043 | FALSE | 0.5 | TRUE | Amp | N |
| F9 | SMUG1 | SYBR | None | 26.317 | | 7.973 | | -0.5 | | 1.414214 | FALSE | 0.5 | TRUE | Amp | N |
| F10 | TDG | SYBR | None | 21.553 | | 3.209 | | -0.392 | | 1.312211 | FALSE | 0.5 | TRUE | Amp | N |
| F11 | TOP3A | SYBR | None | 25.303 | | 6.959 | | 0.275 | | 0.82645 | FALSE | 0.5 | TRUE | Amp | N |
| F12 | TOP3B | SYBR | None | 27.482 | | 9.138 | | -0.112 | | 1.080725 | FALSE | 0.5 | TRUE | Amp | N |
| G1 | TREX1 | SYBR | None | 27.454 | | 9.11 | | -0.524 | | 1.437937 | FALSE | 0.5 | TRUE | Amp | N |
| G2 | UNG | SYBR | None | 19.995 | | 1.651 | | 0.101 | | 0.932386 | FALSE | 0.5 | TRUE | Amp | N |
| G3 | XAB2 | SYBR | None | 23.63 | | 5.286 | | -0.108 | | 1.077733 | FALSE | 0.5 | TRUE | Amp | N |
| G4 | XPA | SYBR | None | 31.278 | | 12.934 | | -0.12 | | 1.086735 | FALSE | 0.5 | TRUE | Amp | N |
| G5 | XPC | SYBR | None | 24.987 | | 6.643 | | -0.458 | | 1.373636 | FALSE | 0.5 | TRUE | Amp | N |
| G6 | XRCC1 | SYBR | None | 20.222 | | 1.878 | | -0.4 | | 1.319508 | FALSE | 0.5 | TRUE | Amp | N |
| G7 | XRCC2 | SYBR | None | 21.965 | | 3.621 | | 0.219 | | 0.859161 | FALSE | 0.5 | TRUE | Amp | N |
| G8 | XRCC3 | SYBR | None | 23.41 | | 5.066 | | -0.387 | | 1.307671 | FALSE | 0.5 | TRUE | Amp | N |
| G9 | XRCC4 | SYBR | None | 24.56 | | 6.216 | | -0.474 | | 1.388955 | FALSE | 0.5 | TRUE | Amp | N |
| G10 | XRCC5 | SYBR | None | 17.214 | | -1.13 | | -0.311 | | 1.240567 | FALSE | 0.5 | TRUE | Amp | N |
| G11 | XRCC6 | SYBR | None | 19.667 | | 1.323 | | 0.217 | | 0.860353 | FALSE | 0.5 | TRUE | Amp | N |
| G12 | XRCC6BP1 | SYBR | None | 24.163 | | 5.819 | | 0.285 | | 0.820742 | FALSE | 0.5 | TRUE | Amp | N |
| H1 | ACTB | SYBR | None | 18.237 | |  | |  | |  | FALSE | 0.5 | TRUE | Amp | N |
| H2 | B2M | SYBR | None | 16.915 | |  | |  | |  | FALSE | 0.5 | TRUE | Amp | N |
| H3 | GAPDH | SYBR | None | 20.751 | |  | |  | |  | FALSE | 0.5 | TRUE | Amp | N |
| H4 | HPRT1 | SYBR | None | 20.819 | |  | |  | |  | FALSE | 0.5 | TRUE | Amp | N |
| H5 | RPLP0 | SYBR | None | 14.998 | |  | |  | |  | FALSE | 0.5 | TRUE | Amp | N |
| POI 4 |  |  |  |  | |  | |  | |  |  |  |  |  |  |
| Well Position | Gene Symbol | Reporter | Quencher | CT | | Delta Ct | | Delta Delta Ct | | 2^(-Delta Delta Ct) | Automatic Ct Threshold | Ct Threshold | Automatic Baseline | Amp Status | MTP |
| A1 | APEX1 | SYBR | None | 18.573 | | 0.2678 | | 0.3168 | | 0.802849 | FALSE | 0.5 | TRUE | Amp | N |
| A2 | APEX2 | SYBR | None | 22.329 | | 4.0238 | | 0.2888 | | 0.818583 | FALSE | 0.5 | TRUE | Amp | N |
| A3 | ATM | SYBR | None | 23.946 | | 5.6408 | | 0.4968 | | 0.708677 | FALSE | 0.5 | TRUE | Amp | N |
| A4 | ATR | SYBR | None | 23.057 | | 4.7518 | | 0.2458 | | 0.843348 | FALSE | 0.5 | TRUE | Amp | N |
| A5 | ATXN3 | SYBR | None | 21.097 | | 2.7918 | | 0.5718 | | 0.672777 | FALSE | 0.5 | TRUE | Amp | N |
| A6 | BRCA1 | SYBR | None | 21.261 | | 2.9558 | | -0.2922 | | 1.224506 | FALSE | 0.5 | TRUE | Amp | N |
| A7 | BRCA2 | SYBR | None | 20.523 | | 2.2178 | | 1.7738 | | 0.292437 | FALSE | 0.5 | TRUE | Amp | N |
| A8 | BRIP1 | SYBR | None | 23.294 | | 4.9888 | | 0.2548 | | 0.838103 | FALSE | 0.5 | TRUE | Amp | N |
| A9 | CCNH | SYBR | None | 25.178 | | 6.8728 | | 0.3238 | | 0.798963 | FALSE | 0.5 | TRUE | Amp | N |
| A10 | CCNO | SYBR | None | 23.202 | | 4.8968 | | 0.6578 | | 0.633844 | FALSE | 0.5 | TRUE | Amp | N |
| A11 | CDK7 | SYBR | None | 14.403 | | -3.9022 | | 0.2488 | | 0.841596 | FALSE | 0.5 | TRUE | Amp | N |
| A12 | DDB1 | SYBR | None | 20.591 | | 2.2858 | | -0.0712 | | 1.05059 | FALSE | 0.5 | TRUE | Amp | N |
| B1 | DDB2 | SYBR | None | 23.488 | | 5.1828 | | 0.2878 | | 0.81915 | FALSE | 0.5 | TRUE | Amp | N |
| B2 | DMC1 | SYBR | None | 26.991 | | 8.6858 | | -0.3872 | | 1.307853 | FALSE | 0.5 | TRUE | Amp | N |
| B3 | ERCC1 | SYBR | None | 22.096 | | 3.7908 | | -0.0532 | | 1.037564 | FALSE | 0.5 | TRUE | Amp | N |
| B4 | ERCC2 | SYBR | None | 24.283 | | 5.9778 | | 0.2598 | | 0.835204 | FALSE | 0.5 | TRUE | Amp | N |
| B5 | ERCC3 | SYBR | None | 22.177 | | 3.8718 | | 0.6618 | | 0.632089 | FALSE | 0.5 | TRUE | Amp | N |
| B6 | ERCC4 | SYBR | None | 27.611 | | 9.3058 | | -0.0582 | | 1.041166 | FALSE | 0.5 | TRUE | Amp | N |
| B7 | ERCC5 | SYBR | None | 25.23 | | 6.9248 | | 0.2338 | | 0.850392 | FALSE | 0.5 | TRUE | Amp | N |
| B8 | ERCC6 | SYBR | None | 18.356 | | 0.0508 | | 0.5528 | | 0.681696 | FALSE | 0.5 | TRUE | Amp | N |
| B9 | ERCC8 | SYBR | None | 22.615 | | 4.3098 | | -0.0672 | | 1.047681 | FALSE | 0.5 | TRUE | Amp | N |
| B10 | EXO1 | SYBR | None | 20.831 | | 2.5258 | | 0.5998 | | 0.659845 | FALSE | 0.5 | TRUE | Amp | N |
| B11 | FEN1 | SYBR | None | 17.337 | | -0.9682 | | -0.2882 | | 1.221116 | FALSE | 0.5 | TRUE | Amp | N |
| B12 | LIG1 | SYBR | None | 22.373 | | 4.0678 | | 0.3518 | | 0.783606 | FALSE | 0.5 | TRUE | Amp | N |
| C1 | LIG3 | SYBR | None | 25.694 | | 7.3888 | | 0.2758 | | 0.825992 | FALSE | 0.5 | TRUE | Amp | N |
| C2 | LIG4 | SYBR | None | 25.802 | | 7.4968 | | 0.6638 | | 0.631214 | FALSE | 0.5 | TRUE | Amp | N |
| C3 | MGMT | SYBR | None | 21.162 | | 2.8568 | | -0.3072 | | 1.237304 | FALSE | 0.5 | TRUE | Amp | N |
| C4 | MLH1 | SYBR | None | 22.002 | | 3.6968 | | -0.2592 | | 1.196815 | FALSE | 0.5 | TRUE | Amp | N |
| C5 | MLH3 | SYBR | None | 24.701 | | 6.3958 | | -0.3512 | | 1.275621 | FALSE | 0.5 | TRUE | Amp | N |
| C6 | MMS19 | SYBR | None | 25.362 | | 7.0568 | | -0.3272 | | 1.254576 | FALSE | 0.5 | TRUE | Amp | N |
| C7 | MPG | SYBR | None | 21.632 | | 3.3268 | | 0.2538 | | 0.838684 | FALSE | 0.5 | TRUE | Amp | N |
| C8 | MRE11A | SYBR | None | 18.806 | | 0.5008 | | 0.5648 | | 0.676049 | FALSE | 0.5 | TRUE | Amp | N |
| C9 | MSH2 | SYBR | None | 16.295 | | -2.0102 | | -0.3792 | | 1.30062 | FALSE | 0.5 | TRUE | Amp | N |
| C10 | MSH3 | SYBR | None | 20.034 | | 1.7288 | | 0.3698 | | 0.77389 | FALSE | 0.5 | TRUE | Amp | N |
| C11 | MSH4 | SYBR | None | 20.593 | | 2.2878 | | -0.2442 | | 1.184436 | FALSE | 0.5 | TRUE | Amp | N |
| C12 | MSH5 | SYBR | None | 28.623 | | 10.3178 | | -0.0382 | | 1.026832 | FALSE | 0.5 | TRUE | Amp | N |
| D1 | MSH6 | SYBR | None | 20.682 | | 2.3768 | | -0.0252 | | 1.017621 | FALSE | 0.5 | TRUE | Amp | N |
| D2 | MUTYH | SYBR | None | 24.317 | | 6.0118 | | 0.5998 | | 0.659845 | FALSE | 0.5 | TRUE | Amp | N |
| D3 | NEIL1 | SYBR | None | 26.547 | | 8.2418 | | -0.3812 | | 1.302425 | FALSE | 0.5 | TRUE | Amp | N |
| D4 | NEIL2 | SYBR | None | 23.505 | | 5.1998 | | -0.3712 | | 1.293428 | FALSE | 0.5 | TRUE | Amp | N |
| D5 | NEIL3 | SYBR | None | 20.92 | | 2.6148 | | -0.2492 | | 1.188548 | FALSE | 0.5 | TRUE | Amp | N |
| D6 | NTHL1 | SYBR | None | 20.627 | | 2.3218 | | -0.2762 | | 1.211001 | FALSE | 0.5 | TRUE | Amp | N |
| D7 | OGG1 | SYBR | None | 21.648 | | 3.3428 | | -0.2542 | | 1.192674 | FALSE | 0.5 | TRUE | Amp | N |
| D8 | PARP1 | SYBR | None | 21.417 | | 3.1118 | | 0.4358 | | 0.739284 | FALSE | 0.5 | TRUE | Amp | N |
| D9 | PARP2 | SYBR | None | 20.648 | | 2.3428 | | -0.3702 | | 1.292532 | FALSE | 0.5 | TRUE | Amp | N |
| D10 | PARP3 | SYBR | None | 24.712 | | 6.4068 | | 0.6758 | | 0.625985 | FALSE | 0.5 | TRUE | Amp | N |
| D11 | PMS1 | SYBR | None | 18.153 | | -0.1522 | | -0.0562 | | 1.039724 | FALSE | 0.5 | TRUE | Amp | N |
| D12 | PMS2 | SYBR | None | 22.078 | | 3.7728 | | -0.0692 | | 1.049135 | FALSE | 0.5 | TRUE | Amp | N |
| E1 | PNKP | SYBR | None | 24.215 | | 5.9098 | | -0.3112 | | 1.240739 | FALSE | 0.5 | TRUE | Amp | N |
| E2 | POLB | SYBR | None | 18.179 | | -0.1262 | | 0.5188 | | 0.697952 | FALSE | 0.5 | TRUE | Amp | N |
| E3 | POLD3 | SYBR | None | 20.394 | | 2.0888 | | 0.3238 | | 0.798963 | FALSE | 0.5 | TRUE | Amp | N |
| E4 | POLL | SYBR | None | 24.496 | | 6.1908 | | -0.3262 | | 1.253707 | FALSE | 0.5 | TRUE | Amp | N |
| E5 | PRKDC | SYBR | None | 24.247 | | 5.9418 | | 0.4828 | | 0.715587 | FALSE | 0.5 | TRUE | Amp | N |
| E6 | RAD18 | SYBR | None | 17.391 | | -0.9142 | | -0.4822 | | 1.396872 | FALSE | 0.5 | TRUE | Amp | N |
| E7 | RAD21 | SYBR | None | 19.046 | | 0.7408 | | 0.6058 | | 0.657107 | FALSE | 0.5 | TRUE | Amp | N |
| E8 | RAD23A | SYBR | None | 22.148 | | 3.8428 | | -0.2902 | | 1.22281 | FALSE | 0.5 | TRUE | Amp | N |
| E9 | RAD23B | SYBR | None | 24.538 | | 6.2328 | | -0.0612 | | 1.043333 | FALSE | 0.5 | TRUE | Amp | N |
| E10 | RAD50 | SYBR | None | 19.557 | | 1.2518 | | -0.3642 | | 1.287168 | FALSE | 0.5 | TRUE | Amp | N |
| E11 | RAD51 | SYBR | None | 21.893 | | 3.5878 | | 0.5468 | | 0.684537 | FALSE | 0.5 | TRUE | Amp | N |
| E12 | RAD51B | SYBR | None | 20.641 | | 2.3358 | | 0.5138 | | 0.700375 | FALSE | 0.5 | TRUE | Amp | N |
| F1 | RAD51C | SYBR | None | 19.597 | | 1.2918 | | 0.5848 | | 0.666742 | FALSE | 0.5 | TRUE | Amp | N |
| F2 | RAD51D | SYBR | None | 23.563 | | 5.2578 | | -0.2642 | | 1.20097 | FALSE | 0.5 | TRUE | Amp | N |
| F3 | RAD52 | SYBR | None | 26.556 | | 8.2508 | | 0.6508 | | 0.636927 | FALSE | 0.5 | TRUE | Amp | N |
| F4 | RAD54L | SYBR | None | 22.329 | | 4.0238 | | -0.3702 | | 1.292532 | FALSE | 0.5 | TRUE | Amp | N |
| F5 | RFC1 | SYBR | None | 23.59 | | 5.2848 | | -0.2702 | | 1.205975 | FALSE | 0.5 | TRUE | Amp | N |
| F6 | RPA1 | SYBR | None | 17.144 | | -1.1612 | | -0.2472 | | 1.186901 | FALSE | 0.5 | TRUE | Amp | N |
| F7 | RPA3 | SYBR | None | 23.597 | | 5.2918 | | -0.0532 | | 1.037564 | FALSE | 0.5 | TRUE | Amp | N |
| F8 | SLK | SYBR | None | 20.598 | | 2.2928 | | -0.2662 | | 1.202636 | FALSE | 0.5 | TRUE | Amp | N |
| F9 | SMUG1 | SYBR | None | 26.259 | | 7.9538 | | -0.5192 | | 1.43316 | FALSE | 0.5 | TRUE | Amp | N |
| F10 | TDG | SYBR | None | 22.476 | | 4.1708 | | 0.5698 | | 0.67371 | FALSE | 0.5 | TRUE | Amp | N |
| F11 | TOP3A | SYBR | None | 25.622 | | 7.3168 | | 0.6328 | | 0.644924 | FALSE | 0.5 | TRUE | Amp | N |
| F12 | TOP3B | SYBR | None | 27.528 | | 9.2228 | | -0.0272 | | 1.019032 | FALSE | 0.5 | TRUE | Amp | N |
| G1 | TREX1 | SYBR | None | 27.544 | | 9.2388 | | -0.3952 | | 1.315125 | FALSE | 0.5 | TRUE | Amp | N |
| G2 | UNG | SYBR | None | 20.253 | | 1.9478 | | 0.3978 | | 0.759015 | FALSE | 0.5 | TRUE | Amp | N |
| G3 | XAB2 | SYBR | None | 23.336 | | 5.0308 | | -0.3632 | | 1.286276 | FALSE | 0.5 | TRUE | Amp | N |
| G4 | XPA | SYBR | None | 32.058 | | 13.7528 | | 0.6988 | | 0.616084 | FALSE | 0.5 | TRUE | Amp | N |
| G5 | XPC | SYBR | None | 25.071 | | 6.7658 | | -0.3352 | | 1.261552 | FALSE | 0.5 | TRUE | Amp | N |
| G6 | XRCC1 | SYBR | None | 20.509 | | 2.2038 | | -0.0742 | | 1.052777 | FALSE | 0.5 | TRUE | Amp | N |
| G7 | XRCC2 | SYBR | None | 22.272 | | 3.9668 | | 0.5648 | | 0.676049 | FALSE | 0.5 | TRUE | Amp | N |
| G8 | XRCC3 | SYBR | None | 23.743 | | 5.4378 | | -0.0152 | | 1.010592 | FALSE | 0.5 | TRUE | Amp | N |
| G9 | XRCC4 | SYBR | None | 25.259 | | 6.9538 | | 0.2638 | | 0.832891 | FALSE | 0.5 | TRUE | Amp | N |
| G10 | XRCC5 | SYBR | None | 17.467 | | -0.8382 | | -0.0192 | | 1.013397 | FALSE | 0.5 | TRUE | Amp | N |
| G11 | XRCC6 | SYBR | None | 19.165 | | 0.8598 | | -0.2462 | | 1.186079 | FALSE | 0.5 | TRUE | Amp | N |
| G12 | XRCC6BP1 | SYBR | None | 24.399 | | 6.0938 | | 0.5598 | | 0.678396 | FALSE | 0.5 | TRUE | Amp | N |
| H1 | ACTB | SYBR | None | 18.179 | |  | |  | |  | FALSE | 0.5 | TRUE | Amp | N |
| H2 | B2M | SYBR | None | 16.574 | |  | |  | |  | FALSE | 0.5 | TRUE | Amp | N |
| H3 | GAPDH | SYBR | None | 20.937 | |  | |  | |  | FALSE | 0.5 | TRUE | Amp | N |
| H4 | HPRT1 | SYBR | None | 20.504 | |  | |  | |  | FALSE | 0.5 | TRUE | Amp | N |
| H5 | RPLP0 | SYBR | None | 15.332 | |  | |  | |  | FALSE | 0.5 | TRUE | Amp | N |
| POI 5 |  |  |  |  | |  | |  | |  |  |  |  |  |  |
| Well Position | Gene Symbol | Reporter | Quencher | CT | | Delta Ct | | Delta Delta Ct | | 2^(-Delta Delta Ct) | Automatic Ct Threshold | Ct Threshold | Automatic Baseline | Amp Status | MTP |
| A1 | APEX1 | SYBR | None | 18.932 | | 0.2106 | | 0.2596 | | 0.835319 | FALSE | 0.5 | TRUE | Amp | N |
| A2 | APEX2 | SYBR | None | 22.776 | | 4.0546 | | 0.3196 | | 0.801292 | FALSE | 0.5 | TRUE | Amp | N |
| A3 | ATM | SYBR | None | 23.364 | | 4.6426 | | -0.5014 | | 1.415587 | FALSE | 0.5 | TRUE | Amp | N |
| A4 | ATR | SYBR | None | 22.678 | | 3.9566 | | -0.5494 | | 1.463477 | FALSE | 0.5 | TRUE | Amp | N |
| A5 | ATXN3 | SYBR | None | 20.419 | | 1.6976 | | -0.5224 | | 1.436343 | FALSE | 0.5 | TRUE | Amp | N |
| A6 | BRCA1 | SYBR | None | 22.269 | | 3.5476 | | 0.2996 | | 0.812478 | FALSE | 0.5 | TRUE | Amp | N |
| A7 | BRCA2 | SYBR | None | 18.998 | | 0.2766 | | -0.1674 | | 1.123033 | FALSE | 0.5 | TRUE | Amp | N |
| A8 | BRIP1 | SYBR | None | 24.181 | | 5.4596 | | 0.7256 | | 0.604745 | FALSE | 0.5 | TRUE | Amp | N |
| A9 | CCNH | SYBR | None | 25.985 | | 7.2636 | | 0.7146 | | 0.609374 | FALSE | 0.5 | TRUE | Amp | N |
| A10 | CCNO | SYBR | None | 22.379 | | 3.6576 | | -0.5814 | | 1.496301 | FALSE | 0.5 | TRUE | Amp | N |
| A11 | CDK7 | SYBR | None | 14.873 | | -3.8484 | | 0.3026 | | 0.81079 | FALSE | 0.5 | TRUE | Amp | N |
| A12 | DDB1 | SYBR | None | 20.724 | | 2.0026 | | -0.3544 | | 1.278454 | FALSE | 0.5 | TRUE | Amp | N |
| B1 | DDB2 | SYBR | None | 23.906 | | 5.1846 | | 0.2896 | | 0.818129 | FALSE | 0.5 | TRUE | Amp | N |
| B2 | DMC1 | SYBR | None | 28.588 | | 9.8666 | | 0.7936 | | 0.576903 | FALSE | 0.5 | TRUE | Amp | N |
| B3 | ERCC1 | SYBR | None | 23.129 | | 4.4076 | | 0.5636 | | 0.676612 | FALSE | 0.5 | TRUE | Amp | N |
| B4 | ERCC2 | SYBR | None | 24.007 | | 5.2856 | | -0.4324 | | 1.349477 | FALSE | 0.5 | TRUE | Amp | N |
| B5 | ERCC3 | SYBR | None | 22.605 | | 3.8836 | | 0.6736 | | 0.62694 | FALSE | 0.5 | TRUE | Amp | N |
| B6 | ERCC4 | SYBR | None | 27.645 | | 8.9236 | | -0.4404 | | 1.356981 | FALSE | 0.5 | TRUE | Amp | N |
| B7 | ERCC5 | SYBR | None | 25.228 | | 6.5066 | | -0.1844 | | 1.136344 | FALSE | 0.5 | TRUE | Amp | N |
| B8 | ERCC6 | SYBR | None | 17.688 | | -1.0334 | | -0.5314 | | 1.445331 | FALSE | 0.5 | TRUE | Amp | N |
| B9 | ERCC8 | SYBR | None | 22.92 | | 4.1986 | | -0.1784 | | 1.131628 | FALSE | 0.5 | TRUE | Amp | N |
| B10 | EXO1 | SYBR | None | 21.055 | | 2.3336 | | 0.4076 | | 0.753876 | FALSE | 0.5 | TRUE | Amp | N |
| B11 | FEN1 | SYBR | None | 18.581 | | -0.1404 | | 0.5396 | | 0.687962 | FALSE | 0.5 | TRUE | Amp | N |
| B12 | LIG1 | SYBR | None | 22.063 | | 3.3416 | | -0.3744 | | 1.2963 | FALSE | 0.5 | TRUE | Amp | N |
| C1 | LIG3 | SYBR | None | 26.585 | | 7.8636 | | 0.7506 | | 0.594356 | FALSE | 0.5 | TRUE | Amp | N |
| C2 | LIG4 | SYBR | None | 26.192 | | 7.4706 | | 0.6376 | | 0.642781 | FALSE | 0.5 | TRUE | Amp | N |
| C3 | MGMT | SYBR | None | 21.443 | | 2.7216 | | -0.4424 | | 1.358863 | FALSE | 0.5 | TRUE | Amp | N |
| C4 | MLH1 | SYBR | None | 22.198 | | 3.4766 | | -0.4794 | | 1.394164 | FALSE | 0.5 | TRUE | Amp | N |
| C5 | MLH3 | SYBR | None | 25.768 | | 7.0466 | | 0.2996 | | 0.812478 | FALSE | 0.5 | TRUE | Amp | N |
| C6 | MMS19 | SYBR | None | 25.597 | | 6.8756 | | -0.5084 | | 1.422472 | FALSE | 0.5 | TRUE | Amp | N |
| C7 | MPG | SYBR | None | 21.615 | | 2.8936 | | -0.1794 | | 1.132413 | FALSE | 0.5 | TRUE | Amp | N |
| C8 | MRE11A | SYBR | None | 19.151 | | 0.4296 | | 0.4936 | | 0.710251 | FALSE | 0.5 | TRUE | Amp | N |
| C9 | MSH2 | SYBR | None | 16.908 | | -1.8134 | | -0.1824 | | 1.13477 | FALSE | 0.5 | TRUE | Amp | N |
| C10 | MSH3 | SYBR | None | 19.984 | | 1.2626 | | -0.0964 | | 1.069102 | FALSE | 0.5 | TRUE | Amp | N |
| C11 | MSH4 | SYBR | None | 20.714 | | 1.9926 | | -0.5394 | | 1.453368 | FALSE | 0.5 | TRUE | Amp | N |
| C12 | MSH5 | SYBR | None | 28.936 | | 10.2146 | | -0.1414 | | 1.102975 | FALSE | 0.5 | TRUE | Amp | N |
| D1 | MSH6 | SYBR | None | 20.729 | | 2.0076 | | -0.3944 | | 1.314396 | FALSE | 0.5 | TRUE | Amp | N |
| D2 | MUTYH | SYBR | None | 23.594 | | 4.8726 | | -0.5394 | | 1.453368 | FALSE | 0.5 | TRUE | Amp | N |
| D3 | NEIL1 | SYBR | None | 27.908 | | 9.1866 | | 0.5636 | | 0.676612 | FALSE | 0.5 | TRUE | Amp | N |
| D4 | NEIL2 | SYBR | None | 24.936 | | 6.2146 | | 0.6436 | | 0.640114 | FALSE | 0.5 | TRUE | Amp | N |
| D5 | NEIL3 | SYBR | None | 21.877 | | 3.1556 | | 0.2916 | | 0.816995 | FALSE | 0.5 | TRUE | Amp | N |
| D6 | NTHL1 | SYBR | None | 21.709 | | 2.9876 | | 0.3896 | | 0.763341 | FALSE | 0.5 | TRUE | Amp | N |
| D7 | OGG1 | SYBR | None | 22.914 | | 4.1926 | | 0.5956 | | 0.661769 | FALSE | 0.5 | TRUE | Amp | N |
| D8 | PARP1 | SYBR | None | 21.288 | | 2.5666 | | -0.1094 | | 1.078779 | FALSE | 0.5 | TRUE | Amp | N |
| D9 | PARP2 | SYBR | None | 21.726 | | 3.0046 | | 0.2916 | | 0.816995 | FALSE | 0.5 | TRUE | Amp | N |
| D10 | PARP3 | SYBR | None | 24.034 | | 5.3126 | | -0.4184 | | 1.336445 | FALSE | 0.5 | TRUE | Amp | N |
| D11 | PMS1 | SYBR | None | 18.915 | | 0.1936 | | 0.2896 | | 0.818129 | FALSE | 0.5 | TRUE | Amp | N |
| D12 | PMS2 | SYBR | None | 23.214 | | 4.4926 | | 0.6506 | | 0.637015 | FALSE | 0.5 | TRUE | Amp | N |
| E1 | PNKP | SYBR | None | 25.486 | | 6.7646 | | 0.5436 | | 0.686057 | FALSE | 0.5 | TRUE | Amp | N |
| E2 | POLB | SYBR | None | 18.656 | | -0.0654 | | 0.5796 | | 0.669149 | FALSE | 0.5 | TRUE | Amp | N |
| E3 | POLD3 | SYBR | None | 20.784 | | 2.0626 | | 0.2976 | | 0.813605 | FALSE | 0.5 | TRUE | Amp | N |
| E4 | POLL | SYBR | None | 25.781 | | 7.0596 | | 0.5426 | | 0.686533 | FALSE | 0.5 | TRUE | Amp | N |
| E5 | PRKDC | SYBR | None | 23.781 | | 5.0596 | | -0.3994 | | 1.318959 | FALSE | 0.5 | TRUE | Amp | N |
| E6 | RAD18 | SYBR | None | 18.109 | | -0.6124 | | -0.1804 | | 1.133198 | FALSE | 0.5 | TRUE | Amp | N |
| E7 | RAD21 | SYBR | None | 18.478 | | -0.2434 | | -0.3784 | | 1.299899 | FALSE | 0.5 | TRUE | Amp | N |
| E8 | RAD23A | SYBR | None | 23.53 | | 4.8086 | | 0.6756 | | 0.626072 | FALSE | 0.5 | TRUE | Amp | N |
| E9 | RAD23B | SYBR | None | 24.526 | | 5.8046 | | -0.4894 | | 1.403861 | FALSE | 0.5 | TRUE | Amp | N |
| E10 | RAD50 | SYBR | None | 20.915 | | 2.1936 | | 0.5776 | | 0.670078 | FALSE | 0.5 | TRUE | Amp | N |
| E11 | RAD51 | SYBR | None | 22.416 | | 3.6946 | | 0.6536 | | 0.635692 | FALSE | 0.5 | TRUE | Amp | N |
| E12 | RAD51B | SYBR | None | 20.806 | | 2.0846 | | 0.2626 | | 0.833584 | FALSE | 0.5 | TRUE | Amp | N |
| F1 | RAD51C | SYBR | None | 20.059 | | 1.3376 | | 0.6306 | | 0.645908 | FALSE | 0.5 | TRUE | Amp | N |
| F2 | RAD51D | SYBR | None | 24.143 | | 5.4216 | | -0.1004 | | 1.072071 | FALSE | 0.5 | TRUE | Amp | N |
| F3 | RAD52 | SYBR | None | 25.884 | | 7.1626 | | -0.4374 | | 1.354162 | FALSE | 0.5 | TRUE | Amp | N |
| F4 | RAD54L | SYBR | None | 22.654 | | 3.9326 | | -0.4614 | | 1.376877 | FALSE | 0.5 | TRUE | Amp | N |
| F5 | RFC1 | SYBR | None | 23.842 | | 5.1206 | | -0.4344 | | 1.351349 | FALSE | 0.5 | TRUE | Amp | N |
| F6 | RPA1 | SYBR | None | 18.371 | | -0.3504 | | 0.5636 | | 0.676612 | FALSE | 0.5 | TRUE | Amp | N |
| F7 | RPA3 | SYBR | None | 24.744 | | 6.0226 | | 0.6776 | | 0.625204 | FALSE | 0.5 | TRUE | Amp | N |
| F8 | SLK | SYBR | None | 20.761 | | 2.0396 | | -0.5194 | | 1.433359 | FALSE | 0.5 | TRUE | Amp | N |
| F9 | SMUG1 | SYBR | None | 26.637 | | 7.9156 | | -0.5574 | | 1.471615 | FALSE | 0.5 | TRUE | Amp | N |
| F10 | TDG | SYBR | None | 22.961 | | 4.2396 | | 0.6386 | | 0.642336 | FALSE | 0.5 | TRUE | Amp | N |
| F11 | TOP3A | SYBR | None | 26.168 | | 7.4466 | | 0.7626 | | 0.589433 | FALSE | 0.5 | TRUE | Amp | N |
| F12 | TOP3B | SYBR | None | 27.83 | | 9.1086 | | -0.1414 | | 1.102975 | FALSE | 0.5 | TRUE | Amp | N |
| G1 | TREX1 | SYBR | None | 29.123 | | 10.4016 | | 0.7676 | | 0.587394 | FALSE | 0.5 | TRUE | Amp | N |
| G2 | UNG | SYBR | None | 20.097 | | 1.3756 | | -0.1744 | | 1.128495 | FALSE | 0.5 | TRUE | Amp | N |
| G3 | XAB2 | SYBR | None | 24.767 | | 6.0456 | | 0.6516 | | 0.636574 | FALSE | 0.5 | TRUE | Amp | N |
| G4 | XPA | SYBR | None | 31.634 | | 12.9126 | | -0.1414 | | 1.102975 | FALSE | 0.5 | TRUE | Amp | N |
| G5 | XPC | SYBR | None | 26.107 | | 7.3856 | | 0.2846 | | 0.820969 | FALSE | 0.5 | TRUE | Amp | N |
| G6 | XRCC1 | SYBR | None | 20.56 | | 1.8386 | | -0.4394 | | 1.35604 | FALSE | 0.5 | TRUE | Amp | N |
| G7 | XRCC2 | SYBR | None | 21.753 | | 3.0316 | | -0.3704 | | 1.292711 | FALSE | 0.5 | TRUE | Amp | N |
| G8 | XRCC3 | SYBR | None | 23.688 | | 4.9666 | | -0.4864 | | 1.400945 | FALSE | 0.5 | TRUE | Amp | N |
| G9 | XRCC4 | SYBR | None | 25.238 | | 6.5166 | | -0.1734 | | 1.127713 | FALSE | 0.5 | TRUE | Amp | N |
| G10 | XRCC5 | SYBR | None | 17.515 | | -1.2064 | | -0.3874 | | 1.308034 | FALSE | 0.5 | TRUE | Amp | N |
| G11 | XRCC6 | SYBR | None | 20.411 | | 1.6896 | | 0.5836 | | 0.667297 | FALSE | 0.5 | TRUE | Amp | N |
| G12 | XRCC6BP1 | SYBR | None | 23.686 | | 4.9646 | | -0.5694 | | 1.483906 | FALSE | 0.5 | TRUE | Amp | N |
| H1 | ACTB | SYBR | None | 18.521 | |  | |  | |  | FALSE | 0.5 | TRUE | Amp | N |
| H2 | B2M | SYBR | None | 16.835 | |  | |  | |  | FALSE | 0.5 | TRUE | Amp | N |
| H3 | GAPDH | SYBR | None | 21.08 | |  | |  | |  | FALSE | 0.5 | TRUE | Amp | N |
| H4 | HPRT1 | SYBR | None | 21.319 | |  | |  | |  | FALSE | 0.5 | TRUE | Amp | N |
| H5 | RPLP0 | SYBR | None | 15.852 | |  | |  | |  | FALSE | 0.5 | TRUE | Amp | N |
| POI 6 |  |  |  |  | |  | |  | |  |  |  |  |  |  |
| Well Position | Gene Symbol | Reporter | Quencher | CT | | Delta Ct | | Delta Delta Ct | | 2^(-Delta Delta Ct) | Automatic Ct Threshold | Ct Threshold | Automatic Baseline | Amp Status | MTP |
| A1 | APEX1 | SYBR | None | 17.753 | | -0.5372 | | -0.4882 | | 1.402694 | FALSE | 0.5 | TRUE | Amp | N |
| A2 | APEX2 | SYBR | None | 21.608 | | 3.3178 | | -0.4172 | | 1.335333 | FALSE | 0.5 | TRUE | Amp | N |
| A3 | ATM | SYBR | None | 24.126 | | 5.8358 | | 0.6918 | | 0.619081 | FALSE | 0.5 | TRUE | Amp | N |
| A4 | ATR | SYBR | None | 23.384 | | 5.0938 | | 0.5878 | | 0.665357 | FALSE | 0.5 | TRUE | Amp | N |
| A5 | ATXN3 | SYBR | None | 20.786 | | 2.4958 | | 0.2758 | | 0.825992 | FALSE | 0.5 | TRUE | Amp | N |
| A6 | BRCA1 | SYBR | None | 21.983 | | 3.6928 | | 0.4448 | | 0.734686 | FALSE | 0.5 | TRUE | Amp | N |
| A7 | BRCA2 | SYBR | None | 18.455 | | 0.1648 | | -0.2792 | | 1.213522 | FALSE | 0.5 | TRUE | Amp | N |
| A8 | BRIP1 | SYBR | None | 23.259 | | 4.9688 | | 0.2348 | | 0.849803 | FALSE | 0.5 | TRUE | Amp | N |
| A9 | CCNH | SYBR | None | 24.517 | | 6.2268 | | -0.3222 | | 1.250236 | FALSE | 0.5 | TRUE | Amp | N |
| A10 | CCNO | SYBR | None | 22.874 | | 4.5838 | | 0.3448 | | 0.787417 | FALSE | 0.5 | TRUE | Amp | N |
| A11 | CDK7 | SYBR | None | 14.097 | | -4.1932 | | -0.0422 | | 1.029683 | FALSE | 0.5 | TRUE | Amp | N |
| A12 | DDB1 | SYBR | None | 20.211 | | 1.9208 | | -0.4362 | | 1.353036 | FALSE | 0.5 | TRUE | Amp | N |
| B1 | DDB2 | SYBR | None | 23.819 | | 5.5288 | | 0.6338 | | 0.644477 | FALSE | 0.5 | TRUE | Amp | N |
| B2 | DMC1 | SYBR | None | 28.111 | | 9.8208 | | 0.7478 | | 0.595511 | FALSE | 0.5 | TRUE | Amp | N |
| B3 | ERCC1 | SYBR | None | 22.094 | | 3.8038 | | -0.0402 | | 1.028256 | FALSE | 0.5 | TRUE | Amp | N |
| B4 | ERCC2 | SYBR | None | 24.72 | | 6.4298 | | 0.7118 | | 0.610558 | FALSE | 0.5 | TRUE | Amp | N |
| B5 | ERCC3 | SYBR | None | 21.216 | | 2.9258 | | -0.2842 | | 1.217735 | FALSE | 0.5 | TRUE | Amp | N |
| B6 | ERCC4 | SYBR | None | 27.316 | | 9.0258 | | -0.3382 | | 1.264178 | FALSE | 0.5 | TRUE | Amp | N |
| B7 | ERCC5 | SYBR | None | 25.697 | | 7.4068 | | 0.7158 | | 0.608867 | FALSE | 0.5 | TRUE | Amp | N |
| B8 | ERCC6 | SYBR | None | 17.331 | | -0.9592 | | -0.4572 | | 1.372875 | FALSE | 0.5 | TRUE | Amp | N |
| B9 | ERCC8 | SYBR | None | 22.629 | | 4.3388 | | -0.0382 | | 1.026832 | FALSE | 0.5 | TRUE | Amp | N |
| B10 | EXO1 | SYBR | None | 20.772 | | 2.4818 | | 0.5558 | | 0.68028 | FALSE | 0.5 | TRUE | Amp | N |
| B11 | FEN1 | SYBR | None | 18.143 | | -0.1472 | | 0.5328 | | 0.691212 | FALSE | 0.5 | TRUE | Amp | N |
| B12 | LIG1 | SYBR | None | 21.676 | | 3.3858 | | -0.3302 | | 1.257188 | FALSE | 0.5 | TRUE | Amp | N |
| C1 | LIG3 | SYBR | None | 25.029 | | 6.7388 | | -0.3742 | | 1.296121 | FALSE | 0.5 | TRUE | Amp | N |
| C2 | LIG4 | SYBR | None | 25.38 | | 7.0898 | | 0.2568 | | 0.836942 | FALSE | 0.5 | TRUE | Amp | N |
| C3 | MGMT | SYBR | None | 21.437 | | 3.1468 | | -0.0172 | | 1.011993 | FALSE | 0.5 | TRUE | Amp | N |
| C4 | MLH1 | SYBR | None | 22.476 | | 4.1858 | | 0.2298 | | 0.852753 | FALSE | 0.5 | TRUE | Amp | N |
| C5 | MLH3 | SYBR | None | 25.285 | | 6.9948 | | 0.2478 | | 0.84218 | FALSE | 0.5 | TRUE | Amp | N |
| C6 | MMS19 | SYBR | None | 25.631 | | 7.3408 | | -0.0432 | | 1.030397 | FALSE | 0.5 | TRUE | Amp | N |
| C7 | MPG | SYBR | None | 21.634 | | 3.3438 | | 0.2708 | | 0.82886 | FALSE | 0.5 | TRUE | Amp | N |
| C8 | MRE11A | SYBR | None | 18.789 | | 0.4988 | | 0.5628 | | 0.676987 | FALSE | 0.5 | TRUE | Amp | N |
| C9 | MSH2 | SYBR | None | 16.372 | | -1.9182 | | -0.2872 | | 1.22027 | FALSE | 0.5 | TRUE | Amp | N |
| C10 | MSH3 | SYBR | None | 20.078 | | 1.7878 | | 0.4288 | | 0.742879 | FALSE | 0.5 | TRUE | Amp | N |
| C11 | MSH4 | SYBR | None | 20.421 | | 2.1308 | | -0.4012 | | 1.320606 | FALSE | 0.5 | TRUE | Amp | N |
| C12 | MSH5 | SYBR | None | 29.013 | | 10.7228 | | 0.3668 | | 0.775501 | FALSE | 0.5 | TRUE | Amp | N |
| D1 | MSH6 | SYBR | None | 20.207 | | 1.9168 | | -0.4852 | | 1.39978 | FALSE | 0.5 | TRUE | Amp | N |
| D2 | MUTYH | SYBR | None | 23.354 | | 5.0638 | | -0.3482 | | 1.272971 | FALSE | 0.5 | TRUE | Amp | N |
| D3 | NEIL1 | SYBR | None | 27.194 | | 8.9038 | | 0.2808 | | 0.823134 | FALSE | 0.5 | TRUE | Amp | N |
| D4 | NEIL2 | SYBR | None | 23.538 | | 5.2478 | | -0.3232 | | 1.251103 | FALSE | 0.5 | TRUE | Amp | N |
| D5 | NEIL3 | SYBR | None | 20.893 | | 2.6028 | | -0.2612 | | 1.198475 | FALSE | 0.5 | TRUE | Amp | N |
| D6 | NTHL1 | SYBR | None | 21.448 | | 3.1578 | | 0.5598 | | 0.678396 | FALSE | 0.5 | TRUE | Amp | N |
| D7 | OGG1 | SYBR | None | 21.575 | | 3.2848 | | -0.3122 | | 1.2416 | FALSE | 0.5 | TRUE | Amp | N |
| D8 | PARP1 | SYBR | None | 21.396 | | 3.1058 | | 0.4298 | | 0.742365 | FALSE | 0.5 | TRUE | Amp | N |
| D9 | PARP2 | SYBR | None | 21.245 | | 2.9548 | | 0.2418 | | 0.84569 | FALSE | 0.5 | TRUE | Amp | N |
| D10 | PARP3 | SYBR | None | 24.636 | | 6.3458 | | 0.6148 | | 0.65302 | FALSE | 0.5 | TRUE | Amp | N |
| D11 | PMS1 | SYBR | None | 18.425 | | 0.1348 | | 0.2308 | | 0.852162 | FALSE | 0.5 | TRUE | Amp | N |
| D12 | PMS2 | SYBR | None | 21.776 | | 3.4858 | | -0.3562 | | 1.28005 | FALSE | 0.5 | TRUE | Amp | N |
| E1 | PNKP | SYBR | None | 24.882 | | 6.5918 | | 0.3708 | | 0.773354 | FALSE | 0.5 | TRUE | Amp | N |
| E2 | POLB | SYBR | None | 17.897 | | -0.3932 | | 0.2518 | | 0.839848 | FALSE | 0.5 | TRUE | Amp | N |
| E3 | POLD3 | SYBR | None | 20.329 | | 2.0388 | | 0.2738 | | 0.827138 | FALSE | 0.5 | TRUE | Amp | N |
| E4 | POLL | SYBR | None | 24.414 | | 6.1238 | | -0.3932 | | 1.313303 | FALSE | 0.5 | TRUE | Amp | N |
| E5 | PRKDC | SYBR | None | 23.38 | | 5.0898 | | -0.3692 | | 1.291636 | FALSE | 0.5 | TRUE | Amp | N |
| E6 | RAD18 | SYBR | None | 17.837 | | -0.4532 | | -0.0212 | | 1.014803 | FALSE | 0.5 | TRUE | Amp | N |
| E7 | RAD21 | SYBR | None | 18.958 | | 0.6678 | | 0.5328 | | 0.691212 | FALSE | 0.5 | TRUE | Amp | N |
| E8 | RAD23A | SYBR | None | 22.753 | | 4.4628 | | 0.3298 | | 0.795647 | FALSE | 0.5 | TRUE | Amp | N |
| E9 | RAD23B | SYBR | None | 25.278 | | 6.9878 | | 0.6938 | | 0.618223 | FALSE | 0.5 | TRUE | Amp | N |
| E10 | RAD50 | SYBR | None | 20.14 | | 1.8498 | | 0.2338 | | 0.850392 | FALSE | 0.5 | TRUE | Amp | N |
| E11 | RAD51 | SYBR | None | 21.914 | | 3.6238 | | 0.5828 | | 0.667667 | FALSE | 0.5 | TRUE | Amp | N |
| E12 | RAD51B | SYBR | None | 20.736 | | 2.4458 | | 0.6238 | | 0.648959 | FALSE | 0.5 | TRUE | Amp | N |
| F1 | RAD51C | SYBR | None | 19.498 | | 1.2078 | | 0.5008 | | 0.706715 | FALSE | 0.5 | TRUE | Amp | N |
| F2 | RAD51D | SYBR | None | 23.408 | | 5.1178 | | -0.4042 | | 1.323355 | FALSE | 0.5 | TRUE | Amp | N |
| F3 | RAD52 | SYBR | None | 26.57 | | 8.2798 | | 0.6798 | | 0.624252 | FALSE | 0.5 | TRUE | Amp | N |
| F4 | RAD54L | SYBR | None | 22.353 | | 4.0628 | | -0.3312 | | 1.258059 | FALSE | 0.5 | TRUE | Amp | N |
| F5 | RFC1 | SYBR | None | 23.55 | | 5.2598 | | -0.2952 | | 1.227055 | FALSE | 0.5 | TRUE | Amp | N |
| F6 | RPA1 | SYBR | None | 17.642 | | -0.6482 | | 0.2658 | | 0.831737 | FALSE | 0.5 | TRUE | Amp | N |
| F7 | RPA3 | SYBR | None | 23.623 | | 5.3328 | | -0.0122 | | 1.008492 | FALSE | 0.5 | TRUE | Amp | N |
| F8 | SLK | SYBR | None | 21.271 | | 2.9808 | | 0.4218 | | 0.746493 | FALSE | 0.5 | TRUE | Amp | N |
| F9 | SMUG1 | SYBR | None | 27.327 | | 9.0368 | | 0.5638 | | 0.676518 | FALSE | 0.5 | TRUE | Amp | N |
| F10 | TDG | SYBR | None | 22.486 | | 4.1958 | | 0.5948 | | 0.662136 | FALSE | 0.5 | TRUE | Amp | N |
| F11 | TOP3A | SYBR | None | 25.675 | | 7.3848 | | 0.7008 | | 0.615231 | FALSE | 0.5 | TRUE | Amp | N |
| F12 | TOP3B | SYBR | None | 26.366 | | 8.0758 | | -1.1742 | | 2.256677 | FALSE | 0.5 | TRUE | Amp | N |
| G1 | TREX1 | SYBR | None | 27.454 | | 9.1638 | | -0.4702 | | 1.385301 | FALSE | 0.5 | TRUE | Amp | N |
| G2 | UNG | SYBR | None | 19.536 | | 1.2458 | | -0.3042 | | 1.234734 | FALSE | 0.5 | TRUE | Amp | N |
| G3 | XAB2 | SYBR | None | 23.25 | | 4.9598 | | -0.4342 | | 1.351161 | FALSE | 0.5 | TRUE | Amp | N |
| G4 | XPA | SYBR | None | 32.178 | | 13.8878 | | 0.8338 | | 0.56105 | FALSE | 0.5 | TRUE | Amp | N |
| G5 | XPC | SYBR | None | 25.639 | | 7.3488 | | 0.2478 | | 0.84218 | FALSE | 0.5 | TRUE | Amp | N |
| G6 | XRCC1 | SYBR | None | 20.824 | | 2.5338 | | 0.2558 | | 0.837523 | FALSE | 0.5 | TRUE | Amp | N |
| G7 | XRCC2 | SYBR | None | 21.327 | | 3.0368 | | -0.3652 | | 1.28806 | FALSE | 0.5 | TRUE | Amp | N |
| G8 | XRCC3 | SYBR | None | 23.391 | | 5.1008 | | -0.3522 | | 1.276506 | FALSE | 0.5 | TRUE | Amp | N |
| G9 | XRCC4 | SYBR | None | 25.253 | | 6.9628 | | 0.2728 | | 0.827712 | FALSE | 0.5 | TRUE | Amp | N |
| G10 | XRCC5 | SYBR | None | 17.253 | | -1.0372 | | -0.2182 | | 1.163281 | FALSE | 0.5 | TRUE | Amp | N |
| G11 | XRCC6 | SYBR | None | 19.97 | | 1.6798 | | 0.5738 | | 0.671845 | FALSE | 0.5 | TRUE | Amp | N |
| G12 | XRCC6BP1 | SYBR | None | 23.381 | | 5.0908 | | -0.4432 | | 1.359617 | FALSE | 0.5 | TRUE | Amp | N |
| H1 | ACTB | SYBR | None | 18.192 | |  | |  | |  | FALSE | 0.5 | TRUE | Amp | N |
| H2 | B2M | SYBR | None | 16.539 | |  | |  | |  | FALSE | 0.5 | TRUE | Amp | N |
| H3 | GAPDH | SYBR | None | 20.774 | |  | |  | |  | FALSE | 0.5 | TRUE | Amp | N |
| H4 | HPRT1 | SYBR | None | 20.509 | |  | |  | |  | FALSE | 0.5 | TRUE | Amp | N |
| H5 | RPLP0 | SYBR | None | 15.437 | |  | |  | |  | FALSE | 0.5 | TRUE | Amp | N |
| POI 7 |  |  |  |  | |  | |  | |  |  |  |  |  |  |
| Well Position | Gene Symbol | Reporter | Quencher | CT | | Delta Ct | | Delta Delta Ct | | 2^(-Delta Delta Ct) | Automatic Ct Threshold | Ct Threshold | Automatic Baseline | Amp Status | MTP |
| A1 | APEX1 | SYBR | None | 18.592 | | 0.3658 | | 0.4148 | | 0.750123 | FALSE | 0.5 | TRUE | Amp | N |
| A2 | APEX2 | SYBR | None | 22.258 | | 4.0318 | | 0.2968 | | 0.814056 | FALSE | 0.5 | TRUE | Amp | N |
| A3 | ATM | SYBR | None | 23.045 | | 4.8188 | | -0.3252 | | 1.252838 | FALSE | 0.5 | TRUE | Amp | N |
| A4 | ATR | SYBR | None | 23.37 | | 5.1438 | | 0.6378 | | 0.642692 | FALSE | 0.5 | TRUE | Amp | N |
| A5 | ATXN3 | SYBR | None | 20.092 | | 1.8658 | | -0.3542 | | 1.278277 | FALSE | 0.5 | TRUE | Amp | N |
| A6 | BRCA1 | SYBR | None | 21.43 | | 3.2038 | | -0.0442 | | 1.031111 | FALSE | 0.5 | TRUE | Amp | N |
| A7 | BRCA2 | SYBR | None | 18.973 | | 0.7468 | | 0.3028 | | 0.810677 | FALSE | 0.5 | TRUE | Amp | N |
| A8 | BRIP1 | SYBR | None | 22.937 | | 4.7108 | | -0.0232 | | 1.016211 | FALSE | 0.5 | TRUE | Amp | N |
| A9 | CCNH | SYBR | None | 25.015 | | 6.7888 | | 0.2398 | | 0.846863 | FALSE | 0.5 | TRUE | Amp | N |
| A10 | CCNO | SYBR | None | 22.453 | | 4.2268 | | -0.0122 | | 1.008492 | FALSE | 0.5 | TRUE | Amp | N |
| A11 | CDK7 | SYBR | None | 14.485 | | -3.7412 | | 0.4098 | | 0.752728 | FALSE | 0.5 | TRUE | Amp | N |
| A12 | DDB1 | SYBR | None | 21.096 | | 2.8698 | | 0.5128 | | 0.700861 | FALSE | 0.5 | TRUE | Amp | N |
| B1 | DDB2 | SYBR | None | 23.78 | | 5.5538 | | 0.6588 | | 0.633405 | FALSE | 0.5 | TRUE | Amp | N |
| B2 | DMC1 | SYBR | None | 26.99 | | 8.7638 | | -0.3092 | | 1.23902 | FALSE | 0.5 | TRUE | Amp | N |
| B3 | ERCC1 | SYBR | None | 22.327 | | 4.1008 | | 0.2568 | | 0.836942 | FALSE | 0.5 | TRUE | Amp | N |
| B4 | ERCC2 | SYBR | None | 24.198 | | 5.9718 | | 0.2538 | | 0.838684 | FALSE | 0.5 | TRUE | Amp | N |
| B5 | ERCC3 | SYBR | None | 21.393 | | 3.1668 | | -0.0432 | | 1.030397 | FALSE | 0.5 | TRUE | Amp | N |
| B6 | ERCC4 | SYBR | None | 28.195 | | 9.9688 | | 0.6048 | | 0.657563 | FALSE | 0.5 | TRUE | Amp | N |
| B7 | ERCC5 | SYBR | None | 25.173 | | 6.9468 | | 0.2558 | | 0.837523 | FALSE | 0.5 | TRUE | Amp | N |
| B8 | ERCC6 | SYBR | None | 17.931 | | -0.2952 | | 0.2068 | | 0.866457 | FALSE | 0.5 | TRUE | Amp | N |
| B9 | ERCC8 | SYBR | None | 22.584 | | 4.3578 | | -0.0192 | | 1.013397 | FALSE | 0.5 | TRUE | Amp | N |
| B10 | EXO1 | SYBR | None | 20.658 | | 2.4318 | | 0.5058 | | 0.70427 | FALSE | 0.5 | TRUE | Amp | N |
| B11 | FEN1 | SYBR | None | 17.782 | | -0.4442 | | 0.2358 | | 0.849214 | FALSE | 0.5 | TRUE | Amp | N |
| B12 | LIG1 | SYBR | None | 22.209 | | 3.9828 | | 0.2668 | | 0.831161 | FALSE | 0.5 | TRUE | Amp | N |
| C1 | LIG3 | SYBR | None | 25.584 | | 7.3578 | | 0.2448 | | 0.843933 | FALSE | 0.5 | TRUE | Amp | N |
| C2 | LIG4 | SYBR | None | 25.039 | | 6.8128 | | -0.0202 | | 1.0141 | FALSE | 0.5 | TRUE | Amp | N |
| C3 | MGMT | SYBR | None | 21.37 | | 3.1438 | | -0.0202 | | 1.0141 | FALSE | 0.5 | TRUE | Amp | N |
| C4 | MLH1 | SYBR | None | 22.121 | | 3.8948 | | -0.0612 | | 1.043333 | FALSE | 0.5 | TRUE | Amp | N |
| C5 | MLH3 | SYBR | None | 24.552 | | 6.3258 | | -0.4212 | | 1.339041 | FALSE | 0.5 | TRUE | Amp | N |
| C6 | MMS19 | SYBR | None | 25.161 | | 6.9348 | | -0.4492 | | 1.365283 | FALSE | 0.5 | TRUE | Amp | N |
| C7 | MPG | SYBR | None | 21.864 | | 3.6378 | | 0.5648 | | 0.676049 | FALSE | 0.5 | TRUE | Amp | N |
| C8 | MRE11A | SYBR | None | 18.376 | | 0.1498 | | 0.2138 | | 0.862263 | FALSE | 0.5 | TRUE | Amp | N |
| C9 | MSH2 | SYBR | None | 16.32 | | -1.9062 | | -0.2752 | | 1.210162 | FALSE | 0.5 | TRUE | Amp | N |
| C10 | MSH3 | SYBR | None | 19.148 | | 0.9218 | | -0.4372 | | 1.353974 | FALSE | 0.5 | TRUE | Amp | N |
| C11 | MSH4 | SYBR | None | 20.39 | | 2.1638 | | -0.3682 | | 1.290741 | FALSE | 0.5 | TRUE | Amp | N |
| C12 | MSH5 | SYBR | None | 28.892 | | 10.6658 | | 0.3098 | | 0.806754 | FALSE | 0.5 | TRUE | Amp | N |
| D1 | MSH6 | SYBR | None | 20.336 | | 2.1098 | | -0.2922 | | 1.224506 | FALSE | 0.5 | TRUE | Amp | N |
| D2 | MUTYH | SYBR | None | 23.631 | | 5.4048 | | -0.0072 | | 1.005003 | FALSE | 0.5 | TRUE | Amp | N |
| D3 | NEIL1 | SYBR | None | 26.772 | | 8.5458 | | -0.0772 | | 1.054969 | FALSE | 0.5 | TRUE | Amp | N |
| D4 | NEIL2 | SYBR | None | 24.286 | | 6.0598 | | 0.4888 | | 0.712618 | FALSE | 0.5 | TRUE | Amp | N |
| D5 | NEIL3 | SYBR | None | 21.307 | | 3.0808 | | 0.2168 | | 0.860472 | FALSE | 0.5 | TRUE | Amp | N |
| D6 | NTHL1 | SYBR | None | 21.028 | | 2.8018 | | 0.2038 | | 0.868261 | FALSE | 0.5 | TRUE | Amp | N |
| D7 | OGG1 | SYBR | None | 22.298 | | 4.0718 | | 0.4748 | | 0.719567 | FALSE | 0.5 | TRUE | Amp | N |
| D8 | PARP1 | SYBR | None | 21.413 | | 3.1868 | | 0.5108 | | 0.701833 | FALSE | 0.5 | TRUE | Amp | N |
| D9 | PARP2 | SYBR | None | 21.194 | | 2.9678 | | 0.2548 | | 0.838103 | FALSE | 0.5 | TRUE | Amp | N |
| D10 | PARP3 | SYBR | None | 23.591 | | 5.3648 | | -0.3662 | | 1.288953 | FALSE | 0.5 | TRUE | Amp | N |
| D11 | PMS1 | SYBR | None | 18.36 | | 0.1338 | | 0.2298 | | 0.852753 | FALSE | 0.5 | TRUE | Amp | N |
| D12 | PMS2 | SYBR | None | 22.597 | | 4.3708 | | 0.5288 | | 0.693131 | FALSE | 0.5 | TRUE | Amp | N |
| E1 | PNKP | SYBR | None | 25.069 | | 6.8428 | | 0.6218 | | 0.64986 | FALSE | 0.5 | TRUE | Amp | N |
| E2 | POLB | SYBR | None | 16.672 | | -1.5542 | | -0.9092 | | 1.878004 | FALSE | 0.5 | TRUE | Amp | N |
| E3 | POLD3 | SYBR | None | 19.695 | | 1.4688 | | -0.2962 | | 1.227906 | FALSE | 0.5 | TRUE | Amp | N |
| E4 | POLL | SYBR | None | 24.31 | | 6.0838 | | -0.4332 | | 1.350225 | FALSE | 0.5 | TRUE | Amp | N |
| E5 | PRKDC | SYBR | None | 23.244 | | 5.0178 | | -0.4412 | | 1.357733 | FALSE | 0.5 | TRUE | Amp | N |
| E6 | RAD18 | SYBR | None | 17.749 | | -0.4772 | | -0.0452 | | 1.031826 | FALSE | 0.5 | TRUE | Amp | N |
| E7 | RAD21 | SYBR | None | 18.583 | | 0.3568 | | 0.2218 | | 0.857495 | FALSE | 0.5 | TRUE | Amp | N |
| E8 | RAD23A | SYBR | None | 22.875 | | 4.6488 | | 0.5158 | | 0.699405 | FALSE | 0.5 | TRUE | Amp | N |
| E9 | RAD23B | SYBR | None | 24.447 | | 6.2208 | | -0.0732 | | 1.052048 | FALSE | 0.5 | TRUE | Amp | N |
| E10 | RAD50 | SYBR | None | 20.041 | | 1.8148 | | 0.1988 | | 0.871275 | FALSE | 0.5 | TRUE | Amp | N |
| E11 | RAD51 | SYBR | None | 21.264 | | 3.0378 | | -0.0032 | | 1.002221 | FALSE | 0.5 | TRUE | Amp | N |
| E12 | RAD51B | SYBR | None | 20.638 | | 2.4118 | | 0.5898 | | 0.664435 | FALSE | 0.5 | TRUE | Amp | N |
| F1 | RAD51C | SYBR | None | 19.131 | | 0.9048 | | 0.1978 | | 0.871879 | FALSE | 0.5 | TRUE | Amp | N |
| F2 | RAD51D | SYBR | None | 23.204 | | 4.9778 | | -0.5442 | | 1.458212 | FALSE | 0.5 | TRUE | Amp | N |
| F3 | RAD52 | SYBR | None | 25.464 | | 7.2378 | | -0.3622 | | 1.285385 | FALSE | 0.5 | TRUE | Amp | N |
| F4 | RAD54L | SYBR | None | 22.285 | | 4.0588 | | -0.3352 | | 1.261552 | FALSE | 0.5 | TRUE | Amp | N |
| F5 | RFC1 | SYBR | None | 24.307 | | 6.0808 | | 0.5258 | | 0.694574 | FALSE | 0.5 | TRUE | Amp | N |
| F6 | RPA1 | SYBR | None | 17.732 | | -0.4942 | | 0.4198 | | 0.747528 | FALSE | 0.5 | TRUE | Amp | N |
| F7 | RPA3 | SYBR | None | 23.529 | | 5.3028 | | -0.0422 | | 1.029683 | FALSE | 0.5 | TRUE | Amp | N |
| F8 | SLK | SYBR | None | 21.26 | | 3.0338 | | 0.4748 | | 0.719567 | FALSE | 0.5 | TRUE | Amp | N |
| F9 | SMUG1 | SYBR | None | 27.267 | | 9.0408 | | 0.5678 | | 0.674645 | FALSE | 0.5 | TRUE | Amp | N |
| F10 | TDG | SYBR | None | 21.445 | | 3.2188 | | -0.3822 | | 1.303328 | FALSE | 0.5 | TRUE | Amp | N |
| F11 | TOP3A | SYBR | None | 25.126 | | 6.8998 | | 0.2158 | | 0.861069 | FALSE | 0.5 | TRUE | Amp | N |
| F12 | TOP3B | SYBR | None | 27.448 | | 9.2218 | | -0.0282 | | 1.019739 | FALSE | 0.5 | TRUE | Amp | N |
| G1 | TREX1 | SYBR | None | 27.464 | | 9.2378 | | -0.3962 | | 1.316037 | FALSE | 0.5 | TRUE | Amp | N |
| G2 | UNG | SYBR | None | 20.229 | | 2.0028 | | 0.4528 | | 0.730623 | FALSE | 0.5 | TRUE | Amp | N |
| G3 | XAB2 | SYBR | None | 24.206 | | 5.9798 | | 0.5858 | | 0.66628 | FALSE | 0.5 | TRUE | Amp | N |
| G4 | XPA | SYBR | None | 31.553 | | 13.3268 | | 0.2728 | | 0.827712 | FALSE | 0.5 | TRUE | Amp | N |
| G5 | XPC | SYBR | None | 25.537 | | 7.3108 | | 0.2098 | | 0.864657 | FALSE | 0.5 | TRUE | Amp | N |
| G6 | XRCC1 | SYBR | None | 20.724 | | 2.4978 | | 0.2198 | | 0.858684 | FALSE | 0.5 | TRUE | Amp | N |
| G7 | XRCC2 | SYBR | None | 22.194 | | 3.9678 | | 0.5658 | | 0.675581 | FALSE | 0.5 | TRUE | Amp | N |
| G8 | XRCC3 | SYBR | None | 23.305 | | 5.0788 | | -0.3742 | | 1.296121 | FALSE | 0.5 | TRUE | Amp | N |
| G9 | XRCC4 | SYBR | None | 24.557 | | 6.3308 | | -0.3592 | | 1.282714 | FALSE | 0.5 | TRUE | Amp | N |
| G10 | XRCC5 | SYBR | None | 17.107 | | -1.1192 | | -0.3002 | | 1.231315 | FALSE | 0.5 | TRUE | Amp | N |
| G11 | XRCC6 | SYBR | None | 19.747 | | 1.5208 | | 0.4148 | | 0.750123 | FALSE | 0.5 | TRUE | Amp | N |
| G12 | XRCC6BP1 | SYBR | None | 23.387 | | 5.1608 | | -0.3732 | | 1.295223 | FALSE | 0.5 | TRUE | Amp | N |
| H1 | ACTB | SYBR | None | 18.265 | |  | |  | |  | FALSE | 0.5 | TRUE | Amp | N |
| H2 | B2M | SYBR | None | 16.547 | |  | |  | |  | FALSE | 0.5 | TRUE | Amp | N |
| H3 | GAPDH | SYBR | None | 20.663 | |  | |  | |  | FALSE | 0.5 | TRUE | Amp | N |
| H4 | HPRT1 | SYBR | None | 20.532 | |  | |  | |  | FALSE | 0.5 | TRUE | Amp | N |
| H5 | RPLP0 | SYBR | None | 15.124 | |  | |  | |  | FALSE | 0.5 | TRUE | Amp | N |
| POI 8 |  |  |  |  | |  | |  | |  |  |  |  |  |  |
| Well Position | Gene Symbol | Reporter | Quencher | CT | | Delta Ct | | Delta Delta Ct | | 2^(-Delta Delta Ct) | Automatic Ct Threshold | Ct Threshold | Automatic Baseline | Amp Status | MTP |
| A1 | APEX1 | SYBR | None | 18.793 | | 0.0648 | | 0.1138 | | 0.924151 | FALSE | 0.5 | TRUE | Amp | N |
| A2 | APEX2 | SYBR | None | 22.654 | | 3.9258 | | 0.1908 | | 0.87612 | FALSE | 0.5 | TRUE | Amp | N |
| A3 | ATM | SYBR | None | 23.728 | | 4.9998 | | -0.1442 | | 1.105118 | FALSE | 0.5 | TRUE | Amp | N |
| A4 | ATR | SYBR | None | 23.132 | | 4.4038 | | -0.1022 | | 1.073409 | FALSE | 0.5 | TRUE | Amp | N |
| A5 | ATXN3 | SYBR | None | 21.023 | | 2.2948 | | 0.0748 | | 0.949474 | FALSE | 0.5 | TRUE | Amp | N |
| A6 | BRCA1 | SYBR | None | 22.12 | | 3.3918 | | 0.1438 | | 0.905132 | FALSE | 0.5 | TRUE | Amp | N |
| A7 | BRCA2 | SYBR | None | 18.898 | | 0.1698 | | -0.2742 | | 1.209323 | FALSE | 0.5 | TRUE | Amp | N |
| A8 | BRIP1 | SYBR | None | 23.659 | | 4.9308 | | 0.1968 | | 0.872484 | FALSE | 0.5 | TRUE | Amp | N |
| A9 | CCNH | SYBR | None | 25.122 | | 6.3938 | | -0.1552 | | 1.113576 | FALSE | 0.5 | TRUE | Amp | N |
| A10 | CCNO | SYBR | None | 22.697 | | 3.9688 | | -0.2702 | | 1.205975 | FALSE | 0.5 | TRUE | Amp | N |
| A11 | CDK7 | SYBR | None | 14.812 | | -3.9162 | | 0.2348 | | 0.849803 | FALSE | 0.5 | TRUE | Amp | N |
| A12 | DDB1 | SYBR | None | 20.98 | | 2.2518 | | -0.1052 | | 1.075643 | FALSE | 0.5 | TRUE | Amp | N |
| B1 | DDB2 | SYBR | None | 23.695 | | 4.9668 | | 0.0718 | | 0.95145 | FALSE | 0.5 | TRUE | Amp | N |
| B2 | DMC1 | SYBR | None | 27.546 | | 8.8178 | | -0.2552 | | 1.193501 | FALSE | 0.5 | TRUE | Amp | N |
| B3 | ERCC1 | SYBR | None | 22.653 | | 3.9248 | | 0.0808 | | 0.945533 | FALSE | 0.5 | TRUE | Amp | N |
| B4 | ERCC2 | SYBR | None | 24.319 | | 5.5908 | | -0.1272 | | 1.092172 | FALSE | 0.5 | TRUE | Amp | N |
| B5 | ERCC3 | SYBR | None | 21.582 | | 2.8538 | | -0.3562 | | 1.28005 | FALSE | 0.5 | TRUE | Amp | N |
| B6 | ERCC4 | SYBR | None | 28.211 | | 9.4828 | | 0.1188 | | 0.920953 | FALSE | 0.5 | TRUE | Amp | N |
| B7 | ERCC5 | SYBR | None | 25.557 | | 6.8288 | | 0.1378 | | 0.908904 | FALSE | 0.5 | TRUE | Amp | N |
| B8 | ERCC6 | SYBR | None | 18.278 | | -0.4502 | | 0.0518 | | 0.964732 | FALSE | 0.5 | TRUE | Amp | N |
| B9 | ERCC8 | SYBR | None | 23.16 | | 4.4318 | | 0.0548 | | 0.962728 | FALSE | 0.5 | TRUE | Amp | N |
| B10 | EXO1 | SYBR | None | 20.665 | | 1.9368 | | 0.0108 | | 0.992542 | FALSE | 0.5 | TRUE | Amp | N |
| B11 | FEN1 | SYBR | None | 17.836 | | -0.8922 | | -0.2122 | | 1.158453 | FALSE | 0.5 | TRUE | Amp | N |
| B12 | LIG1 | SYBR | None | 22.554 | | 3.8258 | | 0.1098 | | 0.926717 | FALSE | 0.5 | TRUE | Amp | N |
| C1 | LIG3 | SYBR | None | 26.003 | | 7.2748 | | 0.1618 | | 0.893909 | FALSE | 0.5 | TRUE | Amp | N |
| C2 | LIG4 | SYBR | None | 25.66 | | 6.9318 | | 0.0988 | | 0.933809 | FALSE | 0.5 | TRUE | Amp | N |
| C3 | MGMT | SYBR | None | 21.676 | | 2.9478 | | -0.2162 | | 1.16167 | FALSE | 0.5 | TRUE | Amp | N |
| C4 | MLH1 | SYBR | None | 22.814 | | 4.0858 | | 0.1298 | | 0.913958 | FALSE | 0.5 | TRUE | Amp | N |
| C5 | MLH3 | SYBR | None | 25.612 | | 6.8838 | | 0.1368 | | 0.909534 | FALSE | 0.5 | TRUE | Amp | N |
| C6 | MMS19 | SYBR | None | 25.928 | | 7.1998 | | -0.1842 | | 1.136187 | FALSE | 0.5 | TRUE | Amp | N |
| C7 | MPG | SYBR | None | 21.731 | | 3.0028 | | -0.0702 | | 1.049862 | FALSE | 0.5 | TRUE | Amp | N |
| C8 | MRE11A | SYBR | None | 18.658 | | -0.0702 | | -0.0062 | | 1.004307 | FALSE | 0.5 | TRUE | Amp | N |
| C9 | MSH2 | SYBR | None | 16.794 | | -1.9342 | | -0.3032 | | 1.233878 | FALSE | 0.5 | TRUE | Amp | N |
| C10 | MSH3 | SYBR | None | 20.152 | | 1.4238 | | 0.0648 | | 0.956078 | FALSE | 0.5 | TRUE | Amp | N |
| C11 | MSH4 | SYBR | None | 21.045 | | 2.3168 | | -0.2152 | | 1.160865 | FALSE | 0.5 | TRUE | Amp | N |
| C12 | MSH5 | SYBR | None | 28.92 | | 10.1918 | | -0.1642 | | 1.120545 | FALSE | 0.5 | TRUE | Amp | N |
| D1 | MSH6 | SYBR | None | 20.918 | | 2.1898 | | -0.2122 | | 1.158453 | FALSE | 0.5 | TRUE | Amp | N |
| D2 | MUTYH | SYBR | None | 23.869 | | 5.1408 | | -0.2712 | | 1.206811 | FALSE | 0.5 | TRUE | Amp | N |
| D3 | NEIL1 | SYBR | None | 27.011 | | 8.2828 | | -0.3402 | | 1.265932 | FALSE | 0.5 | TRUE | Amp | N |
| D4 | NEIL2 | SYBR | None | 24.182 | | 5.4538 | | -0.1172 | | 1.084628 | FALSE | 0.5 | TRUE | Amp | N |
| D5 | NEIL3 | SYBR | None | 21.455 | | 2.7268 | | -0.1372 | | 1.099769 | FALSE | 0.5 | TRUE | Amp | N |
| D6 | NTHL1 | SYBR | None | 21.23 | | 2.5018 | | -0.0962 | | 1.068954 | FALSE | 0.5 | TRUE | Amp | N |
| D7 | OGG1 | SYBR | None | 22.092 | | 3.3638 | | -0.2332 | | 1.175439 | FALSE | 0.5 | TRUE | Amp | N |
| D8 | PARP1 | SYBR | None | 22.55 | | 3.8218 | | 1.1458 | | 0.451939 | FALSE | 0.5 | TRUE | Amp | N |
| D9 | PARP2 | SYBR | None | 21.557 | | 2.8288 | | 0.1158 | | 0.92287 | FALSE | 0.5 | TRUE | Amp | N |
| D10 | PARP3 | SYBR | None | 24.301 | | 5.5728 | | -0.1582 | | 1.115894 | FALSE | 0.5 | TRUE | Amp | N |
| D11 | PMS1 | SYBR | None | 18.656 | | -0.0722 | | 0.0238 | | 0.983638 | FALSE | 0.5 | TRUE | Amp | N |
| D12 | PMS2 | SYBR | None | 22.548 | | 3.8198 | | -0.0222 | | 1.015507 | FALSE | 0.5 | TRUE | Amp | N |
| E1 | PNKP | SYBR | None | 24.981 | | 6.2528 | | 0.0318 | | 0.978199 | FALSE | 0.5 | TRUE | Amp | N |
| E2 | POLB | SYBR | None | 17.904 | | -0.8242 | | -0.1792 | | 1.132256 | FALSE | 0.5 | TRUE | Amp | N |
| E3 | POLD3 | SYBR | None | 20.69 | | 1.9618 | | 0.1968 | | 0.872484 | FALSE | 0.5 | TRUE | Amp | N |
| E4 | POLL | SYBR | None | 25.103 | | 6.3748 | | -0.1422 | | 1.103587 | FALSE | 0.5 | TRUE | Amp | N |
| E5 | PRKDC | SYBR | None | 24.092 | | 5.3638 | | -0.0952 | | 1.068213 | FALSE | 0.5 | TRUE | Amp | N |
| E6 | RAD18 | SYBR | None | 18.446 | | -0.2822 | | 0.1498 | | 0.901375 | FALSE | 0.5 | TRUE | Amp | N |
| E7 | RAD21 | SYBR | None | 18.575 | | -0.1532 | | -0.2882 | | 1.221116 | FALSE | 0.5 | TRUE | Amp | N |
| E8 | RAD23A | SYBR | None | 22.668 | | 3.9398 | | -0.1932 | | 1.143297 | FALSE | 0.5 | TRUE | Amp | N |
| E9 | RAD23B | SYBR | None | 25.177 | | 6.4488 | | 0.1548 | | 0.898257 | FALSE | 0.5 | TRUE | Amp | N |
| E10 | RAD50 | SYBR | None | 20.53 | | 1.8018 | | 0.1858 | | 0.879161 | FALSE | 0.5 | TRUE | Amp | N |
| E11 | RAD51 | SYBR | None | 21.543 | | 2.8148 | | -0.2262 | | 1.16975 | FALSE | 0.5 | TRUE | Amp | N |
| E12 | RAD51B | SYBR | None | 20.664 | | 1.9358 | | 0.1138 | | 0.924151 | FALSE | 0.5 | TRUE | Amp | N |
| F1 | RAD51C | SYBR | None | 19.66 | | 0.9318 | | 0.2248 | | 0.855714 | FALSE | 0.5 | TRUE | Amp | N |
| F2 | RAD51D | SYBR | None | 24.253 | | 5.5248 | | 0.0028 | | 0.998061 | FALSE | 0.5 | TRUE | Amp | N |
| F3 | RAD52 | SYBR | None | 26.236 | | 7.5078 | | -0.0922 | | 1.065995 | FALSE | 0.5 | TRUE | Amp | N |
| F4 | RAD54L | SYBR | None | 22.962 | | 4.2338 | | -0.1602 | | 1.117442 | FALSE | 0.5 | TRUE | Amp | N |
| F5 | RFC1 | SYBR | None | 24.231 | | 5.5028 | | -0.0522 | | 1.036845 | FALSE | 0.5 | TRUE | Amp | N |
| F6 | RPA1 | SYBR | None | 17.648 | | -1.0802 | | -0.1662 | | 1.122099 | FALSE | 0.5 | TRUE | Amp | N |
| F7 | RPA3 | SYBR | None | 24.032 | | 5.3038 | | -0.0412 | | 1.028969 | FALSE | 0.5 | TRUE | Amp | N |
| F8 | SLK | SYBR | None | 21.067 | | 2.3388 | | -0.2202 | | 1.164895 | FALSE | 0.5 | TRUE | Amp | N |
| F9 | SMUG1 | SYBR | None | 27.292 | | 8.5638 | | 0.0908 | | 0.939002 | FALSE | 0.5 | TRUE | Amp | N |
| F10 | TDG | SYBR | None | 22.134 | | 3.4058 | | -0.1952 | | 1.144883 | FALSE | 0.5 | TRUE | Amp | N |
| F11 | TOP3A | SYBR | None | 25.658 | | 6.9298 | | 0.2458 | | 0.843348 | FALSE | 0.5 | TRUE | Amp | N |
| F12 | TOP3B | SYBR | None | 27.776 | | 9.0478 | | -0.2022 | | 1.150451 | FALSE | 0.5 | TRUE | Amp | N |
| G1 | TREX1 | SYBR | None | 28.46 | | 9.7318 | | 0.0978 | | 0.934457 | FALSE | 0.5 | TRUE | Amp | N |
| G2 | UNG | SYBR | None | 20.231 | | 1.5028 | | -0.0472 | | 1.033258 | FALSE | 0.5 | TRUE | Amp | N |
| G3 | XAB2 | SYBR | None | 24.213 | | 5.4848 | | 0.0908 | | 0.939002 | FALSE | 0.5 | TRUE | Amp | N |
| G4 | XPA | SYBR | None | 31.944 | | 13.2158 | | 0.1618 | | 0.893909 | FALSE | 0.5 | TRUE | Amp | N |
| G5 | XPC | SYBR | None | 26.021 | | 7.2928 | | 0.1918 | | 0.875513 | FALSE | 0.5 | TRUE | Amp | N |
| G6 | XRCC1 | SYBR | None | 20.768 | | 2.0398 | | -0.2382 | | 1.17952 | FALSE | 0.5 | TRUE | Amp | N |
| G7 | XRCC2 | SYBR | None | 21.94 | | 3.2118 | | -0.1902 | | 1.140922 | FALSE | 0.5 | TRUE | Amp | N |
| G8 | XRCC3 | SYBR | None | 24.063 | | 5.3348 | | -0.1182 | | 1.08538 | FALSE | 0.5 | TRUE | Amp | N |
| G9 | XRCC4 | SYBR | None | 25.529 | | 6.8008 | | 0.1108 | | 0.926074 | FALSE | 0.5 | TRUE | Amp | N |
| G10 | XRCC5 | SYBR | None | 17.72 | | -1.0082 | | -0.1892 | | 1.140131 | FALSE | 0.5 | TRUE | Amp | N |
| G11 | XRCC6 | SYBR | None | 19.736 | | 1.0078 | | -0.0982 | | 1.070437 | FALSE | 0.5 | TRUE | Amp | N |
| G12 | XRCC6BP1 | SYBR | None | 24.082 | | 5.3538 | | -0.1802 | | 1.133041 | FALSE | 0.5 | TRUE | Amp | N |
| H1 | ACTB | SYBR | None | 18.792 | |  | |  | |  | FALSE | 0.5 | TRUE | Amp | N |
| H2 | B2M | SYBR | None | 17.234 | |  | |  | |  | FALSE | 0.5 | TRUE | Amp | N |
| H3 | GAPDH | SYBR | None | 21.131 | |  | |  | |  | FALSE | 0.5 | TRUE | Amp | N |
| H4 | HPRT1 | SYBR | None | 21.07 | |  | |  | |  | FALSE | 0.5 | TRUE | Amp | N |
| H5 | RPLP0 | SYBR | None | 15.414 | |  | |  | |  | FALSE | 0.5 | TRUE | Amp | N |
| POI 9 |  |  |  |  | |  | |  | |  |  |  |  |  |  |
| Well Position | Gene Symbol | Reporter | Quencher | CT | | Delta Ct | | Delta Delta Ct | | 2^(-Delta Delta Ct) | Automatic Ct Threshold | Ct Threshold | Automatic Baseline | Amp Status | MTP |
| A1 | APEX1 | SYBR | None | 19.032 | | 0.1304 | | 0.1794 | | 0.88307 | FALSE | 0.5 | TRUE | Amp | N |
| A2 | APEX2 | SYBR | None | 23.163 | | 4.2614 | | 0.5264 | | 0.694285 | FALSE | 0.5 | TRUE | Amp | N |
| A3 | ATM | SYBR | None | 23.983 | | 5.0814 | | -0.0626 | | 1.044346 | FALSE | 0.5 | TRUE | Amp | N |
| A4 | ATR | SYBR | None | 23.258 | | 4.3564 | | -0.1496 | | 1.109262 | FALSE | 0.5 | TRUE | Amp | N |
| A5 | ATXN3 | SYBR | None | 20.99 | | 2.0884 | | -0.1316 | | 1.095508 | FALSE | 0.5 | TRUE | Amp | N |
| A6 | BRCA1 | SYBR | None | 21.7 | | 2.7984 | | -0.4496 | | 1.365662 | FALSE | 0.5 | TRUE | Amp | N |
| A7 | BRCA2 | SYBR | None | 18.811 | | -0.0906 | | -0.5346 | | 1.44854 | FALSE | 0.5 | TRUE | Amp | N |
| A8 | BRIP1 | SYBR | None | 23.646 | | 4.7444 | | 0.0104 | | 0.992817 | FALSE | 0.5 | TRUE | Amp | N |
| A9 | CCNH | SYBR | None | 24.903 | | 6.0014 | | -0.5476 | | 1.461652 | FALSE | 0.5 | TRUE | Amp | N |
| A10 | CCNO | SYBR | None | 23.132 | | 4.2304 | | -0.0086 | | 1.005979 | FALSE | 0.5 | TRUE | Amp | N |
| A11 | CDK7 | SYBR | None | 14.983 | | -3.9186 | | 0.2324 | | 0.851218 | FALSE | 0.5 | TRUE | Amp | N |
| A12 | DDB1 | SYBR | None | 20.848 | | 1.9464 | | -0.4106 | | 1.329239 | FALSE | 0.5 | TRUE | Amp | N |
| B1 | DDB2 | SYBR | None | 23.364 | | 4.4624 | | -0.4326 | | 1.349664 | FALSE | 0.5 | TRUE | Amp | N |
| B2 | DMC1 | SYBR | None | 27.531 | | 8.6294 | | -0.4436 | | 1.359994 | FALSE | 0.5 | TRUE | Amp | N |
| B3 | ERCC1 | SYBR | None | 22.959 | | 4.0574 | | 0.2134 | | 0.862502 | FALSE | 0.5 | TRUE | Amp | N |
| B4 | ERCC2 | SYBR | None | 24.816 | | 5.9144 | | 0.1964 | | 0.872726 | FALSE | 0.5 | TRUE | Amp | N |
| B5 | ERCC3 | SYBR | None | 22.678 | | 3.7764 | | 0.5664 | | 0.6753 | FALSE | 0.5 | TRUE | Amp | N |
| B6 | ERCC4 | SYBR | None | 27.825 | | 8.9234 | | -0.4406 | | 1.357169 | FALSE | 0.5 | TRUE | Amp | N |
| B7 | ERCC5 | SYBR | None | 25.074 | | 6.1724 | | -0.5186 | | 1.432564 | FALSE | 0.5 | TRUE | Amp | N |
| B8 | ERCC6 | SYBR | None | 18.908 | | 0.0064 | | 0.5084 | | 0.703002 | FALSE | 0.5 | TRUE | Amp | N |
| B9 | ERCC8 | SYBR | None | 22.791 | | 3.8894 | | -0.4876 | | 1.40211 | FALSE | 0.5 | TRUE | Amp | N |
| B10 | EXO1 | SYBR | None | 20.825 | | 1.9234 | | -0.0026 | | 1.001804 | FALSE | 0.5 | TRUE | Amp | N |
| B11 | FEN1 | SYBR | None | 17.848 | | -1.0536 | | -0.3736 | | 1.295582 | FALSE | 0.5 | TRUE | Amp | N |
| B12 | LIG1 | SYBR | None | 22.177 | | 3.2754 | | -0.4406 | | 1.357169 | FALSE | 0.5 | TRUE | Amp | N |
| C1 | LIG3 | SYBR | None | 25.588 | | 6.6864 | | -0.4266 | | 1.344062 | FALSE | 0.5 | TRUE | Amp | N |
| C2 | LIG4 | SYBR | None | 26.386 | | 7.4844 | | 0.6514 | | 0.636662 | FALSE | 0.5 | TRUE | Amp | N |
| C3 | MGMT | SYBR | None | 21.671 | | 2.7694 | | -0.3946 | | 1.314578 | FALSE | 0.5 | TRUE | Amp | N |
| C4 | MLH1 | SYBR | None | 23.459 | | 4.5574 | | 0.6014 | | 0.659114 | FALSE | 0.5 | TRUE | Amp | N |
| C5 | MLH3 | SYBR | None | 25.15 | | 6.2484 | | -0.4986 | | 1.412842 | FALSE | 0.5 | TRUE | Amp | N |
| C6 | MMS19 | SYBR | None | 26.899 | | 7.9974 | | 0.6134 | | 0.653654 | FALSE | 0.5 | TRUE | Amp | N |
| C7 | MPG | SYBR | None | 22.529 | | 3.6274 | | 0.5544 | | 0.68094 | FALSE | 0.5 | TRUE | Amp | N |
| C8 | MRE11A | SYBR | None | 18.403 | | -0.4986 | | -0.4346 | | 1.351536 | FALSE | 0.5 | TRUE | Amp | N |
| C9 | MSH2 | SYBR | None | 17.685 | | -1.2166 | | 0.4144 | | 0.750331 | FALSE | 0.5 | TRUE | Amp | N |
| C10 | MSH3 | SYBR | None | 20.151 | | 1.2494 | | -0.1096 | | 1.078929 | FALSE | 0.5 | TRUE | Amp | N |
| C11 | MSH4 | SYBR | None | 21.015 | | 2.1134 | | -0.4186 | | 1.33663 | FALSE | 0.5 | TRUE | Amp | N |
| C12 | MSH5 | SYBR | None | 29.139 | | 10.2374 | | -0.1186 | | 1.085681 | FALSE | 0.5 | TRUE | Amp | N |
| D1 | MSH6 | SYBR | None | 21.762 | | 2.8604 | | 0.4584 | | 0.727793 | FALSE | 0.5 | TRUE | Amp | N |
| D2 | MUTYH | SYBR | None | 24.875 | | 5.9734 | | 0.5614 | | 0.677644 | FALSE | 0.5 | TRUE | Amp | N |
| D3 | NEIL1 | SYBR | None | 28.236 | | 9.3344 | | 0.7114 | | 0.610727 | FALSE | 0.5 | TRUE | Amp | N |
| D4 | NEIL2 | SYBR | None | 25.003 | | 6.1014 | | 0.5304 | | 0.692363 | FALSE | 0.5 | TRUE | Amp | N |
| D5 | NEIL3 | SYBR | None | 21.935 | | 3.0334 | | 0.1694 | | 0.889212 | FALSE | 0.5 | TRUE | Amp | N |
| D6 | NTHL1 | SYBR | None | 22.049 | | 3.1474 | | 0.5494 | | 0.683304 | FALSE | 0.5 | TRUE | Amp | N |
| D7 | OGG1 | SYBR | None | 22.063 | | 3.1614 | | -0.4356 | | 1.352473 | FALSE | 0.5 | TRUE | Amp | N |
| D8 | PARP1 | SYBR | None | 21.832 | | 2.9304 | | 0.2544 | | 0.838336 | FALSE | 0.5 | TRUE | Amp | N |
| D9 | PARP2 | SYBR | None | 22.057 | | 3.1554 | | 0.4424 | | 0.735909 | FALSE | 0.5 | TRUE | Amp | N |
| D10 | PARP3 | SYBR | None | 25.193 | | 6.2914 | | 0.5604 | | 0.678114 | FALSE | 0.5 | TRUE | Amp | N |
| D11 | PMS1 | SYBR | None | 18.396 | | -0.5056 | | -0.4096 | | 1.328317 | FALSE | 0.5 | TRUE | Amp | N |
| D12 | PMS2 | SYBR | None | 23.318 | | 4.4164 | | 0.5744 | | 0.671565 | FALSE | 0.5 | TRUE | Amp | N |
| E1 | PNKP | SYBR | None | 25.392 | | 6.4904 | | 0.2694 | | 0.829665 | FALSE | 0.5 | TRUE | Amp | N |
| E2 | POLB | SYBR | None | 18.194 | | -0.7076 | | -0.0626 | | 1.044346 | FALSE | 0.5 | TRUE | Amp | N |
| E3 | POLD3 | SYBR | None | 21.235 | | 2.3334 | | 0.5684 | | 0.674364 | FALSE | 0.5 | TRUE | Amp | N |
| E4 | POLL | SYBR | None | 24.949 | | 6.0474 | | -0.4696 | | 1.384725 | FALSE | 0.5 | TRUE | Amp | N |
| E5 | PRKDC | SYBR | None | 24.239 | | 5.3374 | | -0.1216 | | 1.087941 | FALSE | 0.5 | TRUE | Amp | N |
| E6 | RAD18 | SYBR | None | 17.988 | | -0.9136 | | -0.4816 | | 1.396291 | FALSE | 0.5 | TRUE | Amp | N |
| E7 | RAD21 | SYBR | None | 18.634 | | -0.2676 | | -0.4026 | | 1.321888 | FALSE | 0.5 | TRUE | Amp | N |
| E8 | RAD23A | SYBR | None | 22.939 | | 4.0374 | | -0.0956 | | 1.06851 | FALSE | 0.5 | TRUE | Amp | N |
| E9 | RAD23B | SYBR | None | 24.676 | | 5.7744 | | -0.5196 | | 1.433558 | FALSE | 0.5 | TRUE | Amp | N |
| E10 | RAD50 | SYBR | None | 20.144 | | 1.2424 | | -0.3736 | | 1.295582 | FALSE | 0.5 | TRUE | Amp | N |
| E11 | RAD51 | SYBR | None | 21.942 | | 3.0404 | | -0.0006 | | 1.000416 | FALSE | 0.5 | TRUE | Amp | N |
| E12 | RAD51B | SYBR | None | 20.581 | | 1.6794 | | -0.1426 | | 1.103893 | FALSE | 0.5 | TRUE | Amp | N |
| F1 | RAD51C | SYBR | None | 20.12 | | 1.2184 | | 0.5114 | | 0.701541 | FALSE | 0.5 | TRUE | Amp | N |
| F2 | RAD51D | SYBR | None | 24.976 | | 6.0744 | | 0.5524 | | 0.681885 | FALSE | 0.5 | TRUE | Amp | N |
| F3 | RAD52 | SYBR | None | 25.969 | | 7.0674 | | -0.5326 | | 1.446534 | FALSE | 0.5 | TRUE | Amp | N |
| F4 | RAD54L | SYBR | None | 22.84 | | 3.9384 | | -0.4556 | | 1.371353 | FALSE | 0.5 | TRUE | Amp | N |
| F5 | RFC1 | SYBR | None | 24.044 | | 5.1424 | | -0.4126 | | 1.331083 | FALSE | 0.5 | TRUE | Amp | N |
| F6 | RPA1 | SYBR | None | 17.624 | | -1.2776 | | -0.3636 | | 1.286632 | FALSE | 0.5 | TRUE | Amp | N |
| F7 | RPA3 | SYBR | None | 23.662 | | 4.7604 | | -0.5846 | | 1.499623 | FALSE | 0.5 | TRUE | Amp | N |
| F8 | SLK | SYBR | None | 22.038 | | 3.1364 | | 0.5774 | | 0.67017 | FALSE | 0.5 | TRUE | Amp | N |
| F9 | SMUG1 | SYBR | None | 27.259 | | 8.3574 | | -0.1156 | | 1.083426 | FALSE | 0.5 | TRUE | Amp | N |
| F10 | TDG | SYBR | None | 22.353 | | 3.4514 | | -0.1496 | | 1.109262 | FALSE | 0.5 | TRUE | Amp | N |
| F11 | TOP3A | SYBR | None | 26.133 | | 7.2314 | | 0.5474 | | 0.684252 | FALSE | 0.5 | TRUE | Amp | N |
| F12 | TOP3B | SYBR | None | 28.042 | | 9.1404 | | -0.1096 | | 1.078929 | FALSE | 0.5 | TRUE | Amp | N |
| G1 | TREX1 | SYBR | None | 29.192 | | 10.2904 | | 0.6564 | | 0.63446 | FALSE | 0.5 | TRUE | Amp | N |
| G2 | UNG | SYBR | None | 20.062 | | 1.1604 | | -0.3896 | | 1.31003 | FALSE | 0.5 | TRUE | Amp | N |
| G3 | XAB2 | SYBR | None | 23.816 | | 4.9144 | | -0.4796 | | 1.394357 | FALSE | 0.5 | TRUE | Amp | N |
| G4 | XPA | SYBR | None | 31.816 | | 12.9144 | | -0.1396 | | 1.1016 | FALSE | 0.5 | TRUE | Amp | N |
| G5 | XPC | SYBR | None | 25.532 | | 6.6304 | | -0.4706 | | 1.385686 | FALSE | 0.5 | TRUE | Amp | N |
| G6 | XRCC1 | SYBR | None | 21.059 | | 2.1574 | | -0.1206 | | 1.087187 | FALSE | 0.5 | TRUE | Amp | N |
| G7 | XRCC2 | SYBR | None | 22.234 | | 3.3324 | | -0.0696 | | 1.049426 | FALSE | 0.5 | TRUE | Amp | N |
| G8 | XRCC3 | SYBR | None | 24.201 | | 5.2994 | | -0.1536 | | 1.112342 | FALSE | 0.5 | TRUE | Amp | N |
| G9 | XRCC4 | SYBR | None | 26.128 | | 7.2264 | | 0.5364 | | 0.689489 | FALSE | 0.5 | TRUE | Amp | N |
| G10 | XRCC5 | SYBR | None | 18.546 | | -0.3556 | | 0.4634 | | 0.725275 | FALSE | 0.5 | TRUE | Amp | N |
| G11 | XRCC6 | SYBR | None | 20.464 | | 1.5624 | | 0.4564 | | 0.728803 | FALSE | 0.5 | TRUE | Amp | N |
| G12 | XRCC6BP1 | SYBR | None | 24.404 | | 5.5024 | | -0.0316 | | 1.022145 | FALSE | 0.5 | TRUE | Amp | N |
| H1 | ACTB | SYBR | None | 18.986 | |  | |  | |  | FALSE | 0.5 | TRUE | Amp | N |
| H2 | B2M | SYBR | None | 17.437 | |  | |  | |  | FALSE | 0.5 | TRUE | Amp | N |
| H3 | GAPDH | SYBR | None | 21.394 | |  | |  | |  | FALSE | 0.5 | TRUE | Amp | N |
| H4 | HPRT1 | SYBR | None | 21.082 | |  | |  | |  | FALSE | 0.5 | TRUE | Amp | N |
| H5 | RPLP0 | SYBR | None | 15.609 | |  | |  | |  | FALSE | 0.5 | TRUE | Amp | N |
| POI 10 | Showed turbulent expression of the control genes and was excluded. | | | | | | | | | |  |  |  |  |  |
| POI 11 |  |  |  |  |  | |  | |  | |  |  |  |  |  |
| Well Position | Gene Symbol | Reporter | Quencher | CT | Delta Ct | | Delta Delta Ct | | 2^(-Delta Delta Ct) | | Automatic Ct Threshold | Ct Threshold | Automatic Baseline | Amp Status | MTP |
| A1 | APEX1 | SYBR | None | 18.589 | 0.1334 | | 0.1824 | | 0.881236 | | FALSE | 0.5 | TRUE | Amp | N |
| A2 | APEX2 | SYBR | None | 22.114 | 3.6584 | | -0.0766 | | 1.05453 | | FALSE | 0.5 | TRUE | Amp | N |
| A3 | ATM | SYBR | None | 23.762 | 5.3064 | | 0.1624 | | 0.893537 | | FALSE | 0.5 | TRUE | Amp | N |
| A4 | ATR | SYBR | None | 22.951 | 4.4954 | | -0.0106 | | 1.007374 | | FALSE | 0.5 | TRUE | Amp | N |
| A5 | ATXN3 | SYBR | None | 21.137 | 2.6814 | | 0.4614 | | 0.726281 | | FALSE | 0.5 | TRUE | Amp | N |
| A6 | BRCA1 | SYBR | None | 22.231 | 3.7754 | | 0.5274 | | 0.693804 | | FALSE | 0.5 | TRUE | Amp | N |
| A7 | BRCA2 | SYBR | None | 18.432 | -0.0236 | | -0.4676 | | 1.382807 | | FALSE | 0.5 | TRUE | Amp | N |
| A8 | BRIP1 | SYBR | None | 23.108 | 4.6524 | | -0.0816 | | 1.058191 | | FALSE | 0.5 | TRUE | Amp | N |
| A9 | CCNH | SYBR | None | 24.872 | 6.4164 | | -0.1326 | | 1.096268 | | FALSE | 0.5 | TRUE | Amp | N |
| A10 | CCNO | SYBR | None | 22.687 | 4.2314 | | -0.0076 | | 1.005282 | | FALSE | 0.5 | TRUE | Amp | N |
| A11 | CDK7 | SYBR | None | 14.691 | -3.7646 | | 0.3864 | | 0.765036 | | FALSE | 0.5 | TRUE | Amp | N |
| A12 | DDB1 | SYBR | None | 20.691 | 2.2354 | | -0.1216 | | 1.087941 | | FALSE | 0.5 | TRUE | Amp | N |
| B1 | DDB2 | SYBR | None | 23.559 | 5.1034 | | 0.2084 | | 0.865497 | | FALSE | 0.5 | TRUE | Amp | N |
| B2 | DMC1 | SYBR | None | 27.328 | 8.8724 | | -0.2006 | | 1.149176 | | FALSE | 0.5 | TRUE | Amp | N |
| B3 | ERCC1 | SYBR | None | 22.574 | 4.1184 | | 0.2744 | | 0.826794 | | FALSE | 0.5 | TRUE | Amp | N |
| B4 | ERCC2 | SYBR | None | 23.61 | 5.1544 | | -0.5636 | | 1.477953 | | FALSE | 0.5 | TRUE | Amp | N |
| B5 | ERCC3 | SYBR | None | 21.608 | 3.1524 | | -0.0576 | | 1.040733 | | FALSE | 0.5 | TRUE | Amp | N |
| B6 | ERCC4 | SYBR | None | 28.499 | 10.0434 | | 0.6794 | | 0.624425 | | FALSE | 0.5 | TRUE | Amp | N |
| B7 | ERCC5 | SYBR | None | 24.704 | 6.2484 | | -0.4426 | | 1.359051 | | FALSE | 0.5 | TRUE | Amp | N |
| B8 | ERCC6 | SYBR | None | 17.523 | -0.9326 | | -0.4306 | | 1.347794 | | FALSE | 0.5 | TRUE | Amp | N |
| B9 | ERCC8 | SYBR | None | 22.821 | 4.3654 | | -0.0116 | | 1.008073 | | FALSE | 0.5 | TRUE | Amp | N |
| B10 | EXO1 | SYBR | None | 20.885 | 2.4294 | | 0.5034 | | 0.705442 | | FALSE | 0.5 | TRUE | Amp | N |
| B11 | FEN1 | SYBR | None | 18.153 | -0.3026 | | 0.3774 | | 0.769824 | | FALSE | 0.5 | TRUE | Amp | N |
| B12 | LIG1 | SYBR | None | 21.722 | 3.2664 | | -0.4496 | | 1.365662 | | FALSE | 0.5 | TRUE | Amp | N |
| C1 | LIG3 | SYBR | None | 25.382 | 6.9264 | | -0.1866 | | 1.138078 | | FALSE | 0.5 | TRUE | Amp | N |
| C2 | LIG4 | SYBR | None | 25.912 | 7.4564 | | 0.6234 | | 0.649139 | | FALSE | 0.5 | TRUE | Amp | N |
| C3 | MGMT | SYBR | None | 22.063 | 3.6074 | | 0.4434 | | 0.735399 | | FALSE | 0.5 | TRUE | Amp | N |
| C4 | MLH1 | SYBR | None | 21.952 | 3.4964 | | -0.4596 | | 1.37516 | | FALSE | 0.5 | TRUE | Amp | N |
| C5 | MLH3 | SYBR | None | 25.009 | 6.5534 | | -0.1936 | | 1.143614 | | FALSE | 0.5 | TRUE | Amp | N |
| C6 | MMS19 | SYBR | None | 26.413 | 7.9574 | | 0.5734 | | 0.672031 | | FALSE | 0.5 | TRUE | Amp | N |
| C7 | MPG | SYBR | None | 21.392 | 2.9364 | | -0.1366 | | 1.099311 | | FALSE | 0.5 | TRUE | Amp | N |
| C8 | MRE11A | SYBR | None | 17.97 | -0.4856 | | -0.4216 | | 1.339412 | | FALSE | 0.5 | TRUE | Amp | N |
| C9 | MSH2 | SYBR | None | 16.441 | -2.0146 | | -0.3836 | | 1.304593 | | FALSE | 0.5 | TRUE | Amp | N |
| C10 | MSH3 | SYBR | None | 20.289 | 1.8334 | | 0.4744 | | 0.719766 | | FALSE | 0.5 | TRUE | Amp | N |
| C11 | MSH4 | SYBR | None | 20.503 | 2.0474 | | -0.4846 | | 1.399198 | | FALSE | 0.5 | TRUE | Amp | N |
| C12 | MSH5 | SYBR | None | 29.423 | 10.9674 | | 0.6114 | | 0.654561 | | FALSE | 0.5 | TRUE | Amp | N |
| D1 | MSH6 | SYBR | None | 20.732 | 2.2764 | | -0.1256 | | 1.090961 | | FALSE | 0.5 | TRUE | Amp | N |
| D2 | MUTYH | SYBR | None | 23.396 | 4.9404 | | -0.4716 | | 1.386646 | | FALSE | 0.5 | TRUE | Amp | N |
| D3 | NEIL1 | SYBR | None | 27.885 | 9.4294 | | 0.8064 | | 0.571807 | | FALSE | 0.5 | TRUE | Amp | N |
| D4 | NEIL2 | SYBR | None | 24.509 | 6.0534 | | 0.4824 | | 0.715786 | | FALSE | 0.5 | TRUE | Amp | N |
| D5 | NEIL3 | SYBR | None | 21.197 | 2.7414 | | -0.1226 | | 1.088695 | | FALSE | 0.5 | TRUE | Amp | N |
| D6 | NTHL1 | SYBR | None | 20.556 | 2.1004 | | -0.4976 | | 1.411863 | | FALSE | 0.5 | TRUE | Amp | N |
| D7 | OGG1 | SYBR | None | 21.676 | 3.2204 | | -0.3766 | | 1.298279 | | FALSE | 0.5 | TRUE | Amp | N |
| D8 | PARP1 | SYBR | None | 21.637 | 3.1814 | | 0.5054 | | 0.704465 | | FALSE | 0.5 | TRUE | Amp | N |
| D9 | PARP2 | SYBR | None | 21.375 | 2.9194 | | 0.2064 | | 0.866697 | | FALSE | 0.5 | TRUE | Amp | N |
| D10 | PARP3 | SYBR | None | 24.124 | 5.6684 | | -0.0626 | | 1.044346 | | FALSE | 0.5 | TRUE | Amp | N |
| D11 | PMS1 | SYBR | None | 17.843 | -0.6126 | | -0.5166 | | 1.43058 | | FALSE | 0.5 | TRUE | Amp | N |
| D12 | PMS2 | SYBR | None | 22.782 | 4.3264 | | 0.4844 | | 0.714794 | | FALSE | 0.5 | TRUE | Amp | N |
| E1 | PNKP | SYBR | None | 24.273 | 5.8174 | | -0.4036 | | 1.322805 | | FALSE | 0.5 | TRUE | Amp | N |
| E2 | POLB | SYBR | None | 17.362 | -1.0936 | | -0.4486 | | 1.364715 | | FALSE | 0.5 | TRUE | Amp | N |
| E3 | POLD3 | SYBR | None | 19.86 | 1.4044 | | -0.3606 | | 1.28396 | | FALSE | 0.5 | TRUE | Amp | N |
| E4 | POLL | SYBR | None | 24.565 | 6.1094 | | -0.4076 | | 1.326477 | | FALSE | 0.5 | TRUE | Amp | N |
| E5 | PRKDC | SYBR | None | 23.495 | 5.0394 | | -0.4196 | | 1.337557 | | FALSE | 0.5 | TRUE | Amp | N |
| E6 | RAD18 | SYBR | None | 17.891 | -0.5646 | | -0.1326 | | 1.096268 | | FALSE | 0.5 | TRUE | Amp | N |
| E7 | RAD21 | SYBR | None | 18.229 | -0.2266 | | -0.3616 | | 1.28485 | | FALSE | 0.5 | TRUE | Amp | N |
| E8 | RAD23A | SYBR | None | 23.131 | 4.6754 | | 0.5424 | | 0.686628 | | FALSE | 0.5 | TRUE | Amp | N |
| E9 | RAD23B | SYBR | None | 24.743 | 6.2874 | | -0.0066 | | 1.004585 | | FALSE | 0.5 | TRUE | Amp | N |
| E10 | RAD50 | SYBR | None | 19.702 | 1.2464 | | -0.3696 | | 1.291995 | | FALSE | 0.5 | TRUE | Amp | N |
| E11 | RAD51 | SYBR | None | 20.547 | 2.0914 | | -0.9496 | | 1.931337 | | FALSE | 0.5 | TRUE | Amp | N |
| E12 | RAD51B | SYBR | None | 20.744 | 2.2884 | | 0.4664 | | 0.723768 | | FALSE | 0.5 | TRUE | Amp | N |
| F1 | RAD51C | SYBR | None | 19.126 | 0.6704 | | -0.0366 | | 1.025694 | | FALSE | 0.5 | TRUE | Amp | N |
| F2 | RAD51D | SYBR | None | 23.539 | 5.0834 | | -0.4386 | | 1.355289 | | FALSE | 0.5 | TRUE | Amp | N |
| F3 | RAD52 | SYBR | None | 26.644 | 8.1884 | | 0.5884 | | 0.66508 | | FALSE | 0.5 | TRUE | Amp | N |
| F4 | RAD54L | SYBR | None | 22.454 | 3.9984 | | -0.3956 | | 1.31549 | | FALSE | 0.5 | TRUE | Amp | N |
| F5 | RFC1 | SYBR | None | 23.886 | 5.4304 | | -0.1246 | | 1.090205 | | FALSE | 0.5 | TRUE | Amp | N |
| F6 | RPA1 | SYBR | None | 17.673 | -0.7826 | | 0.1314 | | 0.912945 | | FALSE | 0.5 | TRUE | Amp | N |
| F7 | RPA3 | SYBR | None | 23.685 | 5.2294 | | -0.1156 | | 1.083426 | | FALSE | 0.5 | TRUE | Amp | N |
| F8 | SLK | SYBR | None | 21.194 | 2.7384 | | 0.1794 | | 0.88307 | | FALSE | 0.5 | TRUE | Amp | N |
| F9 | SMUG1 | SYBR | None | 26.49 | 8.0344 | | -0.4386 | | 1.355289 | | FALSE | 0.5 | TRUE | Amp | N |
| F10 | TDG | SYBR | None | 21.885 | 3.4294 | | -0.1716 | | 1.126307 | | FALSE | 0.5 | TRUE | Amp | N |
| F11 | TOP3A | SYBR | None | 25.137 | 6.6814 | | -0.0026 | | 1.001804 | | FALSE | 0.5 | TRUE | Amp | N |
| F12 | TOP3B | SYBR | None | 28.358 | 9.9024 | | 0.6524 | | 0.636221 | | FALSE | 0.5 | TRUE | Amp | N |
| G1 | TREX1 | SYBR | None | 27.561 | 9.1054 | | -0.5286 | | 1.442529 | | FALSE | 0.5 | TRUE | Amp | N |
| G2 | UNG | SYBR | None | 19.534 | 1.0784 | | -0.4716 | | 1.386646 | | FALSE | 0.5 | TRUE | Amp | N |
| G3 | XAB2 | SYBR | None | 23.317 | 4.8614 | | -0.5326 | | 1.446534 | | FALSE | 0.5 | TRUE | Amp | N |
| G4 | XPA | SYBR | None | 31.493 | 13.0374 | | -0.0166 | | 1.011573 | | FALSE | 0.5 | TRUE | Amp | N |
| G5 | XPC | SYBR | None | 25.762 | 7.3064 | | 0.2054 | | 0.867298 | | FALSE | 0.5 | TRUE | Amp | N |
| G6 | XRCC1 | SYBR | None | 20.286 | 1.8304 | | -0.4476 | | 1.36377 | | FALSE | 0.5 | TRUE | Amp | N |
| G7 | XRCC2 | SYBR | None | 21.398 | 2.9424 | | -0.4596 | | 1.37516 | | FALSE | 0.5 | TRUE | Amp | N |
| G8 | XRCC3 | SYBR | None | 23.782 | 5.3264 | | -0.1266 | | 1.091718 | | FALSE | 0.5 | TRUE | Amp | N |
| G9 | XRCC4 | SYBR | None | 24.71 | 6.2544 | | -0.4356 | | 1.352473 | | FALSE | 0.5 | TRUE | Amp | N |
| G10 | XRCC5 | SYBR | None | 17.49 | -0.9656 | | -0.1466 | | 1.106958 | | FALSE | 0.5 | TRUE | Amp | N |
| G11 | XRCC6 | SYBR | None | 19.203 | 0.7474 | | -0.3586 | | 1.282181 | | FALSE | 0.5 | TRUE | Amp | N |
| G12 | XRCC6BP1 | SYBR | None | 23.485 | 5.0294 | | -0.5046 | | 1.41873 | | FALSE | 0.5 | TRUE | Amp | N |
| H1 | ACTB | SYBR | None | 18.425 |  | |  | |  | | FALSE | 0.5 | TRUE | Amp | N |
| H2 | B2M | SYBR | None | 16.984 |  | |  | |  | | FALSE | 0.5 | TRUE | Amp | N |
| H3 | GAPDH | SYBR | None | 20.998 |  | |  | |  | | FALSE | 0.5 | TRUE | Amp | N |
| H4 | HPRT1 | SYBR | None | 20.775 |  | |  | |  | | FALSE | 0.5 | TRUE | Amp | N |
| H5 | RPLP0 | SYBR | None | 15.096 |  | |  | |  | | FALSE | 0.5 | TRUE | Amp | N |
| POI 12 |  |  |  |  |  | |  | |  | |  |  |  |  |  |
| Well Position | Gene Symbol | Reporter | Quencher | CT | Delta Ct | | Delta Delta Ct | | 2^(-Delta Delta Ct) | | Automatic Ct Threshold | Ct Threshold | Automatic Baseline | Amp Status | MTP |
| A1 | APEX1 | SYBR | None | 18.212 | -0.1392 | | -0.0902 | | 1.064518 | | FALSE | 0.5 | TRUE | Amp | N |
| A2 | APEX2 | SYBR | None | 22.335 | 3.9838 | | 0.2488 | | 0.841596 | | FALSE | 0.5 | TRUE | Amp | N |
| A3 | ATM | SYBR | None | 23.046 | 4.6948 | | -0.4492 | | 1.365283 | | FALSE | 0.5 | TRUE | Amp | N |
| A4 | ATR | SYBR | None | 22.34 | 3.9888 | | -0.5172 | | 1.431175 | | FALSE | 0.5 | TRUE | Amp | N |
| A5 | ATXN3 | SYBR | None | 20.218 | 1.8668 | | -0.3532 | | 1.277391 | | FALSE | 0.5 | TRUE | Amp | N |
| A6 | BRCA1 | SYBR | None | 21.784 | 3.4328 | | 0.1848 | | 0.879771 | | FALSE | 0.5 | TRUE | Amp | N |
| A7 | BRCA2 | SYBR | None | 18.681 | 0.3298 | | -0.1142 | | 1.082375 | | FALSE | 0.5 | TRUE | Amp | N |
| A8 | BRIP1 | SYBR | None | 22.662 | 4.3108 | | -0.4232 | | 1.340898 | | FALSE | 0.5 | TRUE | Amp | N |
| A9 | CCNH | SYBR | None | 24.46 | 6.1088 | | -0.4402 | | 1.356792 | | FALSE | 0.5 | TRUE | Amp | N |
| A10 | CCNO | SYBR | None | 22.17 | 3.8188 | | -0.4202 | | 1.338113 | | FALSE | 0.5 | TRUE | Amp | N |
| A11 | CDK7 | SYBR | None | 14.093 | -4.2582 | | -0.1072 | | 1.077136 | | FALSE | 0.5 | TRUE | Amp | N |
| A12 | DDB1 | SYBR | None | 20.598 | 2.2468 | | -0.1102 | | 1.079378 | | FALSE | 0.5 | TRUE | Amp | N |
| B1 | DDB2 | SYBR | None | 23.134 | 4.7828 | | -0.1122 | | 1.080875 | | FALSE | 0.5 | TRUE | Amp | N |
| B2 | DMC1 | SYBR | None | 27.005 | 8.6538 | | -0.4192 | | 1.337186 | | FALSE | 0.5 | TRUE | Amp | N |
| B3 | ERCC1 | SYBR | None | 22.19 | 3.8388 | | -0.0052 | | 1.003611 | | FALSE | 0.5 | TRUE | Amp | N |
| B4 | ERCC2 | SYBR | None | 24.327 | 5.9758 | | 0.2578 | | 0.836362 | | FALSE | 0.5 | TRUE | Amp | N |
| B5 | ERCC3 | SYBR | None | 21.449 | 3.0978 | | -0.1122 | | 1.080875 | | FALSE | 0.5 | TRUE | Amp | N |
| B6 | ERCC4 | SYBR | None | 27.291 | 8.9398 | | -0.4242 | | 1.341828 | | FALSE | 0.5 | TRUE | Amp | N |
| B7 | ERCC5 | SYBR | None | 24.989 | 6.6378 | | -0.0532 | | 1.037564 | | FALSE | 0.5 | TRUE | Amp | N |
| B8 | ERCC6 | SYBR | None | 17.498 | -0.8532 | | -0.3512 | | 1.275621 | | FALSE | 0.5 | TRUE | Amp | N |
| B9 | ERCC8 | SYBR | None | 22.298 | 3.9468 | | -0.4302 | | 1.34742 | | FALSE | 0.5 | TRUE | Amp | N |
| B10 | EXO1 | SYBR | None | 21.376 | 3.0248 | | 1.0988 | | 0.466905 | | FALSE | 0.5 | TRUE | Amp | N |
| B11 | FEN1 | SYBR | None | 18.148 | -0.2032 | | 0.4768 | | 0.71857 | | FALSE | 0.5 | TRUE | Amp | N |
| B12 | LIG1 | SYBR | None | 21.676 | 3.3248 | | -0.3912 | | 1.311484 | | FALSE | 0.5 | TRUE | Amp | N |
| C1 | LIG3 | SYBR | None | 26.07 | 7.7188 | | 0.6058 | | 0.657107 | | FALSE | 0.5 | TRUE | Amp | N |
| C2 | LIG4 | SYBR | None | 25.742 | 7.3908 | | 0.5578 | | 0.679337 | | FALSE | 0.5 | TRUE | Amp | N |
| C3 | MGMT | SYBR | None | 21.105 | 2.7538 | | -0.4102 | | 1.32887 | | FALSE | 0.5 | TRUE | Amp | N |
| C4 | MLH1 | SYBR | None | 22.22 | 3.8688 | | -0.0872 | | 1.062306 | | FALSE | 0.5 | TRUE | Amp | N |
| C5 | MLH3 | SYBR | None | 24.66 | 6.3088 | | -0.4382 | | 1.354913 | | FALSE | 0.5 | TRUE | Amp | N |
| C6 | MMS19 | SYBR | None | 25.325 | 6.9738 | | -0.4102 | | 1.32887 | | FALSE | 0.5 | TRUE | Amp | N |
| C7 | MPG | SYBR | None | 21.009 | 2.6578 | | -0.4152 | | 1.333484 | | FALSE | 0.5 | TRUE | Amp | N |
| C8 | MRE11A | SYBR | None | 18.774 | 0.4228 | | 0.4868 | | 0.713606 | | FALSE | 0.5 | TRUE | Amp | N |
| C9 | MSH2 | SYBR | None | 16.424 | -1.9272 | | -0.2962 | | 1.227906 | | FALSE | 0.5 | TRUE | Amp | N |
| C10 | MSH3 | SYBR | None | 19.377 | 1.0258 | | -0.3332 | | 1.259805 | | FALSE | 0.5 | TRUE | Amp | N |
| C11 | MSH4 | SYBR | None | 20.468 | 2.1168 | | -0.4152 | | 1.333484 | | FALSE | 0.5 | TRUE | Amp | N |
| C12 | MSH5 | SYBR | None | 28.221 | 9.8698 | | -0.4862 | | 1.40075 | | FALSE | 0.5 | TRUE | Amp | N |
| D1 | MSH6 | SYBR | None | 21.257 | 2.9058 | | 0.5038 | | 0.705247 | | FALSE | 0.5 | TRUE | Amp | N |
| D2 | MUTYH | SYBR | None | 24.295 | 5.9438 | | 0.5318 | | 0.691691 | | FALSE | 0.5 | TRUE | Amp | N |
| D3 | NEIL1 | SYBR | None | 27.142 | 8.7908 | | 0.1678 | | 0.890199 | | FALSE | 0.5 | TRUE | Amp | N |
| D4 | NEIL2 | SYBR | None | 23.433 | 5.0818 | | -0.4892 | | 1.403666 | | FALSE | 0.5 | TRUE | Amp | N |
| D5 | NEIL3 | SYBR | None | 21.143 | 2.7918 | | -0.0722 | | 1.051319 | | FALSE | 0.5 | TRUE | Amp | N |
| D6 | NTHL1 | SYBR | None | 21.215 | 2.8638 | | 0.2658 | | 0.831737 | | FALSE | 0.5 | TRUE | Amp | N |
| D7 | OGG1 | SYBR | None | 21.552 | 3.2008 | | -0.3962 | | 1.316037 | | FALSE | 0.5 | TRUE | Amp | N |
| D8 | PARP1 | SYBR | None | 21.341 | 2.9898 | | 0.3138 | | 0.80452 | | FALSE | 0.5 | TRUE | Amp | N |
| D9 | PARP2 | SYBR | None | 21.576 | 3.2248 | | 0.5118 | | 0.701347 | | FALSE | 0.5 | TRUE | Amp | N |
| D10 | PARP3 | SYBR | None | 24.688 | 6.3368 | | 0.6058 | | 0.657107 | | FALSE | 0.5 | TRUE | Amp | N |
| D11 | PMS1 | SYBR | None | 18.436 | 0.0848 | | 0.1808 | | 0.882214 | | FALSE | 0.5 | TRUE | Amp | N |
| D12 | PMS2 | SYBR | None | 22.786 | 4.4348 | | 0.5928 | | 0.663055 | | FALSE | 0.5 | TRUE | Amp | N |
| E1 | PNKP | SYBR | None | 24.483 | 6.1318 | | -0.0892 | | 1.06378 | | FALSE | 0.5 | TRUE | Amp | N |
| E2 | POLB | SYBR | None | 17.367 | -0.9842 | | -0.3392 | | 1.265055 | | FALSE | 0.5 | TRUE | Amp | N |
| E3 | POLD3 | SYBR | None | 20.337 | 1.9858 | | 0.2208 | | 0.858089 | | FALSE | 0.5 | TRUE | Amp | N |
| E4 | POLL | SYBR | None | 24.75 | 6.3988 | | -0.1182 | | 1.08538 | | FALSE | 0.5 | TRUE | Amp | N |
| E5 | PRKDC | SYBR | None | 24.4 | 6.0488 | | 0.5898 | | 0.664435 | | FALSE | 0.5 | TRUE | Amp | N |
| E6 | RAD18 | SYBR | None | 17.587 | -0.7642 | | -0.3322 | | 1.258932 | | FALSE | 0.5 | TRUE | Amp | N |
| E7 | RAD21 | SYBR | None | 18.961 | 0.6098 | | 0.4748 | | 0.719567 | | FALSE | 0.5 | TRUE | Amp | N |
| E8 | RAD23A | SYBR | None | 23.073 | 4.7218 | | 0.5888 | | 0.664896 | | FALSE | 0.5 | TRUE | Amp | N |
| E9 | RAD23B | SYBR | None | 25.221 | 6.8698 | | 0.5758 | | 0.670914 | | FALSE | 0.5 | TRUE | Amp | N |
| E10 | RAD50 | SYBR | None | 19.473 | 1.1218 | | -0.4942 | | 1.408539 | | FALSE | 0.5 | TRUE | Amp | N |
| E11 | RAD51 | SYBR | None | 21.826 | 3.4748 | | 0.4338 | | 0.740309 | | FALSE | 0.5 | TRUE | Amp | N |
| E12 | RAD51B | SYBR | None | 20.64 | 2.2888 | | 0.4668 | | 0.723568 | | FALSE | 0.5 | TRUE | Amp | N |
| F1 | RAD51C | SYBR | None | 19.499 | 1.1478 | | 0.4408 | | 0.736726 | | FALSE | 0.5 | TRUE | Amp | N |
| F2 | RAD51D | SYBR | None | 23.313 | 4.9618 | | -0.5602 | | 1.474474 | | FALSE | 0.5 | TRUE | Amp | N |
| F3 | RAD52 | SYBR | None | 25.404 | 7.0528 | | -0.5472 | | 1.461247 | | FALSE | 0.5 | TRUE | Amp | N |
| F4 | RAD54L | SYBR | None | 23.259 | 4.9078 | | 0.5138 | | 0.700375 | | FALSE | 0.5 | TRUE | Amp | N |
| F5 | RFC1 | SYBR | None | 23.826 | 5.4748 | | -0.0802 | | 1.057165 | | FALSE | 0.5 | TRUE | Amp | N |
| F6 | RPA1 | SYBR | None | 17.792 | -0.5592 | | 0.3548 | | 0.781978 | | FALSE | 0.5 | TRUE | Amp | N |
| F7 | RPA3 | SYBR | None | 23.57 | 5.2188 | | -0.1262 | | 1.091415 | | FALSE | 0.5 | TRUE | Amp | N |
| F8 | SLK | SYBR | None | 21.165 | 2.8138 | | 0.2548 | | 0.838103 | | FALSE | 0.5 | TRUE | Amp | N |
| F9 | SMUG1 | SYBR | None | 26.754 | 8.4028 | | -0.0702 | | 1.049862 | | FALSE | 0.5 | TRUE | Amp | N |
| F10 | TDG | SYBR | None | 21.879 | 3.5278 | | -0.0732 | | 1.052048 | | FALSE | 0.5 | TRUE | Amp | N |
| F11 | TOP3A | SYBR | None | 24.649 | 6.2978 | | -0.3862 | | 1.306946 | | FALSE | 0.5 | TRUE | Amp | N |
| F12 | TOP3B | SYBR | None | 27.181 | 8.8298 | | -0.4202 | | 1.338113 | | FALSE | 0.5 | TRUE | Amp | N |
| G1 | TREX1 | SYBR | None | 28.678 | 10.3268 | | 0.6928 | | 0.618652 | | FALSE | 0.5 | TRUE | Amp | N |
| G2 | UNG | SYBR | None | 19.875 | 1.5238 | | -0.0262 | | 1.018326 | | FALSE | 0.5 | TRUE | Amp | N |
| G3 | XAB2 | SYBR | None | 23.632 | 5.2808 | | -0.1132 | | 1.081625 | | FALSE | 0.5 | TRUE | Amp | N |
| G4 | XPA | SYBR | None | 31.584 | 13.2328 | | 0.1788 | | 0.883438 | | FALSE | 0.5 | TRUE | Amp | N |
| G5 | XPC | SYBR | None | 25.651 | 7.2998 | | 0.1988 | | 0.871275 | | FALSE | 0.5 | TRUE | Amp | N |
| G6 | XRCC1 | SYBR | None | 20.218 | 1.8668 | | -0.4112 | | 1.329791 | | FALSE | 0.5 | TRUE | Amp | N |
| G7 | XRCC2 | SYBR | None | 22.327 | 3.9758 | | 0.5738 | | 0.671845 | | FALSE | 0.5 | TRUE | Amp | N |
| G8 | XRCC3 | SYBR | None | 23.412 | 5.0608 | | -0.3922 | | 1.312393 | | FALSE | 0.5 | TRUE | Amp | N |
| G9 | XRCC4 | SYBR | None | 24.573 | 6.2218 | | -0.4682 | | 1.383382 | | FALSE | 0.5 | TRUE | Amp | N |
| G10 | XRCC5 | SYBR | None | 17.116 | -1.2352 | | -0.4162 | | 1.334408 | | FALSE | 0.5 | TRUE | Amp | N |
| G11 | XRCC6 | SYBR | None | 19.372 | 1.0208 | | -0.0852 | | 1.060835 | | FALSE | 0.5 | TRUE | Amp | N |
| G12 | XRCC6BP1 | SYBR | None | 23.409 | 5.0578 | | -0.4762 | | 1.391075 | | FALSE | 0.5 | TRUE | Amp | N |
| H1 | ACTB | SYBR | None | 18.215 |  | |  | |  | | FALSE | 0.5 | TRUE | Amp | N |
| H2 | B2M | SYBR | None | 16.904 |  | |  | |  | | FALSE | 0.5 | TRUE | Amp | N |
| H3 | GAPDH | SYBR | None | 20.766 |  | |  | |  | | FALSE | 0.5 | TRUE | Amp | N |
| H4 | HPRT1 | SYBR | None | 20.523 |  | |  | |  | | FALSE | 0.5 | TRUE | Amp | N |
| H5 | RPLP0 | SYBR | None | 15.348 |  | |  | |  | | FALSE | 0.5 | TRUE | Amp | N |
| POI 13 | Showed turbulent expression of the control genes and was excluded. | | | | | | | | | |  |  |  |  |  |
| POI 14 |  |  |  |  |  | |  | |  | |  |  |  |  |  |
| Well Position | Gene Symbol | Reporter | Quencher | CT | Delta Ct | | Delta Delta Ct | | 2^(-Delta Delta Ct) | | Automatic Ct Threshold | Ct Threshold | Automatic Baseline | Amp Status | MTP |
| A1 | APEX1 | SYBR | None | 18.143 | -0.54 | | -0.491 | | 1.405419 | | FALSE | 0.5 | TRUE | Amp | N |
| A2 | APEX2 | SYBR | None | 22.922 | 4.239 | | 0.504 | | 0.705149 | | FALSE | 0.5 | TRUE | Amp | N |
| A3 | ATM | SYBR | None | 24.445 | 5.762 | | 0.618 | | 0.651574 | | FALSE | 0.5 | TRUE | Amp | N |
| A4 | ATR | SYBR | None | 23.663 | 4.98 | | 0.474 | | 0.719966 | | FALSE | 0.5 | TRUE | Amp | N |
| A5 | ATXN3 | SYBR | None | 21.5 | 2.817 | | 0.597 | | 0.661127 | | FALSE | 0.5 | TRUE | Amp | N |
| A6 | BRCA1 | SYBR | None | 21.753 | 3.07 | | -0.178 | | 1.131314 | | FALSE | 0.5 | TRUE | Amp | N |
| A7 | BRCA2 | SYBR | None | 19.608 | 0.925 | | 0.481 | | 0.716481 | | FALSE | 0.5 | TRUE | Amp | N |
| A8 | BRIP1 | SYBR | None | 23.296 | 4.613 | | -0.121 | | 1.087488 | | FALSE | 0.5 | TRUE | Amp | N |
| A9 | CCNH | SYBR | None | 24.78 | 6.097 | | -0.452 | | 1.367935 | | FALSE | 0.5 | TRUE | Amp | N |
| A10 | CCNO | SYBR | None | 23.477 | 4.794 | | 0.555 | | 0.680657 | | FALSE | 0.5 | TRUE | Amp | N |
| A11 | CDK7 | SYBR | None | 14.168 | -4.515 | | -0.364 | | 1.286989 | | FALSE | 0.5 | TRUE | Amp | N |
| A12 | DDB1 | SYBR | None | 20.841 | 2.158 | | -0.199 | | 1.147902 | | FALSE | 0.5 | TRUE | Amp | N |
| B1 | DDB2 | SYBR | None | 24.106 | 5.423 | | 0.528 | | 0.693515 | | FALSE | 0.5 | TRUE | Amp | N |
| B2 | DMC1 | SYBR | None | 27.228 | 8.545 | | -0.528 | | 1.441929 | | FALSE | 0.5 | TRUE | Amp | N |
| B3 | ERCC1 | SYBR | None | 22.808 | 4.125 | | 0.281 | | 0.82302 | | FALSE | 0.5 | TRUE | Amp | N |
| B4 | ERCC2 | SYBR | None | 23.887 | 5.204 | | -0.514 | | 1.428004 | | FALSE | 0.5 | TRUE | Amp | N |
| B5 | ERCC3 | SYBR | None | 21.449 | 2.766 | | -0.444 | | 1.360371 | | FALSE | 0.5 | TRUE | Amp | N |
| B6 | ERCC4 | SYBR | None | 28.688 | 10.005 | | 0.641 | | 0.641268 | | FALSE | 0.5 | TRUE | Amp | N |
| B7 | ERCC5 | SYBR | None | 26.052 | 7.369 | | 0.678 | | 0.625031 | | FALSE | 0.5 | TRUE | Amp | N |
| B8 | ERCC6 | SYBR | None | 18.095 | -0.588 | | -0.086 | | 1.061423 | | FALSE | 0.5 | TRUE | Amp | N |
| B9 | ERCC8 | SYBR | None | 22.884 | 4.201 | | -0.176 | | 1.129747 | | FALSE | 0.5 | TRUE | Amp | N |
| B10 | EXO1 | SYBR | None | 20.438 | 1.755 | | -0.171 | | 1.125839 | | FALSE | 0.5 | TRUE | Amp | N |
| B11 | FEN1 | SYBR | None | 17.81 | -0.873 | | -0.193 | | 1.143138 | | FALSE | 0.5 | TRUE | Amp | N |
| B12 | LIG1 | SYBR | None | 21.882 | 3.199 | | -0.517 | | 1.430977 | | FALSE | 0.5 | TRUE | Amp | N |
| C1 | LIG3 | SYBR | None | 25.286 | 6.603 | | -0.51 | | 1.42405 | | FALSE | 0.5 | TRUE | Amp | N |
| C2 | LIG4 | SYBR | None | 26.132 | 7.449 | | 0.616 | | 0.652477 | | FALSE | 0.5 | TRUE | Amp | N |
| C3 | MGMT | SYBR | None | 22.094 | 3.411 | | 0.247 | | 0.842647 | | FALSE | 0.5 | TRUE | Amp | N |
| C4 | MLH1 | SYBR | None | 22.199 | 3.516 | | -0.44 | | 1.356604 | | FALSE | 0.5 | TRUE | Amp | N |
| C5 | MLH3 | SYBR | None | 25.227 | 6.544 | | -0.203 | | 1.151089 | | FALSE | 0.5 | TRUE | Amp | N |
| C6 | MMS19 | SYBR | None | 26.678 | 7.995 | | 0.611 | | 0.654743 | | FALSE | 0.5 | TRUE | Amp | N |
| C7 | MPG | SYBR | None | 21.592 | 2.909 | | -0.164 | | 1.120389 | | FALSE | 0.5 | TRUE | Amp | N |
| C8 | MRE11A | SYBR | None | 19.032 | 0.349 | | 0.413 | | 0.75106 | | FALSE | 0.5 | TRUE | Amp | N |
| C9 | MSH2 | SYBR | None | 16.889 | -1.794 | | -0.163 | | 1.119613 | | FALSE | 0.5 | TRUE | Amp | N |
| C10 | MSH3 | SYBR | None | 19.873 | 1.19 | | -0.169 | | 1.124279 | | FALSE | 0.5 | TRUE | Amp | N |
| C11 | MSH4 | SYBR | None | 21.749 | 3.066 | | 0.534 | | 0.690637 | | FALSE | 0.5 | TRUE | Amp | N |
| C12 | MSH5 | SYBR | None | 29.227 | 10.544 | | 0.188 | | 0.877822 | | FALSE | 0.5 | TRUE | Amp | N |
| D1 | MSH6 | SYBR | None | 20.944 | 2.261 | | -0.141 | | 1.102669 | | FALSE | 0.5 | TRUE | Amp | N |
| D2 | MUTYH | SYBR | None | 24.762 | 6.079 | | 0.667 | | 0.629815 | | FALSE | 0.5 | TRUE | Amp | N |
| D3 | NEIL1 | SYBR | None | 27.945 | 9.262 | | 0.639 | | 0.642158 | | FALSE | 0.5 | TRUE | Amp | N |
| D4 | NEIL2 | SYBR | None | 24.478 | 5.795 | | 0.224 | | 0.856188 | | FALSE | 0.5 | TRUE | Amp | N |
| D5 | NEIL3 | SYBR | None | 21.813 | 3.13 | | 0.266 | | 0.831622 | | FALSE | 0.5 | TRUE | Amp | N |
| D6 | NTHL1 | SYBR | None | 21.092 | 2.409 | | -0.189 | | 1.139973 | | FALSE | 0.5 | TRUE | Amp | N |
| D7 | OGG1 | SYBR | None | 21.77 | 3.087 | | -0.51 | | 1.42405 | | FALSE | 0.5 | TRUE | Amp | N |
| D8 | PARP1 | SYBR | None | 20.922 | 2.239 | | -0.437 | | 1.353786 | | FALSE | 0.5 | TRUE | Amp | N |
| D9 | PARP2 | SYBR | None | 21.869 | 3.186 | | 0.473 | | 0.720465 | | FALSE | 0.5 | TRUE | Amp | N |
| D10 | PARP3 | SYBR | None | 23.934 | 5.251 | | -0.48 | | 1.394744 | | FALSE | 0.5 | TRUE | Amp | N |
| D11 | PMS1 | SYBR | None | 18.457 | -0.226 | | -0.13 | | 1.094294 | | FALSE | 0.5 | TRUE | Amp | N |
| D12 | PMS2 | SYBR | None | 23.07 | 4.387 | | 0.545 | | 0.685391 | | FALSE | 0.5 | TRUE | Amp | N |
| E1 | PNKP | SYBR | None | 25.486 | 6.803 | | 0.582 | | 0.668037 | | FALSE | 0.5 | TRUE | Amp | N |
| E2 | POLB | SYBR | None | 17.88 | -0.803 | | -0.158 | | 1.115739 | | FALSE | 0.5 | TRUE | Amp | N |
| E3 | POLD3 | SYBR | None | 20.637 | 1.954 | | 0.189 | | 0.877214 | | FALSE | 0.5 | TRUE | Amp | N |
| E4 | POLL | SYBR | None | 24.702 | 6.019 | | -0.498 | | 1.412254 | | FALSE | 0.5 | TRUE | Amp | N |
| E5 | PRKDC | SYBR | None | 24.025 | 5.342 | | -0.117 | | 1.084477 | | FALSE | 0.5 | TRUE | Amp | N |
| E6 | RAD18 | SYBR | None | 18.139 | -0.544 | | -0.112 | | 1.080725 | | FALSE | 0.5 | TRUE | Amp | N |
| E7 | RAD21 | SYBR | None | 18.338 | -0.345 | | -0.48 | | 1.394744 | | FALSE | 0.5 | TRUE | Amp | N |
| E8 | RAD23A | SYBR | None | 22.315 | 3.632 | | -0.501 | | 1.415194 | | FALSE | 0.5 | TRUE | Amp | N |
| E9 | RAD23B | SYBR | None | 25.514 | 6.831 | | 0.537 | | 0.689203 | | FALSE | 0.5 | TRUE | Amp | N |
| E10 | RAD50 | SYBR | None | 20.785 | 2.102 | | 0.486 | | 0.714002 | | FALSE | 0.5 | TRUE | Amp | N |
| E11 | RAD51 | SYBR | None | 21.245 | 2.562 | | -0.479 | | 1.393777 | | FALSE | 0.5 | TRUE | Amp | N |
| E12 | RAD51B | SYBR | None | 20.967 | 2.284 | | 0.462 | | 0.725979 | | FALSE | 0.5 | TRUE | Amp | N |
| F1 | RAD51C | SYBR | None | 18.992 | 0.309 | | -0.398 | | 1.31768 | | FALSE | 0.5 | TRUE | Amp | N |
| F2 | RAD51D | SYBR | None | 24.014 | 5.331 | | -0.191 | | 1.141555 | | FALSE | 0.5 | TRUE | Amp | N |
| F3 | RAD52 | SYBR | None | 25.716 | 7.033 | | -0.567 | | 1.48144 | | FALSE | 0.5 | TRUE | Amp | N |
| F4 | RAD54L | SYBR | None | 23.572 | 4.889 | | 0.495 | | 0.709562 | | FALSE | 0.5 | TRUE | Amp | N |
| F5 | RFC1 | SYBR | None | 23.665 | 4.982 | | -0.573 | | 1.487614 | | FALSE | 0.5 | TRUE | Amp | N |
| F6 | RPA1 | SYBR | None | 18.192 | -0.491 | | 0.423 | | 0.745872 | | FALSE | 0.5 | TRUE | Amp | N |
| F7 | RPA3 | SYBR | None | 23.554 | 4.871 | | -0.474 | | 1.388955 | | FALSE | 0.5 | TRUE | Amp | N |
| F8 | SLK | SYBR | None | 21.108 | 2.425 | | -0.134 | | 1.097332 | | FALSE | 0.5 | TRUE | Amp | N |
| F9 | SMUG1 | SYBR | None | 27.052 | 8.369 | | -0.104 | | 1.074749 | | FALSE | 0.5 | TRUE | Amp | N |
| F10 | TDG | SYBR | None | 21.858 | 3.175 | | -0.426 | | 1.343503 | | FALSE | 0.5 | TRUE | Amp | N |
| F11 | TOP3A | SYBR | None | 25.191 | 6.508 | | -0.176 | | 1.129747 | | FALSE | 0.5 | TRUE | Amp | N |
| F12 | TOP3B | SYBR | None | 27.849 | 9.166 | | -0.084 | | 1.059953 | | FALSE | 0.5 | TRUE | Amp | N |
| G1 | TREX1 | SYBR | None | 27.807 | 9.124 | | -0.51 | | 1.42405 | | FALSE | 0.5 | TRUE | Amp | N |
| G2 | UNG | SYBR | None | 19.78 | 1.097 | | -0.453 | | 1.368884 | | FALSE | 0.5 | TRUE | Amp | N |
| G3 | XAB2 | SYBR | None | 23.908 | 5.225 | | -0.169 | | 1.124279 | | FALSE | 0.5 | TRUE | Amp | N |
| G4 | XPA | SYBR | None | 31.593 | 12.91 | | -0.144 | | 1.104964 | | FALSE | 0.5 | TRUE | Amp | N |
| G5 | XPC | SYBR | None | 25.626 | 6.943 | | -0.158 | | 1.115739 | | FALSE | 0.5 | TRUE | Amp | N |
| G6 | XRCC1 | SYBR | None | 20.541 | 1.858 | | -0.42 | | 1.337928 | | FALSE | 0.5 | TRUE | Amp | N |
| G7 | XRCC2 | SYBR | None | 21.619 | 2.936 | | -0.466 | | 1.381274 | | FALSE | 0.5 | TRUE | Amp | N |
| G8 | XRCC3 | SYBR | None | 23.685 | 5.002 | | -0.451 | | 1.366987 | | FALSE | 0.5 | TRUE | Amp | N |
| G9 | XRCC4 | SYBR | None | 24.864 | 6.181 | | -0.509 | | 1.423063 | | FALSE | 0.5 | TRUE | Amp | N |
| G10 | XRCC5 | SYBR | None | 18.281 | -0.402 | | 0.417 | | 0.74898 | | FALSE | 0.5 | TRUE | Amp | N |
| G11 | XRCC6 | SYBR | None | 19.657 | 0.974 | | -0.132 | | 1.095812 | | FALSE | 0.5 | TRUE | Amp | N |
| G12 | XRCC6BP1 | SYBR | None | 24.012 | 5.329 | | -0.205 | | 1.152686 | | FALSE | 0.5 | TRUE | Amp | N |
| H1 | ACTB | SYBR | None | 18.921 |  | |  | |  | | FALSE | 0.5 | TRUE | Amp | N |
| H2 | B2M | SYBR | None | 16.873 |  | |  | |  | | FALSE | 0.5 | TRUE | Amp | N |
| H3 | GAPDH | SYBR | None | 21.224 |  | |  | |  | | FALSE | 0.5 | TRUE | Amp | N |
| H4 | HPRT1 | SYBR | None | 20.858 |  | |  | |  | | FALSE | 0.5 | TRUE | Amp | N |
| H5 | RPLP0 | SYBR | None | 15.539 |  | |  | |  | | FALSE | 0.5 | TRUE | Amp | N |
| POI 15 |  |  |  |  |  | |  | |  | |  |  |  |  |  |
| Well Position | Gene Symbol | Reporter | Quencher | CT | Delta Ct | | Delta Delta Ct | | 2^(-Delta Delta Ct) | | Automatic Ct Threshold | Ct Threshold | Automatic Baseline | Amp Status | MTP |
| A1 | APEX1 | SYBR | None | 17.942 | -0.3954 | | -0.3464 | | 1.271384 | | FALSE | 0.5 | TRUE | Amp | N |
| A2 | APEX2 | SYBR | None | 22.684 | 4.3466 | | 0.6116 | | 0.65447 | | FALSE | 0.5 | TRUE | Amp | N |
| A3 | ATM | SYBR | None | 23.424 | 5.0866 | | -0.0574 | | 1.040589 | | FALSE | 0.5 | TRUE | Amp | N |
| A4 | ATR | SYBR | None | 22.28 | 3.9426 | | -0.5634 | | 1.477748 | | FALSE | 0.5 | TRUE | Amp | N |
| A5 | ATXN3 | SYBR | None | 21.14 | 2.8026 | | 0.5826 | | 0.667759 | | FALSE | 0.5 | TRUE | Amp | N |
| A6 | BRCA1 | SYBR | None | 22.171 | 3.8336 | | 0.5856 | | 0.666372 | | FALSE | 0.5 | TRUE | Amp | N |
| A7 | BRCA2 | SYBR | None | 19.308 | 0.9706 | | 0.5266 | | 0.694189 | | FALSE | 0.5 | TRUE | Amp | N |
| A8 | BRIP1 | SYBR | None | 23.552 | 5.2146 | | 0.4806 | | 0.71668 | | FALSE | 0.5 | TRUE | Amp | N |
| A9 | CCNH | SYBR | None | 25.512 | 7.1746 | | 0.6256 | | 0.64815 | | FALSE | 0.5 | TRUE | Amp | N |
| A10 | CCNO | SYBR | None | 23.236 | 4.8986 | | 0.6596 | | 0.633054 | | FALSE | 0.5 | TRUE | Amp | N |
| A11 | CDK7 | SYBR | None | 14.093 | -4.2444 | | -0.0934 | | 1.066882 | | FALSE | 0.5 | TRUE | Amp | N |
| A12 | DDB1 | SYBR | None | 20.324 | 1.9866 | | -0.3704 | | 1.292711 | | FALSE | 0.5 | TRUE | Amp | N |
| B1 | DDB2 | SYBR | None | 23.917 | 5.5796 | | 0.6846 | | 0.622178 | | FALSE | 0.5 | TRUE | Amp | N |
| B2 | DMC1 | SYBR | None | 27.395 | 9.0576 | | -0.0154 | | 1.010732 | | FALSE | 0.5 | TRUE | Amp | N |
| B3 | ERCC1 | SYBR | None | 21.758 | 3.4206 | | -0.4234 | | 1.341084 | | FALSE | 0.5 | TRUE | Amp | N |
| B4 | ERCC2 | SYBR | None | 23.994 | 5.6566 | | -0.0614 | | 1.043478 | | FALSE | 0.5 | TRUE | Amp | N |
| B5 | ERCC3 | SYBR | None | 21.857 | 3.5196 | | 0.3096 | | 0.806865 | | FALSE | 0.5 | TRUE | Amp | N |
| B6 | ERCC4 | SYBR | None | 27.033 | 8.6956 | | -0.6684 | | 1.589309 | | FALSE | 0.5 | TRUE | Amp | N |
| B7 | ERCC5 | SYBR | None | 24.64 | 6.3026 | | -0.3884 | | 1.308941 | | FALSE | 0.5 | TRUE | Amp | N |
| B8 | ERCC6 | SYBR | None | 18.291 | -0.0464 | | 0.4556 | | 0.729207 | | FALSE | 0.5 | TRUE | Amp | N |
| B9 | ERCC8 | SYBR | None | 22.613 | 4.2756 | | -0.1014 | | 1.072814 | | FALSE | 0.5 | TRUE | Amp | N |
| B10 | EXO1 | SYBR | None | 20.839 | 2.5016 | | 0.5756 | | 0.671007 | | FALSE | 0.5 | TRUE | Amp | N |
| B11 | FEN1 | SYBR | None | 18.199 | -0.1384 | | 0.5416 | | 0.687009 | | FALSE | 0.5 | TRUE | Amp | N |
| B12 | LIG1 | SYBR | None | 22.278 | 3.9406 | | 0.2246 | | 0.855832 | | FALSE | 0.5 | TRUE | Amp | N |
| C1 | LIG3 | SYBR | None | 25.685 | 7.3476 | | 0.2346 | | 0.849921 | | FALSE | 0.5 | TRUE | Amp | N |
| C2 | LIG4 | SYBR | None | 25.778 | 7.4406 | | 0.6076 | | 0.656288 | | FALSE | 0.5 | TRUE | Amp | N |
| C3 | MGMT | SYBR | None | 21.088 | 2.7506 | | -0.4134 | | 1.331821 | | FALSE | 0.5 | TRUE | Amp | N |
| C4 | MLH1 | SYBR | None | 22.171 | 3.8336 | | -0.1224 | | 1.088544 | | FALSE | 0.5 | TRUE | Amp | N |
| C5 | MLH3 | SYBR | None | 25.722 | 7.3846 | | 0.6376 | | 0.642781 | | FALSE | 0.5 | TRUE | Amp | N |
| C6 | MMS19 | SYBR | None | 25.656 | 7.3186 | | -0.0654 | | 1.046375 | | FALSE | 0.5 | TRUE | Amp | N |
| C7 | MPG | SYBR | None | 21.932 | 3.5946 | | 0.5216 | | 0.696599 | | FALSE | 0.5 | TRUE | Amp | N |
| C8 | MRE11A | SYBR | None | 18.792 | 0.4546 | | 0.5186 | | 0.698049 | | FALSE | 0.5 | TRUE | Amp | N |
| C9 | MSH2 | SYBR | None | 16.353 | -1.9844 | | -0.3534 | | 1.277568 | | FALSE | 0.5 | TRUE | Amp | N |
| C10 | MSH3 | SYBR | None | 19.66 | 1.3226 | | -0.0364 | | 1.025552 | | FALSE | 0.5 | TRUE | Amp | N |
| C11 | MSH4 | SYBR | None | 20.526 | 2.1886 | | -0.3434 | | 1.268743 | | FALSE | 0.5 | TRUE | Amp | N |
| C12 | MSH5 | SYBR | None | 28.263 | 9.9256 | | -0.4304 | | 1.347607 | | FALSE | 0.5 | TRUE | Amp | N |
| D1 | MSH6 | SYBR | None | 20.388 | 2.0506 | | -0.3514 | | 1.275798 | | FALSE | 0.5 | TRUE | Amp | N |
| D2 | MUTYH | SYBR | None | 23.207 | 4.8696 | | -0.5424 | | 1.456393 | | FALSE | 0.5 | TRUE | Amp | N |
| D3 | NEIL1 | SYBR | None | 27.573 | 9.2356 | | 0.6126 | | 0.654017 | | FALSE | 0.5 | TRUE | Amp | N |
| D4 | NEIL2 | SYBR | None | 23.521 | 5.1836 | | -0.3874 | | 1.308034 | | FALSE | 0.5 | TRUE | Amp | N |
| D5 | NEIL3 | SYBR | None | 21.836 | 3.4986 | | 0.6346 | | 0.644119 | | FALSE | 0.5 | TRUE | Amp | N |
| D6 | NTHL1 | SYBR | None | 20.421 | 2.0836 | | -0.5144 | | 1.4284 | | FALSE | 0.5 | TRUE | Amp | N |
| D7 | OGG1 | SYBR | None | 21.854 | 3.5166 | | -0.0804 | | 1.057311 | | FALSE | 0.5 | TRUE | Amp | N |
| D8 | PARP1 | SYBR | None | 20.972 | 2.6346 | | -0.0414 | | 1.029112 | | FALSE | 0.5 | TRUE | Amp | N |
| D9 | PARP2 | SYBR | None | 21.647 | 3.3096 | | 0.5966 | | 0.661311 | | FALSE | 0.5 | TRUE | Amp | N |
| D10 | PARP3 | SYBR | None | 23.581 | 5.2436 | | -0.4874 | | 1.401916 | | FALSE | 0.5 | TRUE | Amp | N |
| D11 | PMS1 | SYBR | None | 18.153 | -0.1844 | | -0.0884 | | 1.06319 | | FALSE | 0.5 | TRUE | Amp | N |
| D12 | PMS2 | SYBR | None | 22.07 | 3.7326 | | -0.1094 | | 1.078779 | | FALSE | 0.5 | TRUE | Amp | N |
| E1 | PNKP | SYBR | None | 24.435 | 6.0976 | | -0.1234 | | 1.089299 | | FALSE | 0.5 | TRUE | Amp | N |
| E2 | POLB | SYBR | None | 17.632 | -0.7054 | | -0.0604 | | 1.042755 | | FALSE | 0.5 | TRUE | Amp | N |
| E3 | POLD3 | SYBR | None | 20.644 | 2.3066 | | 0.5416 | | 0.687009 | | FALSE | 0.5 | TRUE | Amp | N |
| E4 | POLL | SYBR | None | 24.45 | 6.1126 | | -0.4044 | | 1.323538 | | FALSE | 0.5 | TRUE | Amp | N |
| E5 | PRKDC | SYBR | None | 23.387 | 5.0496 | | -0.4094 | | 1.328133 | | FALSE | 0.5 | TRUE | Amp | N |
| E6 | RAD18 | SYBR | None | 18.457 | 0.1196 | | 0.5516 | | 0.682263 | | FALSE | 0.5 | TRUE | Amp | N |
| E7 | RAD21 | SYBR | None | 18.154 | -0.1834 | | -0.3184 | | 1.246947 | | FALSE | 0.5 | TRUE | Amp | N |
| E8 | RAD23A | SYBR | None | 22.103 | 3.7656 | | -0.3674 | | 1.290026 | | FALSE | 0.5 | TRUE | Amp | N |
| E9 | RAD23B | SYBR | None | 24.523 | 6.1856 | | -0.1084 | | 1.078032 | | FALSE | 0.5 | TRUE | Amp | N |
| E10 | RAD50 | SYBR | None | 19.542 | 1.2046 | | -0.4114 | | 1.329976 | | FALSE | 0.5 | TRUE | Amp | N |
| E11 | RAD51 | SYBR | None | 20.897 | 2.5596 | | -0.4814 | | 1.396098 | | FALSE | 0.5 | TRUE | Amp | N |
| E12 | RAD51B | SYBR | None | 20.633 | 2.2956 | | 0.4736 | | 0.720165 | | FALSE | 0.5 | TRUE | Amp | N |
| F1 | RAD51C | SYBR | None | 19.533 | 1.1956 | | 0.4886 | | 0.712716 | | FALSE | 0.5 | TRUE | Amp | N |
| F2 | RAD51D | SYBR | None | 23.741 | 5.4036 | | -0.1184 | | 1.08553 | | FALSE | 0.5 | TRUE | Amp | N |
| F3 | RAD52 | SYBR | None | 26.173 | 7.8356 | | 0.2356 | | 0.849332 | | FALSE | 0.5 | TRUE | Amp | N |
| F4 | RAD54L | SYBR | None | 22.608 | 4.2706 | | -0.1234 | | 1.089299 | | FALSE | 0.5 | TRUE | Amp | N |
| F5 | RFC1 | SYBR | None | 24.112 | 5.7746 | | 0.2196 | | 0.858804 | | FALSE | 0.5 | TRUE | Amp | N |
| F6 | RPA1 | SYBR | None | 17.939 | -0.3984 | | 0.5156 | | 0.699502 | | FALSE | 0.5 | TRUE | Amp | N |
| F7 | RPA3 | SYBR | None | 23.31 | 4.9726 | | -0.3724 | | 1.294505 | | FALSE | 0.5 | TRUE | Amp | N |
| F8 | SLK | SYBR | None | 20.84 | 2.5026 | | -0.0564 | | 1.039868 | | FALSE | 0.5 | TRUE | Amp | N |
| F9 | SMUG1 | SYBR | None | 26.375 | 8.0376 | | -0.4354 | | 1.352286 | | FALSE | 0.5 | TRUE | Amp | N |
| F10 | TDG | SYBR | None | 21.831 | 3.4936 | | -0.1074 | | 1.077285 | | FALSE | 0.5 | TRUE | Amp | N |
| F11 | TOP3A | SYBR | None | 25.713 | 7.3756 | | 0.6916 | | 0.619167 | | FALSE | 0.5 | TRUE | Amp | N |
| F12 | TOP3B | SYBR | None | 28.293 | 9.9556 | | 0.7056 | | 0.613187 | | FALSE | 0.5 | TRUE | Amp | N |
| G1 | TREX1 | SYBR | None | 27.57 | 9.2326 | | -0.4014 | | 1.320789 | | FALSE | 0.5 | TRUE | Amp | N |
| G2 | UNG | SYBR | None | 19.772 | 1.4346 | | -0.1154 | | 1.083275 | | FALSE | 0.5 | TRUE | Amp | N |
| G3 | XAB2 | SYBR | None | 24.371 | 6.0336 | | 0.6396 | | 0.641891 | | FALSE | 0.5 | TRUE | Amp | N |
| G4 | XPA | SYBR | None | 30.919 | 12.5816 | | -0.4724 | | 1.387416 | | FALSE | 0.5 | TRUE | Amp | N |
| G5 | XPC | SYBR | None | 25.659 | 7.3216 | | 0.2206 | | 0.858208 | | FALSE | 0.5 | TRUE | Amp | N |
| G6 | XRCC1 | SYBR | None | 20.27 | 1.9326 | | -0.3454 | | 1.270503 | | FALSE | 0.5 | TRUE | Amp | N |
| G7 | XRCC2 | SYBR | None | 21.368 | 3.0306 | | -0.3714 | | 1.293608 | | FALSE | 0.5 | TRUE | Amp | N |
| G8 | XRCC3 | SYBR | None | 23.348 | 5.0106 | | -0.4424 | | 1.358863 | | FALSE | 0.5 | TRUE | Amp | N |
| G9 | XRCC4 | SYBR | None | 24.92 | 6.5826 | | -0.1074 | | 1.077285 | | FALSE | 0.5 | TRUE | Amp | N |
| G10 | XRCC5 | SYBR | None | 17.192 | -1.1454 | | -0.3264 | | 1.253881 | | FALSE | 0.5 | TRUE | Amp | N |
| G11 | XRCC6 | SYBR | None | 19.719 | 1.3816 | | 0.2756 | | 0.826107 | | FALSE | 0.5 | TRUE | Amp | N |
| G12 | XRCC6BP1 | SYBR | None | 23.753 | 5.4156 | | -0.1184 | | 1.08553 | | FALSE | 0.5 | TRUE | Amp | N |
| H1 | ACTB | SYBR | None | 18.156 |  | |  | |  | | FALSE | 0.5 | TRUE | Amp | N |
| H2 | B2M | SYBR | None | 16.532 |  | |  | |  | | FALSE | 0.5 | TRUE | Amp | N |
| H3 | GAPDH | SYBR | None | 20.968 |  | |  | |  | | FALSE | 0.5 | TRUE | Amp | N |
| H4 | HPRT1 | SYBR | None | 20.724 |  | |  | |  | | FALSE | 0.5 | TRUE | Amp | N |
| H5 | RPLP0 | SYBR | None | 15.307 |  | |  | |  | | FALSE | 0.5 | TRUE | Amp | N |
| POI 16 |  |  |  |  |  | |  | |  | |  |  |  |  |  |
| Well Position | Gene Symbol | Reporter | Quencher | CT | Delta Ct | | Delta Delta Ct | | 2^(-Delta Delta Ct) | | Automatic Ct Threshold | Ct Threshold | Automatic Baseline | Amp Status | MTP |
| A1 | APEX1 | SYBR | None | 18.964 | 0.2554 | | 0.3044 | | 0.809779 | | FALSE | 0.5 | TRUE | Amp | N |
| A2 | APEX2 | SYBR | None | 23.092 | 4.3834 | | 0.6484 | | 0.637987 | | FALSE | 0.5 | TRUE | Amp | N |
| A3 | ATM | SYBR | None | 23.279 | 4.5704 | | -0.5736 | | 1.488233 | | FALSE | 0.5 | TRUE | Amp | N |
| A4 | ATR | SYBR | None | 22.847 | 4.1384 | | -0.3676 | | 1.290205 | | FALSE | 0.5 | TRUE | Amp | N |
| A5 | ATXN3 | SYBR | None | 20.534 | 1.8254 | | -0.3946 | | 1.314578 | | FALSE | 0.5 | TRUE | Amp | N |
| A6 | BRCA1 | SYBR | None | 21.518 | 2.8094 | | -0.4386 | | 1.355289 | | FALSE | 0.5 | TRUE | Amp | N |
| A7 | BRCA2 | SYBR | None | 18.802 | 0.0934 | | -0.3506 | | 1.275091 | | FALSE | 0.5 | TRUE | Amp | N |
| A8 | BRIP1 | SYBR | None | 23.374 | 4.6654 | | -0.0686 | | 1.048699 | | FALSE | 0.5 | TRUE | Amp | N |
| A9 | CCNH | SYBR | None | 25.758 | 7.0494 | | 0.5004 | | 0.706911 | | FALSE | 0.5 | TRUE | Amp | N |
| A10 | CCNO | SYBR | None | 22.873 | 4.1644 | | -0.0746 | | 1.053069 | | FALSE | 0.5 | TRUE | Amp | N |
| A11 | CDK7 | SYBR | None | 15.063 | -3.6456 | | 0.5054 | | 0.704465 | | FALSE | 0.5 | TRUE | Amp | N |
| A12 | DDB1 | SYBR | None | 20.709 | 2.0004 | | -0.3566 | | 1.280405 | | FALSE | 0.5 | TRUE | Amp | N |
| B1 | DDB2 | SYBR | None | 23.561 | 4.8524 | | -0.0426 | | 1.029968 | | FALSE | 0.5 | TRUE | Amp | N |
| B2 | DMC1 | SYBR | None | 27.36 | 8.6514 | | -0.4216 | | 1.339412 | | FALSE | 0.5 | TRUE | Amp | N |
| B3 | ERCC1 | SYBR | None | 22.497 | 3.7884 | | -0.0556 | | 1.039291 | | FALSE | 0.5 | TRUE | Amp | N |
| B4 | ERCC2 | SYBR | None | 25.121 | 6.4124 | | 0.6944 | | 0.617966 | | FALSE | 0.5 | TRUE | Amp | N |
| B5 | ERCC3 | SYBR | None | 21.414 | 2.7054 | | -0.5046 | | 1.41873 | | FALSE | 0.5 | TRUE | Amp | N |
| B6 | ERCC4 | SYBR | None | 27.998 | 9.2894 | | -0.0746 | | 1.053069 | | FALSE | 0.5 | TRUE | Amp | N |
| B7 | ERCC5 | SYBR | None | 24.975 | 6.2664 | | -0.4246 | | 1.3422 | | FALSE | 0.5 | TRUE | Amp | N |
| B8 | ERCC6 | SYBR | None | 18.75 | 0.0414 | | 0.5434 | | 0.686152 | | FALSE | 0.5 | TRUE | Amp | N |
| B9 | ERCC8 | SYBR | None | 22.659 | 3.9504 | | -0.4266 | | 1.344062 | | FALSE | 0.5 | TRUE | Amp | N |
| B10 | EXO1 | SYBR | None | 21.136 | 2.4274 | | 0.5014 | | 0.706421 | | FALSE | 0.5 | TRUE | Amp | N |
| B11 | FEN1 | SYBR | None | 17.908 | -0.8006 | | -0.1206 | | 1.087187 | | FALSE | 0.5 | TRUE | Amp | N |
| B12 | LIG1 | SYBR | None | 22.031 | 3.3224 | | -0.3936 | | 1.313667 | | FALSE | 0.5 | TRUE | Amp | N |
| C1 | LIG3 | SYBR | None | 25.72 | 7.0114 | | -0.1016 | | 1.072963 | | FALSE | 0.5 | TRUE | Amp | N |
| C2 | LIG4 | SYBR | None | 26.286 | 7.5774 | | 0.7444 | | 0.596916 | | FALSE | 0.5 | TRUE | Amp | N |
| C3 | MGMT | SYBR | None | 21.409 | 2.7004 | | -0.4636 | | 1.378979 | | FALSE | 0.5 | TRUE | Amp | N |
| C4 | MLH1 | SYBR | None | 22.645 | 3.9364 | | -0.0196 | | 1.013678 | | FALSE | 0.5 | TRUE | Amp | N |
| C5 | MLH3 | SYBR | None | 25.456 | 6.7474 | | 0.0004 | | 0.999723 | | FALSE | 0.5 | TRUE | Amp | N |
| C6 | MMS19 | SYBR | None | 26.785 | 8.0764 | | 0.6924 | | 0.618824 | | FALSE | 0.5 | TRUE | Amp | N |
| C7 | MPG | SYBR | None | 21.709 | 3.0004 | | -0.0726 | | 1.05161 | | FALSE | 0.5 | TRUE | Amp | N |
| C8 | MRE11A | SYBR | None | 19.181 | 0.4724 | | 0.5364 | | 0.689489 | | FALSE | 0.5 | TRUE | Amp | N |
| C9 | MSH2 | SYBR | None | 16.662 | -2.0466 | | -0.4156 | | 1.333853 | | FALSE | 0.5 | TRUE | Amp | N |
| C10 | MSH3 | SYBR | None | 19.644 | 0.9354 | | -0.4236 | | 1.34127 | | FALSE | 0.5 | TRUE | Amp | N |
| C11 | MSH4 | SYBR | None | 20.845 | 2.1364 | | -0.3956 | | 1.31549 | | FALSE | 0.5 | TRUE | Amp | N |
| C12 | MSH5 | SYBR | None | 28.96 | 10.2514 | | -0.1046 | | 1.075196 | | FALSE | 0.5 | TRUE | Amp | N |
| D1 | MSH6 | SYBR | None | 21.082 | 2.3734 | | -0.0286 | | 1.020022 | | FALSE | 0.5 | TRUE | Amp | N |
| D2 | MUTYH | SYBR | None | 23.599 | 4.8904 | | -0.5216 | | 1.435546 | | FALSE | 0.5 | TRUE | Amp | N |
| D3 | NEIL1 | SYBR | None | 28.137 | 9.4284 | | 0.8054 | | 0.572203 | | FALSE | 0.5 | TRUE | Amp | N |
| D4 | NEIL2 | SYBR | None | 23.903 | 5.1944 | | -0.3766 | | 1.298279 | | FALSE | 0.5 | TRUE | Amp | N |
| D5 | NEIL3 | SYBR | None | 21.525 | 2.8164 | | -0.0476 | | 1.033544 | | FALSE | 0.5 | TRUE | Amp | N |
| D6 | NTHL1 | SYBR | None | 20.958 | 2.2494 | | -0.3486 | | 1.273324 | | FALSE | 0.5 | TRUE | Amp | N |
| D7 | OGG1 | SYBR | None | 22.213 | 3.5044 | | -0.0926 | | 1.06629 | | FALSE | 0.5 | TRUE | Amp | N |
| D8 | PARP1 | SYBR | None | 20.985 | 2.2764 | | -0.3996 | | 1.319142 | | FALSE | 0.5 | TRUE | Amp | N |
| D9 | PARP2 | SYBR | None | 21.312 | 2.6034 | | -0.1096 | | 1.078929 | | FALSE | 0.5 | TRUE | Amp | N |
| D10 | PARP3 | SYBR | None | 24.337 | 5.6284 | | -0.1026 | | 1.073707 | | FALSE | 0.5 | TRUE | Amp | N |
| D11 | PMS1 | SYBR | None | 19.17 | 0.4614 | | 0.5574 | | 0.679526 | | FALSE | 0.5 | TRUE | Amp | N |
| D12 | PMS2 | SYBR | None | 23.182 | 4.4734 | | 0.6314 | | 0.64555 | | FALSE | 0.5 | TRUE | Amp | N |
| E1 | PNKP | SYBR | None | 25.618 | 6.9094 | | 0.6884 | | 0.620542 | | FALSE | 0.5 | TRUE | Amp | N |
| E2 | POLB | SYBR | None | 18.057 | -0.6516 | | -0.0066 | | 1.004585 | | FALSE | 0.5 | TRUE | Amp | N |
| E3 | POLD3 | SYBR | None | 20.769 | 2.0604 | | 0.2954 | | 0.814846 | | FALSE | 0.5 | TRUE | Amp | N |
| E4 | POLL | SYBR | None | 25.136 | 6.4274 | | -0.0896 | | 1.064075 | | FALSE | 0.5 | TRUE | Amp | N |
| E5 | PRKDC | SYBR | None | 24.051 | 5.3424 | | -0.1166 | | 1.084177 | | FALSE | 0.5 | TRUE | Amp | N |
| E6 | RAD18 | SYBR | None | 18.874 | 0.1654 | | 0.5974 | | 0.660944 | | FALSE | 0.5 | TRUE | Amp | N |
| E7 | RAD21 | SYBR | None | 18.748 | 0.0394 | | -0.0956 | | 1.06851 | | FALSE | 0.5 | TRUE | Amp | N |
| E8 | RAD23A | SYBR | None | 23.465 | 4.7564 | | 0.6234 | | 0.649139 | | FALSE | 0.5 | TRUE | Amp | N |
| E9 | RAD23B | SYBR | None | 25.7 | 6.9914 | | 0.6974 | | 0.616683 | | FALSE | 0.5 | TRUE | Amp | N |
| E10 | RAD50 | SYBR | None | 19.766 | 1.0574 | | -0.5586 | | 1.472839 | | FALSE | 0.5 | TRUE | Amp | N |
| E11 | RAD51 | SYBR | None | 22.027 | 3.3184 | | 0.2774 | | 0.825077 | | FALSE | 0.5 | TRUE | Amp | N |
| E12 | RAD51B | SYBR | None | 20.16 | 1.4514 | | -0.3706 | | 1.29289 | | FALSE | 0.5 | TRUE | Amp | N |
| F1 | RAD51C | SYBR | None | 19.414 | 0.7054 | | -0.0016 | | 1.00111 | | FALSE | 0.5 | TRUE | Amp | N |
| F2 | RAD51D | SYBR | None | 24.757 | 6.0484 | | 0.5264 | | 0.694285 | | FALSE | 0.5 | TRUE | Amp | N |
| F3 | RAD52 | SYBR | None | 26.176 | 7.4674 | | -0.1326 | | 1.096268 | | FALSE | 0.5 | TRUE | Amp | N |
| F4 | RAD54L | SYBR | None | 22.698 | 3.9894 | | -0.4046 | | 1.323722 | | FALSE | 0.5 | TRUE | Amp | N |
| F5 | RFC1 | SYBR | None | 24.19 | 5.4814 | | -0.0736 | | 1.052339 | | FALSE | 0.5 | TRUE | Amp | N |
| F6 | RPA1 | SYBR | None | 18.38 | -0.3286 | | 0.5854 | | 0.666465 | | FALSE | 0.5 | TRUE | Amp | N |
| F7 | RPA3 | SYBR | None | 23.932 | 5.2234 | | -0.1216 | | 1.087941 | | FALSE | 0.5 | TRUE | Amp | N |
| F8 | SLK | SYBR | None | 21.894 | 3.1854 | | 0.6264 | | 0.647791 | | FALSE | 0.5 | TRUE | Amp | N |
| F9 | SMUG1 | SYBR | None | 27.884 | 9.1754 | | 0.7024 | | 0.614549 | | FALSE | 0.5 | TRUE | Amp | N |
| F10 | TDG | SYBR | None | 21.867 | 3.1584 | | -0.4426 | | 1.359051 | | FALSE | 0.5 | TRUE | Amp | N |
| F11 | TOP3A | SYBR | None | 26.108 | 7.3994 | | 0.7154 | | 0.609036 | | FALSE | 0.5 | TRUE | Amp | N |
| F12 | TOP3B | SYBR | None | 28.773 | 10.0644 | | 0.8144 | | 0.568645 | | FALSE | 0.5 | TRUE | Amp | N |
| G1 | TREX1 | SYBR | None | 27.699 | 8.9904 | | -0.6436 | | 1.562223 | | FALSE | 0.5 | TRUE | Amp | N |
| G2 | UNG | SYBR | None | 20.243 | 1.5344 | | -0.0156 | | 1.010872 | | FALSE | 0.5 | TRUE | Amp | N |
| G3 | XAB2 | SYBR | None | 23.732 | 5.0234 | | -0.3706 | | 1.29289 | | FALSE | 0.5 | TRUE | Amp | N |
| G4 | XPA | SYBR | None | 31.671 | 12.9624 | | -0.0916 | | 1.065551 | | FALSE | 0.5 | TRUE | Amp | N |
| G5 | XPC | SYBR | None | 26.139 | 7.4304 | | 0.3294 | | 0.795867 | | FALSE | 0.5 | TRUE | Amp | N |
| G6 | XRCC1 | SYBR | None | 20.611 | 1.9024 | | -0.3756 | | 1.297379 | | FALSE | 0.5 | TRUE | Amp | N |
| G7 | XRCC2 | SYBR | None | 22.026 | 3.3174 | | -0.0846 | | 1.060394 | | FALSE | 0.5 | TRUE | Amp | N |
| G8 | XRCC3 | SYBR | None | 24.874 | 6.1654 | | 0.7124 | | 0.610304 | | FALSE | 0.5 | TRUE | Amp | N |
| G9 | XRCC4 | SYBR | None | 25.756 | 7.0474 | | 0.3574 | | 0.78057 | | FALSE | 0.5 | TRUE | Amp | N |
| G10 | XRCC5 | SYBR | None | 18.465 | -0.2436 | | 0.5754 | | 0.6711 | | FALSE | 0.5 | TRUE | Amp | N |
| G11 | XRCC6 | SYBR | None | 20.183 | 1.4744 | | 0.3684 | | 0.774641 | | FALSE | 0.5 | TRUE | Amp | N |
| G12 | XRCC6BP1 | SYBR | None | 23.806 | 5.0974 | | -0.4366 | | 1.353411 | | FALSE | 0.5 | TRUE | Amp | N |
| H1 | ACTB | SYBR | None | 18.836 |  | |  | |  | | FALSE | 0.5 | TRUE | Amp | N |
| H2 | B2M | SYBR | None | 16.962 |  | |  | |  | | FALSE | 0.5 | TRUE | Amp | N |
| H3 | GAPDH | SYBR | None | 21.07 |  | |  | |  | | FALSE | 0.5 | TRUE | Amp | N |
| H4 | HPRT1 | SYBR | None | 20.951 |  | |  | |  | | FALSE | 0.5 | TRUE | Amp | N |
| H5 | RPLP0 | SYBR | None | 15.724 |  | |  | |  | | FALSE | 0.5 | TRUE | Amp | N |
| POI 17 |  |  |  |  |  | |  | |  | |  |  |  |  |  |
| Well Position | Gene Symbol | Reporter | Quencher | CT | Delta Ct | | Delta Delta Ct | | 2^(-Delta Delta Ct) | | Automatic Ct Threshold | Ct Threshold | Automatic Baseline | Amp Status | MTP |
| A1 | APEX1 | SYBR | None | 18.747 | 0.4696 | | 0.5186 | | 0.698049 | | FALSE | 0.5 | TRUE | Amp | N |
| A2 | APEX2 | SYBR | None | 22.608 | 4.3306 | | 0.5956 | | 0.661769 | | FALSE | 0.5 | TRUE | Amp | N |
| A3 | ATM | SYBR | None | 23.957 | 5.6796 | | 0.5356 | | 0.689872 | | FALSE | 0.5 | TRUE | Amp | N |
| A4 | ATR | SYBR | None | 22.406 | 4.1286 | | -0.3774 | | 1.298999 | | FALSE | 0.5 | TRUE | Amp | N |
| A5 | ATXN3 | SYBR | None | 20.732 | 2.4546 | | 0.2346 | | 0.849921 | | FALSE | 0.5 | TRUE | Amp | N |
| A6 | BRCA1 | SYBR | None | 21.781 | 3.5036 | | 0.2556 | | 0.837639 | | FALSE | 0.5 | TRUE | Amp | N |
| A7 | BRCA2 | SYBR | None | 18.22 | -0.0574 | | -0.5014 | | 1.415587 | | FALSE | 0.5 | TRUE | Amp | N |
| A8 | BRIP1 | SYBR | None | 23.312 | 5.0346 | | 0.3006 | | 0.811915 | | FALSE | 0.5 | TRUE | Amp | N |
| A9 | CCNH | SYBR | None | 24.385 | 6.1076 | | -0.4414 | | 1.357921 | | FALSE | 0.5 | TRUE | Amp | N |
| A10 | CCNO | SYBR | None | 22.737 | 4.4596 | | 0.2206 | | 0.858208 | | FALSE | 0.5 | TRUE | Amp | N |
| A11 | CDK7 | SYBR | None | 14.606 | -3.6714 | | 0.4796 | | 0.717176 | | FALSE | 0.5 | TRUE | Amp | N |
| A12 | DDB1 | SYBR | None | 20.598 | 2.3206 | | -0.0364 | | 1.025552 | | FALSE | 0.5 | TRUE | Amp | N |
| B1 | DDB2 | SYBR | None | 23.511 | 5.2336 | | 0.3386 | | 0.790808 | | FALSE | 0.5 | TRUE | Amp | N |
| B2 | DMC1 | SYBR | None | 27.304 | 9.0266 | | -0.0464 | | 1.032685 | | FALSE | 0.5 | TRUE | Amp | N |
| B3 | ERCC1 | SYBR | None | 22.592 | 4.3146 | | 0.4706 | | 0.721664 | | FALSE | 0.5 | TRUE | Amp | N |
| B4 | ERCC2 | SYBR | None | 24.223 | 5.9456 | | 0.2276 | | 0.854054 | | FALSE | 0.5 | TRUE | Amp | N |
| B5 | ERCC3 | SYBR | None | 21.925 | 3.6476 | | 0.4376 | | 0.738362 | | FALSE | 0.5 | TRUE | Amp | N |
| B6 | ERCC4 | SYBR | None | 28.197 | 9.9196 | | 0.5556 | | 0.680374 | | FALSE | 0.5 | TRUE | Amp | N |
| B7 | ERCC5 | SYBR | None | 25.256 | 6.9786 | | 0.2876 | | 0.819264 | | FALSE | 0.5 | TRUE | Amp | N |
| B8 | ERCC6 | SYBR | None | 18.275 | -0.0024 | | 0.4996 | | 0.707303 | | FALSE | 0.5 | TRUE | Amp | N |
| B9 | ERCC8 | SYBR | None | 22.336 | 4.0586 | | -0.3184 | | 1.246947 | | FALSE | 0.5 | TRUE | Amp | N |
| B10 | EXO1 | SYBR | None | 20.781 | 2.5036 | | 0.5776 | | 0.670078 | | FALSE | 0.5 | TRUE | Amp | N |
| B11 | FEN1 | SYBR | None | 17.832 | -0.4454 | | 0.2346 | | 0.849921 | | FALSE | 0.5 | TRUE | Amp | N |
| B12 | LIG1 | SYBR | None | 22.299 | 4.0216 | | 0.3056 | | 0.809106 | | FALSE | 0.5 | TRUE | Amp | N |
| C1 | LIG3 | SYBR | None | 25.724 | 7.4466 | | 0.3336 | | 0.793554 | | FALSE | 0.5 | TRUE | Amp | N |
| C2 | LIG4 | SYBR | None | 25.35 | 7.0726 | | 0.2396 | | 0.84698 | | FALSE | 0.5 | TRUE | Amp | N |
| C3 | MGMT | SYBR | None | 20.988 | 2.7106 | | -0.4534 | | 1.369263 | | FALSE | 0.5 | TRUE | Amp | N |
| C4 | MLH1 | SYBR | None | 22.171 | 3.8936 | | -0.0624 | | 1.044201 | | FALSE | 0.5 | TRUE | Amp | N |
| C5 | MLH3 | SYBR | None | 25.023 | 6.7456 | | -0.0014 | | 1.000971 | | FALSE | 0.5 | TRUE | Amp | N |
| C6 | MMS19 | SYBR | None | 25.197 | 6.9196 | | -0.4644 | | 1.379743 | | FALSE | 0.5 | TRUE | Amp | N |
| C7 | MPG | SYBR | None | 21.276 | 2.9986 | | -0.0744 | | 1.052923 | | FALSE | 0.5 | TRUE | Amp | N |
| C8 | MRE11A | SYBR | None | 17.928 | -0.3494 | | -0.2854 | | 1.218748 | | FALSE | 0.5 | TRUE | Amp | N |
| C9 | MSH2 | SYBR | None | 16.178 | -2.0994 | | -0.4684 | | 1.383574 | | FALSE | 0.5 | TRUE | Amp | N |
| C10 | MSH3 | SYBR | None | 19.547 | 1.2696 | | -0.0894 | | 1.063928 | | FALSE | 0.5 | TRUE | Amp | N |
| C11 | MSH4 | SYBR | None | 20.326 | 2.0486 | | -0.4834 | | 1.398035 | | FALSE | 0.5 | TRUE | Amp | N |
| C12 | MSH5 | SYBR | None | 28.948 | 10.6706 | | 0.3146 | | 0.804074 | | FALSE | 0.5 | TRUE | Amp | N |
| D1 | MSH6 | SYBR | None | 20.313 | 2.0356 | | -0.3664 | | 1.289132 | | FALSE | 0.5 | TRUE | Amp | N |
| D2 | MUTYH | SYBR | None | 23.208 | 4.9306 | | -0.4814 | | 1.396098 | | FALSE | 0.5 | TRUE | Amp | N |
| D3 | NEIL1 | SYBR | None | 27.638 | 9.3606 | | 0.7376 | | 0.599736 | | FALSE | 0.5 | TRUE | Amp | N |
| D4 | NEIL2 | SYBR | None | 23.764 | 5.4866 | | -0.0844 | | 1.060247 | | FALSE | 0.5 | TRUE | Amp | N |
| D5 | NEIL3 | SYBR | None | 21.732 | 3.4546 | | 0.5906 | | 0.664067 | | FALSE | 0.5 | TRUE | Amp | N |
| D6 | NTHL1 | SYBR | None | 21.137 | 2.8596 | | 0.2616 | | 0.834162 | | FALSE | 0.5 | TRUE | Amp | N |
| D7 | OGG1 | SYBR | None | 21.828 | 3.5506 | | -0.0464 | | 1.032685 | | FALSE | 0.5 | TRUE | Amp | N |
| D8 | PARP1 | SYBR | None | 21.564 | 3.2866 | | 0.6106 | | 0.654924 | | FALSE | 0.5 | TRUE | Amp | N |
| D9 | PARP2 | SYBR | None | 21.276 | 2.9986 | | 0.2856 | | 0.8204 | | FALSE | 0.5 | TRUE | Amp | N |
| D10 | PARP3 | SYBR | None | 24.587 | 6.3096 | | 0.5786 | | 0.669613 | | FALSE | 0.5 | TRUE | Amp | N |
| D11 | PMS1 | SYBR | None | 18.733 | 0.4556 | | 0.5516 | | 0.682263 | | FALSE | 0.5 | TRUE | Amp | N |
| D12 | PMS2 | SYBR | None | 22.733 | 4.4556 | | 0.6136 | | 0.653564 | | FALSE | 0.5 | TRUE | Amp | N |
| E1 | PNKP | SYBR | None | 25.166 | 6.8886 | | 0.6676 | | 0.629553 | | FALSE | 0.5 | TRUE | Amp | N |
| E2 | POLB | SYBR | None | 17.565 | -0.7124 | | -0.0674 | | 1.047827 | | FALSE | 0.5 | TRUE | Amp | N |
| E3 | POLD3 | SYBR | None | 20.003 | 1.7256 | | -0.0394 | | 1.027686 | | FALSE | 0.5 | TRUE | Amp | N |
| E4 | POLL | SYBR | None | 24.748 | 6.4706 | | -0.0464 | | 1.032685 | | FALSE | 0.5 | TRUE | Amp | N |
| E5 | PRKDC | SYBR | None | 23.385 | 5.1076 | | -0.3514 | | 1.275798 | | FALSE | 0.5 | TRUE | Amp | N |
| E6 | RAD18 | SYBR | None | 17.334 | -0.9434 | | -0.5114 | | 1.425433 | | FALSE | 0.5 | TRUE | Amp | N |
| E7 | RAD21 | SYBR | None | 18.058 | -0.2194 | | -0.3544 | | 1.278454 | | FALSE | 0.5 | TRUE | Amp | N |
| E8 | RAD23A | SYBR | None | 22.055 | 3.7776 | | -0.3554 | | 1.27934 | | FALSE | 0.5 | TRUE | Amp | N |
| E9 | RAD23B | SYBR | None | 24.514 | 6.2366 | | -0.0574 | | 1.040589 | | FALSE | 0.5 | TRUE | Amp | N |
| E10 | RAD50 | SYBR | None | 19.415 | 1.1376 | | -0.4784 | | 1.393198 | | FALSE | 0.5 | TRUE | Amp | N |
| E11 | RAD51 | SYBR | None | 21.311 | 3.0336 | | -0.0074 | | 1.005142 | | FALSE | 0.5 | TRUE | Amp | N |
| E12 | RAD51B | SYBR | None | 20.67 | 2.3926 | | 0.5706 | | 0.673337 | | FALSE | 0.5 | TRUE | Amp | N |
| F1 | RAD51C | SYBR | None | 18.649 | 0.3716 | | -0.3354 | | 1.261727 | | FALSE | 0.5 | TRUE | Amp | N |
| F2 | RAD51D | SYBR | None | 23.793 | 5.5156 | | -0.0064 | | 1.004446 | | FALSE | 0.5 | TRUE | Amp | N |
| F3 | RAD52 | SYBR | None | 25.376 | 7.0986 | | -0.5014 | | 1.415587 | | FALSE | 0.5 | TRUE | Amp | N |
| F4 | RAD54L | SYBR | None | 22.581 | 4.3036 | | -0.0904 | | 1.064665 | | FALSE | 0.5 | TRUE | Amp | N |
| F5 | RFC1 | SYBR | None | 23.809 | 5.5316 | | -0.0234 | | 1.016352 | | FALSE | 0.5 | TRUE | Amp | N |
| F6 | RPA1 | SYBR | None | 17.345 | -0.9324 | | -0.0184 | | 1.012836 | | FALSE | 0.5 | TRUE | Amp | N |
| F7 | RPA3 | SYBR | None | 23.293 | 5.0156 | | -0.3294 | | 1.256491 | | FALSE | 0.5 | TRUE | Amp | N |
| F8 | SLK | SYBR | None | 20.452 | 2.1746 | | -0.3844 | | 1.305317 | | FALSE | 0.5 | TRUE | Amp | N |
| F9 | SMUG1 | SYBR | None | 26.707 | 8.4296 | | -0.0434 | | 1.03054 | | FALSE | 0.5 | TRUE | Amp | N |
| F10 | TDG | SYBR | None | 21.557 | 3.2796 | | -0.3214 | | 1.249543 | | FALSE | 0.5 | TRUE | Amp | N |
| F11 | TOP3A | SYBR | None | 25.527 | 7.2496 | | 0.5656 | | 0.675674 | | FALSE | 0.5 | TRUE | Amp | N |
| F12 | TOP3B | SYBR | None | 28.134 | 9.8566 | | 0.6066 | | 0.656743 | | FALSE | 0.5 | TRUE | Amp | N |
| G1 | TREX1 | SYBR | None | 28.666 | 10.3886 | | 0.7546 | | 0.592711 | | FALSE | 0.5 | TRUE | Amp | N |
| G2 | UNG | SYBR | None | 19.463 | 1.1856 | | -0.3644 | | 1.287346 | | FALSE | 0.5 | TRUE | Amp | N |
| G3 | XAB2 | SYBR | None | 23.096 | 4.8186 | | -0.5754 | | 1.490091 | | FALSE | 0.5 | TRUE | Amp | N |
| G4 | XPA | SYBR | None | 31.573 | 13.2956 | | 0.2416 | | 0.845807 | | FALSE | 0.5 | TRUE | Amp | N |
| G5 | XPC | SYBR | None | 25.862 | 7.5846 | | 0.4836 | | 0.715191 | | FALSE | 0.5 | TRUE | Amp | N |
| G6 | XRCC1 | SYBR | None | 20.074 | 1.7966 | | -0.4814 | | 1.396098 | | FALSE | 0.5 | TRUE | Amp | N |
| G7 | XRCC2 | SYBR | None | 23.608 | 5.3306 | | 1.9286 | | 0.262684 | | FALSE | 0.5 | TRUE | Amp | N |
| G8 | XRCC3 | SYBR | None | 24.015 | 5.7376 | | 0.2846 | | 0.820969 | | FALSE | 0.5 | TRUE | Amp | N |
| G9 | XRCC4 | SYBR | None | 25.302 | 7.0246 | | 0.3346 | | 0.793004 | | FALSE | 0.5 | TRUE | Amp | N |
| G10 | XRCC5 | SYBR | None | 17.661 | -0.6164 | | 0.2026 | | 0.868983 | | FALSE | 0.5 | TRUE | Amp | N |
| G11 | XRCC6 | SYBR | None | 18.908 | 0.6306 | | -0.4754 | | 1.390304 | | FALSE | 0.5 | TRUE | Amp | N |
| G12 | XRCC6BP1 | SYBR | None | 23.754 | 5.4766 | | -0.0574 | | 1.040589 | | FALSE | 0.5 | TRUE | Amp | N |
| H1 | ACTB | SYBR | None | 17.993 |  | |  | |  | | FALSE | 0.5 | TRUE | Amp | N |
| H2 | B2M | SYBR | None | 16.812 |  | |  | |  | | FALSE | 0.5 | TRUE | Amp | N |
| H3 | GAPDH | SYBR | None | 20.976 |  | |  | |  | | FALSE | 0.5 | TRUE | Amp | N |
| H4 | HPRT1 | SYBR | None | 20.489 |  | |  | |  | | FALSE | 0.5 | TRUE | Amp | N |
| H5 | RPLP0 | SYBR | None | 15.117 |  | |  | |  | | FALSE | 0.5 | TRUE | Amp | N |
| POI 18 | Failed in the test of single cell library assessment (GAPDH quality control before running array). | | | | | | | | | | | | |  |  |
| POI 19 |  |  |  |  |  | |  | |  | |  |  |  |  |  |
| Well Position | Gene Symbol | Reporter | Quencher | CT | Delta Ct | | Delta Delta Ct | | 2^(-Delta Delta Ct) | | Automatic Ct Threshold | Ct Threshold | Automatic Baseline | Amp Status | MTP |
| A1 | APEX1 | SYBR | None | 18.662 | 0.3248 | | 0.3738 | | 0.771747 | | FALSE | 0.5 | TRUE | Amp | N |
| A2 | APEX2 | SYBR | None | 22.577 | 4.2398 | | 0.5048 | | 0.704758 | | FALSE | 0.5 | TRUE | Amp | N |
| A3 | ATM | SYBR | None | 23.743 | 5.4058 | | 0.2618 | | 0.834047 | | FALSE | 0.5 | TRUE | Amp | N |
| A4 | ATR | SYBR | None | 22.348 | 4.0108 | | -0.4952 | | 1.409516 | | FALSE | 0.5 | TRUE | Amp | N |
| A5 | ATXN3 | SYBR | None | 20.19 | 1.8528 | | -0.3672 | | 1.289847 | | FALSE | 0.5 | TRUE | Amp | N |
| A6 | BRCA1 | SYBR | None | 21.092 | 2.7548 | | -0.4932 | | 1.407563 | | FALSE | 0.5 | TRUE | Amp | N |
| A7 | BRCA2 | SYBR | None | 18.363 | 0.0258 | | -0.4182 | | 1.336259 | | FALSE | 0.5 | TRUE | Amp | N |
| A8 | BRIP1 | SYBR | None | 22.609 | 4.2718 | | -0.4622 | | 1.377641 | | FALSE | 0.5 | TRUE | Amp | N |
| A9 | CCNH | SYBR | None | 24.73 | 6.3928 | | -0.1562 | | 1.114348 | | FALSE | 0.5 | TRUE | Amp | N |
| A10 | CCNO | SYBR | None | 23.136 | 4.7988 | | 0.5598 | | 0.678396 | | FALSE | 0.5 | TRUE | Amp | N |
| A11 | CDK7 | SYBR | None | 14.454 | -3.8832 | | 0.2678 | | 0.830585 | | FALSE | 0.5 | TRUE | Amp | N |
| A12 | DDB1 | SYBR | None | 21.243 | 2.9058 | | 0.5488 | | 0.683588 | | FALSE | 0.5 | TRUE | Amp | N |
| B1 | DDB2 | SYBR | None | 23.563 | 5.2258 | | 0.3308 | | 0.795095 | | FALSE | 0.5 | TRUE | Amp | N |
| B2 | DMC1 | SYBR | None | 28.159 | 9.8218 | | 0.7488 | | 0.595098 | | FALSE | 0.5 | TRUE | Amp | N |
| B3 | ERCC1 | SYBR | None | 22.128 | 3.7908 | | -0.0532 | | 1.037564 | | FALSE | 0.5 | TRUE | Amp | N |
| B4 | ERCC2 | SYBR | None | 23.894 | 5.5568 | | -0.1612 | | 1.118217 | | FALSE | 0.5 | TRUE | Amp | N |
| B5 | ERCC3 | SYBR | None | 21.112 | 2.7748 | | -0.4352 | | 1.352098 | | FALSE | 0.5 | TRUE | Amp | N |
| B6 | ERCC4 | SYBR | None | 27.144 | 8.8068 | | -0.5572 | | 1.471411 | | FALSE | 0.5 | TRUE | Amp | N |
| B7 | ERCC5 | SYBR | None | 24.593 | 6.2558 | | -0.4352 | | 1.352098 | | FALSE | 0.5 | TRUE | Amp | N |
| B8 | ERCC6 | SYBR | None | 18.3 | -0.0372 | | 0.4648 | | 0.724572 | | FALSE | 0.5 | TRUE | Amp | N |
| B9 | ERCC8 | SYBR | None | 22.58 | 4.2428 | | -0.1342 | | 1.097484 | | FALSE | 0.5 | TRUE | Amp | N |
| B10 | EXO1 | SYBR | None | 20.523 | 2.1858 | | 0.2598 | | 0.835204 | | FALSE | 0.5 | TRUE | Amp | N |
| B11 | FEN1 | SYBR | None | 18.189 | -0.1482 | | 0.5318 | | 0.691691 | | FALSE | 0.5 | TRUE | Amp | N |
| B12 | LIG1 | SYBR | None | 21.894 | 3.5568 | | -0.1592 | | 1.116668 | | FALSE | 0.5 | TRUE | Amp | N |
| C1 | LIG3 | SYBR | None | 24.981 | 6.6438 | | -0.4692 | | 1.384342 | | FALSE | 0.5 | TRUE | Amp | N |
| C2 | LIG4 | SYBR | None | 25.884 | 7.5468 | | 0.7138 | | 0.609712 | | FALSE | 0.5 | TRUE | Amp | N |
| C3 | MGMT | SYBR | None | 22.156 | 3.8188 | | 0.6548 | | 0.635164 | | FALSE | 0.5 | TRUE | Amp | N |
| C4 | MLH1 | SYBR | None | 23 | 4.6628 | | 0.7068 | | 0.612678 | | FALSE | 0.5 | TRUE | Amp | N |
| C5 | MLH3 | SYBR | None | 24.986 | 6.6488 | | -0.0982 | | 1.070437 | | FALSE | 0.5 | TRUE | Amp | N |
| C6 | MMS19 | SYBR | None | 25.201 | 6.8638 | | -0.5202 | | 1.434154 | | FALSE | 0.5 | TRUE | Amp | N |
| C7 | MPG | SYBR | None | 21.002 | 2.6648 | | -0.4082 | | 1.327029 | | FALSE | 0.5 | TRUE | Amp | N |
| C8 | MRE11A | SYBR | None | 17.861 | -0.4762 | | -0.4122 | | 1.330714 | | FALSE | 0.5 | TRUE | Amp | N |
| C9 | MSH2 | SYBR | None | 16.561 | -1.7762 | | -0.1452 | | 1.105884 | | FALSE | 0.5 | TRUE | Amp | N |
| C10 | MSH3 | SYBR | None | 19.138 | 0.8008 | | -0.5582 | | 1.472431 | | FALSE | 0.5 | TRUE | Amp | N |
| C11 | MSH4 | SYBR | None | 21.306 | 2.9688 | | 0.4368 | | 0.738771 | | FALSE | 0.5 | TRUE | Amp | N |
| C12 | MSH5 | SYBR | None | 28.126 | 9.7888 | | -0.5672 | | 1.481645 | | FALSE | 0.5 | TRUE | Amp | N |
| D1 | MSH6 | SYBR | None | 21.012 | 2.6748 | | 0.2728 | | 0.827712 | | FALSE | 0.5 | TRUE | Amp | N |
| D2 | MUTYH | SYBR | None | 23.191 | 4.8538 | | -0.5582 | | 1.472431 | | FALSE | 0.5 | TRUE | Amp | N |
| D3 | NEIL1 | SYBR | None | 26.412 | 8.0748 | | -0.5482 | | 1.46226 | | FALSE | 0.5 | TRUE | Amp | N |
| D4 | NEIL2 | SYBR | None | 23.801 | 5.4638 | | -0.1072 | | 1.077136 | | FALSE | 0.5 | TRUE | Amp | N |
| D5 | NEIL3 | SYBR | None | 21.131 | 2.7938 | | -0.0702 | | 1.049862 | | FALSE | 0.5 | TRUE | Amp | N |
| D6 | NTHL1 | SYBR | None | 20.864 | 2.5268 | | -0.0712 | | 1.05059 | | FALSE | 0.5 | TRUE | Amp | N |
| D7 | OGG1 | SYBR | None | 22.55 | 4.2128 | | 0.6158 | | 0.652568 | | FALSE | 0.5 | TRUE | Amp | N |
| D8 | PARP1 | SYBR | None | 21.603 | 3.2658 | | 0.5898 | | 0.664435 | | FALSE | 0.5 | TRUE | Amp | N |
| D9 | PARP2 | SYBR | None | 20.891 | 2.5538 | | -0.1592 | | 1.116668 | | FALSE | 0.5 | TRUE | Amp | N |
| D10 | PARP3 | SYBR | None | 23.972 | 5.6348 | | -0.0962 | | 1.068954 | | FALSE | 0.5 | TRUE | Amp | N |
| D11 | PMS1 | SYBR | None | 18.134 | -0.2032 | | -0.1072 | | 1.077136 | | FALSE | 0.5 | TRUE | Amp | N |
| D12 | PMS2 | SYBR | None | 22.863 | 4.5258 | | 0.6838 | | 0.622523 | | FALSE | 0.5 | TRUE | Amp | N |
| E1 | PNKP | SYBR | None | 24.115 | 5.7778 | | -0.4432 | | 1.359617 | | FALSE | 0.5 | TRUE | Amp | N |
| E2 | POLB | SYBR | None | 17.247 | -1.0902 | | -0.4452 | | 1.361503 | | FALSE | 0.5 | TRUE | Amp | N |
| E3 | POLD3 | SYBR | None | 20.632 | 2.2948 | | 0.5298 | | 0.692651 | | FALSE | 0.5 | TRUE | Amp | N |
| E4 | POLL | SYBR | None | 24.408 | 6.0708 | | -0.4462 | | 1.362447 | | FALSE | 0.5 | TRUE | Amp | N |
| E5 | PRKDC | SYBR | None | 24.425 | 6.0878 | | 0.6288 | | 0.646714 | | FALSE | 0.5 | TRUE | Amp | N |
| E6 | RAD18 | SYBR | None | 17.803 | -0.5342 | | -0.1022 | | 1.073409 | | FALSE | 0.5 | TRUE | Amp | N |
| E7 | RAD21 | SYBR | None | 18.983 | 0.6458 | | 0.5108 | | 0.701833 | | FALSE | 0.5 | TRUE | Amp | N |
| E8 | RAD23A | SYBR | None | 22.328 | 3.9908 | | -0.1422 | | 1.103587 | | FALSE | 0.5 | TRUE | Amp | N |
| E9 | RAD23B | SYBR | None | 24.182 | 5.8448 | | -0.4492 | | 1.365283 | | FALSE | 0.5 | TRUE | Amp | N |
| E10 | RAD50 | SYBR | None | 19.87 | 1.5328 | | -0.0832 | | 1.059365 | | FALSE | 0.5 | TRUE | Amp | N |
| E11 | RAD51 | SYBR | None | 21.977 | 3.6398 | | 0.5988 | | 0.660303 | | FALSE | 0.5 | TRUE | Amp | N |
| E12 | RAD51B | SYBR | None | 20.744 | 2.4068 | | 0.5848 | | 0.666742 | | FALSE | 0.5 | TRUE | Amp | N |
| F1 | RAD51C | SYBR | None | 19.638 | 1.3008 | | 0.5938 | | 0.662595 | | FALSE | 0.5 | TRUE | Amp | N |
| F2 | RAD51D | SYBR | None | 23.338 | 5.0008 | | -0.5212 | | 1.435148 | | FALSE | 0.5 | TRUE | Amp | N |
| F3 | RAD52 | SYBR | None | 26.713 | 8.3758 | | 0.7758 | | 0.584065 | | FALSE | 0.5 | TRUE | Amp | N |
| F4 | RAD54L | SYBR | None | 23.319 | 4.9818 | | 0.5878 | | 0.665357 | | FALSE | 0.5 | TRUE | Amp | N |
| F5 | RFC1 | SYBR | None | 23.744 | 5.4068 | | -0.1482 | | 1.108186 | | FALSE | 0.5 | TRUE | Amp | N |
| F6 | RPA1 | SYBR | None | 17.36 | -0.9772 | | -0.0632 | | 1.044781 | | FALSE | 0.5 | TRUE | Amp | N |
| F7 | RPA3 | SYBR | None | 23.576 | 5.2388 | | -0.1062 | | 1.076389 | | FALSE | 0.5 | TRUE | Amp | N |
| F8 | SLK | SYBR | None | 21.216 | 2.8788 | | 0.3198 | | 0.801181 | | FALSE | 0.5 | TRUE | Amp | N |
| F9 | SMUG1 | SYBR | None | 26.141 | 7.8038 | | -0.6692 | | 1.590191 | | FALSE | 0.5 | TRUE | Amp | N |
| F10 | TDG | SYBR | None | 21.794 | 3.4568 | | -0.1442 | | 1.105118 | | FALSE | 0.5 | TRUE | Amp | N |
| F11 | TOP3A | SYBR | None | 25.762 | 7.4248 | | 0.7408 | | 0.598407 | | FALSE | 0.5 | TRUE | Amp | N |
| F12 | TOP3B | SYBR | None | 27.542 | 9.2048 | | -0.0452 | | 1.031826 | | FALSE | 0.5 | TRUE | Amp | N |
| G1 | TREX1 | SYBR | None | 27.498 | 9.1608 | | -0.4732 | | 1.388185 | | FALSE | 0.5 | TRUE | Amp | N |
| G2 | UNG | SYBR | None | 19.425 | 1.0878 | | -0.4622 | | 1.377641 | | FALSE | 0.5 | TRUE | Amp | N |
| G3 | XAB2 | SYBR | None | 23.273 | 4.9358 | | -0.4582 | | 1.373827 | | FALSE | 0.5 | TRUE | Amp | N |
| G4 | XPA | SYBR | None | 31.24 | 12.9028 | | -0.1512 | | 1.110493 | | FALSE | 0.5 | TRUE | Amp | N |
| G5 | XPC | SYBR | None | 24.967 | 6.6298 | | -0.4712 | | 1.386262 | | FALSE | 0.5 | TRUE | Amp | N |
| G6 | XRCC1 | SYBR | None | 20.554 | 2.2168 | | -0.0612 | | 1.043333 | | FALSE | 0.5 | TRUE | Amp | N |
| G7 | XRCC2 | SYBR | None | 22.265 | 3.9278 | | 0.5258 | | 0.694574 | | FALSE | 0.5 | TRUE | Amp | N |
| G8 | XRCC3 | SYBR | None | 24.231 | 5.8938 | | 0.4408 | | 0.736726 | | FALSE | 0.5 | TRUE | Amp | N |
| G9 | XRCC4 | SYBR | None | 25.626 | 7.2888 | | 0.5988 | | 0.660303 | | FALSE | 0.5 | TRUE | Amp | N |
| G10 | XRCC5 | SYBR | None | 17.165 | -1.1722 | | -0.3532 | | 1.277391 | | FALSE | 0.5 | TRUE | Amp | N |
| G11 | XRCC6 | SYBR | None | 19.96 | 1.6228 | | 0.5168 | | 0.69892 | | FALSE | 0.5 | TRUE | Amp | N |
| G12 | XRCC6BP1 | SYBR | None | 23.752 | 5.4148 | | -0.1192 | | 1.086132 | | FALSE | 0.5 | TRUE | Amp | N |
| H1 | ACTB | SYBR | None | 18.173 |  | |  | |  | | FALSE | 0.5 | TRUE | Amp | N |
| H2 | B2M | SYBR | None | 16.982 |  | |  | |  | | FALSE | 0.5 | TRUE | Amp | N |
| H3 | GAPDH | SYBR | None | 20.736 |  | |  | |  | | FALSE | 0.5 | TRUE | Amp | N |
| H4 | HPRT1 | SYBR | None | 20.904 |  | |  | |  | | FALSE | 0.5 | TRUE | Amp | N |
| H5 | RPLP0 | SYBR | None | 14.891 |  | |  | |  | | FALSE | 0.5 | TRUE | Amp | N |
| POI 20 |  |  |  |  |  | |  | |  | |  |  |  |  |  |
| Well Position | Gene Symbol | Reporter | Quencher | CT | Delta Ct | | Delta Delta Ct | | 2^(-Delta Delta Ct) | | Automatic Ct Threshold | Ct Threshold | Automatic Baseline | Amp Status | MTP |
| A1 | APEX1 | SYBR | None | 18.318 | -0.0672 | | -0.0176 | | 1.012276 | | FALSE | 0.5 | TRUE | Amp | N |
| A2 | APEX2 | SYBR | None | 22.117 | 3.7318 | | -0.00357 | | 1.002479 | | FALSE | 0.5 | TRUE | Amp | N |
| A3 | ATM | SYBR | None | 22.75 | 4.3648 | | -0.77957 | | 1.71662 | | FALSE | 0.5 | TRUE | Amp | N |
| A4 | ATR | SYBR | None | 22.483 | 4.0978 | | -0.40829 | | 1.327113 | | FALSE | 0.5 | TRUE | Amp | N |
| A5 | ATXN3 | SYBR | None | 20.717 | 2.3318 | | 0.111782 | | 0.925444 | | FALSE | 0.5 | TRUE | Amp | N |
| A6 | BRCA1 | SYBR | None | 21.459 | 3.0738 | | -0.1738 | | 1.128027 | | FALSE | 0.5 | TRUE | Amp | N |
| A7 | BRCA2 | SYBR | None | 21.026 | 2.6408 | | 2.19685 | | 0.218113 | | FALSE | 0.5 | TRUE | Amp | N |
| A8 | BRIP1 | SYBR | None | 23.586 | 5.2008 | | 0.467291 | | 0.723321 | | FALSE | 0.5 | TRUE | Amp | N |
| A9 | CCNH | SYBR | None | 24.749 | 6.3638 | | -0.18529 | | 1.137046 | | FALSE | 0.5 | TRUE | Amp | N |
| A10 | CCNO | SYBR | None | 22.159 | 3.7738 | | -0.46495 | | 1.38027 | | FALSE | 0.5 | TRUE | Amp | N |
| A11 | CDK7 | SYBR | None | 14.108 | -4.2772 | | -0.1258 | | 1.091113 | | FALSE | 0.5 | TRUE | Amp | N |
| A12 | DDB1 | SYBR | None | 20.832 | 2.4468 | | 0.089838 | | 0.939628 | | FALSE | 0.5 | TRUE | Amp | N |
| B1 | DDB2 | SYBR | None | 22.822 | 4.4368 | | -0.45817 | | 1.3738 | | FALSE | 0.5 | TRUE | Amp | N |
| B2 | DMC1 | SYBR | None | 27.003 | 8.6178 | | -0.45555 | | 1.37131 | | FALSE | 0.5 | TRUE | Amp | N |
| B3 | ERCC1 | SYBR | None | 22.014 | 3.6288 | | -0.21559 | | 1.161181 | | FALSE | 0.5 | TRUE | Amp | N |
| B4 | ERCC2 | SYBR | None | 24.004 | 5.6188 | | -0.09933 | | 1.071273 | | FALSE | 0.5 | TRUE | Amp | N |
| B5 | ERCC3 | SYBR | None | 22.095 | 3.7098 | | 0.499477 | | 0.707363 | | FALSE | 0.5 | TRUE | Amp | N |
| B6 | ERCC4 | SYBR | None | 27.906 | 9.5208 | | 0.157161 | | 0.896788 | | FALSE | 0.5 | TRUE | Amp | N |
| B7 | ERCC5 | SYBR | None | 24.912 | 6.5268 | | -0.16453 | | 1.120799 | | FALSE | 0.5 | TRUE | Amp | N |
| B8 | ERCC6 | SYBR | None | 18.013 | -0.3722 | | 0.129975 | | 0.913847 | | FALSE | 0.5 | TRUE | Amp | N |
| B9 | ERCC8 | SYBR | None | 22.16 | 3.7748 | | -0.60255 | | 1.518399 | | FALSE | 0.5 | TRUE | Amp | N |
| B10 | EXO1 | SYBR | None | 20.41 | 2.0248 | | 0.09916 | | 0.933576 | | FALSE | 0.5 | TRUE | Amp | N |
| B11 | FEN1 | SYBR | None | 18.069 | -0.3162 | | 0.363551 | | 0.777249 | | FALSE | 0.5 | TRUE | Amp | N |
| B12 | LIG1 | SYBR | None | 21.981 | 3.5958 | | -0.1204 | | 1.087037 | | FALSE | 0.5 | TRUE | Amp | N |
| C1 | LIG3 | SYBR | None | 24.934 | 6.5488 | | -0.56398 | | 1.478341 | | FALSE | 0.5 | TRUE | Amp | N |
| C2 | LIG4 | SYBR | None | 25.002 | 6.6168 | | -0.2161 | | 1.161588 | | FALSE | 0.5 | TRUE | Amp | N |
| C3 | MGMT | SYBR | None | 21.059 | 2.6738 | | -0.4904 | | 1.404832 | | FALSE | 0.5 | TRUE | Amp | N |
| C4 | MLH1 | SYBR | None | 22.175 | 3.7898 | | -0.16627 | | 1.122156 | | FALSE | 0.5 | TRUE | Amp | N |
| C5 | MLH3 | SYBR | None | 25.059 | 6.6738 | | -0.0729 | | 1.051827 | | FALSE | 0.5 | TRUE | Amp | N |
| C6 | MMS19 | SYBR | None | 26.348 | 7.9628 | | 0.578853 | | 0.669496 | | FALSE | 0.5 | TRUE | Amp | N |
| C7 | MPG | SYBR | None | 21.286 | 2.9008 | | -0.17177 | | 1.126441 | | FALSE | 0.5 | TRUE | Amp | N |
| C8 | MRE11A | SYBR | None | 18.403 | 0.0178 | | 0.082177 | | 0.944631 | | FALSE | 0.5 | TRUE | Amp | N |
| C9 | MSH2 | SYBR | None | 16.989 | -1.3962 | | 0.234675 | | 0.849876 | | FALSE | 0.5 | TRUE | Amp | N |
| C10 | MSH3 | SYBR | None | 19.826 | 1.4408 | | 0.081423 | | 0.945125 | | FALSE | 0.5 | TRUE | Amp | N |
| C11 | MSH4 | SYBR | None | 20.902 | 2.5168 | | -0.01461 | | 1.010177 | | FALSE | 0.5 | TRUE | Amp | N |
| C12 | MSH5 | SYBR | None | 28.536 | 10.1508 | | -0.20485 | | 1.152568 | | FALSE | 0.5 | TRUE | Amp | N |
| D1 | MSH6 | SYBR | None | 21.228 | 2.8428 | | 0.441382 | | 0.736429 | | FALSE | 0.5 | TRUE | Amp | N |
| D2 | MUTYH | SYBR | None | 23.334 | 4.9488 | | -0.46285 | | 1.378265 | | FALSE | 0.5 | TRUE | Amp | N |
| D3 | NEIL1 | SYBR | None | 26.991 | 8.6058 | | -0.0174 | | 1.012133 | | FALSE | 0.5 | TRUE | Amp | N |
| D4 | NEIL2 | SYBR | None | 23.837 | 5.4518 | | -0.11881 | | 1.085837 | | FALSE | 0.5 | TRUE | Amp | N |
| D5 | NEIL3 | SYBR | None | 21.16 | 2.7748 | | -0.0887 | | 1.063412 | | FALSE | 0.5 | TRUE | Amp | N |
| D6 | NTHL1 | SYBR | None | 20.56 | 2.1748 | | -0.42354 | | 1.341215 | | FALSE | 0.5 | TRUE | Amp | N |
| D7 | OGG1 | SYBR | None | 22.453 | 4.0678 | | 0.471315 | | 0.721307 | | FALSE | 0.5 | TRUE | Amp | N |
| D8 | PARP1 | SYBR | None | 21.027 | 2.6418 | | -0.03454 | | 1.024227 | | FALSE | 0.5 | TRUE | Amp | N |
| D9 | PARP2 | SYBR | None | 21.541 | 3.1558 | | 0.44254 | | 0.735838 | | FALSE | 0.5 | TRUE | Amp | N |
| D10 | PARP3 | SYBR | None | 24.004 | 5.6188 | | -0.11223 | | 1.0809 | | FALSE | 0.5 | TRUE | Amp | N |
| D11 | PMS1 | SYBR | None | 18.412 | 0.0268 | | 0.122832 | | 0.918383 | | FALSE | 0.5 | TRUE | Amp | N |
| D12 | PMS2 | SYBR | None | 22.006 | 3.6208 | | -0.22063 | | 1.165243 | | FALSE | 0.5 | TRUE | Amp | N |
| E1 | PNKP | SYBR | None | 24.429 | 6.0438 | | -0.17703 | | 1.130553 | | FALSE | 0.5 | TRUE | Amp | N |
| E2 | POLB | SYBR | None | 17.703 | -0.6822 | | -0.03667 | | 1.025741 | | FALSE | 0.5 | TRUE | Amp | N |
| E3 | POLD3 | SYBR | None | 20.095 | 1.7098 | | -0.05506 | | 1.038899 | | FALSE | 0.5 | TRUE | Amp | N |
| E4 | POLL | SYBR | None | 24.465 | 6.0798 | | -0.43687 | | 1.353668 | | FALSE | 0.5 | TRUE | Amp | N |
| E5 | PRKDC | SYBR | None | 23.624 | 5.2388 | | -0.22027 | | 1.164953 | | FALSE | 0.5 | TRUE | Amp | N |
| E6 | RAD18 | SYBR | None | 18.131 | -0.2542 | | 0.177819 | | 0.884039 | | FALSE | 0.5 | TRUE | Amp | N |
| E7 | RAD21 | SYBR | None | 18.063 | -0.3222 | | -0.45749 | | 1.373154 | | FALSE | 0.5 | TRUE | Amp | N |
| E8 | RAD23A | SYBR | None | 22.043 | 3.6578 | | -0.47479 | | 1.389717 | | FALSE | 0.5 | TRUE | Amp | N |
| E9 | RAD23B | SYBR | None | 24.158 | 5.7728 | | -0.52142 | | 1.435365 | | FALSE | 0.5 | TRUE | Amp | N |
| E10 | RAD50 | SYBR | None | 19.906 | 1.5208 | | -0.09527 | | 1.068262 | | FALSE | 0.5 | TRUE | Amp | N |
| E11 | RAD51 | SYBR | None | 20.946 | 2.5608 | | -0.48001 | | 1.394751 | | FALSE | 0.5 | TRUE | Amp | N |
| E12 | RAD51B | SYBR | None | 20.01 | 1.6248 | | -0.19702 | | 1.146332 | | FALSE | 0.5 | TRUE | Amp | N |
| F1 | RAD51C | SYBR | None | 19.162 | 0.7768 | | 0.069422 | | 0.95302 | | FALSE | 0.5 | TRUE | Amp | N |
| F2 | RAD51D | SYBR | None | 24.39 | 6.0048 | | 0.482591 | | 0.715691 | | FALSE | 0.5 | TRUE | Amp | N |
| F3 | RAD52 | SYBR | None | 25.774 | 7.3888 | | -0.21133 | | 1.157753 | | FALSE | 0.5 | TRUE | Amp | N |
| F4 | RAD54L | SYBR | None | 22.358 | 3.9728 | | -0.42098 | | 1.33884 | | FALSE | 0.5 | TRUE | Amp | N |
| F5 | RFC1 | SYBR | None | 23.9 | 5.5148 | | -0.04 | | 1.028112 | | FALSE | 0.5 | TRUE | Amp | N |
| F6 | RPA1 | SYBR | None | 17.864 | -0.5212 | | 0.392447 | | 0.761836 | | FALSE | 0.5 | TRUE | Amp | N |
| F7 | RPA3 | SYBR | None | 23.821 | 5.4358 | | 0.090545 | | 0.939168 | | FALSE | 0.5 | TRUE | Amp | N |
| F8 | SLK | SYBR | None | 20.779 | 2.3938 | | -0.16508 | | 1.121226 | | FALSE | 0.5 | TRUE | Amp | N |
| F9 | SMUG1 | SYBR | None | 26.401 | 8.0158 | | -0.45675 | | 1.372448 | | FALSE | 0.5 | TRUE | Amp | N |
| F10 | TDG | SYBR | None | 21.835 | 3.4498 | | -0.15082 | | 1.110197 | | FALSE | 0.5 | TRUE | Amp | N |
| F11 | TOP3A | SYBR | None | 25.604 | 7.2188 | | 0.534629 | | 0.690336 | | FALSE | 0.5 | TRUE | Amp | N |
| F12 | TOP3B | SYBR | None | 27.112 | 8.7268 | | -0.52268 | | 1.436624 | | FALSE | 0.5 | TRUE | Amp | N |
| G1 | TREX1 | SYBR | None | 27.384 | 8.9988 | | -0.63476 | | 1.552677 | | FALSE | 0.5 | TRUE | Amp | N |
| G2 | UNG | SYBR | None | 20.184 | 1.7988 | | 0.249312 | | 0.841297 | | FALSE | 0.5 | TRUE | Amp | N |
| G3 | XAB2 | SYBR | None | 23.555 | 5.1698 | | -0.2243 | | 1.168213 | | FALSE | 0.5 | TRUE | Amp | N |
| G4 | XPA | SYBR | None | 30.962 | 12.5768 | | -0.47668 | | 1.391534 | | FALSE | 0.5 | TRUE | Amp | N |
| G5 | XPC | SYBR | None | 24.998 | 6.6128 | | -0.48795 | | 1.402448 | | FALSE | 0.5 | TRUE | Amp | N |
| G6 | XRCC1 | SYBR | None | 20.531 | 2.1458 | | -0.1323 | | 1.096042 | | FALSE | 0.5 | TRUE | Amp | N |
| G7 | XRCC2 | SYBR | None | 21.872 | 3.4868 | | 0.084712 | | 0.942973 | | FALSE | 0.5 | TRUE | Amp | N |
| G8 | XRCC3 | SYBR | None | 23.653 | 5.2678 | | -0.18507 | | 1.136874 | | FALSE | 0.5 | TRUE | Amp | N |
| G9 | XRCC4 | SYBR | None | 25.623 | 7.2378 | | 0.54771 | | 0.684105 | | FALSE | 0.5 | TRUE | Amp | N |
| G10 | XRCC5 | SYBR | None | 17.634 | -0.7512 | | 0.067907 | | 0.954021 | | FALSE | 0.5 | TRUE | Amp | N |
| G11 | XRCC6 | SYBR | None | 19.399 | 1.0138 | | -0.09181 | | 1.065703 | | FALSE | 0.5 | TRUE | Amp | N |
| G12 | XRCC6BP1 | SYBR | None | 23.917 | 5.5318 | | -0.00232 | | 1.00161 | | FALSE | 0.5 | TRUE | Amp | N |
| H1 | ACTB | SYBR | None | 18.412 |  | |  | |  | | FALSE | 0.5 | TRUE | Amp | N |
| H2 | B2M | SYBR | None | 16.583 |  | |  | |  | | FALSE | 0.5 | TRUE | Amp | N |
| H3 | GAPDH | SYBR | None | 20.92 |  | |  | |  | | FALSE | 0.5 | TRUE | Amp | N |
| H4 | HPRT1 | SYBR | None | 20.805 |  | |  | |  | | FALSE | 0.5 | TRUE | Amp | N |
| H5 | RPLP0 | SYBR | None | 15.206 |  | |  | |  | | FALSE | 0.5 | TRUE | Amp | N |
| POI 21 |  |  |  |  |  | |  | |  | |  |  |  |  |  |
| Well Position | Gene Symbol | Reporter | Quencher | CT | Delta Ct | | Delta Delta Ct | | 2^(-Delta Delta Ct) | | Automatic Ct Threshold | Ct Threshold | Automatic Baseline | Amp Status | MTP |
| A1 | APEX1 | SYBR | None | 19.33 | 0.5542 | | 0.6032 | | 0.658292 | | FALSE | 0.5 | TRUE | Amp | N |
| A2 | APEX2 | SYBR | None | 23.128 | 4.3522 | | 0.6172 | | 0.651935 | | FALSE | 0.5 | TRUE | Amp | N |
| A3 | ATM | SYBR | None | 23.852 | 5.0762 | | -0.0678 | | 1.048117 | | FALSE | 0.5 | TRUE | Amp | N |
| A4 | ATR | SYBR | None | 22.882 | 4.1062 | | -0.3998 | | 1.319325 | | FALSE | 0.5 | TRUE | Amp | N |
| A5 | ATXN3 | SYBR | None | 21.666 | 2.8902 | | 0.6702 | | 0.62842 | | FALSE | 0.5 | TRUE | Amp | N |
| A6 | BRCA1 | SYBR | None | 21.967 | 3.1912 | | -0.0568 | | 1.040156 | | FALSE | 0.5 | TRUE | Amp | N |
| A7 | BRCA2 | SYBR | None | 19.517 | 0.7412 | | 0.2972 | | 0.81383 | | FALSE | 0.5 | TRUE | Amp | N |
| A8 | BRIP1 | SYBR | None | 23.809 | 5.0332 | | 0.2992 | | 0.812703 | | FALSE | 0.5 | TRUE | Amp | N |
| A9 | CCNH | SYBR | None | 24.923 | 6.1472 | | -0.4018 | | 1.321155 | | FALSE | 0.5 | TRUE | Amp | N |
| A10 | CCNO | SYBR | None | 23.701 | 4.9252 | | 0.6862 | | 0.621489 | | FALSE | 0.5 | TRUE | Amp | N |
| A11 | CDK7 | SYBR | None | 14.598 | -4.1778 | | -0.0268 | | 1.01875 | | FALSE | 0.5 | TRUE | Amp | N |
| A12 | DDB1 | SYBR | None | 20.783 | 2.0072 | | -0.3498 | | 1.274384 | | FALSE | 0.5 | TRUE | Amp | N |
| B1 | DDB2 | SYBR | None | 24.01 | 5.2342 | | 0.3392 | | 0.79048 | | FALSE | 0.5 | TRUE | Amp | N |
| B2 | DMC1 | SYBR | None | 28.159 | 9.3832 | | 0.3102 | | 0.80653 | | FALSE | 0.5 | TRUE | Amp | N |
| B3 | ERCC1 | SYBR | None | 23.057 | 4.2812 | | 0.4372 | | 0.738567 | | FALSE | 0.5 | TRUE | Amp | N |
| B4 | ERCC2 | SYBR | None | 24.109 | 5.3332 | | -0.3848 | | 1.305679 | | FALSE | 0.5 | TRUE | Amp | N |
| B5 | ERCC3 | SYBR | None | 22.674 | 3.8982 | | 0.6882 | | 0.620628 | | FALSE | 0.5 | TRUE | Amp | N |
| B6 | ERCC4 | SYBR | None | 28.112 | 9.3362 | | -0.0278 | | 1.019456 | | FALSE | 0.5 | TRUE | Amp | N |
| B7 | ERCC5 | SYBR | None | 26.225 | 7.4492 | | 0.7582 | | 0.591234 | | FALSE | 0.5 | TRUE | Amp | N |
| B8 | ERCC6 | SYBR | None | 17.902 | -0.8738 | | -0.3718 | | 1.293966 | | FALSE | 0.5 | TRUE | Amp | N |
| B9 | ERCC8 | SYBR | None | 22.681 | 3.9052 | | -0.4718 | | 1.386839 | | FALSE | 0.5 | TRUE | Amp | N |
| B10 | EXO1 | SYBR | None | 21.337 | 2.5612 | | 0.6352 | | 0.643852 | | FALSE | 0.5 | TRUE | Amp | N |
| B11 | FEN1 | SYBR | None | 17.698 | -1.0778 | | -0.3978 | | 1.317497 | | FALSE | 0.5 | TRUE | Amp | N |
| B12 | LIG1 | SYBR | None | 22.934 | 4.1582 | | 0.4422 | | 0.736011 | | FALSE | 0.5 | TRUE | Amp | N |
| C1 | LIG3 | SYBR | None | 25.327 | 6.5512 | | -0.5618 | | 1.47611 | | FALSE | 0.5 | TRUE | Amp | N |
| C2 | LIG4 | SYBR | None | 26.264 | 7.4882 | | 0.6552 | | 0.634987 | | FALSE | 0.5 | TRUE | Amp | N |
| C3 | MGMT | SYBR | None | 21.012 | 2.2362 | | -0.9278 | | 1.902373 | | FALSE | 0.5 | TRUE | Amp | N |
| C4 | MLH1 | SYBR | None | 22.616 | 3.8402 | | -0.1158 | | 1.083576 | | FALSE | 0.5 | TRUE | Amp | N |
| C5 | MLH3 | SYBR | None | 25.502 | 6.7262 | | -0.0208 | | 1.014522 | | FALSE | 0.5 | TRUE | Amp | N |
| C6 | MMS19 | SYBR | None | 25.745 | 6.9692 | | -0.4148 | | 1.333114 | | FALSE | 0.5 | TRUE | Amp | N |
| C7 | MPG | SYBR | None | 21.76 | 2.9842 | | -0.0888 | | 1.063485 | | FALSE | 0.5 | TRUE | Amp | N |
| C8 | MRE11A | SYBR | None | 18.987 | 0.2112 | | 0.2752 | | 0.826336 | | FALSE | 0.5 | TRUE | Amp | N |
| C9 | MSH2 | SYBR | None | 17.036 | -1.7398 | | -0.1088 | | 1.078331 | | FALSE | 0.5 | TRUE | Amp | N |
| C10 | MSH3 | SYBR | None | 19.731 | 0.9552 | | -0.4038 | | 1.322988 | | FALSE | 0.5 | TRUE | Amp | N |
| C11 | MSH4 | SYBR | None | 20.959 | 2.1832 | | -0.3488 | | 1.273501 | | FALSE | 0.5 | TRUE | Amp | N |
| C12 | MSH5 | SYBR | None | 28.99 | 10.2142 | | -0.1418 | | 1.103281 | | FALSE | 0.5 | TRUE | Amp | N |
| D1 | MSH6 | SYBR | None | 20.605 | 1.8292 | | -0.5728 | | 1.487408 | | FALSE | 0.5 | TRUE | Amp | N |
| D2 | MUTYH | SYBR | None | 24.759 | 5.9832 | | 0.5712 | | 0.673057 | | FALSE | 0.5 | TRUE | Amp | N |
| D3 | NEIL1 | SYBR | None | 26.878 | 8.1022 | | -0.5208 | | 1.434751 | | FALSE | 0.5 | TRUE | Amp | N |
| D4 | NEIL2 | SYBR | None | 24.645 | 5.8692 | | 0.2982 | | 0.813266 | | FALSE | 0.5 | TRUE | Amp | N |
| D5 | NEIL3 | SYBR | None | 21.534 | 2.7582 | | -0.1058 | | 1.076091 | | FALSE | 0.5 | TRUE | Amp | N |
| D6 | NTHL1 | SYBR | None | 21.692 | 2.9162 | | 0.3182 | | 0.80207 | | FALSE | 0.5 | TRUE | Amp | N |
| D7 | OGG1 | SYBR | None | 22.288 | 3.5122 | | -0.0848 | | 1.060541 | | FALSE | 0.5 | TRUE | Amp | N |
| D8 | PARP1 | SYBR | None | 21.821 | 3.0452 | | 0.3692 | | 0.774212 | | FALSE | 0.5 | TRUE | Amp | N |
| D9 | PARP2 | SYBR | None | 21.939 | 3.1632 | | 0.4502 | | 0.731941 | | FALSE | 0.5 | TRUE | Amp | N |
| D10 | PARP3 | SYBR | None | 25.241 | 6.4652 | | 0.7342 | | 0.601151 | | FALSE | 0.5 | TRUE | Amp | N |
| D11 | PMS1 | SYBR | None | 19.253 | 0.4772 | | 0.5732 | | 0.672124 | | FALSE | 0.5 | TRUE | Amp | N |
| D12 | PMS2 | SYBR | None | 22.903 | 4.1272 | | 0.2852 | | 0.820628 | | FALSE | 0.5 | TRUE | Amp | N |
| E1 | PNKP | SYBR | None | 24.923 | 6.1472 | | -0.0738 | | 1.052485 | | FALSE | 0.5 | TRUE | Amp | N |
| E2 | POLB | SYBR | None | 18.698 | -0.0778 | | 0.5672 | | 0.674925 | | FALSE | 0.5 | TRUE | Amp | N |
| E3 | POLD3 | SYBR | None | 21.069 | 2.2932 | | 0.5282 | | 0.693419 | | FALSE | 0.5 | TRUE | Amp | N |
| E4 | POLL | SYBR | None | 24.697 | 5.9212 | | -0.5958 | | 1.51131 | | FALSE | 0.5 | TRUE | Amp | N |
| E5 | PRKDC | SYBR | None | 23.666 | 4.8902 | | -0.5688 | | 1.483289 | | FALSE | 0.5 | TRUE | Amp | N |
| E6 | RAD18 | SYBR | None | 18.932 | 0.1562 | | 0.5882 | | 0.665172 | | FALSE | 0.5 | TRUE | Amp | N |
| E7 | RAD21 | SYBR | None | 18.545 | -0.2308 | | -0.3658 | | 1.288596 | | FALSE | 0.5 | TRUE | Amp | N |
| E8 | RAD23A | SYBR | None | 22.809 | 4.0332 | | -0.0998 | | 1.071625 | | FALSE | 0.5 | TRUE | Amp | N |
| E9 | RAD23B | SYBR | None | 24.933 | 6.1572 | | -0.1368 | | 1.099464 | | FALSE | 0.5 | TRUE | Amp | N |
| E10 | RAD50 | SYBR | None | 20.948 | 2.1722 | | 0.5562 | | 0.680091 | | FALSE | 0.5 | TRUE | Amp | N |
| E11 | RAD51 | SYBR | None | 21.784 | 3.0082 | | -0.0328 | | 1.022996 | | FALSE | 0.5 | TRUE | Amp | N |
| E12 | RAD51B | SYBR | None | 20.195 | 1.4192 | | -0.4028 | | 1.322071 | | FALSE | 0.5 | TRUE | Amp | N |
| F1 | RAD51C | SYBR | None | 19.47 | 0.6942 | | -0.0128 | | 1.008912 | | FALSE | 0.5 | TRUE | Amp | N |
| F2 | RAD51D | SYBR | None | 24.193 | 5.4172 | | -0.1048 | | 1.075345 | | FALSE | 0.5 | TRUE | Amp | N |
| F3 | RAD52 | SYBR | None | 27.034 | 8.2582 | | 0.6582 | | 0.633668 | | FALSE | 0.5 | TRUE | Amp | N |
| F4 | RAD54L | SYBR | None | 22.783 | 4.0072 | | -0.3868 | | 1.30749 | | FALSE | 0.5 | TRUE | Amp | N |
| F5 | RFC1 | SYBR | None | 23.93 | 5.1542 | | -0.4008 | | 1.32024 | | FALSE | 0.5 | TRUE | Amp | N |
| F6 | RPA1 | SYBR | None | 17.82 | -0.9558 | | -0.0418 | | 1.029397 | | FALSE | 0.5 | TRUE | Amp | N |
| F7 | RPA3 | SYBR | None | 24.061 | 5.2852 | | -0.0598 | | 1.042321 | | FALSE | 0.5 | TRUE | Amp | N |
| F8 | SLK | SYBR | None | 20.974 | 2.1982 | | -0.3608 | | 1.284138 | | FALSE | 0.5 | TRUE | Amp | N |
| F9 | SMUG1 | SYBR | None | 27.973 | 9.1972 | | 0.7242 | | 0.605333 | | FALSE | 0.5 | TRUE | Amp | N |
| F10 | TDG | SYBR | None | 21.894 | 3.1182 | | -0.4828 | | 1.397453 | | FALSE | 0.5 | TRUE | Amp | N |
| F11 | TOP3A | SYBR | None | 25.381 | 6.6052 | | -0.0788 | | 1.056139 | | FALSE | 0.5 | TRUE | Amp | N |
| F12 | TOP3B | SYBR | None | 27.895 | 9.1192 | | -0.1308 | | 1.094901 | | FALSE | 0.5 | TRUE | Amp | N |
| G1 | TREX1 | SYBR | None | 27.913 | 9.1372 | | -0.4968 | | 1.41108 | | FALSE | 0.5 | TRUE | Amp | N |
| G2 | UNG | SYBR | None | 20.63 | 1.8542 | | 0.3042 | | 0.809891 | | FALSE | 0.5 | TRUE | Amp | N |
| G3 | XAB2 | SYBR | None | 24.076 | 5.3002 | | -0.0938 | | 1.067177 | | FALSE | 0.5 | TRUE | Amp | N |
| G4 | XPA | SYBR | None | 32.122 | 13.3462 | | 0.2922 | | 0.816656 | | FALSE | 0.5 | TRUE | Amp | N |
| G5 | XPC | SYBR | None | 26.514 | 7.7382 | | 0.6372 | | 0.64296 | | FALSE | 0.5 | TRUE | Amp | N |
| G6 | XRCC1 | SYBR | None | 20.667 | 1.8912 | | -0.3868 | | 1.30749 | | FALSE | 0.5 | TRUE | Amp | N |
| G7 | XRCC2 | SYBR | None | 21.688 | 2.9122 | | -0.4898 | | 1.40425 | | FALSE | 0.5 | TRUE | Amp | N |
| G8 | XRCC3 | SYBR | None | 24.096 | 5.3202 | | -0.1328 | | 1.09642 | | FALSE | 0.5 | TRUE | Amp | N |
| G9 | XRCC4 | SYBR | None | 25.071 | 6.2952 | | -0.3948 | | 1.31476 | | FALSE | 0.5 | TRUE | Amp | N |
| G10 | XRCC5 | SYBR | None | 17.872 | -0.9038 | | -0.0848 | | 1.060541 | | FALSE | 0.5 | TRUE | Amp | N |
| G11 | XRCC6 | SYBR | None | 20.203 | 1.4272 | | 0.3212 | | 0.800404 | | FALSE | 0.5 | TRUE | Amp | N |
| G12 | XRCC6BP1 | SYBR | None | 24.831 | 6.0552 | | 0.5212 | | 0.696792 | | FALSE | 0.5 | TRUE | Amp | N |
| H1 | ACTB | SYBR | None | 19.153 |  | |  | |  | | FALSE | 0.5 | TRUE | Amp | N |
| H2 | B2M | SYBR | None | 17.049 |  | |  | |  | | FALSE | 0.5 | TRUE | Amp | N |
| H3 | GAPDH | SYBR | None | 21.102 |  | |  | |  | | FALSE | 0.5 | TRUE | Amp | N |
| H4 | HPRT1 | SYBR | None | 21.054 |  | |  | |  | | FALSE | 0.5 | TRUE | Amp | N |
| H5 | RPLP0 | SYBR | None | 15.521 |  | |  | |  | | FALSE | 0.5 | TRUE | Amp | N |
| POI 22 |  |  |  |  |  | |  | |  | |  |  |  |  |  |
| Well Position | Gene Symbol | Reporter | Quencher | CT | Delta Ct | | Delta Delta Ct | | 2^(-Delta Delta Ct) | | Automatic Ct Threshold | Ct Threshold | Automatic Baseline | Amp Status | MTP |
| A1 | APEX1 | SYBR | None | 17.926 | -0.6448 | | -0.5958 | | 1.51131 | | FALSE | 0.5 | TRUE | Amp | N |
| A2 | APEX2 | SYBR | None | 21.842 | 3.2712 | | -0.4638 | | 1.37917 | | FALSE | 0.5 | TRUE | Amp | N |
| A3 | ATM | SYBR | None | 23.6 | 5.0292 | | -0.1148 | | 1.082825 | | FALSE | 0.5 | TRUE | Amp | N |
| A4 | ATR | SYBR | None | 22.885 | 4.3142 | | -0.1918 | | 1.142188 | | FALSE | 0.5 | TRUE | Amp | N |
| A5 | ATXN3 | SYBR | None | 21.24 | 2.6692 | | 0.4492 | | 0.732449 | | FALSE | 0.5 | TRUE | Amp | N |
| A6 | BRCA1 | SYBR | None | 22.072 | 3.5012 | | 0.2532 | | 0.839033 | | FALSE | 0.5 | TRUE | Amp | N |
| A7 | BRCA2 | SYBR | None | 18.921 | 0.3502 | | -0.0938 | | 1.067177 | | FALSE | 0.5 | TRUE | Amp | N |
| A8 | BRIP1 | SYBR | None | 23.48 | 4.9092 | | 0.1752 | | 0.885645 | | FALSE | 0.5 | TRUE | Amp | N |
| A9 | CCNH | SYBR | None | 25.743 | 7.1722 | | 0.6232 | | 0.649229 | | FALSE | 0.5 | TRUE | Amp | N |
| A10 | CCNO | SYBR | None | 23.376 | 4.8052 | | 0.5662 | | 0.675393 | | FALSE | 0.5 | TRUE | Amp | N |
| A11 | CDK7 | SYBR | None | 14.775 | -3.7958 | | 0.3552 | | 0.781761 | | FALSE | 0.5 | TRUE | Amp | N |
| A12 | DDB1 | SYBR | None | 20.46 | 1.8892 | | -0.4678 | | 1.382999 | | FALSE | 0.5 | TRUE | Amp | N |
| B1 | DDB2 | SYBR | None | 23.612 | 5.0412 | | 0.1462 | | 0.903627 | | FALSE | 0.5 | TRUE | Amp | N |
| B2 | DMC1 | SYBR | None | 28.306 | 9.7352 | | 0.6622 | | 0.631914 | | FALSE | 0.5 | TRUE | Amp | N |
| B3 | ERCC1 | SYBR | None | 22.637 | 4.0662 | | 0.2222 | | 0.857257 | | FALSE | 0.5 | TRUE | Amp | N |
| B4 | ERCC2 | SYBR | None | 24.062 | 5.4912 | | -0.2268 | | 1.170236 | | FALSE | 0.5 | TRUE | Amp | N |
| B5 | ERCC3 | SYBR | None | 21.235 | 2.6642 | | -0.5458 | | 1.45983 | | FALSE | 0.5 | TRUE | Amp | N |
| B6 | ERCC4 | SYBR | None | 27.8 | 9.2292 | | -0.1348 | | 1.097941 | | FALSE | 0.5 | TRUE | Amp | N |
| B7 | ERCC5 | SYBR | None | 25.483 | 6.9122 | | 0.2212 | | 0.857852 | | FALSE | 0.5 | TRUE | Amp | N |
| B8 | ERCC6 | SYBR | None | 17.871 | -0.6998 | | -0.1978 | | 1.146948 | | FALSE | 0.5 | TRUE | Amp | N |
| B9 | ERCC8 | SYBR | None | 22.48 | 3.9092 | | -0.4678 | | 1.382999 | | FALSE | 0.5 | TRUE | Amp | N |
| B10 | EXO1 | SYBR | None | 21.048 | 2.4772 | | 0.5512 | | 0.682452 | | FALSE | 0.5 | TRUE | Amp | N |
| B11 | FEN1 | SYBR | None | 17.685 | -0.8858 | | -0.2058 | | 1.153326 | | FALSE | 0.5 | TRUE | Amp | N |
| B12 | LIG1 | SYBR | None | 22.5 | 3.9292 | | 0.2132 | | 0.862622 | | FALSE | 0.5 | TRUE | Amp | N |
| C1 | LIG3 | SYBR | None | 26.329 | 7.7582 | | 0.6452 | | 0.639404 | | FALSE | 0.5 | TRUE | Amp | N |
| C2 | LIG4 | SYBR | None | 25.961 | 7.3902 | | 0.5572 | | 0.67962 | | FALSE | 0.5 | TRUE | Amp | N |
| C3 | MGMT | SYBR | None | 21.65 | 3.0792 | | -0.0848 | | 1.060541 | | FALSE | 0.5 | TRUE | Amp | N |
| C4 | MLH1 | SYBR | None | 22.438 | 3.8672 | | -0.0888 | | 1.063485 | | FALSE | 0.5 | TRUE | Amp | N |
| C5 | MLH3 | SYBR | None | 25.897 | 7.3262 | | 0.5792 | | 0.669335 | | FALSE | 0.5 | TRUE | Amp | N |
| C6 | MMS19 | SYBR | None | 25.432 | 6.8612 | | -0.5228 | | 1.436741 | | FALSE | 0.5 | TRUE | Amp | N |
| C7 | MPG | SYBR | None | 21.559 | 2.9882 | | -0.0848 | | 1.060541 | | FALSE | 0.5 | TRUE | Amp | N |
| C8 | MRE11A | SYBR | None | 18.752 | 0.1812 | | 0.2452 | | 0.843699 | | FALSE | 0.5 | TRUE | Amp | N |
| C9 | MSH2 | SYBR | None | 16.503 | -2.0678 | | -0.4368 | | 1.353599 | | FALSE | 0.5 | TRUE | Amp | N |
| C10 | MSH3 | SYBR | None | 19.709 | 1.1382 | | -0.2208 | | 1.16538 | | FALSE | 0.5 | TRUE | Amp | N |
| C11 | MSH4 | SYBR | None | 20.665 | 2.0942 | | -0.4378 | | 1.354537 | | FALSE | 0.5 | TRUE | Amp | N |
| C12 | MSH5 | SYBR | None | 28.392 | 9.8212 | | -0.5348 | | 1.448741 | | FALSE | 0.5 | TRUE | Amp | N |
| D1 | MSH6 | SYBR | None | 20.807 | 2.2362 | | -0.1658 | | 1.121788 | | FALSE | 0.5 | TRUE | Amp | N |
| D2 | MUTYH | SYBR | None | 23.93 | 5.3592 | | -0.0528 | | 1.037276 | | FALSE | 0.5 | TRUE | Amp | N |
| D3 | NEIL1 | SYBR | None | 26.62 | 8.0492 | | -0.5738 | | 1.488439 | | FALSE | 0.5 | TRUE | Amp | N |
| D4 | NEIL2 | SYBR | None | 23.626 | 5.0552 | | -0.5158 | | 1.429787 | | FALSE | 0.5 | TRUE | Amp | N |
| D5 | NEIL3 | SYBR | None | 20.879 | 2.3082 | | -0.5558 | | 1.469984 | | FALSE | 0.5 | TRUE | Amp | N |
| D6 | NTHL1 | SYBR | None | 21.007 | 2.4362 | | -0.1618 | | 1.118682 | | FALSE | 0.5 | TRUE | Amp | N |
| D7 | OGG1 | SYBR | None | 21.688 | 3.1172 | | -0.4798 | | 1.39455 | | FALSE | 0.5 | TRUE | Amp | N |
| D8 | PARP1 | SYBR | None | 21.519 | 2.9482 | | 0.2722 | | 0.828056 | | FALSE | 0.5 | TRUE | Amp | N |
| D9 | PARP2 | SYBR | None | 21.534 | 2.9632 | | 0.2502 | | 0.84078 | | FALSE | 0.5 | TRUE | Amp | N |
| D10 | PARP3 | SYBR | None | 24.798 | 6.2272 | | 0.4962 | | 0.708972 | | FALSE | 0.5 | TRUE | Amp | N |
| D11 | PMS1 | SYBR | None | 18.282 | -0.2888 | | -0.1928 | | 1.14298 | | FALSE | 0.5 | TRUE | Amp | N |
| D12 | PMS2 | SYBR | None | 22.909 | 4.3382 | | 0.4962 | | 0.708972 | | FALSE | 0.5 | TRUE | Amp | N |
| E1 | PNKP | SYBR | None | 25.301 | 6.7302 | | 0.5092 | | 0.702612 | | FALSE | 0.5 | TRUE | Amp | N |
| E2 | POLB | SYBR | None | 17.749 | -0.8218 | | -0.1768 | | 1.130374 | | FALSE | 0.5 | TRUE | Amp | N |
| E3 | POLD3 | SYBR | None | 20.824 | 2.2532 | | 0.4882 | | 0.712914 | | FALSE | 0.5 | TRUE | Amp | N |
| E4 | POLL | SYBR | None | 24.551 | 5.9802 | | -0.5368 | | 1.450751 | | FALSE | 0.5 | TRUE | Amp | N |
| E5 | PRKDC | SYBR | None | 23.873 | 5.3022 | | -0.1568 | | 1.114812 | | FALSE | 0.5 | TRUE | Amp | N |
| E6 | RAD18 | SYBR | None | 18.578 | 0.0072 | | 0.4392 | | 0.737543 | | FALSE | 0.5 | TRUE | Amp | N |
| E7 | RAD21 | SYBR | None | 18.552 | -0.0188 | | -0.1538 | | 1.112496 | | FALSE | 0.5 | TRUE | Amp | N |
| E8 | RAD23A | SYBR | None | 22.929 | 4.3582 | | 0.2252 | | 0.855476 | | FALSE | 0.5 | TRUE | Amp | N |
| E9 | RAD23B | SYBR | None | 25.084 | 6.5132 | | 0.2192 | | 0.859042 | | FALSE | 0.5 | TRUE | Amp | N |
| E10 | RAD50 | SYBR | None | 19.723 | 1.1522 | | -0.4638 | | 1.37917 | | FALSE | 0.5 | TRUE | Amp | N |
| E11 | RAD51 | SYBR | None | 21.486 | 2.9152 | | -0.1258 | | 1.091113 | | FALSE | 0.5 | TRUE | Amp | N |
| E12 | RAD51B | SYBR | None | 20.329 | 1.7582 | | -0.0638 | | 1.045215 | | FALSE | 0.5 | TRUE | Amp | N |
| F1 | RAD51C | SYBR | None | 19.5 | 0.9292 | | 0.2222 | | 0.857257 | | FALSE | 0.5 | TRUE | Amp | N |
| F2 | RAD51D | SYBR | None | 23.545 | 4.9742 | | -0.5478 | | 1.461855 | | FALSE | 0.5 | TRUE | Amp | N |
| F3 | RAD52 | SYBR | None | 25.343 | 6.7722 | | -0.8278 | | 1.774977 | | FALSE | 0.5 | TRUE | Amp | N |
| F4 | RAD54L | SYBR | None | 23.547 | 4.9762 | | 0.5822 | | 0.667944 | | FALSE | 0.5 | TRUE | Amp | N |
| F5 | RFC1 | SYBR | None | 24.046 | 5.4752 | | -0.0798 | | 1.056872 | | FALSE | 0.5 | TRUE | Amp | N |
| F6 | RPA1 | SYBR | None | 17.843 | -0.7278 | | 0.1862 | | 0.878918 | | FALSE | 0.5 | TRUE | Amp | N |
| F7 | RPA3 | SYBR | None | 24.985 | 6.4142 | | 1.0692 | | 0.476583 | | FALSE | 0.5 | TRUE | Amp | N |
| F8 | SLK | SYBR | None | 20.649 | 2.0782 | | -0.4808 | | 1.395517 | | FALSE | 0.5 | TRUE | Amp | N |
| F9 | SMUG1 | SYBR | None | 27.679 | 9.1082 | | 0.6352 | | 0.643852 | | FALSE | 0.5 | TRUE | Amp | N |
| F10 | TDG | SYBR | None | 21.984 | 3.4132 | | -0.1878 | | 1.139025 | | FALSE | 0.5 | TRUE | Amp | N |
| F11 | TOP3A | SYBR | None | 25.805 | 7.2342 | | 0.5502 | | 0.682925 | | FALSE | 0.5 | TRUE | Amp | N |
| F12 | TOP3B | SYBR | None | 28.465 | 9.8942 | | 0.6442 | | 0.639847 | | FALSE | 0.5 | TRUE | Amp | N |
| G1 | TREX1 | SYBR | None | 27.492 | 8.9212 | | -0.7128 | | 1.638982 | | FALSE | 0.5 | TRUE | Amp | N |
| G2 | UNG | SYBR | None | 20.642 | 2.0712 | | 0.5212 | | 0.696792 | | FALSE | 0.5 | TRUE | Amp | N |
| G3 | XAB2 | SYBR | None | 23.805 | 5.2342 | | -0.1598 | | 1.117132 | | FALSE | 0.5 | TRUE | Amp | N |
| G4 | XPA | SYBR | None | 32.38 | 13.8092 | | 0.7552 | | 0.592464 | | FALSE | 0.5 | TRUE | Amp | N |
| G5 | XPC | SYBR | None | 25.838 | 7.2672 | | 0.1662 | | 0.891187 | | FALSE | 0.5 | TRUE | Amp | N |
| G6 | XRCC1 | SYBR | None | 20.68 | 2.1092 | | -0.1688 | | 1.124123 | | FALSE | 0.5 | TRUE | Amp | N |
| G7 | XRCC2 | SYBR | None | 21.967 | 3.3962 | | -0.0058 | | 1.004028 | | FALSE | 0.5 | TRUE | Amp | N |
| G8 | XRCC3 | SYBR | None | 24.508 | 5.9372 | | 0.4842 | | 0.714893 | | FALSE | 0.5 | TRUE | Amp | N |
| G9 | XRCC4 | SYBR | None | 25.866 | 7.2952 | | 0.6052 | | 0.65738 | | FALSE | 0.5 | TRUE | Amp | N |
| G10 | XRCC5 | SYBR | None | 17.942 | -0.6288 | | 0.1902 | | 0.876484 | | FALSE | 0.5 | TRUE | Amp | N |
| G11 | XRCC6 | SYBR | None | 19.025 | 0.4542 | | -0.6518 | | 1.571127 | | FALSE | 0.5 | TRUE | Amp | N |
| G12 | XRCC6BP1 | SYBR | None | 24.357 | 5.7862 | | 0.2522 | | 0.839615 | | FALSE | 0.5 | TRUE | Amp | N |
| H1 | ACTB | SYBR | None | 18.658 |  | |  | |  | | FALSE | 0.5 | TRUE | Amp | N |
| H2 | B2M | SYBR | None | 17.151 |  | |  | |  | | FALSE | 0.5 | TRUE | Amp | N |
| H3 | GAPDH | SYBR | None | 20.872 |  | |  | |  | | FALSE | 0.5 | TRUE | Amp | N |
| H4 | HPRT1 | SYBR | None | 20.899 |  | |  | |  | | FALSE | 0.5 | TRUE | Amp | N |
| H5 | RPLP0 | SYBR | None | 15.274 |  | |  | |  | | FALSE | 0.5 | TRUE | Amp | N |
| POI 23 | Dead during in vitro culture. | | |  |  | |  | |  | |  |  |  |  |  |
| POI 24 |  |  |  |  |  | |  | |  | |  |  |  |  |  |
| Well Position | Gene Symbol | Reporter | Quencher | CT | Delta Ct | | Delta Delta Ct | | 2^(-Delta Delta Ct) | | Automatic Ct Threshold | Ct Threshold | Automatic Baseline | Amp Status | MTP |
| A1 | APEX1 | SYBR | None | 19.02 | 0.072 | | 0.121 | | 0.91955 | | FALSE | 0.5 | TRUE | Amp | N |
| A2 | APEX2 | SYBR | None | 23.186 | 4.238 | | 0.503 | | 0.705638 | | FALSE | 0.5 | TRUE | Amp | N |
| A3 | ATM | SYBR | None | 23.958 | 5.01 | | -0.134 | | 1.097332 | | FALSE | 0.5 | TRUE | Amp | N |
| A4 | ATR | SYBR | None | 22.998 | 4.05 | | -0.456 | | 1.371733 | | FALSE | 0.5 | TRUE | Amp | N |
| A5 | ATXN3 | SYBR | None | 21.102 | 2.154 | | -0.066 | | 1.04681 | | FALSE | 0.5 | TRUE | Amp | N |
| A6 | BRCA1 | SYBR | None | 22.368 | 3.42 | | 0.172 | | 0.887611 | | FALSE | 0.5 | TRUE | Amp | N |
| A7 | BRCA2 | SYBR | None | 19.214 | 0.266 | | -0.178 | | 1.131314 | | FALSE | 0.5 | TRUE | Amp | N |
| A8 | BRIP1 | SYBR | None | 23.085 | 4.137 | | -0.597 | | 1.512568 | | FALSE | 0.5 | TRUE | Amp | N |
| A9 | CCNH | SYBR | None | 25.038 | 6.09 | | -0.459 | | 1.374589 | | FALSE | 0.5 | TRUE | Amp | N |
| A10 | CCNO | SYBR | None | 22.761 | 3.813 | | -0.426 | | 1.343503 | | FALSE | 0.5 | TRUE | Amp | N |
| A11 | CDK7 | SYBR | None | 15.117 | -3.831 | | 0.32 | | 0.80107 | | FALSE | 0.5 | TRUE | Amp | N |
| A12 | DDB1 | SYBR | None | 20.723 | 1.775 | | -0.582 | | 1.496923 | | FALSE | 0.5 | TRUE | Amp | N |
| B1 | DDB2 | SYBR | None | 23.806 | 4.858 | | -0.037 | | 1.025978 | | FALSE | 0.5 | TRUE | Amp | N |
| B2 | DMC1 | SYBR | None | 27.923 | 8.975 | | -0.098 | | 1.070289 | | FALSE | 0.5 | TRUE | Amp | N |
| B3 | ERCC1 | SYBR | None | 22.647 | 3.699 | | -0.145 | | 1.105731 | | FALSE | 0.5 | TRUE | Amp | N |
| B4 | ERCC2 | SYBR | None | 24.563 | 5.615 | | -0.103 | | 1.074004 | | FALSE | 0.5 | TRUE | Amp | N |
| B5 | ERCC3 | SYBR | None | 21.499 | 2.551 | | -0.659 | | 1.578988 | | FALSE | 0.5 | TRUE | Amp | N |
| B6 | ERCC4 | SYBR | None | 28.245 | 9.297 | | -0.067 | | 1.047536 | | FALSE | 0.5 | TRUE | Amp | N |
| B7 | ERCC5 | SYBR | None | 25.756 | 6.808 | | 0.117 | | 0.922103 | | FALSE | 0.5 | TRUE | Amp | N |
| B8 | ERCC6 | SYBR | None | 18.046 | -0.902 | | -0.4 | | 1.319508 | | FALSE | 0.5 | TRUE | Amp | N |
| B9 | ERCC8 | SYBR | None | 22.731 | 3.783 | | -0.594 | | 1.509426 | | FALSE | 0.5 | TRUE | Amp | N |
| B10 | EXO1 | SYBR | None | 21.285 | 2.337 | | 0.411 | | 0.752102 | | FALSE | 0.5 | TRUE | Amp | N |
| B11 | FEN1 | SYBR | None | 17.798 | -1.15 | | -0.47 | | 1.385109 | | FALSE | 0.5 | TRUE | Amp | N |
| B12 | LIG1 | SYBR | None | 22.113 | 3.165 | | -0.551 | | 1.465101 | | FALSE | 0.5 | TRUE | Amp | N |
| C1 | LIG3 | SYBR | None | 26.22 | 7.272 | | 0.159 | | 0.895646 | | FALSE | 0.5 | TRUE | Amp | N |
| C2 | LIG4 | SYBR | None | 26.289 | 7.341 | | 0.508 | | 0.703197 | | FALSE | 0.5 | TRUE | Amp | N |
| C3 | MGMT | SYBR | None | 21.718 | 2.77 | | -0.394 | | 1.314032 | | FALSE | 0.5 | TRUE | Amp | N |
| C4 | MLH1 | SYBR | None | 22.445 | 3.497 | | -0.459 | | 1.374589 | | FALSE | 0.5 | TRUE | Amp | N |
| C5 | MLH3 | SYBR | None | 25.852 | 6.904 | | 0.157 | | 0.896888 | | FALSE | 0.5 | TRUE | Amp | N |
| C6 | MMS19 | SYBR | None | 26.919 | 7.971 | | 0.587 | | 0.665726 | | FALSE | 0.5 | TRUE | Amp | N |
| C7 | MPG | SYBR | None | 21.885 | 2.937 | | -0.136 | | 1.098854 | | FALSE | 0.5 | TRUE | Amp | N |
| C8 | MRE11A | SYBR | None | 18.972 | 0.024 | | 0.088 | | 0.940826 | | FALSE | 0.5 | TRUE | Amp | N |
| C9 | MSH2 | SYBR | None | 17.496 | -1.452 | | 0.179 | | 0.883315 | | FALSE | 0.5 | TRUE | Amp | N |
| C10 | MSH3 | SYBR | None | 20.157 | 1.209 | | -0.15 | | 1.109569 | | FALSE | 0.5 | TRUE | Amp | N |
| C11 | MSH4 | SYBR | None | 21.048 | 2.1 | | -0.432 | | 1.349103 | | FALSE | 0.5 | TRUE | Amp | N |
| C12 | MSH5 | SYBR | None | 29.16 | 10.212 | | -0.144 | | 1.104964 | | FALSE | 0.5 | TRUE | Amp | N |
| D1 | MSH6 | SYBR | None | 20.645 | 1.697 | | -0.705 | | 1.630145 | | FALSE | 0.5 | TRUE | Amp | N |
| D2 | MUTYH | SYBR | None | 24.853 | 5.905 | | 0.493 | | 0.710546 | | FALSE | 0.5 | TRUE | Amp | N |
| D3 | NEIL1 | SYBR | None | 27.087 | 8.139 | | -0.484 | | 1.398616 | | FALSE | 0.5 | TRUE | Amp | N |
| D4 | NEIL2 | SYBR | None | 24.071 | 5.123 | | -0.448 | | 1.364148 | | FALSE | 0.5 | TRUE | Amp | N |
| D5 | NEIL3 | SYBR | None | 21.424 | 2.476 | | -0.388 | | 1.308578 | | FALSE | 0.5 | TRUE | Amp | N |
| D6 | NTHL1 | SYBR | None | 21.155 | 2.207 | | -0.391 | | 1.311302 | | FALSE | 0.5 | TRUE | Amp | N |
| D7 | OGG1 | SYBR | None | 21.868 | 2.92 | | -0.677 | | 1.598812 | | FALSE | 0.5 | TRUE | Amp | N |
| D8 | PARP1 | SYBR | None | 21.464 | 2.516 | | -0.16 | | 1.117287 | | FALSE | 0.5 | TRUE | Amp | N |
| D9 | PARP2 | SYBR | None | 21.803 | 2.855 | | 0.142 | | 0.906262 | | FALSE | 0.5 | TRUE | Amp | N |
| D10 | PARP3 | SYBR | None | 24.63 | 5.682 | | -0.049 | | 1.034548 | | FALSE | 0.5 | TRUE | Amp | N |
| D11 | PMS1 | SYBR | None | 19.235 | 0.287 | | 0.383 | | 0.766841 | | FALSE | 0.5 | TRUE | Amp | N |
| D12 | PMS2 | SYBR | None | 22.762 | 3.814 | | -0.028 | | 1.019598 | | FALSE | 0.5 | TRUE | Amp | N |
| E1 | PNKP | SYBR | None | 24.693 | 5.745 | | -0.476 | | 1.390882 | | FALSE | 0.5 | TRUE | Amp | N |
| E2 | POLB | SYBR | None | 17.892 | -1.056 | | -0.411 | | 1.329607 | | FALSE | 0.5 | TRUE | Amp | N |
| E3 | POLD3 | SYBR | None | 20.538 | 1.59 | | -0.175 | | 1.128964 | | FALSE | 0.5 | TRUE | Amp | N |
| E4 | POLL | SYBR | None | 24.958 | 6.01 | | -0.507 | | 1.421092 | | FALSE | 0.5 | TRUE | Amp | N |
| E5 | PRKDC | SYBR | None | 23.945 | 4.997 | | -0.462 | | 1.37745 | | FALSE | 0.5 | TRUE | Amp | N |
| E6 | RAD18 | SYBR | None | 18.37 | -0.578 | | -0.146 | | 1.106497 | | FALSE | 0.5 | TRUE | Amp | N |
| E7 | RAD21 | SYBR | None | 18.716 | -0.232 | | -0.367 | | 1.289668 | | FALSE | 0.5 | TRUE | Amp | N |
| E8 | RAD23A | SYBR | None | 22.616 | 3.668 | | -0.465 | | 1.380317 | | FALSE | 0.5 | TRUE | Amp | N |
| E9 | RAD23B | SYBR | None | 24.763 | 5.815 | | -0.479 | | 1.393777 | | FALSE | 0.5 | TRUE | Amp | N |
| E10 | RAD50 | SYBR | None | 20.394 | 1.446 | | -0.17 | | 1.125058 | | FALSE | 0.5 | TRUE | Amp | N |
| E11 | RAD51 | SYBR | None | 21.313 | 2.365 | | -0.676 | | 1.597704 | | FALSE | 0.5 | TRUE | Amp | N |
| E12 | RAD51B | SYBR | None | 20.736 | 1.788 | | -0.034 | | 1.023847 | | FALSE | 0.5 | TRUE | Amp | N |
| F1 | RAD51C | SYBR | None | 19.546 | 0.598 | | -0.109 | | 1.07848 | | FALSE | 0.5 | TRUE | Amp | N |
| F2 | RAD51D | SYBR | None | 24.439 | 5.491 | | -0.031 | | 1.02172 | | FALSE | 0.5 | TRUE | Amp | N |
| F3 | RAD52 | SYBR | None | 26.04 | 7.092 | | -0.508 | | 1.422077 | | FALSE | 0.5 | TRUE | Amp | N |
| F4 | RAD54L | SYBR | None | 22.829 | 3.881 | | -0.513 | | 1.427015 | | FALSE | 0.5 | TRUE | Amp | N |
| F5 | RFC1 | SYBR | None | 24.608 | 5.66 | | 0.105 | | 0.929805 | | FALSE | 0.5 | TRUE | Amp | N |
| F6 | RPA1 | SYBR | None | 17.673 | -1.275 | | -0.361 | | 1.284316 | | FALSE | 0.5 | TRUE | Amp | N |
| F7 | RPA3 | SYBR | None | 24.159 | 5.211 | | -0.134 | | 1.097332 | | FALSE | 0.5 | TRUE | Amp | N |
| F8 | SLK | SYBR | None | 22.007 | 3.059 | | 0.5 | | 0.707107 | | FALSE | 0.5 | TRUE | Amp | N |
| F9 | SMUG1 | SYBR | None | 27.396 | 8.448 | | -0.025 | | 1.01748 | | FALSE | 0.5 | TRUE | Amp | N |
| F10 | TDG | SYBR | None | 22.062 | 3.114 | | -0.487 | | 1.401527 | | FALSE | 0.5 | TRUE | Amp | N |
| F11 | TOP3A | SYBR | None | 25.605 | 6.657 | | -0.027 | | 1.018891 | | FALSE | 0.5 | TRUE | Amp | N |
| F12 | TOP3B | SYBR | None | 28.158 | 9.21 | | -0.04 | | 1.028114 | | FALSE | 0.5 | TRUE | Amp | N |
| G1 | TREX1 | SYBR | None | 28.118 | 9.17 | | -0.464 | | 1.379361 | | FALSE | 0.5 | TRUE | Amp | N |
| G2 | UNG | SYBR | None | 20.356 | 1.408 | | -0.142 | | 1.103434 | | FALSE | 0.5 | TRUE | Amp | N |
| G3 | XAB2 | SYBR | None | 24.189 | 5.241 | | -0.153 | | 1.111879 | | FALSE | 0.5 | TRUE | Amp | N |
| G4 | XPA | SYBR | None | 31.84 | 12.892 | | -0.162 | | 1.118837 | | FALSE | 0.5 | TRUE | Amp | N |
| G5 | XPC | SYBR | None | 25.976 | 7.028 | | -0.073 | | 1.051902 | | FALSE | 0.5 | TRUE | Amp | N |
| G6 | XRCC1 | SYBR | None | 20.707 | 1.759 | | -0.519 | | 1.432962 | | FALSE | 0.5 | TRUE | Amp | N |
| G7 | XRCC2 | SYBR | None | 22.856 | 3.908 | | 0.506 | | 0.704172 | | FALSE | 0.5 | TRUE | Amp | N |
| G8 | XRCC3 | SYBR | None | 24.274 | 5.326 | | -0.127 | | 1.092021 | | FALSE | 0.5 | TRUE | Amp | N |
| G9 | XRCC4 | SYBR | None | 25.596 | 6.648 | | -0.042 | | 1.02954 | | FALSE | 0.5 | TRUE | Amp | N |
| G10 | XRCC5 | SYBR | None | 18.553 | -0.395 | | 0.424 | | 0.745355 | | FALSE | 0.5 | TRUE | Amp | N |
| G11 | XRCC6 | SYBR | None | 19.579 | 0.631 | | -0.475 | | 1.389918 | | FALSE | 0.5 | TRUE | Amp | N |
| G12 | XRCC6BP1 | SYBR | None | 24.604 | 5.656 | | 0.122 | | 0.918913 | | FALSE | 0.5 | TRUE | Amp | N |
| H1 | ACTB | SYBR | None | 18.787 |  | |  | |  | | FALSE | 0.5 | TRUE | Amp | N |
| H2 | B2M | SYBR | None | 17.631 |  | |  | |  | | FALSE | 0.5 | TRUE | Amp | N |
| H3 | GAPDH | SYBR | None | 20.958 |  | |  | |  | | FALSE | 0.5 | TRUE | Amp | N |
| H4 | HPRT1 | SYBR | None | 21.343 |  | |  | |  | | FALSE | 0.5 | TRUE | Amp | N |
| H5 | RPLP0 | SYBR | None | 16.021 |  | |  | |  | | FALSE | 0.5 | TRUE | Amp | N |
| POI 25 |  |  |  |  |  | |  | |  | |  |  |  |  |  |
| Well Position | Gene Symbol | Reporter | Quencher | CT | Delta Ct | | Delta Delta Ct | | 2^(-Delta Delta Ct) | | Automatic Ct Threshold | Ct Threshold | Automatic Baseline | Amp Status | MTP |
| A1 | APEX1 | SYBR | None | 18.177 | -0.0744 | | -0.0254 | | 1.017762 | | FALSE | 0.5 | TRUE | Amp | N |
| A2 | APEX2 | SYBR | None | 22.475 | 4.2236 | | 0.4886 | | 0.712716 | | FALSE | 0.5 | TRUE | Amp | N |
| A3 | ATM | SYBR | None | 23.033 | 4.7816 | | -0.3624 | | 1.285563 | | FALSE | 0.5 | TRUE | Amp | N |
| A4 | ATR | SYBR | None | 22.969 | 4.7176 | | 0.2116 | | 0.863579 | | FALSE | 0.5 | TRUE | Amp | N |
| A5 | ATXN3 | SYBR | None | 19.917 | 1.6656 | | -0.5544 | | 1.468558 | | FALSE | 0.5 | TRUE | Amp | N |
| A6 | BRCA1 | SYBR | None | 21.067 | 2.8156 | | -0.4324 | | 1.349477 | | FALSE | 0.5 | TRUE | Amp | N |
| A7 | BRCA2 | SYBR | None | 18.165 | -0.0864 | | -0.5304 | | 1.44433 | | FALSE | 0.5 | TRUE | Amp | N |
| A8 | BRIP1 | SYBR | None | 22.565 | 4.3136 | | -0.4204 | | 1.338299 | | FALSE | 0.5 | TRUE | Amp | N |
| A9 | CCNH | SYBR | None | 25.387 | 7.1356 | | 0.5866 | | 0.66591 | | FALSE | 0.5 | TRUE | Amp | N |
| A10 | CCNO | SYBR | None | 22.151 | 3.8996 | | -0.3394 | | 1.26523 | | FALSE | 0.5 | TRUE | Amp | N |
| A11 | CDK7 | SYBR | None | 14.096 | -4.1554 | | -0.0044 | | 1.003055 | | FALSE | 0.5 | TRUE | Amp | N |
| A12 | DDB1 | SYBR | None | 20.243 | 1.9916 | | -0.3654 | | 1.288239 | | FALSE | 0.5 | TRUE | Amp | N |
| B1 | DDB2 | SYBR | None | 23.674 | 5.4226 | | 0.5276 | | 0.693708 | | FALSE | 0.5 | TRUE | Amp | N |
| B2 | DMC1 | SYBR | None | 27.523 | 9.2716 | | 0.1986 | | 0.871396 | | FALSE | 0.5 | TRUE | Amp | N |
| B3 | ERCC1 | SYBR | None | 22.291 | 4.0396 | | 0.1956 | | 0.87321 | | FALSE | 0.5 | TRUE | Amp | N |
| B4 | ERCC2 | SYBR | None | 23.549 | 5.2976 | | -0.4204 | | 1.338299 | | FALSE | 0.5 | TRUE | Amp | N |
| B5 | ERCC3 | SYBR | None | 21.427 | 3.1756 | | -0.0344 | | 1.024131 | | FALSE | 0.5 | TRUE | Amp | N |
| B6 | ERCC4 | SYBR | None | 27.16 | 8.9086 | | -0.4554 | | 1.371163 | | FALSE | 0.5 | TRUE | Amp | N |
| B7 | ERCC5 | SYBR | None | 25.116 | 6.8646 | | 0.1736 | | 0.886627 | | FALSE | 0.5 | TRUE | Amp | N |
| B8 | ERCC6 | SYBR | None | 18.179 | -0.0724 | | 0.4296 | | 0.742468 | | FALSE | 0.5 | TRUE | Amp | N |
| B9 | ERCC8 | SYBR | None | 22.227 | 3.9756 | | -0.4014 | | 1.320789 | | FALSE | 0.5 | TRUE | Amp | N |
| B10 | EXO1 | SYBR | None | 19.838 | 1.5866 | | -0.3394 | | 1.26523 | | FALSE | 0.5 | TRUE | Amp | N |
| B11 | FEN1 | SYBR | None | 17.203 | -1.0484 | | -0.3684 | | 1.29092 | | FALSE | 0.5 | TRUE | Amp | N |
| B12 | LIG1 | SYBR | None | 22.314 | 4.0626 | | 0.3466 | | 0.786435 | | FALSE | 0.5 | TRUE | Amp | N |
| C1 | LIG3 | SYBR | None | 25.888 | 7.6366 | | 0.5236 | | 0.695634 | | FALSE | 0.5 | TRUE | Amp | N |
| C2 | LIG4 | SYBR | None | 24.768 | 6.5166 | | -0.3164 | | 1.245219 | | FALSE | 0.5 | TRUE | Amp | N |
| C3 | MGMT | SYBR | None | 21.061 | 2.8096 | | -0.3544 | | 1.278454 | | FALSE | 0.5 | TRUE | Amp | N |
| C4 | MLH1 | SYBR | None | 22.173 | 3.9216 | | -0.0344 | | 1.024131 | | FALSE | 0.5 | TRUE | Amp | N |
| C5 | MLH3 | SYBR | None | 25.504 | 7.2526 | | 0.5056 | | 0.704367 | | FALSE | 0.5 | TRUE | Amp | N |
| C6 | MMS19 | SYBR | None | 25.585 | 7.3336 | | -0.0504 | | 1.035552 | | FALSE | 0.5 | TRUE | Amp | N |
| C7 | MPG | SYBR | None | 21.296 | 3.0446 | | -0.0284 | | 1.01988 | | FALSE | 0.5 | TRUE | Amp | N |
| C8 | MRE11A | SYBR | None | 18.173 | -0.0784 | | -0.0144 | | 1.010031 | | FALSE | 0.5 | TRUE | Amp | N |
| C9 | MSH2 | SYBR | None | 16.94 | -1.3114 | | 0.3196 | | 0.801292 | | FALSE | 0.5 | TRUE | Amp | N |
| C10 | MSH3 | SYBR | None | 20.046 | 1.7946 | | 0.4356 | | 0.739386 | | FALSE | 0.5 | TRUE | Amp | N |
| C11 | MSH4 | SYBR | None | 20.373 | 2.1216 | | -0.4104 | | 1.329054 | | FALSE | 0.5 | TRUE | Amp | N |
| C12 | MSH5 | SYBR | None | 27.895 | 9.6436 | | -0.7124 | | 1.638528 | | FALSE | 0.5 | TRUE | Amp | N |
| D1 | MSH6 | SYBR | None | 20.631 | 2.3796 | | -0.0224 | | 1.015648 | | FALSE | 0.5 | TRUE | Amp | N |
| D2 | MUTYH | SYBR | None | 24.227 | 5.9756 | | 0.5636 | | 0.676612 | | FALSE | 0.5 | TRUE | Amp | N |
| D3 | NEIL1 | SYBR | None | 27.039 | 8.7876 | | 0.1646 | | 0.892176 | | FALSE | 0.5 | TRUE | Amp | N |
| D4 | NEIL2 | SYBR | None | 24.107 | 5.8556 | | 0.2846 | | 0.820969 | | FALSE | 0.5 | TRUE | Amp | N |
| D5 | NEIL3 | SYBR | None | 21.288 | 3.0366 | | 0.1726 | | 0.887242 | | FALSE | 0.5 | TRUE | Amp | N |
| D6 | NTHL1 | SYBR | None | 20.34 | 2.0886 | | -0.5094 | | 1.423458 | | FALSE | 0.5 | TRUE | Amp | N |
| D7 | OGG1 | SYBR | None | 21.459 | 3.2076 | | -0.3894 | | 1.309849 | | FALSE | 0.5 | TRUE | Amp | N |
| D8 | PARP1 | SYBR | None | 20.581 | 2.3296 | | -0.3464 | | 1.271384 | | FALSE | 0.5 | TRUE | Amp | N |
| D9 | PARP2 | SYBR | None | 21.456 | 3.2046 | | 0.4916 | | 0.711236 | | FALSE | 0.5 | TRUE | Amp | N |
| D10 | PARP3 | SYBR | None | 24.13 | 5.8786 | | 0.1476 | | 0.902751 | | FALSE | 0.5 | TRUE | Amp | N |
| D11 | PMS1 | SYBR | None | 18.436 | 0.1846 | | 0.2806 | | 0.823249 | | FALSE | 0.5 | TRUE | Amp | N |
| D12 | PMS2 | SYBR | None | 21.738 | 3.4866 | | -0.3554 | | 1.27934 | | FALSE | 0.5 | TRUE | Amp | N |
| E1 | PNKP | SYBR | None | 24.116 | 5.8646 | | -0.3564 | | 1.280227 | | FALSE | 0.5 | TRUE | Amp | N |
| E2 | POLB | SYBR | None | 17.565 | -0.6864 | | -0.0414 | | 1.029112 | | FALSE | 0.5 | TRUE | Amp | N |
| E3 | POLD3 | SYBR | None | 20.195 | 1.9436 | | 0.1786 | | 0.88356 | | FALSE | 0.5 | TRUE | Amp | N |
| E4 | POLL | SYBR | None | 24.716 | 6.4646 | | -0.0524 | | 1.036989 | | FALSE | 0.5 | TRUE | Amp | N |
| E5 | PRKDC | SYBR | None | 24.213 | 5.9616 | | 0.5026 | | 0.705834 | | FALSE | 0.5 | TRUE | Amp | N |
| E6 | RAD18 | SYBR | None | 18.293 | 0.0416 | | 0.4736 | | 0.720165 | | FALSE | 0.5 | TRUE | Amp | N |
| E7 | RAD21 | SYBR | None | 18.381 | 0.1296 | | -0.0054 | | 1.00375 | | FALSE | 0.5 | TRUE | Amp | N |
| E8 | RAD23A | SYBR | None | 22.088 | 3.8366 | | -0.2964 | | 1.228076 | | FALSE | 0.5 | TRUE | Amp | N |
| E9 | RAD23B | SYBR | None | 25.144 | 6.8926 | | 0.5986 | | 0.660394 | | FALSE | 0.5 | TRUE | Amp | N |
| E10 | RAD50 | SYBR | None | 20.077 | 1.8256 | | 0.2096 | | 0.864777 | | FALSE | 0.5 | TRUE | Amp | N |
| E11 | RAD51 | SYBR | None | 21.243 | 2.9916 | | -0.0494 | | 1.034834 | | FALSE | 0.5 | TRUE | Amp | N |
| E12 | RAD51B | SYBR | None | 20.26 | 2.0086 | | 0.1866 | | 0.878674 | | FALSE | 0.5 | TRUE | Amp | N |
| F1 | RAD51C | SYBR | None | 19.124 | 0.8726 | | 0.1656 | | 0.891558 | | FALSE | 0.5 | TRUE | Amp | N |
| F2 | RAD51D | SYBR | None | 23.445 | 5.1936 | | -0.3284 | | 1.25562 | | FALSE | 0.5 | TRUE | Amp | N |
| F3 | RAD52 | SYBR | None | 25.788 | 7.5366 | | -0.0634 | | 1.044925 | | FALSE | 0.5 | TRUE | Amp | N |
| F4 | RAD54L | SYBR | None | 22.283 | 4.0316 | | -0.3624 | | 1.285563 | | FALSE | 0.5 | TRUE | Amp | N |
| F5 | RFC1 | SYBR | None | 24.373 | 6.1216 | | 0.5666 | | 0.675206 | | FALSE | 0.5 | TRUE | Amp | N |
| F6 | RPA1 | SYBR | None | 17.699 | -0.5524 | | 0.3616 | | 0.778301 | | FALSE | 0.5 | TRUE | Amp | N |
| F7 | RPA3 | SYBR | None | 24.075 | 5.8236 | | 0.4786 | | 0.717674 | | FALSE | 0.5 | TRUE | Amp | N |
| F8 | SLK | SYBR | None | 20.755 | 2.5036 | | -0.0554 | | 1.039147 | | FALSE | 0.5 | TRUE | Amp | N |
| F9 | SMUG1 | SYBR | None | 26.376 | 8.1246 | | -0.3484 | | 1.273148 | | FALSE | 0.5 | TRUE | Amp | N |
| F10 | TDG | SYBR | None | 21.555 | 3.3036 | | -0.2974 | | 1.228928 | | FALSE | 0.5 | TRUE | Amp | N |
| F11 | TOP3A | SYBR | None | 25.192 | 6.9406 | | 0.2566 | | 0.837058 | | FALSE | 0.5 | TRUE | Amp | N |
| F12 | TOP3B | SYBR | None | 27.153 | 8.9016 | | -0.3484 | | 1.273148 | | FALSE | 0.5 | TRUE | Amp | N |
| G1 | TREX1 | SYBR | None | 28.447 | 10.1956 | | 0.5616 | | 0.67755 | | FALSE | 0.5 | TRUE | Amp | N |
| G2 | UNG | SYBR | None | 20.266 | 2.0146 | | 0.4646 | | 0.724672 | | FALSE | 0.5 | TRUE | Amp | N |
| G3 | XAB2 | SYBR | None | 23.601 | 5.3496 | | -0.0444 | | 1.031254 | | FALSE | 0.5 | TRUE | Amp | N |
| G4 | XPA | SYBR | None | 31.977 | 13.7256 | | 0.6716 | | 0.62781 | | FALSE | 0.5 | TRUE | Amp | N |
| G5 | XPC | SYBR | None | 25.482 | 7.2306 | | 0.1296 | | 0.914085 | | FALSE | 0.5 | TRUE | Amp | N |
| G6 | XRCC1 | SYBR | None | 20.981 | 2.7296 | | 0.4516 | | 0.731231 | | FALSE | 0.5 | TRUE | Amp | N |
| G7 | XRCC2 | SYBR | None | 21.791 | 3.5396 | | 0.1376 | | 0.90903 | | FALSE | 0.5 | TRUE | Amp | N |
| G8 | XRCC3 | SYBR | None | 24.056 | 5.8046 | | 0.3516 | | 0.783714 | | FALSE | 0.5 | TRUE | Amp | N |
| G9 | XRCC4 | SYBR | None | 25.242 | 6.9906 | | 0.3006 | | 0.811915 | | FALSE | 0.5 | TRUE | Amp | N |
| G10 | XRCC5 | SYBR | None | 17.706 | -0.5454 | | 0.2736 | | 0.827253 | | FALSE | 0.5 | TRUE | Amp | N |
| G11 | XRCC6 | SYBR | None | 18.849 | 0.5976 | | -0.5084 | | 1.422472 | | FALSE | 0.5 | TRUE | Amp | N |
| G12 | XRCC6BP1 | SYBR | None | 24.331 | 6.0796 | | 0.5456 | | 0.685106 | | FALSE | 0.5 | TRUE | Amp | N |
| H1 | ACTB | SYBR | None | 18.183 |  | |  | |  | | FALSE | 0.5 | TRUE | Amp | N |
| H2 | B2M | SYBR | None | 16.822 |  | |  | |  | | FALSE | 0.5 | TRUE | Amp | N |
| H3 | GAPDH | SYBR | None | 20.689 |  | |  | |  | | FALSE | 0.5 | TRUE | Amp | N |
| H4 | HPRT1 | SYBR | None | 20.541 |  | |  | |  | | FALSE | 0.5 | TRUE | Amp | N |
| H5 | RPLP0 | SYBR | None | 15.022 |  | |  | |  | | FALSE | 0.5 | TRUE | Amp | N |
| POI 26 |  |  |  |  |  | |  | |  | |  |  |  |  |  |
| Well Position | Gene Symbol | Reporter | Quencher | CT | Delta Ct | | Delta Delta Ct | | 2^(-Delta Delta Ct) | | Automatic Ct Threshold | Ct Threshold | Automatic Baseline | Amp Status | MTP |
| A1 | APEX1 | SYBR | None | 18.699 | 0.3622 | | 0.411797 | | 0.751686 | | FALSE | 0.5 | TRUE | Amp | N |
| A2 | APEX2 | SYBR | None | 22.624 | 4.2872 | | 0.551828 | | 0.682155 | | FALSE | 0.5 | TRUE | Amp | N |
| A3 | ATM | SYBR | None | 23.091 | 4.7542 | | -0.39017 | | 1.310548 | | FALSE | 0.5 | TRUE | Amp | N |
| A4 | ATR | SYBR | None | 22.474 | 4.1372 | | -0.36889 | | 1.29136 | | FALSE | 0.5 | TRUE | Amp | N |
| A5 | ATXN3 | SYBR | None | 20.703 | 2.3662 | | 0.146182 | | 0.903639 | | FALSE | 0.5 | TRUE | Amp | N |
| A6 | BRCA1 | SYBR | None | 21.451 | 3.1142 | | -0.1334 | | 1.096877 | | FALSE | 0.5 | TRUE | Amp | N |
| A7 | BRCA2 | SYBR | None | 19.013 | 0.6762 | | 0.23225 | | 0.851306 | | FALSE | 0.5 | TRUE | Amp | N |
| A8 | BRIP1 | SYBR | None | 23.002 | 4.6652 | | -0.06831 | | 1.048487 | | FALSE | 0.5 | TRUE | Amp | N |
| A9 | CCNH | SYBR | None | 24.391 | 6.0542 | | -0.49489 | | 1.409214 | | FALSE | 0.5 | TRUE | Amp | N |
| A10 | CCNO | SYBR | None | 23.073 | 4.7362 | | 0.49745 | | 0.708358 | | FALSE | 0.5 | TRUE | Amp | N |
| A11 | CDK7 | SYBR | None | 14.483 | -3.8538 | | 0.2976 | | 0.813605 | | FALSE | 0.5 | TRUE | Amp | N |
| A12 | DDB1 | SYBR | None | 21.161 | 2.8242 | | 0.467238 | | 0.723348 | | FALSE | 0.5 | TRUE | Amp | N |
| B1 | DDB2 | SYBR | None | 23.097 | 4.7602 | | -0.13477 | | 1.097919 | | FALSE | 0.5 | TRUE | Amp | N |
| B2 | DMC1 | SYBR | None | 28.066 | 9.7292 | | 0.655846 | | 0.634703 | | FALSE | 0.5 | TRUE | Amp | N |
| B3 | ERCC1 | SYBR | None | 22.354 | 4.0172 | | 0.172808 | | 0.887115 | | FALSE | 0.5 | TRUE | Amp | N |
| B4 | ERCC2 | SYBR | None | 23.971 | 5.6342 | | -0.08393 | | 1.059898 | | FALSE | 0.5 | TRUE | Amp | N |
| B5 | ERCC3 | SYBR | None | 21.66 | 3.3232 | | 0.112877 | | 0.924742 | | FALSE | 0.5 | TRUE | Amp | N |
| B6 | ERCC4 | SYBR | None | 27.596 | 9.2592 | | -0.10444 | | 1.075077 | | FALSE | 0.5 | TRUE | Amp | N |
| B7 | ERCC5 | SYBR | None | 25.623 | 7.2862 | | 0.594872 | | 0.662103 | | FALSE | 0.5 | TRUE | Amp | N |
| B8 | ERCC6 | SYBR | None | 17.713 | -0.6238 | | -0.12163 | | 1.08796 | | FALSE | 0.5 | TRUE | Amp | N |
| B9 | ERCC8 | SYBR | None | 22.931 | 4.5942 | | 0.216849 | | 0.860443 | | FALSE | 0.5 | TRUE | Amp | N |
| B10 | EXO1 | SYBR | None | 20.1 | 1.7632 | | -0.16244 | | 1.119178 | | FALSE | 0.5 | TRUE | Amp | N |
| B11 | FEN1 | SYBR | None | 17.837 | -0.4998 | | 0.179951 | | 0.882733 | | FALSE | 0.5 | TRUE | Amp | N |
| B12 | LIG1 | SYBR | None | 22.009 | 3.6722 | | -0.044 | | 1.030969 | | FALSE | 0.5 | TRUE | Amp | N |
| C1 | LIG3 | SYBR | None | 25.314 | 6.9772 | | -0.13558 | | 1.098534 | | FALSE | 0.5 | TRUE | Amp | N |
| C2 | LIG4 | SYBR | None | 25.006 | 6.6692 | | -0.1637 | | 1.120155 | | FALSE | 0.5 | TRUE | Amp | N |
| C3 | MGMT | SYBR | None | 21.623 | 3.2862 | | 0.122002 | | 0.918911 | | FALSE | 0.5 | TRUE | Amp | N |
| C4 | MLH1 | SYBR | None | 22.714 | 4.3772 | | 0.421127 | | 0.746841 | | FALSE | 0.5 | TRUE | Amp | N |
| C5 | MLH3 | SYBR | None | 25.595 | 7.2582 | | 0.511502 | | 0.701492 | | FALSE | 0.5 | TRUE | Amp | N |
| C6 | MMS19 | SYBR | None | 25.581 | 7.2442 | | -0.13975 | | 1.101712 | | FALSE | 0.5 | TRUE | Amp | N |
| C7 | MPG | SYBR | None | 21.285 | 2.9482 | | -0.12437 | | 1.090033 | | FALSE | 0.5 | TRUE | Amp | N |
| C8 | MRE11A | SYBR | None | 18.441 | 0.1042 | | 0.168577 | | 0.88972 | | FALSE | 0.5 | TRUE | Amp | N |
| C9 | MSH2 | SYBR | None | 16.936 | -1.4008 | | 0.230075 | | 0.852591 | | FALSE | 0.5 | TRUE | Amp | N |
| C10 | MSH3 | SYBR | None | 20.197 | 1.8602 | | 0.500823 | | 0.706703 | | FALSE | 0.5 | TRUE | Amp | N |
| C11 | MSH4 | SYBR | None | 20.435 | 2.0982 | | -0.43321 | | 1.350233 | | FALSE | 0.5 | TRUE | Amp | N |
| C12 | MSH5 | SYBR | None | 29.283 | 10.9462 | | 0.590549 | | 0.66409 | | FALSE | 0.5 | TRUE | Amp | N |
| D1 | MSH6 | SYBR | None | 20.562 | 2.2252 | | -0.17622 | | 1.129918 | | FALSE | 0.5 | TRUE | Amp | N |
| D2 | MUTYH | SYBR | None | 24.322 | 5.9852 | | 0.573547 | | 0.671963 | | FALSE | 0.5 | TRUE | Amp | N |
| D3 | NEIL1 | SYBR | None | 26.409 | 8.0722 | | -0.551 | | 1.4651 | | FALSE | 0.5 | TRUE | Amp | N |
| D4 | NEIL2 | SYBR | None | 24.032 | 5.6952 | | 0.124592 | | 0.917263 | | FALSE | 0.5 | TRUE | Amp | N |
| D5 | NEIL3 | SYBR | None | 21.068 | 2.7312 | | -0.1323 | | 1.09604 | | FALSE | 0.5 | TRUE | Amp | N |
| D6 | NTHL1 | SYBR | None | 20.549 | 2.2122 | | -0.38614 | | 1.306893 | | FALSE | 0.5 | TRUE | Amp | N |
| D7 | OGG1 | SYBR | None | 21.754 | 3.4172 | | -0.17928 | | 1.132322 | | FALSE | 0.5 | TRUE | Amp | N |
| D8 | PARP1 | SYBR | None | 21.011 | 2.6742 | | -0.00214 | | 1.001482 | | FALSE | 0.5 | TRUE | Amp | N |
| D9 | PARP2 | SYBR | None | 21.538 | 3.2012 | | 0.48794 | | 0.713043 | | FALSE | 0.5 | TRUE | Amp | N |
| D10 | PARP3 | SYBR | None | 24.412 | 6.0752 | | 0.344167 | | 0.787763 | | FALSE | 0.5 | TRUE | Amp | N |
| D11 | PMS1 | SYBR | None | 18.536 | 0.1992 | | 0.295232 | | 0.814942 | | FALSE | 0.5 | TRUE | Amp | N |
| D12 | PMS2 | SYBR | None | 22.025 | 3.6882 | | -0.15323 | | 1.112057 | | FALSE | 0.5 | TRUE | Amp | N |
| E1 | PNKP | SYBR | None | 24.374 | 6.0372 | | -0.18363 | | 1.135737 | | FALSE | 0.5 | TRUE | Amp | N |
| E2 | POLB | SYBR | None | 17.619 | -0.7178 | | -0.07227 | | 1.051367 | | FALSE | 0.5 | TRUE | Amp | N |
| E3 | POLD3 | SYBR | None | 20.515 | 2.1782 | | 0.413344 | | 0.750881 | | FALSE | 0.5 | TRUE | Amp | N |
| E4 | POLL | SYBR | None | 24.781 | 6.4442 | | -0.07247 | | 1.051518 | | FALSE | 0.5 | TRUE | Amp | N |
| E5 | PRKDC | SYBR | None | 23.779 | 5.4422 | | -0.01687 | | 1.011763 | | FALSE | 0.5 | TRUE | Amp | N |
| E6 | RAD18 | SYBR | None | 18.341 | 0.0042 | | 0.436219 | | 0.739069 | | FALSE | 0.5 | TRUE | Amp | N |
| E7 | RAD21 | SYBR | None | 18.896 | 0.5592 | | 0.423906 | | 0.745404 | | FALSE | 0.5 | TRUE | Amp | N |
| E8 | RAD23A | SYBR | None | 21.958 | 3.6212 | | -0.51139 | | 1.425424 | | FALSE | 0.5 | TRUE | Amp | N |
| E9 | RAD23B | SYBR | None | 24.106 | 5.7692 | | -0.52502 | | 1.438951 | | FALSE | 0.5 | TRUE | Amp | N |
| E10 | RAD50 | SYBR | None | 19.842 | 1.5052 | | -0.11087 | | 1.079876 | | FALSE | 0.5 | TRUE | Amp | N |
| E11 | RAD51 | SYBR | None | 21.496 | 3.1592 | | 0.118393 | | 0.921213 | | FALSE | 0.5 | TRUE | Amp | N |
| E12 | RAD51B | SYBR | None | 20.573 | 2.2362 | | 0.414375 | | 0.750344 | | FALSE | 0.5 | TRUE | Amp | N |
| F1 | RAD51C | SYBR | None | 19.351 | 1.0142 | | 0.306822 | | 0.808421 | | FALSE | 0.5 | TRUE | Amp | N |
| F2 | RAD51D | SYBR | None | 23.486 | 5.1492 | | -0.37301 | | 1.295051 | | FALSE | 0.5 | TRUE | Amp | N |
| F3 | RAD52 | SYBR | None | 25.497 | 7.1602 | | -0.43993 | | 1.356536 | | FALSE | 0.5 | TRUE | Amp | N |
| F4 | RAD54L | SYBR | None | 22.353 | 4.0162 | | -0.37758 | | 1.299164 | | FALSE | 0.5 | TRUE | Amp | N |
| F5 | RFC1 | SYBR | None | 23.505 | 5.1682 | | -0.3866 | | 1.307307 | | FALSE | 0.5 | TRUE | Amp | N |
| F6 | RPA1 | SYBR | None | 17.606 | -0.7308 | | 0.182847 | | 0.880963 | | FALSE | 0.5 | TRUE | Amp | N |
| F7 | RPA3 | SYBR | None | 23.233 | 4.8962 | | -0.44905 | | 1.365145 | | FALSE | 0.5 | TRUE | Amp | N |
| F8 | SLK | SYBR | None | 21.411 | 3.0742 | | 0.515322 | | 0.699637 | | FALSE | 0.5 | TRUE | Amp | N |
| F9 | SMUG1 | SYBR | None | 26.195 | 7.8582 | | -0.61435 | | 1.530869 | | FALSE | 0.5 | TRUE | Amp | N |
| F10 | TDG | SYBR | None | 21.558 | 3.2212 | | -0.37942 | | 1.300815 | | FALSE | 0.5 | TRUE | Amp | N |
| F11 | TOP3A | SYBR | None | 25.626 | 7.2892 | | 0.605029 | | 0.657458 | | FALSE | 0.5 | TRUE | Amp | N |
| F12 | TOP3B | SYBR | None | 27.8 | 9.4632 | | 0.213717 | | 0.862313 | | FALSE | 0.5 | TRUE | Amp | N |
| G1 | TREX1 | SYBR | None | 27.834 | 9.4972 | | -0.13636 | | 1.099127 | | FALSE | 0.5 | TRUE | Amp | N |
| G2 | UNG | SYBR | None | 20.212 | 1.8752 | | 0.325712 | | 0.797904 | | FALSE | 0.5 | TRUE | Amp | N |
| G3 | XAB2 | SYBR | None | 23.227 | 4.8902 | | -0.5039 | | 1.418045 | | FALSE | 0.5 | TRUE | Amp | N |
| G4 | XPA | SYBR | None | 30.803 | 12.4662 | | -0.58728 | | 1.502407 | | FALSE | 0.5 | TRUE | Amp | N |
| G5 | XPC | SYBR | None | 26.053 | 7.7162 | | 0.615452 | | 0.652725 | | FALSE | 0.5 | TRUE | Amp | N |
| G6 | XRCC1 | SYBR | None | 20.224 | 1.8872 | | -0.3909 | | 1.311213 | | FALSE | 0.5 | TRUE | Amp | N |
| G7 | XRCC2 | SYBR | None | 21.339 | 3.0022 | | -0.39989 | | 1.319405 | | FALSE | 0.5 | TRUE | Amp | N |
| G8 | XRCC3 | SYBR | None | 23.915 | 5.5782 | | 0.125327 | | 0.916796 | | FALSE | 0.5 | TRUE | Amp | N |
| G9 | XRCC4 | SYBR | None | 25.007 | 6.6702 | | -0.01989 | | 1.013882 | | FALSE | 0.5 | TRUE | Amp | N |
| G10 | XRCC5 | SYBR | None | 17.724 | -0.6128 | | 0.206307 | | 0.866753 | | FALSE | 0.5 | TRUE | Amp | N |
| G11 | XRCC6 | SYBR | None | 18.314 | -0.0228 | | -1.12841 | | 2.18617 | | FALSE | 0.5 | TRUE | Amp | N |
| G12 | XRCC6BP1 | SYBR | None | 23.47 | 5.1332 | | -0.40092 | | 1.32035 | | FALSE | 0.5 | TRUE | Amp | N |
| H1 | ACTB | SYBR | None | 18.309 |  | |  | |  | | FALSE | 0.5 | TRUE | Amp | N |
| H2 | B2M | SYBR | None | 16.812 |  | |  | |  | | FALSE | 0.5 | TRUE | Amp | N |
| H3 | GAPDH | SYBR | None | 20.883 |  | |  | |  | | FALSE | 0.5 | TRUE | Amp | N |
| H4 | HPRT1 | SYBR | None | 20.581 |  | |  | |  | | FALSE | 0.5 | TRUE | Amp | N |
| H5 | RPLP0 | SYBR | None | 15.099 |  | |  | |  | | FALSE | 0.5 | TRUE | Amp | N |
| POI 27 | Dead during in vitro culture. | | |  |  | |  | |  | |  |  |  |  |  |
| POI 28 |  |  |  |  |  | |  | |  | |  |  |  |  |  |
| Well Position | Gene Symbol | Reporter | Quencher | CT | Delta Ct | | Delta Delta Ct | | 2^(-Delta Delta Ct) | | Automatic Ct Threshold | Ct Threshold | Automatic Baseline | Amp Status | MTP |
| A1 | APEX1 | SYBR | None | 18.527 | 0.1716 | | 0.221197 | | 0.857853 | | FALSE | 0.5 | TRUE | Amp | N |
| A2 | APEX2 | SYBR | None | 22.556 | 4.2006 | | 0.465228 | | 0.724357 | | FALSE | 0.5 | TRUE | Amp | N |
| A3 | ATM | SYBR | None | 23.321 | 4.9656 | | -0.17877 | | 1.131919 | | FALSE | 0.5 | TRUE | Amp | N |
| A4 | ATR | SYBR | None | 23.363 | 5.0076 | | 0.501508 | | 0.706368 | | FALSE | 0.5 | TRUE | Amp | N |
| A5 | ATXN3 | SYBR | None | 20.542 | 2.1866 | | -0.03342 | | 1.023434 | | FALSE | 0.5 | TRUE | Amp | N |
| A6 | BRCA1 | SYBR | None | 22.151 | 3.7956 | | 0.547998 | | 0.683969 | | FALSE | 0.5 | TRUE | Amp | N |
| A7 | BRCA2 | SYBR | None | 18.991 | 0.6356 | | 0.19165 | | 0.875604 | | FALSE | 0.5 | TRUE | Amp | N |
| A8 | BRIP1 | SYBR | None | 23.004 | 4.6486 | | -0.08491 | | 1.060621 | | FALSE | 0.5 | TRUE | Amp | N |
| A9 | CCNH | SYBR | None | 24.716 | 6.3606 | | -0.18849 | | 1.139571 | | FALSE | 0.5 | TRUE | Amp | N |
| A10 | CCNO | SYBR | None | 22.205 | 3.8496 | | -0.38915 | | 1.309622 | | FALSE | 0.5 | TRUE | Amp | N |
| A11 | CDK7 | SYBR | None | 14.159 | -4.1964 | | -0.045 | | 1.031683 | | FALSE | 0.5 | TRUE | Amp | N |
| A12 | DDB1 | SYBR | None | 20.684 | 2.3286 | | -0.02836 | | 1.019853 | | FALSE | 0.5 | TRUE | Amp | N |
| B1 | DDB2 | SYBR | None | 23.106 | 4.7506 | | -0.14437 | | 1.105249 | | FALSE | 0.5 | TRUE | Amp | N |
| B2 | DMC1 | SYBR | None | 28.083 | 9.7276 | | 0.654246 | | 0.635408 | | FALSE | 0.5 | TRUE | Amp | N |
| B3 | ERCC1 | SYBR | None | 22.377 | 4.0216 | | 0.177208 | | 0.884413 | | FALSE | 0.5 | TRUE | Amp | N |
| B4 | ERCC2 | SYBR | None | 23.996 | 5.6406 | | -0.07753 | | 1.055207 | | FALSE | 0.5 | TRUE | Amp | N |
| B5 | ERCC3 | SYBR | None | 21.684 | 3.3286 | | 0.118277 | | 0.921287 | | FALSE | 0.5 | TRUE | Amp | N |
| B6 | ERCC4 | SYBR | None | 27.576 | 9.2206 | | -0.14304 | | 1.104229 | | FALSE | 0.5 | TRUE | Amp | N |
| B7 | ERCC5 | SYBR | None | 24.895 | 6.5396 | | -0.15173 | | 1.110899 | | FALSE | 0.5 | TRUE | Amp | N |
| B8 | ERCC6 | SYBR | None | 17.742 | -0.6134 | | -0.11123 | | 1.080145 | | FALSE | 0.5 | TRUE | Amp | N |
| B9 | ERCC8 | SYBR | None | 22.252 | 3.8966 | | -0.48075 | | 1.39547 | | FALSE | 0.5 | TRUE | Amp | N |
| B10 | EXO1 | SYBR | None | 20.173 | 1.8176 | | -0.10804 | | 1.077763 | | FALSE | 0.5 | TRUE | Amp | N |
| B11 | FEN1 | SYBR | None | 17.658 | -0.6974 | | -0.01765 | | 1.012309 | | FALSE | 0.5 | TRUE | Amp | N |
| B12 | LIG1 | SYBR | None | 22.004 | 3.6486 | | -0.0676 | | 1.047973 | | FALSE | 0.5 | TRUE | Amp | N |
| C1 | LIG3 | SYBR | None | 25.296 | 6.9406 | | -0.17218 | | 1.126759 | | FALSE | 0.5 | TRUE | Amp | N |
| C2 | LIG4 | SYBR | None | 25.712 | 7.3566 | | 0.523701 | | 0.695585 | | FALSE | 0.5 | TRUE | Amp | N |
| C3 | MGMT | SYBR | None | 21.319 | 2.9636 | | -0.2006 | | 1.149174 | | FALSE | 0.5 | TRUE | Amp | N |
| C4 | MLH1 | SYBR | None | 22.242 | 3.8866 | | -0.06947 | | 1.049334 | | FALSE | 0.5 | TRUE | Amp | N |
| C5 | MLH3 | SYBR | None | 24.986 | 6.6306 | | -0.1161 | | 1.0838 | | FALSE | 0.5 | TRUE | Amp | N |
| C6 | MMS19 | SYBR | None | 25.328 | 6.9726 | | -0.41135 | | 1.329927 | | FALSE | 0.5 | TRUE | Amp | N |
| C7 | MPG | SYBR | None | 21.031 | 2.6756 | | -0.39697 | | 1.316742 | | FALSE | 0.5 | TRUE | Amp | N |
| C8 | MRE11A | SYBR | None | 18.707 | 0.3516 | | 0.415977 | | 0.749512 | | FALSE | 0.5 | TRUE | Amp | N |
| C9 | MSH2 | SYBR | None | 16.926 | -1.4294 | | 0.201475 | | 0.869661 | | FALSE | 0.5 | TRUE | Amp | N |
| C10 | MSH3 | SYBR | None | 20.201 | 1.8456 | | 0.486223 | | 0.713891 | | FALSE | 0.5 | TRUE | Amp | N |
| C11 | MSH4 | SYBR | None | 21.31 | 2.9546 | | 0.423192 | | 0.745773 | | FALSE | 0.5 | TRUE | Amp | N |
| C12 | MSH5 | SYBR | None | 28.205 | 9.8496 | | -0.50605 | | 1.420158 | | FALSE | 0.5 | TRUE | Amp | N |
| D1 | MSH6 | SYBR | None | 20.894 | 2.5386 | | 0.137182 | | 0.909294 | | FALSE | 0.5 | TRUE | Amp | N |
| D2 | MUTYH | SYBR | None | 23.628 | 5.2726 | | -0.13905 | | 1.101182 | | FALSE | 0.5 | TRUE | Amp | N |
| D3 | NEIL1 | SYBR | None | 26.441 | 8.0856 | | -0.5376 | | 1.451555 | | FALSE | 0.5 | TRUE | Amp | N |
| D4 | NEIL2 | SYBR | None | 23.719 | 5.3636 | | -0.20701 | | 1.154292 | | FALSE | 0.5 | TRUE | Amp | N |
| D5 | NEIL3 | SYBR | None | 21.408 | 3.0526 | | 0.1891 | | 0.877153 | | FALSE | 0.5 | TRUE | Amp | N |
| D6 | NTHL1 | SYBR | None | 20.557 | 2.2016 | | -0.39674 | | 1.31653 | | FALSE | 0.5 | TRUE | Amp | N |
| D7 | OGG1 | SYBR | None | 22.448 | 4.0926 | | 0.496115 | | 0.709013 | | FALSE | 0.5 | TRUE | Amp | N |
| D8 | PARP1 | SYBR | None | 21.255 | 2.8996 | | 0.223264 | | 0.856625 | | FALSE | 0.5 | TRUE | Amp | N |
| D9 | PARP2 | SYBR | None | 21.552 | 3.1966 | | 0.48334 | | 0.71532 | | FALSE | 0.5 | TRUE | Amp | N |
| D10 | PARP3 | SYBR | None | 23.896 | 5.5406 | | -0.19043 | | 1.141106 | | FALSE | 0.5 | TRUE | Amp | N |
| D11 | PMS1 | SYBR | None | 18.543 | 0.1876 | | 0.283632 | | 0.82152 | | FALSE | 0.5 | TRUE | Amp | N |
| D12 | PMS2 | SYBR | None | 22.755 | 4.3996 | | 0.55817 | | 0.679163 | | FALSE | 0.5 | TRUE | Amp | N |
| E1 | PNKP | SYBR | None | 24.144 | 5.7886 | | -0.43223 | | 1.349316 | | FALSE | 0.5 | TRUE | Amp | N |
| E2 | POLB | SYBR | None | 17.906 | -0.4494 | | 0.196133 | | 0.872887 | | FALSE | 0.5 | TRUE | Amp | N |
| E3 | POLD3 | SYBR | None | 20.048 | 1.6926 | | -0.07226 | | 1.051359 | | FALSE | 0.5 | TRUE | Amp | N |
| E4 | POLL | SYBR | None | 24.861 | 6.5056 | | -0.01107 | | 1.007705 | | FALSE | 0.5 | TRUE | Amp | N |
| E5 | PRKDC | SYBR | None | 23.679 | 5.3236 | | -0.13547 | | 1.098452 | | FALSE | 0.5 | TRUE | Amp | N |
| E6 | RAD18 | SYBR | None | 17.923 | -0.4324 | | -0.00038 | | 1.000264 | | FALSE | 0.5 | TRUE | Amp | N |
| E7 | RAD21 | SYBR | None | 18.075 | -0.2804 | | -0.41569 | | 1.33394 | | FALSE | 0.5 | TRUE | Amp | N |
| E8 | RAD23A | SYBR | None | 22.317 | 3.9616 | | -0.17099 | | 1.125831 | | FALSE | 0.5 | TRUE | Amp | N |
| E9 | RAD23B | SYBR | None | 24.165 | 5.8096 | | -0.48462 | | 1.399215 | | FALSE | 0.5 | TRUE | Amp | N |
| E10 | RAD50 | SYBR | None | 19.602 | 1.2466 | | -0.36947 | | 1.291874 | | FALSE | 0.5 | TRUE | Amp | N |
| E11 | RAD51 | SYBR | None | 21.84 | 3.4846 | | 0.443793 | | 0.735199 | | FALSE | 0.5 | TRUE | Amp | N |
| E12 | RAD51B | SYBR | None | 20.612 | 2.2566 | | 0.434775 | | 0.739809 | | FALSE | 0.5 | TRUE | Amp | N |
| F1 | RAD51C | SYBR | None | 19.327 | 0.9716 | | 0.264222 | | 0.832648 | | FALSE | 0.5 | TRUE | Amp | N |
| F2 | RAD51D | SYBR | None | 23.684 | 5.3286 | | -0.19361 | | 1.143621 | | FALSE | 0.5 | TRUE | Amp | N |
| F3 | RAD52 | SYBR | None | 25.402 | 7.0466 | | -0.55353 | | 1.46767 | | FALSE | 0.5 | TRUE | Amp | N |
| F4 | RAD54L | SYBR | None | 22.712 | 4.3566 | | -0.03718 | | 1.026108 | | FALSE | 0.5 | TRUE | Amp | N |
| F5 | RFC1 | SYBR | None | 23.508 | 5.1526 | | -0.4022 | | 1.32152 | | FALSE | 0.5 | TRUE | Amp | N |
| F6 | RPA1 | SYBR | None | 17.774 | -0.5814 | | 0.332247 | | 0.794298 | | FALSE | 0.5 | TRUE | Amp | N |
| F7 | RPA3 | SYBR | None | 23.305 | 4.9496 | | -0.39565 | | 1.315539 | | FALSE | 0.5 | TRUE | Amp | N |
| F8 | SLK | SYBR | None | 20.831 | 2.4756 | | -0.08328 | | 1.059422 | | FALSE | 0.5 | TRUE | Amp | N |
| F9 | SMUG1 | SYBR | None | 26.313 | 7.9576 | | -0.51495 | | 1.428946 | | FALSE | 0.5 | TRUE | Amp | N |
| F10 | TDG | SYBR | None | 22.108 | 3.7526 | | 0.151984 | | 0.900012 | | FALSE | 0.5 | TRUE | Amp | N |
| F11 | TOP3A | SYBR | None | 25.009 | 6.6536 | | -0.03057 | | 1.021417 | | FALSE | 0.5 | TRUE | Amp | N |
| F12 | TOP3B | SYBR | None | 27.133 | 8.7776 | | -0.47188 | | 1.386918 | | FALSE | 0.5 | TRUE | Amp | N |
| G1 | TREX1 | SYBR | None | 27.547 | 9.1916 | | -0.44196 | | 1.358447 | | FALSE | 0.5 | TRUE | Amp | N |
| G2 | UNG | SYBR | None | 19.528 | 1.1726 | | -0.37689 | | 1.298538 | | FALSE | 0.5 | TRUE | Amp | N |
| G3 | XAB2 | SYBR | None | 24.288 | 5.9326 | | 0.538496 | | 0.688488 | | FALSE | 0.5 | TRUE | Amp | N |
| G4 | XPA | SYBR | None | 30.782 | 12.4266 | | -0.62688 | | 1.544217 | | FALSE | 0.5 | TRUE | Amp | N |
| G5 | XPC | SYBR | None | 24.929 | 6.5736 | | -0.52715 | | 1.441077 | | FALSE | 0.5 | TRUE | Amp | N |
| G6 | XRCC1 | SYBR | None | 20.563 | 2.2076 | | -0.0705 | | 1.050082 | | FALSE | 0.5 | TRUE | Amp | N |
| G7 | XRCC2 | SYBR | None | 21.914 | 3.5586 | | 0.156512 | | 0.897191 | | FALSE | 0.5 | TRUE | Amp | N |
| G8 | XRCC3 | SYBR | None | 24.36 | 6.0046 | | 0.551727 | | 0.682203 | | FALSE | 0.5 | TRUE | Amp | N |
| G9 | XRCC4 | SYBR | None | 25.603 | 7.2476 | | 0.55751 | | 0.679474 | | FALSE | 0.5 | TRUE | Amp | N |
| G10 | XRCC5 | SYBR | None | 17.849 | -0.5064 | | 0.312707 | | 0.805129 | | FALSE | 0.5 | TRUE | Amp | N |
| G11 | XRCC6 | SYBR | None | 19.653 | 1.2976 | | 0.191994 | | 0.875395 | | FALSE | 0.5 | TRUE | Amp | N |
| G12 | XRCC6BP1 | SYBR | None | 24.405 | 6.0496 | | 0.51548 | | 0.69956 | | FALSE | 0.5 | TRUE | Amp | N |
| H1 | ACTB | SYBR | None | 18.106 |  | |  | |  | | FALSE | 0.5 | TRUE | Amp | N |
| H2 | B2M | SYBR | None | 16.92 |  | |  | |  | | FALSE | 0.5 | TRUE | Amp | N |
| H3 | GAPDH | SYBR | None | 20.773 |  | |  | |  | | FALSE | 0.5 | TRUE | Amp | N |
| H4 | HPRT1 | SYBR | None | 20.683 |  | |  | |  | | FALSE | 0.5 | TRUE | Amp | N |
| H5 | RPLP0 | SYBR | None | 15.295 |  | |  | |  | | FALSE | 0.5 | TRUE | Amp | N |
| POI 29 |  |  |  |  |  | |  | |  | |  |  |  |  |  |
| Well Position | Gene Symbol | Reporter | Quencher | CT | Delta Ct | | Delta Delta Ct | | 2^(-Delta Delta Ct) | | Automatic Ct Threshold | Ct Threshold | Automatic Baseline | Amp Status | MTP |
| A1 | APEX1 | SYBR | None | 18.283 | -0.1558 | | -0.1062 | | 1.076391 | | FALSE | 0.5 | TRUE | Amp | N |
| A2 | APEX2 | SYBR | None | 21.694 | 3.2552 | | -0.48017 | | 1.39491 | | FALSE | 0.5 | TRUE | Amp | N |
| A3 | ATM | SYBR | None | 24.11 | 5.6712 | | 0.526829 | | 0.694078 | | FALSE | 0.5 | TRUE | Amp | N |
| A4 | ATR | SYBR | None | 22.446 | 4.0072 | | -0.49889 | | 1.413128 | | FALSE | 0.5 | TRUE | Amp | N |
| A5 | ATXN3 | SYBR | None | 20.914 | 2.4752 | | 0.255182 | | 0.837881 | | FALSE | 0.5 | TRUE | Amp | N |
| A6 | BRCA1 | SYBR | None | 21.229 | 2.7902 | | -0.4574 | | 1.373067 | | FALSE | 0.5 | TRUE | Amp | N |
| A7 | BRCA2 | SYBR | None | 18.695 | 0.2562 | | -0.18775 | | 1.138986 | | FALSE | 0.5 | TRUE | Amp | N |
| A8 | BRIP1 | SYBR | None | 23.696 | 5.2572 | | 0.523691 | | 0.69559 | | FALSE | 0.5 | TRUE | Amp | N |
| A9 | CCNH | SYBR | None | 25.494 | 7.0552 | | 0.506109 | | 0.704119 | | FALSE | 0.5 | TRUE | Amp | N |
| A10 | CCNO | SYBR | None | 23.157 | 4.7182 | | 0.47945 | | 0.717251 | | FALSE | 0.5 | TRUE | Amp | N |
| A11 | CDK7 | SYBR | None | 14.542 | -3.8968 | | 0.2546 | | 0.83822 | | FALSE | 0.5 | TRUE | Amp | N |
| A12 | DDB1 | SYBR | None | 20.916 | 2.4772 | | 0.120238 | | 0.920036 | | FALSE | 0.5 | TRUE | Amp | N |
| B1 | DDB2 | SYBR | None | 23.168 | 4.7292 | | -0.16577 | | 1.121766 | | FALSE | 0.5 | TRUE | Amp | N |
| B2 | DMC1 | SYBR | None | 27.63 | 9.1912 | | 0.117846 | | 0.921563 | | FALSE | 0.5 | TRUE | Amp | N |
| B3 | ERCC1 | SYBR | None | 22.378 | 3.9392 | | 0.094808 | | 0.936397 | | FALSE | 0.5 | TRUE | Amp | N |
| B4 | ERCC2 | SYBR | None | 23.76 | 5.3212 | | -0.39693 | | 1.316699 | | FALSE | 0.5 | TRUE | Amp | N |
| B5 | ERCC3 | SYBR | None | 21.454 | 3.0152 | | -0.19512 | | 1.144822 | | FALSE | 0.5 | TRUE | Amp | N |
| B6 | ERCC4 | SYBR | None | 28.389 | 9.9502 | | 0.586561 | | 0.665929 | | FALSE | 0.5 | TRUE | Amp | N |
| B7 | ERCC5 | SYBR | None | 25.006 | 6.5672 | | -0.12413 | | 1.089849 | | FALSE | 0.5 | TRUE | Amp | N |
| B8 | ERCC6 | SYBR | None | 17.719 | -0.7198 | | -0.21763 | | 1.162818 | | FALSE | 0.5 | TRUE | Amp | N |
| B9 | ERCC8 | SYBR | None | 22.348 | 3.9092 | | -0.46815 | | 1.383336 | | FALSE | 0.5 | TRUE | Amp | N |
| B10 | EXO1 | SYBR | None | 20.168 | 1.7292 | | -0.19644 | | 1.145867 | | FALSE | 0.5 | TRUE | Amp | N |
| B11 | FEN1 | SYBR | None | 17.974 | -0.4648 | | 0.214951 | | 0.861576 | | FALSE | 0.5 | TRUE | Amp | N |
| B12 | LIG1 | SYBR | None | 22.009 | 3.5702 | | -0.146 | | 1.106498 | | FALSE | 0.5 | TRUE | Amp | N |
| C1 | LIG3 | SYBR | None | 24.937 | 6.4982 | | -0.61458 | | 1.531111 | | FALSE | 0.5 | TRUE | Amp | N |
| C2 | LIG4 | SYBR | None | 24.812 | 6.3732 | | -0.4597 | | 1.375255 | | FALSE | 0.5 | TRUE | Amp | N |
| C3 | MGMT | SYBR | None | 21.391 | 2.9522 | | -0.212 | | 1.158291 | | FALSE | 0.5 | TRUE | Amp | N |
| C4 | MLH1 | SYBR | None | 22.542 | 4.1032 | | 0.147127 | | 0.903047 | | FALSE | 0.5 | TRUE | Amp | N |
| C5 | MLH3 | SYBR | None | 24.978 | 6.5392 | | -0.2075 | | 1.154684 | | FALSE | 0.5 | TRUE | Amp | N |
| C6 | MMS19 | SYBR | None | 25.215 | 6.7762 | | -0.60775 | | 1.523878 | | FALSE | 0.5 | TRUE | Amp | N |
| C7 | MPG | SYBR | None | 21.963 | 3.5242 | | 0.451628 | | 0.731217 | | FALSE | 0.5 | TRUE | Amp | N |
| C8 | MRE11A | SYBR | None | 18.815 | 0.3762 | | 0.440577 | | 0.73684 | | FALSE | 0.5 | TRUE | Amp | N |
| C9 | MSH2 | SYBR | None | 16.806 | -1.6328 | | -0.00192 | | 1.001335 | | FALSE | 0.5 | TRUE | Amp | N |
| C10 | MSH3 | SYBR | None | 19.994 | 1.5552 | | 0.195823 | | 0.873074 | | FALSE | 0.5 | TRUE | Amp | N |
| C11 | MSH4 | SYBR | None | 21.39 | 2.9512 | | 0.419792 | | 0.747532 | | FALSE | 0.5 | TRUE | Amp | N |
| C12 | MSH5 | SYBR | None | 28.29 | 9.8512 | | -0.50445 | | 1.418584 | | FALSE | 0.5 | TRUE | Amp | N |
| D1 | MSH6 | SYBR | None | 20.583 | 2.1442 | | -0.25722 | | 1.195172 | | FALSE | 0.5 | TRUE | Amp | N |
| D2 | MUTYH | SYBR | None | 23.719 | 5.2802 | | -0.13145 | | 1.095396 | | FALSE | 0.5 | TRUE | Amp | N |
| D3 | NEIL1 | SYBR | None | 26.432 | 7.9932 | | -0.63 | | 1.547564 | | FALSE | 0.5 | TRUE | Amp | N |
| D4 | NEIL2 | SYBR | None | 23.882 | 5.4432 | | -0.12741 | | 1.092329 | | FALSE | 0.5 | TRUE | Amp | N |
| D5 | NEIL3 | SYBR | None | 20.869 | 2.4302 | | -0.4333 | | 1.350319 | | FALSE | 0.5 | TRUE | Amp | N |
| D6 | NTHL1 | SYBR | None | 21.141 | 2.7022 | | 0.103859 | | 0.93054 | | FALSE | 0.5 | TRUE | Amp | N |
| D7 | OGG1 | SYBR | None | 21.867 | 3.4282 | | -0.16828 | | 1.123722 | | FALSE | 0.5 | TRUE | Amp | N |
| D8 | PARP1 | SYBR | None | 21.317 | 2.8782 | | 0.201864 | | 0.869426 | | FALSE | 0.5 | TRUE | Amp | N |
| D9 | PARP2 | SYBR | None | 21.143 | 2.7042 | | -0.00906 | | 1.0063 | | FALSE | 0.5 | TRUE | Amp | N |
| D10 | PARP3 | SYBR | None | 24.68 | 6.2412 | | 0.510167 | | 0.702141 | | FALSE | 0.5 | TRUE | Amp | N |
| D11 | PMS1 | SYBR | None | 17.871 | -0.5678 | | -0.47177 | | 1.386808 | | FALSE | 0.5 | TRUE | Amp | N |
| D12 | PMS2 | SYBR | None | 21.808 | 3.3692 | | -0.47223 | | 1.387252 | | FALSE | 0.5 | TRUE | Amp | N |
| E1 | PNKP | SYBR | None | 23.98 | 5.5412 | | -0.67963 | | 1.601727 | | FALSE | 0.5 | TRUE | Amp | N |
| E2 | POLB | SYBR | None | 18.676 | 0.2372 | | 0.882733 | | 0.542339 | | FALSE | 0.5 | TRUE | Amp | N |
| E3 | POLD3 | SYBR | None | 20.345 | 1.9062 | | 0.141344 | | 0.906674 | | FALSE | 0.5 | TRUE | Amp | N |
| E4 | POLL | SYBR | None | 24.404 | 5.9652 | | -0.55147 | | 1.465582 | | FALSE | 0.5 | TRUE | Amp | N |
| E5 | PRKDC | SYBR | None | 23.734 | 5.2952 | | -0.16387 | | 1.12029 | | FALSE | 0.5 | TRUE | Amp | N |
| E6 | RAD18 | SYBR | None | 17.862 | -0.5768 | | -0.14478 | | 1.105563 | | FALSE | 0.5 | TRUE | Amp | N |
| E7 | RAD21 | SYBR | None | 18.969 | 0.5302 | | 0.394906 | | 0.760539 | | FALSE | 0.5 | TRUE | Amp | N |
| E8 | RAD23A | SYBR | None | 22.365 | 3.9262 | | -0.20639 | | 1.153798 | | FALSE | 0.5 | TRUE | Amp | N |
| E9 | RAD23B | SYBR | None | 24.236 | 5.7972 | | -0.49702 | | 1.411293 | | FALSE | 0.5 | TRUE | Amp | N |
| E10 | RAD50 | SYBR | None | 19.538 | 1.0992 | | -0.51687 | | 1.430843 | | FALSE | 0.5 | TRUE | Amp | N |
| E11 | RAD51 | SYBR | None | 21.701 | 3.2622 | | 0.221393 | | 0.857737 | | FALSE | 0.5 | TRUE | Amp | N |
| E12 | RAD51B | SYBR | None | 20.689 | 2.2502 | | 0.428375 | | 0.743098 | | FALSE | 0.5 | TRUE | Amp | N |
| F1 | RAD51C | SYBR | None | 19.269 | 0.8302 | | 0.122822 | | 0.918389 | | FALSE | 0.5 | TRUE | Amp | N |
| F2 | RAD51D | SYBR | None | 23.311 | 4.8722 | | -0.65001 | | 1.569178 | | FALSE | 0.5 | TRUE | Amp | N |
| F3 | RAD52 | SYBR | None | 26.008 | 7.5692 | | -0.03093 | | 1.021669 | | FALSE | 0.5 | TRUE | Amp | N |
| F4 | RAD54L | SYBR | None | 22.357 | 3.9182 | | -0.47558 | | 1.39048 | | FALSE | 0.5 | TRUE | Amp | N |
| F5 | RFC1 | SYBR | None | 23.496 | 5.0572 | | -0.4976 | | 1.411861 | | FALSE | 0.5 | TRUE | Amp | N |
| F6 | RPA1 | SYBR | None | 17.759 | -0.6798 | | 0.233847 | | 0.850364 | | FALSE | 0.5 | TRUE | Amp | N |
| F7 | RPA3 | SYBR | None | 24.287 | 5.8482 | | 0.502945 | | 0.705665 | | FALSE | 0.5 | TRUE | Amp | N |
| F8 | SLK | SYBR | None | 20.782 | 2.3432 | | -0.21568 | | 1.161249 | | FALSE | 0.5 | TRUE | Amp | N |
| F9 | SMUG1 | SYBR | None | 26.692 | 8.2532 | | -0.21935 | | 1.16421 | | FALSE | 0.5 | TRUE | Amp | N |
| F10 | TDG | SYBR | None | 21.128 | 2.6892 | | -0.91142 | | 1.880891 | | FALSE | 0.5 | TRUE | Amp | N |
| F11 | TOP3A | SYBR | None | 25.656 | 7.2172 | | 0.533029 | | 0.691102 | | FALSE | 0.5 | TRUE | Amp | N |
| F12 | TOP3B | SYBR | None | 26.975 | 8.5362 | | -0.71328 | | 1.63953 | | FALSE | 0.5 | TRUE | Amp | N |
| G1 | TREX1 | SYBR | None | 27.872 | 9.4332 | | -0.20036 | | 1.148983 | | FALSE | 0.5 | TRUE | Amp | N |
| G2 | UNG | SYBR | None | 19.594 | 1.1552 | | -0.39429 | | 1.314294 | | FALSE | 0.5 | TRUE | Amp | N |
| G3 | XAB2 | SYBR | None | 23.359 | 4.9202 | | -0.4739 | | 1.388862 | | FALSE | 0.5 | TRUE | Amp | N |
| G4 | XPA | SYBR | None | 30.972 | 12.5332 | | -0.52028 | | 1.43423 | | FALSE | 0.5 | TRUE | Amp | N |
| G5 | XPC | SYBR | None | 25.426 | 6.9872 | | -0.11355 | | 1.081885 | | FALSE | 0.5 | TRUE | Amp | N |
| G6 | XRCC1 | SYBR | None | 20.893 | 2.4542 | | 0.176098 | | 0.885094 | | FALSE | 0.5 | TRUE | Amp | N |
| G7 | XRCC2 | SYBR | None | 21.587 | 3.1482 | | -0.25389 | | 1.192416 | | FALSE | 0.5 | TRUE | Amp | N |
| G8 | XRCC3 | SYBR | None | 23.419 | 4.9802 | | -0.47267 | | 1.387678 | | FALSE | 0.5 | TRUE | Amp | N |
| G9 | XRCC4 | SYBR | None | 25.238 | 6.7992 | | 0.10911 | | 0.92716 | | FALSE | 0.5 | TRUE | Amp | N |
| G10 | XRCC5 | SYBR | None | 17.926 | -0.5128 | | 0.306307 | | 0.808709 | | FALSE | 0.5 | TRUE | Amp | N |
| G11 | XRCC6 | SYBR | None | 19.354 | 0.9152 | | -0.19041 | | 1.141085 | | FALSE | 0.5 | TRUE | Amp | N |
| G12 | XRCC6BP1 | SYBR | None | 23.474 | 5.0352 | | -0.49892 | | 1.413155 | | FALSE | 0.5 | TRUE | Amp | N |
| H1 | ACTB | SYBR | None | 18.625 |  | |  | |  | | FALSE | 0.5 | TRUE | Amp | N |
| H2 | B2M | SYBR | None | 16.91 |  | |  | |  | | FALSE | 0.5 | TRUE | Amp | N |
| H3 | GAPDH | SYBR | None | 20.634 |  | |  | |  | | FALSE | 0.5 | TRUE | Amp | N |
| H4 | HPRT1 | SYBR | None | 20.719 |  | |  | |  | | FALSE | 0.5 | TRUE | Amp | N |
| H5 | RPLP0 | SYBR | None | 15.306 |  | |  | |  | | FALSE | 0.5 | TRUE | Amp | N |
| POI 30 |  |  |  |  |  | |  | |  | |  |  |  |  |  |
| Well Position | Gene Symbol | Reporter | Quencher | CT | Delta Ct | | Delta Delta Ct | | 2^(-Delta Delta Ct) | | Automatic Ct Threshold | Ct Threshold | Automatic Baseline | Amp Status | MTP |
| A1 | APEX1 | SYBR | None | 19.351 | 0.358 | | 0.407597 | | 0.753878 | | FALSE | 0.5 | TRUE | Amp | N |
| A2 | APEX2 | SYBR | None | 23.209 | 4.216 | | 0.480628 | | 0.716666 | | FALSE | 0.5 | TRUE | Amp | N |
| A3 | ATM | SYBR | None | 24.089 | 5.096 | | -0.04837 | | 1.034096 | | FALSE | 0.5 | TRUE | Amp | N |
| A4 | ATR | SYBR | None | 23.454 | 4.461 | | -0.04509 | | 1.031749 | | FALSE | 0.5 | TRUE | Amp | N |
| A5 | ATXN3 | SYBR | None | 20.843 | 1.85 | | -0.37002 | | 1.292369 | | FALSE | 0.5 | TRUE | Amp | N |
| A6 | BRCA1 | SYBR | None | 22.592 | 3.599 | | 0.351398 | | 0.783824 | | FALSE | 0.5 | TRUE | Amp | N |
| A7 | BRCA2 | SYBR | None | 19.624 | 0.631 | | 0.18705 | | 0.8784 | | FALSE | 0.5 | TRUE | Amp | N |
| A8 | BRIP1 | SYBR | None | 23.887 | 4.894 | | 0.160491 | | 0.89472 | | FALSE | 0.5 | TRUE | Amp | N |
| A9 | CCNH | SYBR | None | 25.07 | 6.077 | | -0.47209 | | 1.387118 | | FALSE | 0.5 | TRUE | Amp | N |
| A10 | CCNO | SYBR | None | 23.059 | 4.066 | | -0.17275 | | 1.127205 | | FALSE | 0.5 | TRUE | Amp | N |
| A11 | CDK7 | SYBR | None | 15.144 | -3.849 | | 0.3024 | | 0.810902 | | FALSE | 0.5 | TRUE | Amp | N |
| A12 | DDB1 | SYBR | None | 21.879 | 2.886 | | 0.529038 | | 0.693017 | | FALSE | 0.5 | TRUE | Amp | N |
| B1 | DDB2 | SYBR | None | 23.789 | 4.796 | | -0.09897 | | 1.07101 | | FALSE | 0.5 | TRUE | Amp | N |
| B2 | DMC1 | SYBR | None | 27.65 | 8.657 | | -0.41635 | | 1.334551 | | FALSE | 0.5 | TRUE | Amp | N |
| B3 | ERCC1 | SYBR | None | 23.383 | 4.39 | | 0.545608 | | 0.685103 | | FALSE | 0.5 | TRUE | Amp | N |
| B4 | ERCC2 | SYBR | None | 24.634 | 5.641 | | -0.07713 | | 1.054914 | | FALSE | 0.5 | TRUE | Amp | N |
| B5 | ERCC3 | SYBR | None | 22.381 | 3.388 | | 0.177677 | | 0.884125 | | FALSE | 0.5 | TRUE | Amp | N |
| B6 | ERCC4 | SYBR | None | 28.244 | 9.251 | | -0.11264 | | 1.081205 | | FALSE | 0.5 | TRUE | Amp | N |
| B7 | ERCC5 | SYBR | None | 26.303 | 7.31 | | 0.618672 | | 0.65127 | | FALSE | 0.5 | TRUE | Amp | N |
| B8 | ERCC6 | SYBR | None | 18.982 | -0.011 | | 0.491175 | | 0.711446 | | FALSE | 0.5 | TRUE | Amp | N |
| B9 | ERCC8 | SYBR | None | 23.215 | 4.222 | | -0.15535 | | 1.113693 | | FALSE | 0.5 | TRUE | Amp | N |
| B10 | EXO1 | SYBR | None | 21.457 | 2.464 | | 0.53836 | | 0.688553 | | FALSE | 0.5 | TRUE | Amp | N |
| B11 | FEN1 | SYBR | None | 17.925 | -1.068 | | -0.38825 | | 1.308804 | | FALSE | 0.5 | TRUE | Amp | N |
| B12 | LIG1 | SYBR | None | 22.858 | 3.865 | | 0.148799 | | 0.902001 | | FALSE | 0.5 | TRUE | Amp | N |
| C1 | LIG3 | SYBR | None | 26.31 | 7.317 | | 0.204221 | | 0.868007 | | FALSE | 0.5 | TRUE | Amp | N |
| C2 | LIG4 | SYBR | None | 26.389 | 7.396 | | 0.563101 | | 0.676846 | | FALSE | 0.5 | TRUE | Amp | N |
| C3 | MGMT | SYBR | None | 22.683 | 3.69 | | 0.525802 | | 0.694573 | | FALSE | 0.5 | TRUE | Amp | N |
| C4 | MLH1 | SYBR | None | 23.113 | 4.12 | | 0.163927 | | 0.892592 | | FALSE | 0.5 | TRUE | Amp | N |
| C5 | MLH3 | SYBR | None | 25.637 | 6.644 | | -0.1027 | | 1.07378 | | FALSE | 0.5 | TRUE | Amp | N |
| C6 | MMS19 | SYBR | None | 26 | 7.007 | | -0.37695 | | 1.298591 | | FALSE | 0.5 | TRUE | Amp | N |
| C7 | MPG | SYBR | None | 21.382 | 2.389 | | -0.68357 | | 1.606112 | | FALSE | 0.5 | TRUE | Amp | N |
| C8 | MRE11A | SYBR | None | 19.294 | 0.301 | | 0.365377 | | 0.776266 | | FALSE | 0.5 | TRUE | Amp | N |
| C9 | MSH2 | SYBR | None | 17.333 | -1.66 | | -0.02912 | | 1.020393 | | FALSE | 0.5 | TRUE | Amp | N |
| C10 | MSH3 | SYBR | None | 20.859 | 1.866 | | 0.506623 | | 0.703868 | | FALSE | 0.5 | TRUE | Amp | N |
| C11 | MSH4 | SYBR | None | 21.377 | 2.384 | | -0.14741 | | 1.107578 | | FALSE | 0.5 | TRUE | Amp | N |
| C12 | MSH5 | SYBR | None | 28.932 | 9.939 | | -0.41665 | | 1.334826 | | FALSE | 0.5 | TRUE | Amp | N |
| D1 | MSH6 | SYBR | None | 20.864 | 1.871 | | -0.53042 | | 1.444348 | | FALSE | 0.5 | TRUE | Amp | N |
| D2 | MUTYH | SYBR | None | 24.256 | 5.263 | | -0.14865 | | 1.108534 | | FALSE | 0.5 | TRUE | Amp | N |
| D3 | NEIL1 | SYBR | None | 27.152 | 8.159 | | -0.4642 | | 1.379551 | | FALSE | 0.5 | TRUE | Amp | N |
| D4 | NEIL2 | SYBR | None | 24.737 | 5.744 | | 0.173392 | | 0.886755 | | FALSE | 0.5 | TRUE | Amp | N |
| D5 | NEIL3 | SYBR | None | 21.853 | 2.86 | | -0.0035 | | 1.002429 | | FALSE | 0.5 | TRUE | Amp | N |
| D6 | NTHL1 | SYBR | None | 21.216 | 2.223 | | -0.37534 | | 1.297146 | | FALSE | 0.5 | TRUE | Amp | N |
| D7 | OGG1 | SYBR | None | 22.457 | 3.464 | | -0.13248 | | 1.09618 | | FALSE | 0.5 | TRUE | Amp | N |
| D8 | PARP1 | SYBR | None | 22.171 | 3.178 | | 0.501664 | | 0.706292 | | FALSE | 0.5 | TRUE | Amp | N |
| D9 | PARP2 | SYBR | None | 21.625 | 2.632 | | -0.08126 | | 1.057942 | | FALSE | 0.5 | TRUE | Amp | N |
| D10 | PARP3 | SYBR | None | 25.287 | 6.294 | | 0.562967 | | 0.676909 | | FALSE | 0.5 | TRUE | Amp | N |
| D11 | PMS1 | SYBR | None | 19.404 | 0.411 | | 0.507032 | | 0.703669 | | FALSE | 0.5 | TRUE | Amp | N |
| D12 | PMS2 | SYBR | None | 23.318 | 4.325 | | 0.48357 | | 0.715206 | | FALSE | 0.5 | TRUE | Amp | N |
| E1 | PNKP | SYBR | None | 24.755 | 5.762 | | -0.45883 | | 1.374425 | | FALSE | 0.5 | TRUE | Amp | N |
| E2 | POLB | SYBR | None | 18.759 | -0.234 | | 0.411533 | | 0.751824 | | FALSE | 0.5 | TRUE | Amp | N |
| E3 | POLD3 | SYBR | None | 20.662 | 1.669 | | -0.09586 | | 1.068699 | | FALSE | 0.5 | TRUE | Amp | N |
| E4 | POLL | SYBR | None | 26.07 | 7.077 | | 0.560326 | | 0.678149 | | FALSE | 0.5 | TRUE | Amp | N |
| E5 | PRKDC | SYBR | None | 24.325 | 5.332 | | -0.12707 | | 1.092075 | | FALSE | 0.5 | TRUE | Amp | N |
| E6 | RAD18 | SYBR | None | 18.437 | -0.556 | | -0.12398 | | 1.089738 | | FALSE | 0.5 | TRUE | Amp | N |
| E7 | RAD21 | SYBR | None | 19.313 | 0.32 | | 0.184706 | | 0.879828 | | FALSE | 0.5 | TRUE | Amp | N |
| E8 | RAD23A | SYBR | None | 22.713 | 3.72 | | -0.41259 | | 1.331074 | | FALSE | 0.5 | TRUE | Amp | N |
| E9 | RAD23B | SYBR | None | 25.858 | 6.865 | | 0.570782 | | 0.673252 | | FALSE | 0.5 | TRUE | Amp | N |
| E10 | RAD50 | SYBR | None | 20.605 | 1.612 | | -0.00407 | | 1.002822 | | FALSE | 0.5 | TRUE | Amp | N |
| E11 | RAD51 | SYBR | None | 21.652 | 2.659 | | -0.38181 | | 1.302973 | | FALSE | 0.5 | TRUE | Amp | N |
| E12 | RAD51B | SYBR | None | 21.307 | 2.314 | | 0.492175 | | 0.710952 | | FALSE | 0.5 | TRUE | Amp | N |
| F1 | RAD51C | SYBR | None | 20.152 | 1.159 | | 0.451622 | | 0.73122 | | FALSE | 0.5 | TRUE | Amp | N |
| F2 | RAD51D | SYBR | None | 24.136 | 5.143 | | -0.37921 | | 1.300628 | | FALSE | 0.5 | TRUE | Amp | N |
| F3 | RAD52 | SYBR | None | 26.01 | 7.017 | | -0.58313 | | 1.498094 | | FALSE | 0.5 | TRUE | Amp | N |
| F4 | RAD54L | SYBR | None | 23.216 | 4.223 | | -0.17078 | | 1.125669 | | FALSE | 0.5 | TRUE | Amp | N |
| F5 | RFC1 | SYBR | None | 24.166 | 5.173 | | -0.3818 | | 1.302964 | | FALSE | 0.5 | TRUE | Amp | N |
| F6 | RPA1 | SYBR | None | 18.521 | -0.472 | | 0.441647 | | 0.736294 | | FALSE | 0.5 | TRUE | Amp | N |
| F7 | RPA3 | SYBR | None | 24.265 | 5.272 | | -0.07325 | | 1.052087 | | FALSE | 0.5 | TRUE | Amp | N |
| F8 | SLK | SYBR | None | 21.135 | 2.142 | | -0.41688 | | 1.335035 | | FALSE | 0.5 | TRUE | Amp | N |
| F9 | SMUG1 | SYBR | None | 28.082 | 9.089 | | 0.616449 | | 0.652274 | | FALSE | 0.5 | TRUE | Amp | N |
| F10 | TDG | SYBR | None | 22.055 | 3.062 | | -0.53862 | | 1.452578 | | FALSE | 0.5 | TRUE | Amp | N |
| F11 | TOP3A | SYBR | None | 25.849 | 6.856 | | 0.171829 | | 0.887717 | | FALSE | 0.5 | TRUE | Amp | N |
| F12 | TOP3B | SYBR | None | 27.72 | 8.727 | | -0.52248 | | 1.436425 | | FALSE | 0.5 | TRUE | Amp | N |
| G1 | TREX1 | SYBR | None | 28.045 | 9.052 | | -0.58156 | | 1.496464 | | FALSE | 0.5 | TRUE | Amp | N |
| G2 | UNG | SYBR | None | 20.51 | 1.517 | | -0.03249 | | 1.022774 | | FALSE | 0.5 | TRUE | Amp | N |
| G3 | XAB2 | SYBR | None | 24.648 | 5.655 | | 0.260896 | | 0.834569 | | FALSE | 0.5 | TRUE | Amp | N |
| G4 | XPA | SYBR | None | 31.524 | 12.531 | | -0.52248 | | 1.436418 | | FALSE | 0.5 | TRUE | Amp | N |
| G5 | XPC | SYBR | None | 25.696 | 6.703 | | -0.39775 | | 1.31745 | | FALSE | 0.5 | TRUE | Amp | N |
| G6 | XRCC1 | SYBR | None | 21.828 | 2.835 | | 0.556898 | | 0.679762 | | FALSE | 0.5 | TRUE | Amp | N |
| G7 | XRCC2 | SYBR | None | 21.977 | 2.984 | | -0.41809 | | 1.336155 | | FALSE | 0.5 | TRUE | Amp | N |
| G8 | XRCC3 | SYBR | None | 24.432 | 5.439 | | -0.01387 | | 1.009662 | | FALSE | 0.5 | TRUE | Amp | N |
| G9 | XRCC4 | SYBR | None | 25.641 | 6.648 | | -0.04209 | | 1.029604 | | FALSE | 0.5 | TRUE | Amp | N |
| G10 | XRCC5 | SYBR | None | 17.804 | -1.189 | | -0.36989 | | 1.292257 | | FALSE | 0.5 | TRUE | Amp | N |
| G11 | XRCC6 | SYBR | None | 20.304 | 1.311 | | 0.205394 | | 0.867302 | | FALSE | 0.5 | TRUE | Amp | N |
| G12 | XRCC6BP1 | SYBR | None | 24.125 | 5.132 | | -0.40212 | | 1.321448 | | FALSE | 0.5 | TRUE | Amp | N |
| H1 | ACTB | SYBR | None | 19.159 |  | |  | |  | | FALSE | 0.5 | TRUE | Amp | N |
| H2 | B2M | SYBR | None | 17.444 |  | |  | |  | | FALSE | 0.5 | TRUE | Amp | N |
| H3 | GAPDH | SYBR | None | 21.536 |  | |  | |  | | FALSE | 0.5 | TRUE | Amp | N |
| H4 | HPRT1 | SYBR | None | 21.104 |  | |  | |  | | FALSE | 0.5 | TRUE | Amp | N |
| H5 | RPLP0 | SYBR | None | 15.722 |  | |  | |  | | FALSE | 0.5 | TRUE | Amp | N |
| POI 31 |  |  |  |  |  | |  | |  | |  |  |  |  |  |
| Well Position | Gene Symbol | Reporter | Quencher | CT | Delta Ct | | Delta Delta Ct | | 2^(-Delta Delta Ct) | | Automatic Ct Threshold | Ct Threshold | Automatic Baseline | Amp Status | MTP |
| A1 | APEX1 | SYBR | None | 18.388 | -0.1864 | | -0.1368 | | 1.099466 | | FALSE | 0.5 | TRUE | Amp | N |
| A2 | APEX2 | SYBR | None | 22.85 | 4.2756 | | 0.540228 | | 0.687662 | | FALSE | 0.5 | TRUE | Amp | N |
| A3 | ATM | SYBR | None | 24.307 | 5.7326 | | 0.588229 | | 0.665159 | | FALSE | 0.5 | TRUE | Amp | N |
| A4 | ATR | SYBR | None | 22.629 | 4.0546 | | -0.45149 | | 1.367453 | | FALSE | 0.5 | TRUE | Amp | N |
| A5 | ATXN3 | SYBR | None | 20.676 | 2.1016 | | -0.11842 | | 1.085544 | | FALSE | 0.5 | TRUE | Amp | N |
| A6 | BRCA1 | SYBR | None | 21.653 | 3.0786 | | -0.169 | | 1.124281 | | FALSE | 0.5 | TRUE | Amp | N |
| A7 | BRCA2 | SYBR | None | 19.18 | 0.6056 | | 0.16165 | | 0.894002 | | FALSE | 0.5 | TRUE | Amp | N |
| A8 | BRIP1 | SYBR | None | 23.173 | 4.5986 | | -0.13491 | | 1.098023 | | FALSE | 0.5 | TRUE | Amp | N |
| A9 | CCNH | SYBR | None | 24.915 | 6.3406 | | -0.20849 | | 1.155479 | | FALSE | 0.5 | TRUE | Amp | N |
| A10 | CCNO | SYBR | None | 22.987 | 4.4126 | | 0.17385 | | 0.886474 | | FALSE | 0.5 | TRUE | Amp | N |
| A11 | CDK7 | SYBR | None | 14.352 | -4.2224 | | -0.071 | | 1.050445 | | FALSE | 0.5 | TRUE | Amp | N |
| A12 | DDB1 | SYBR | None | 20.444 | 1.8696 | | -0.48736 | | 1.401879 | | FALSE | 0.5 | TRUE | Amp | N |
| B1 | DDB2 | SYBR | None | 23.752 | 5.1776 | | 0.282629 | | 0.822092 | | FALSE | 0.5 | TRUE | Amp | N |
| B2 | DMC1 | SYBR | None | 26.97 | 8.3956 | | -0.67775 | | 1.599648 | | FALSE | 0.5 | TRUE | Amp | N |
| B3 | ERCC1 | SYBR | None | 22.321 | 3.7466 | | -0.09779 | | 1.070135 | | FALSE | 0.5 | TRUE | Amp | N |
| B4 | ERCC2 | SYBR | None | 24.449 | 5.8746 | | 0.156474 | | 0.897215 | | FALSE | 0.5 | TRUE | Amp | N |
| B5 | ERCC3 | SYBR | None | 21.325 | 2.7506 | | -0.45972 | | 1.375278 | | FALSE | 0.5 | TRUE | Amp | N |
| B6 | ERCC4 | SYBR | None | 27.736 | 9.1616 | | -0.20204 | | 1.150323 | | FALSE | 0.5 | TRUE | Amp | N |
| B7 | ERCC5 | SYBR | None | 25.815 | 7.2406 | | 0.549272 | | 0.683365 | | FALSE | 0.5 | TRUE | Amp | N |
| B8 | ERCC6 | SYBR | None | 18.492 | -0.0824 | | 0.419775 | | 0.747541 | | FALSE | 0.5 | TRUE | Amp | N |
| B9 | ERCC8 | SYBR | None | 23.161 | 4.5866 | | 0.209249 | | 0.864988 | | FALSE | 0.5 | TRUE | Amp | N |
| B10 | EXO1 | SYBR | None | 20.443 | 1.8686 | | -0.05704 | | 1.040329 | | FALSE | 0.5 | TRUE | Amp | N |
| B11 | FEN1 | SYBR | None | 18.153 | -0.4214 | | 0.258351 | | 0.836043 | | FALSE | 0.5 | TRUE | Amp | N |
| B12 | LIG1 | SYBR | None | 21.931 | 3.3566 | | -0.3596 | | 1.283071 | | FALSE | 0.5 | TRUE | Amp | N |
| C1 | LIG3 | SYBR | None | 25.256 | 6.6816 | | -0.43118 | | 1.348335 | | FALSE | 0.5 | TRUE | Amp | N |
| C2 | LIG4 | SYBR | None | 26.026 | 7.4516 | | 0.618701 | | 0.651257 | | FALSE | 0.5 | TRUE | Amp | N |
| C3 | MGMT | SYBR | None | 21.198 | 2.6236 | | -0.5406 | | 1.454575 | | FALSE | 0.5 | TRUE | Amp | N |
| C4 | MLH1 | SYBR | None | 21.999 | 3.4246 | | -0.53147 | | 1.445405 | | FALSE | 0.5 | TRUE | Amp | N |
| C5 | MLH3 | SYBR | None | 24.889 | 6.3146 | | -0.4321 | | 1.349194 | | FALSE | 0.5 | TRUE | Amp | N |
| C6 | MMS19 | SYBR | None | 25.311 | 6.7366 | | -0.64735 | | 1.566285 | | FALSE | 0.5 | TRUE | Amp | N |
| C7 | MPG | SYBR | None | 22.186 | 3.6116 | | 0.539028 | | 0.688235 | | FALSE | 0.5 | TRUE | Amp | N |
| C8 | MRE11A | SYBR | None | 18.742 | 0.1676 | | 0.231977 | | 0.851467 | | FALSE | 0.5 | TRUE | Amp | N |
| C9 | MSH2 | SYBR | None | 17.188 | -1.3864 | | 0.244475 | | 0.844123 | | FALSE | 0.5 | TRUE | Amp | N |
| C10 | MSH3 | SYBR | None | 20.165 | 1.5906 | | 0.231223 | | 0.851912 | | FALSE | 0.5 | TRUE | Amp | N |
| C11 | MSH4 | SYBR | None | 20.885 | 2.3106 | | -0.22081 | | 1.165386 | | FALSE | 0.5 | TRUE | Amp | N |
| C12 | MSH5 | SYBR | None | 28.426 | 9.8516 | | -0.50405 | | 1.418191 | | FALSE | 0.5 | TRUE | Amp | N |
| D1 | MSH6 | SYBR | None | 20.84 | 2.2656 | | -0.13582 | | 1.098716 | | FALSE | 0.5 | TRUE | Amp | N |
| D2 | MUTYH | SYBR | None | 23.433 | 4.8586 | | -0.55305 | | 1.467187 | | FALSE | 0.5 | TRUE | Amp | N |
| D3 | NEIL1 | SYBR | None | 27.302 | 8.7276 | | 0.104401 | | 0.930191 | | FALSE | 0.5 | TRUE | Amp | N |
| D4 | NEIL2 | SYBR | None | 23.522 | 4.9476 | | -0.62301 | | 1.540083 | | FALSE | 0.5 | TRUE | Amp | N |
| D5 | NEIL3 | SYBR | None | 21.287 | 2.7126 | | -0.1509 | | 1.110262 | | FALSE | 0.5 | TRUE | Amp | N |
| D6 | NTHL1 | SYBR | None | 20.649 | 2.0746 | | -0.52374 | | 1.437678 | | FALSE | 0.5 | TRUE | Amp | N |
| D7 | OGG1 | SYBR | None | 21.673 | 3.0986 | | -0.49788 | | 1.412142 | | FALSE | 0.5 | TRUE | Amp | N |
| D8 | PARP1 | SYBR | None | 21.661 | 3.0866 | | 0.410264 | | 0.752486 | | FALSE | 0.5 | TRUE | Amp | N |
| D9 | PARP2 | SYBR | None | 21.426 | 2.8516 | | 0.13834 | | 0.908564 | | FALSE | 0.5 | TRUE | Amp | N |
| D10 | PARP3 | SYBR | None | 23.872 | 5.2976 | | -0.43343 | | 1.350443 | | FALSE | 0.5 | TRUE | Amp | N |
| D11 | PMS1 | SYBR | None | 18.865 | 0.2906 | | 0.386632 | | 0.764913 | | FALSE | 0.5 | TRUE | Amp | N |
| D12 | PMS2 | SYBR | None | 22.934 | 4.3596 | | 0.51817 | | 0.698257 | | FALSE | 0.5 | TRUE | Amp | N |
| E1 | PNKP | SYBR | None | 24.687 | 6.1126 | | -0.10823 | | 1.077904 | | FALSE | 0.5 | TRUE | Amp | N |
| E2 | POLB | SYBR | None | 17.818 | -0.7564 | | -0.11087 | | 1.079877 | | FALSE | 0.5 | TRUE | Amp | N |
| E3 | POLD3 | SYBR | None | 20.783 | 2.2086 | | 0.443744 | | 0.735224 | | FALSE | 0.5 | TRUE | Amp | N |
| E4 | POLL | SYBR | None | 24.576 | 6.0016 | | -0.51507 | | 1.429067 | | FALSE | 0.5 | TRUE | Amp | N |
| E5 | PRKDC | SYBR | None | 24.531 | 5.9566 | | 0.497528 | | 0.708319 | | FALSE | 0.5 | TRUE | Amp | N |
| E6 | RAD18 | SYBR | None | 17.742 | -0.8324 | | -0.40038 | | 1.319857 | | FALSE | 0.5 | TRUE | Amp | N |
| E7 | RAD21 | SYBR | None | 19.096 | 0.5216 | | 0.386306 | | 0.765086 | | FALSE | 0.5 | TRUE | Amp | N |
| E8 | RAD23A | SYBR | None | 23.236 | 4.6616 | | 0.529009 | | 0.693031 | | FALSE | 0.5 | TRUE | Amp | N |
| E9 | RAD23B | SYBR | None | 25.415 | 6.8406 | | 0.546382 | | 0.684735 | | FALSE | 0.5 | TRUE | Amp | N |
| E10 | RAD50 | SYBR | None | 19.71 | 1.1356 | | -0.48047 | | 1.395194 | | FALSE | 0.5 | TRUE | Amp | N |
| E11 | RAD51 | SYBR | None | 21.608 | 3.0336 | | -0.00721 | | 1.005008 | | FALSE | 0.5 | TRUE | Amp | N |
| E12 | RAD51B | SYBR | None | 20.341 | 1.7666 | | -0.05522 | | 1.039021 | | FALSE | 0.5 | TRUE | Amp | N |
| F1 | RAD51C | SYBR | None | 19.499 | 0.9246 | | 0.217222 | | 0.86022 | | FALSE | 0.5 | TRUE | Amp | N |
| F2 | RAD51D | SYBR | None | 23.437 | 4.8626 | | -0.65961 | | 1.579654 | | FALSE | 0.5 | TRUE | Amp | N |
| F3 | RAD52 | SYBR | None | 26.806 | 8.2316 | | 0.631472 | | 0.645517 | | FALSE | 0.5 | TRUE | Amp | N |
| F4 | RAD54L | SYBR | None | 22.319 | 3.7446 | | -0.64918 | | 1.56828 | | FALSE | 0.5 | TRUE | Amp | N |
| F5 | RFC1 | SYBR | None | 23.694 | 5.1196 | | -0.4352 | | 1.352096 | | FALSE | 0.5 | TRUE | Amp | N |
| F6 | RPA1 | SYBR | None | 18.002 | -0.5724 | | 0.341247 | | 0.789359 | | FALSE | 0.5 | TRUE | Amp | N |
| F7 | RPA3 | SYBR | None | 23.887 | 5.3126 | | -0.03265 | | 1.022892 | | FALSE | 0.5 | TRUE | Amp | N |
| F8 | SLK | SYBR | None | 21.054 | 2.4796 | | -0.07928 | | 1.056489 | | FALSE | 0.5 | TRUE | Amp | N |
| F9 | SMUG1 | SYBR | None | 26.555 | 7.9806 | | -0.49195 | | 1.406345 | | FALSE | 0.5 | TRUE | Amp | N |
| F10 | TDG | SYBR | None | 21.75 | 3.1756 | | -0.42502 | | 1.342587 | | FALSE | 0.5 | TRUE | Amp | N |
| F11 | TOP3A | SYBR | None | 25.847 | 7.2726 | | 0.588429 | | 0.665067 | | FALSE | 0.5 | TRUE | Amp | N |
| F12 | TOP3B | SYBR | None | 27.765 | 9.1906 | | -0.05888 | | 1.041659 | | FALSE | 0.5 | TRUE | Amp | N |
| G1 | TREX1 | SYBR | None | 28.061 | 9.4866 | | -0.14696 | | 1.107232 | | FALSE | 0.5 | TRUE | Amp | N |
| G2 | UNG | SYBR | None | 19.93 | 1.3556 | | -0.19389 | | 1.143842 | | FALSE | 0.5 | TRUE | Amp | N |
| G3 | XAB2 | SYBR | None | 24.105 | 5.5306 | | 0.136496 | | 0.909726 | | FALSE | 0.5 | TRUE | Amp | N |
| G4 | XPA | SYBR | None | 31.6 | 13.0256 | | -0.02788 | | 1.01951 | | FALSE | 0.5 | TRUE | Amp | N |
| G5 | XPC | SYBR | None | 25.08 | 6.5056 | | -0.59515 | | 1.510627 | | FALSE | 0.5 | TRUE | Amp | N |
| G6 | XRCC1 | SYBR | None | 20.372 | 1.7976 | | -0.4805 | | 1.39523 | | FALSE | 0.5 | TRUE | Amp | N |
| G7 | XRCC2 | SYBR | None | 21.945 | 3.3706 | | -0.03149 | | 1.022066 | | FALSE | 0.5 | TRUE | Amp | N |
| G8 | XRCC3 | SYBR | None | 23.423 | 4.8486 | | -0.60427 | | 1.520212 | | FALSE | 0.5 | TRUE | Amp | N |
| G9 | XRCC4 | SYBR | None | 25.172 | 6.5976 | | -0.09249 | | 1.066209 | | FALSE | 0.5 | TRUE | Amp | N |
| G10 | XRCC5 | SYBR | None | 17.983 | -0.5914 | | 0.227707 | | 0.853991 | | FALSE | 0.5 | TRUE | Amp | N |
| G11 | XRCC6 | SYBR | None | 19.166 | 0.5916 | | -0.51401 | | 1.42801 | | FALSE | 0.5 | TRUE | Amp | N |
| G12 | XRCC6BP1 | SYBR | None | 23.895 | 5.3206 | | -0.21352 | | 1.159514 | | FALSE | 0.5 | TRUE | Amp | N |
| H1 | ACTB | SYBR | None | 18.603 |  | |  | |  | | FALSE | 0.5 | TRUE | Amp | N |
| H2 | B2M | SYBR | None | 17.021 |  | |  | |  | | FALSE | 0.5 | TRUE | Amp | N |
| H3 | GAPDH | SYBR | None | 20.775 |  | |  | |  | | FALSE | 0.5 | TRUE | Amp | N |
| H4 | HPRT1 | SYBR | None | 20.994 |  | |  | |  | | FALSE | 0.5 | TRUE | Amp | N |
| H5 | RPLP0 | SYBR | None | 15.479 |  | |  | |  | | FALSE | 0.5 | TRUE | Amp | N |
| POI 32 |  |  |  |  |  | |  | |  | |  |  |  |  |  |
| Well Position | Gene Symbol | Reporter | Quencher | CT | Delta Ct | | Delta Delta Ct | | 2^(-Delta Delta Ct) | | Automatic Ct Threshold | Ct Threshold | Automatic Baseline | Amp Status | MTP |
| A1 | APEX1 | SYBR | None | 18.806 | -0.1104 | | -0.0608 | | 1.043046 | | FALSE | 0.5 | TRUE | Amp | N |
| A2 | APEX2 | SYBR | None | 23.228 | 4.3116 | | 0.576228 | | 0.670715 | | FALSE | 0.5 | TRUE | Amp | N |
| A3 | ATM | SYBR | None | 24.35 | 5.4336 | | 0.289229 | | 0.818339 | | FALSE | 0.5 | TRUE | Amp | N |
| A4 | ATR | SYBR | None | 23.082 | 4.1656 | | -0.34049 | | 1.266188 | | FALSE | 0.5 | TRUE | Amp | N |
| A5 | ATXN3 | SYBR | None | 21.625 | 2.7086 | | 0.488582 | | 0.712725 | | FALSE | 0.5 | TRUE | Amp | N |
| A6 | BRCA1 | SYBR | None | 22.425 | 3.5086 | | 0.260998 | | 0.83451 | | FALSE | 0.5 | TRUE | Amp | N |
| A7 | BRCA2 | SYBR | None | 19.319 | 0.4026 | | -0.04135 | | 1.029077 | | FALSE | 0.5 | TRUE | Amp | N |
| A8 | BRIP1 | SYBR | None | 23.884 | 4.9676 | | 0.234091 | | 0.85022 | | FALSE | 0.5 | TRUE | Amp | N |
| A9 | CCNH | SYBR | None | 25.03 | 6.1136 | | -0.43549 | | 1.352371 | | FALSE | 0.5 | TRUE | Amp | N |
| A10 | CCNO | SYBR | None | 23.134 | 4.2176 | | -0.02115 | | 1.014768 | | FALSE | 0.5 | TRUE | Amp | N |
| A11 | CDK7 | SYBR | None | 15.207 | -3.7094 | | 0.442 | | 0.736113 | | FALSE | 0.5 | TRUE | Amp | N |
| A12 | DDB1 | SYBR | None | 20.826 | 1.9096 | | -0.44736 | | 1.363545 | | FALSE | 0.5 | TRUE | Amp | N |
| B1 | DDB2 | SYBR | None | 23.443 | 4.5266 | | -0.36837 | | 1.290895 | | FALSE | 0.5 | TRUE | Amp | N |
| B2 | DMC1 | SYBR | None | 28.313 | 9.3966 | | 0.323246 | | 0.79927 | | FALSE | 0.5 | TRUE | Amp | N |
| B3 | ERCC1 | SYBR | None | 23.34 | 4.4236 | | 0.579208 | | 0.669331 | | FALSE | 0.5 | TRUE | Amp | N |
| B4 | ERCC2 | SYBR | None | 24.607 | 5.6906 | | -0.02753 | | 1.019263 | | FALSE | 0.5 | TRUE | Amp | N |
| B5 | ERCC3 | SYBR | None | 22.031 | 3.1146 | | -0.09572 | | 1.068601 | | FALSE | 0.5 | TRUE | Amp | N |
| B6 | ERCC4 | SYBR | None | 27.668 | 8.7516 | | -0.61204 | | 1.528418 | | FALSE | 0.5 | TRUE | Amp | N |
| B7 | ERCC5 | SYBR | None | 26.595 | 7.6786 | | 0.987272 | | 0.504431 | | FALSE | 0.5 | TRUE | Amp | N |
| B8 | ERCC6 | SYBR | None | 18.954 | 0.0376 | | 0.539775 | | 0.687878 | | FALSE | 0.5 | TRUE | Amp | N |
| B9 | ERCC8 | SYBR | None | 23.251 | 4.3346 | | -0.04275 | | 1.030076 | | FALSE | 0.5 | TRUE | Amp | N |
| B10 | EXO1 | SYBR | None | 21.092 | 2.1756 | | 0.24996 | | 0.84092 | | FALSE | 0.5 | TRUE | Amp | N |
| B11 | FEN1 | SYBR | None | 18.221 | -0.6954 | | -0.01565 | | 1.010906 | | FALSE | 0.5 | TRUE | Amp | N |
| B12 | LIG1 | SYBR | None | 22.919 | 4.0026 | | 0.286399 | | 0.819946 | | FALSE | 0.5 | TRUE | Amp | N |
| C1 | LIG3 | SYBR | None | 26.726 | 7.8096 | | 0.696821 | | 0.61693 | | FALSE | 0.5 | TRUE | Amp | N |
| C2 | LIG4 | SYBR | None | 26.37 | 7.4536 | | 0.620701 | | 0.650355 | | FALSE | 0.5 | TRUE | Amp | N |
| C3 | MGMT | SYBR | None | 21.566 | 2.6496 | | -0.5146 | | 1.428596 | | FALSE | 0.5 | TRUE | Amp | N |
| C4 | MLH1 | SYBR | None | 23.467 | 4.5506 | | 0.594527 | | 0.662262 | | FALSE | 0.5 | TRUE | Amp | N |
| C5 | MLH3 | SYBR | None | 25.275 | 6.3586 | | -0.3881 | | 1.308667 | | FALSE | 0.5 | TRUE | Amp | N |
| C6 | MMS19 | SYBR | None | 26.296 | 7.3796 | | -0.00435 | | 1.003018 | | FALSE | 0.5 | TRUE | Amp | N |
| C7 | MPG | SYBR | None | 21.756 | 2.8396 | | -0.23297 | | 1.175254 | | FALSE | 0.5 | TRUE | Amp | N |
| C8 | MRE11A | SYBR | None | 19.047 | 0.1306 | | 0.194977 | | 0.873587 | | FALSE | 0.5 | TRUE | Amp | N |
| C9 | MSH2 | SYBR | None | 17.719 | -1.1974 | | 0.433475 | | 0.740476 | | FALSE | 0.5 | TRUE | Amp | N |
| C10 | MSH3 | SYBR | None | 19.97 | 1.0536 | | -0.30578 | | 1.236084 | | FALSE | 0.5 | TRUE | Amp | N |
| C11 | MSH4 | SYBR | None | 21.41 | 2.4936 | | -0.03781 | | 1.026553 | | FALSE | 0.5 | TRUE | Amp | N |
| C12 | MSH5 | SYBR | None | 29.194 | 10.2776 | | -0.07805 | | 1.055591 | | FALSE | 0.5 | TRUE | Amp | N |
| D1 | MSH6 | SYBR | None | 21.205 | 2.2886 | | -0.11282 | | 1.081339 | | FALSE | 0.5 | TRUE | Amp | N |
| D2 | MUTYH | SYBR | None | 24.286 | 5.3696 | | -0.04205 | | 1.029578 | | FALSE | 0.5 | TRUE | Amp | N |
| D3 | NEIL1 | SYBR | None | 26.98 | 8.0636 | | -0.5596 | | 1.47386 | | FALSE | 0.5 | TRUE | Amp | N |
| D4 | NEIL2 | SYBR | None | 24.484 | 5.5676 | | -0.00301 | | 1.002087 | | FALSE | 0.5 | TRUE | Amp | N |
| D5 | NEIL3 | SYBR | None | 21.434 | 2.5176 | | -0.3459 | | 1.270944 | | FALSE | 0.5 | TRUE | Amp | N |
| D6 | NTHL1 | SYBR | None | 21.033 | 2.1166 | | -0.48174 | | 1.396427 | | FALSE | 0.5 | TRUE | Amp | N |
| D7 | OGG1 | SYBR | None | 22.435 | 3.5186 | | -0.07788 | | 1.055469 | | FALSE | 0.5 | TRUE | Amp | N |
| D8 | PARP1 | SYBR | None | 21.779 | 2.8626 | | 0.186264 | | 0.878879 | | FALSE | 0.5 | TRUE | Amp | N |
| D9 | PARP2 | SYBR | None | 22.235 | 3.3186 | | 0.60534 | | 0.657317 | | FALSE | 0.5 | TRUE | Amp | N |
| D10 | PARP3 | SYBR | None | 24.285 | 5.3686 | | -0.36243 | | 1.285592 | | FALSE | 0.5 | TRUE | Amp | N |
| D11 | PMS1 | SYBR | None | 19.214 | 0.2976 | | 0.393632 | | 0.761211 | | FALSE | 0.5 | TRUE | Amp | N |
| D12 | PMS2 | SYBR | None | 23.313 | 4.3966 | | 0.55517 | | 0.680577 | | FALSE | 0.5 | TRUE | Amp | N |
| E1 | PNKP | SYBR | None | 25.381 | 6.4646 | | 0.243771 | | 0.844535 | | FALSE | 0.5 | TRUE | Amp | N |
| E2 | POLB | SYBR | None | 18.206 | -0.7104 | | -0.06487 | | 1.045989 | | FALSE | 0.5 | TRUE | Amp | N |
| E3 | POLD3 | SYBR | None | 20.934 | 2.0176 | | 0.252744 | | 0.839299 | | FALSE | 0.5 | TRUE | Amp | N |
| E4 | POLL | SYBR | None | 24.766 | 5.8496 | | -0.66707 | | 1.587849 | | FALSE | 0.5 | TRUE | Amp | N |
| E5 | PRKDC | SYBR | None | 24.262 | 5.3456 | | -0.11347 | | 1.081828 | | FALSE | 0.5 | TRUE | Amp | N |
| E6 | RAD18 | SYBR | None | 18.013 | -0.9034 | | -0.47138 | | 1.386436 | | FALSE | 0.5 | TRUE | Amp | N |
| E7 | RAD21 | SYBR | None | 19.451 | 0.5346 | | 0.399306 | | 0.758223 | | FALSE | 0.5 | TRUE | Amp | N |
| E8 | RAD23A | SYBR | None | 23.454 | 4.5376 | | 0.405009 | | 0.755232 | | FALSE | 0.5 | TRUE | Amp | N |
| E9 | RAD23B | SYBR | None | 25.795 | 6.8786 | | 0.584382 | | 0.666935 | | FALSE | 0.5 | TRUE | Amp | N |
| E10 | RAD50 | SYBR | None | 20.17 | 1.2536 | | -0.36247 | | 1.285621 | | FALSE | 0.5 | TRUE | Amp | N |
| E11 | RAD51 | SYBR | None | 22.468 | 3.5516 | | 0.510793 | | 0.701837 | | FALSE | 0.5 | TRUE | Amp | N |
| E12 | RAD51B | SYBR | None | 21.289 | 2.3726 | | 0.550775 | | 0.682653 | | FALSE | 0.5 | TRUE | Amp | N |
| F1 | RAD51C | SYBR | None | 20.041 | 1.1246 | | 0.417222 | | 0.748865 | | FALSE | 0.5 | TRUE | Amp | N |
| F2 | RAD51D | SYBR | None | 24.037 | 5.1206 | | -0.40161 | | 1.32098 | | FALSE | 0.5 | TRUE | Amp | N |
| F3 | RAD52 | SYBR | None | 26.487 | 7.5706 | | -0.02953 | | 1.020678 | | FALSE | 0.5 | TRUE | Amp | N |
| F4 | RAD54L | SYBR | None | 23.004 | 4.0876 | | -0.30618 | | 1.236432 | | FALSE | 0.5 | TRUE | Amp | N |
| F5 | RFC1 | SYBR | None | 23.762 | 4.8456 | | -0.7092 | | 1.634895 | | FALSE | 0.5 | TRUE | Amp | N |
| F6 | RPA1 | SYBR | None | 18.302 | -0.6144 | | 0.299247 | | 0.812676 | | FALSE | 0.5 | TRUE | Amp | N |
| F7 | RPA3 | SYBR | None | 24.89 | 5.9736 | | 0.628345 | | 0.646918 | | FALSE | 0.5 | TRUE | Amp | N |
| F8 | SLK | SYBR | None | 21.418 | 2.5016 | | -0.05728 | | 1.0405 | | FALSE | 0.5 | TRUE | Amp | N |
| F9 | SMUG1 | SYBR | None | 27.98 | 9.0636 | | 0.591049 | | 0.66386 | | FALSE | 0.5 | TRUE | Amp | N |
| F10 | TDG | SYBR | None | 21.961 | 3.0446 | | -0.55602 | | 1.470204 | | FALSE | 0.5 | TRUE | Amp | N |
| F11 | TOP3A | SYBR | None | 26.196 | 7.2796 | | 0.595429 | | 0.661848 | | FALSE | 0.5 | TRUE | Amp | N |
| F12 | TOP3B | SYBR | None | 27.774 | 8.8576 | | -0.39188 | | 1.312105 | | FALSE | 0.5 | TRUE | Amp | N |
| G1 | TREX1 | SYBR | None | 27.904 | 8.9876 | | -0.64596 | | 1.564778 | | FALSE | 0.5 | TRUE | Amp | N |
| G2 | UNG | SYBR | None | 20.763 | 1.8466 | | 0.297112 | | 0.81388 | | FALSE | 0.5 | TRUE | Amp | N |
| G3 | XAB2 | SYBR | None | 24.192 | 5.2756 | | -0.1185 | | 1.085608 | | FALSE | 0.5 | TRUE | Amp | N |
| G4 | XPA | SYBR | None | 31.47 | 12.5536 | | -0.49988 | | 1.414092 | | FALSE | 0.5 | TRUE | Amp | N |
| G5 | XPC | SYBR | None | 25.506 | 6.5896 | | -0.51115 | | 1.425183 | | FALSE | 0.5 | TRUE | Amp | N |
| G6 | XRCC1 | SYBR | None | 21.758 | 2.8416 | | 0.563498 | | 0.67666 | | FALSE | 0.5 | TRUE | Amp | N |
| G7 | XRCC2 | SYBR | None | 22.776 | 3.8596 | | 0.457512 | | 0.728241 | | FALSE | 0.5 | TRUE | Amp | N |
| G8 | XRCC3 | SYBR | None | 24.298 | 5.3816 | | -0.07127 | | 1.050643 | | FALSE | 0.5 | TRUE | Amp | N |
| G9 | XRCC4 | SYBR | None | 25.927 | 7.0106 | | 0.32051 | | 0.800787 | | FALSE | 0.5 | TRUE | Amp | N |
| G10 | XRCC5 | SYBR | None | 18.424 | -0.4924 | | 0.326707 | | 0.797354 | | FALSE | 0.5 | TRUE | Amp | N |
| G11 | XRCC6 | SYBR | None | 20.239 | 1.3226 | | 0.216994 | | 0.860356 | | FALSE | 0.5 | TRUE | Amp | N |
| G12 | XRCC6BP1 | SYBR | None | 23.949 | 5.0326 | | -0.50152 | | 1.415705 | | FALSE | 0.5 | TRUE | Amp | N |
| H1 | ACTB | SYBR | None | 18.894 |  | |  | |  | | FALSE | 0.5 | TRUE | Amp | N |
| H2 | B2M | SYBR | None | 17.521 |  | |  | |  | | FALSE | 0.5 | TRUE | Amp | N |
| H3 | GAPDH | SYBR | None | 21.36 |  | |  | |  | | FALSE | 0.5 | TRUE | Amp | N |
| H4 | HPRT1 | SYBR | None | 20.934 |  | |  | |  | | FALSE | 0.5 | TRUE | Amp | N |
| H5 | RPLP0 | SYBR | None | 15.873 |  | |  | |  | | FALSE | 0.5 | TRUE | Amp | N |
